# Expression Profiles of Differentially Expressed Circular RNAs and circRNA–miRNA–mRNA Regulatory Networks in SH-SY5Y Cells Infected with Coxsackievirus B5

**DOI:** 10.1155/2022/9298149

**Published:** 2022-10-10

**Authors:** Jing Li, Heng Yang, Huaran Shi, Jihong Zhang, Wei Chen

**Affiliations:** ^1^Medical School, Kunming University of Science and Technology, Kunming, Yunnan Province, China; ^2^College of Agriculture and Life Sciences, Kunming University, Kunming, Yunnan Province, China

## Abstract

Coxsackievirus B5 (CVB5) is the causative agent of hand, foot, and mouth disease (HFMD) that can cause neurological complications and fatalities. Circular RNA (circRNA) has been shown to play an important role in regulating pathogenic processes. However, the functions of circRNA in response to CVB5 infection remain unclear. In our research, RNA-seq was employed to analyze the expression profiles of circRNAs in SH-SY5Y cells with or without CVB5 infection. Out of 5,665 circRNAs identified to be expressed in SH-SY5Y cells, 163 circRNAs were found to be differentially expressed significantly. Moreover, Gene Ontology (GO) and Kyoto Encyclopedia of Genes and Genomes (KEGG) analyses showed that the differentially expressed circRNAs were mainly involved in ubiquitin-mediated proteolysis and signaling pathways during CVB5 infection. Additionally, RT-qPCR was used to validate the RNA-seq data, and a circRNA–miRNA–mRNA interaction network was constructed based on two circRNAs, such as hsa_circ_0008378 and novel_circ_0014617, which were associated with the regulation of innate immune response in host cells. Additionally, we confirmed the two circRANs up-regulated the key factors in the IFN-I signaling pathway, hampering viral replication. Our data provide a new perspective that facilitates further understanding of the virus-host mechanism.

## 1. Introduction

Hand, foot, and mouth disease (HFMD) is an acute infectious disease which mainly affects infants under the age of five. The main manifestations of HFMD are fever and vesicular rashes on hands, feet, and buttocks. Previously, HFMD was mainly caused by enterovirus A71 (EV-A71) that posed serious threats in infants' health. With the recent development of EV-A71 vaccine, Coxsackievirus B5 (CVB5) has become one of the main pathogens of HFMD outbreaks globally [1, 2]. As one of the members of family *Picornaviridae*, CVB5 has a positive-sense single-stranded RNA. HFMD caused by CVB5 has also been found to be associated with neurological complications, including aseptic meningitis, viral encephalitis, and acute flaccid paralysis [3]. To date, there is no effective treatment for HFMD caused by CVB5. There are also very few studies that elucidated the pathogenesis mechanism caused by CVB5 infection.

Circular RNA (circRNA) is a noncoding and single-stranded RNA transcript that can form covalently circular-closed structures without 5′ end caps or 3′ Poly (A) tails. High-throughput RNA-seq studies have detected a large number of different types of circRNAs with varying lengths. Several biological processes, including gene regulation, protein assembly and trafficking, and cell division are strongly regulated by circRNAs [4, 5]. With the development of RNA-seq, many research groups have conducted studies to reveal the expression profiles of circRNA after the viral infection and better understand their pathogenesis [6, 7]. So far, large amounts of circRNAs have been successfully identified in a variety of virus infection models like Coxsackie Virus B3, Marek's disease virus, and Hepatitis B virus [8–10]. In addition, circRNAs have been shown to be widely expressed from humans to viruses as potential regulators of microRNAs (miRNAs) and RNA-binding proteins (RPBs) [11]. CircRNAs act as “miRNA sponges” and inhibit the binding of miRNAs to target genes, therefore regulating the expression of mRNAs in certain physiological activities and resulting diseases. For example, circRNAs, induced by rabies virus (RABV) infection, were mainly related to cell plasticity and synapse function and regulated RABV infection via interaction with miRNAs and mRNAs [12]. Circ_0000479 induced by Hantaan virus infection could indirectly regulate RIG-I expression by sponging miR-149-5p, hampering viral replication [13].

The roles of circRNAs and their functions during viral infection have gained much research attention; therefore, the functions and mechanisms of circRNA is of great significance for the prevention and treatment of CVB5. In our research, to better understand the association between CVB5 infection and circRNA expression, RNA-seq was employed to obtain the circRNAs expression profile in SH-SY5Y cells with or without CVB5 infection. Furthermore, comprehensive bioinformatics analyses were conducted to explore and probe the functions of the differentially expressed circRNAs. Our results revealed the potential function on circRNA in host-virus interaction, aiming at the potential roles of circRNA in diseases caused by CVB5.

## 2. Materials and Methods

### 2.1. Cell Culture and CVB5 Infection

SH-SY5Y, a neuron-specific human cell line, was grown in Dulbecco's modified Eagle's medium (DMEM, HyClone, USA) supplemented with 10% fetal bovine serum (FBS) (AusGeneX, AUS) and maintained in a humidified atmosphere with 5% CO_2_ at 37°C. CVB5 strain was isolated in Kunming, Yunnan province (China), in 2014 and preserved at −80°C. At 70–80% confluency, SH-SY5Y cells were mock-infected or infected with CVB5 at a 1 multiplicity of infection (MOI). The cytopathic effect in SH-SY5Y cells was evaluated at 24 h post-infection. Three biological replicates were performed in each treatment group.

### 2.2. RNA Extraction, Library Construction, and Sequencing

Total RNAs were isolated from infected as well as uninfected cells using RNAiso Plus (Takara, Japan) according to the manufacturer's instructions. The purity, concentration, and integrity of extracted total RNAs were evaluated using NanoPhotomete, Qubit® 2.0, and Agilent 2100. RNA sequencing (RNA-seq) libraries were constructed using rRNA-depleted RNA. Raw sequencing data were obtained using the Illumina HiSeq platform (150 bp paired-end reads). Clean data (clean reads) were subsequently retrieved by eliminating adaptor-polluted reads and low-quality reads. RNA library was sequenced by Novogene Co., Ltd (Beijing, China).

### 2.3. Identification and Analysis of Differentially Expressed circRNAs

Human genome sequences and gene annotations were downloaded from NCBI database (https://www.ncbi.nlm.nih.gov/projects/genome/guide/human/index.shtml). Identification of circRNA was performed using find_circ and CIRI2. In order to lower the rate of false positive identification, we only utilized the data where the circRNA was identified in the same location by two different software [14]. The read numbers mapped to each gene were counted using HTSeq v0.6.0. Quantification of expressed circRNAs were performed using TPM (transcript-per-million). Differential expression analysis of two groups (three biological replicates per condition) was performed using DESeq2 R package (1.10.1). CircRNAs with *p*-values <0.05 and fold changes (FC) >2 were defined to be differentially expressed [15].

### 2.4. Enrichment Analysis of Differentially Expressed circRNA-HostGene

Gene Ontology (GO) enrichment analysis of differentially expressed circRNA-host gene and statistical enrichment in KEGG pathways were implemented by the cluster Profiler R package21 [16]. CircRNAs with *p*-values <0.05 were considered to be significantly enriched.

### 2.5. Real-Time Quantitative PCR (RT-qPCR)

Total RNAs were extracted from mock-infected and CVB5-infected with SHSY-5Y cells. Thereafter, RT-qPCR was performed using One Step SYBR PrimeScript PLUS RT-PCR Kit (Takara, Japan) on a 7500 real-time PCR system programmed with the following cycling conditions: 42°C, 5 min, 95°C, 10 s, and 40 cycles of 95°C, 5°s and 60°C, 34 s. Expression fold change relative to controls was calculated using the 2^−*ΔΔ*Ct^ method [17]. The expression levels of circRNAs were normalized to those of ACTB. Primer sequences are listed in Table 1. Six circRNAs with its fold change >5 and *p*-value <0.05 were selected for validation. Additionally, the expression levels of miRNAs were normalized to those of U6, and the primer sequences are shown in Table 2.

### 2.6. Construction of the Interaction Network

Putative miRNA binding sites in six identified circRNAs were identified by employing the miRanda algorithm [18]. The CircRNA–miRNA–mRNA interaction network was constructed using Cytoscape 3.4.0 [19].

### 2.7. Analysis of the Candidate circRNAs Functions

The pcDNA3.1-circRNA was constructed to verify the impact on CVB5 replication. Also, the expression levels of all miRNAs and the key factors according to the circRNA–miRNA–mRNA interaction network were analyzed.

## 3. Results

### 3.1. General Profiles of circRNA in SH-SY5Y Cells with CVB5 Infection

**Figure 1:**
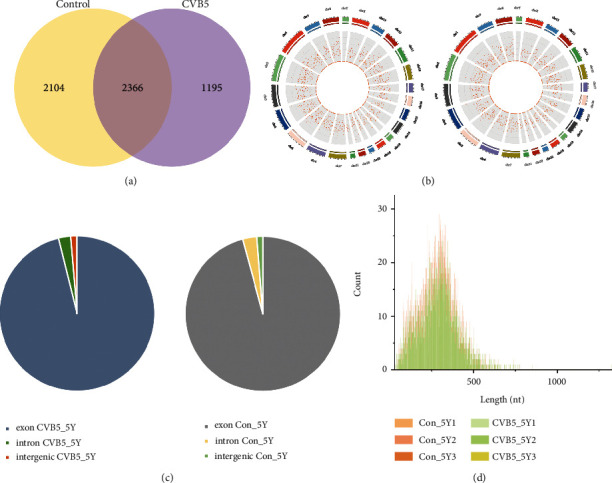
Genomic features of circRNAs detected in CVB5-infected SH-SY5Y cells. (a) Venn diagram of the number of differentially expressed circRNAs in each group. A total of 2,366 circRNAs were expressed in two groups. (b) Genomic distribution of circRNAs. (c) Chromosomal distribution of circRNAs. (d) Length distribution statistics of circRNAs.

Total RNAs without contaminating rRNAs and linear RNAs were extracted from CVB5-infected or mock-infected cell samples and subsequently used as the template for RNA-seq. Following the removal of adapter sequences and low quality sequences, all clean reads generated from the samples were mapped to the reference genome. A total of 5,665 circRNAs were identified in all samples using find_circ and CIRI2 (Supplementary Table 1). Among them, 2,366 circRNAs were observed to be commonly expressed in both treatment groups. Meanwhile, 1,195 and 2,104 circRNAs were identified to be exclusively expressed in CVB5-infected cells and mock-infected cells, respectively (Supplementary Table 1 and Figure 1(a)). These results indicated that circRNAs were differentially expressed in SH-SY5Y cells infected with CVB5 compared to those in uninfected cells.

The length, exon number, chromosome distribution, distribution in the gene position, and the GC content of identified circRNAs were analyzed. Our results showed that the circRNAs were uniformly distributed across different chromosomes, with chromosome *Y* containing the smallest number of circRNAs (Figure 1(b)). In addition, the genomic position distribution results showed that the circRNAs were mainly derived from exons and intron and partly from intergenic regions (Figure 1(c)). Because most of exon-derived circRNAs were generated from two exons by back splicing, exons may be the preferred circRNA expression (Supplementary Table 2). Distribution analysis of circRNA sequence lengths showed that most circRNAs sequences ranged from 26 nt to 1323 nt and that majority of circRNAs were from 100 nt to 500 nt in length (Figure 1(d)). Sequence analysis showed that the circRNAs sequences have an average *G* + *C* content of 49.74% (Supplementary Table 3).

### 3.2. Analysis of Differentially Expressed circRNA

**Figure 2:**
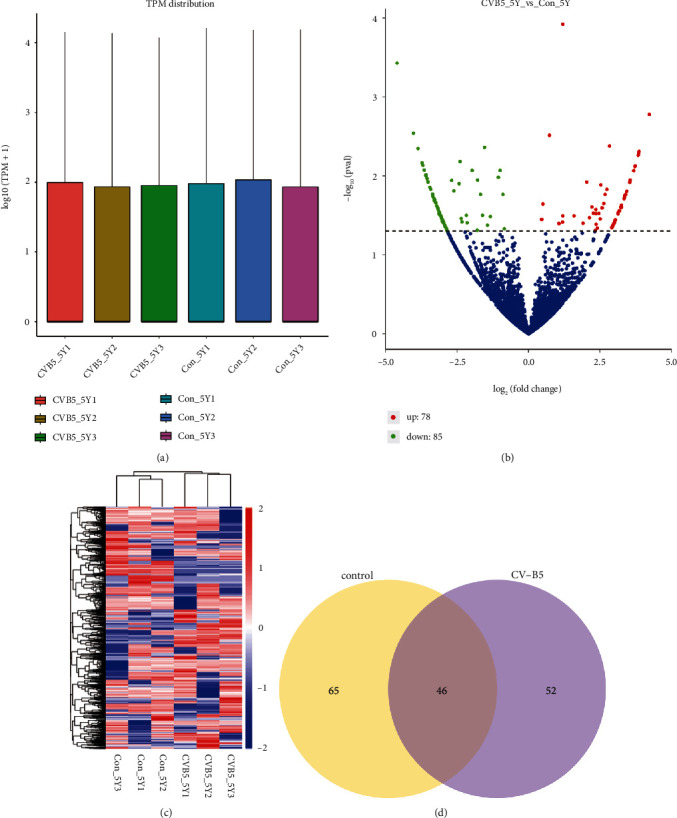
Differentially expressed circRNAs following CVB5 infection. (a) Box plots showing the expression levels of circRNAs from each sample. (b) Volcano plots depicting differentially expressed circRNAs following CVB5 infection. (c) Heatmap of maximally expressed circRNAs across all samples. (d) Venn diagrams of differentially expressed circRNAs identified in mock-infected and CVB5-infected SH-SY5Y cells.

In order to screen for dysregulated circRNAs, the circRNAs expression profiles in CVB5 infection and mock-infected cells were analyzed using DEseq 2. The abundance of each expressed circRNA was calculated based on TPM. The distribution of gene expression in all six samples was observed to be consistent (Figure 2(a)). More than 50% of circRNAs had a TPM value of less than 0.1 (Supplementary Table 4). Analysis of differentially expressed circRNAs by DESeq 2 showed that out of 163 differentially expressed circRNAs upon CVB5 infection, 78 circRNAs were upregulated and 85 circRNAs were downregulated (Figure 2(b)). Intersample correlation analysis demonstrated that there was an obvious distinction in circRNA expression patterns between mock-infected cells and CVB5-infected cells (Figure 2(c)). Of these circRNAs, 52 were newly emerging circRNAs after CVB5 infection and 65 disappeared after CVB5 infection (Figure 2(d)).

### 3.3. Biological Function Analysis for Host circRNAs Transcripts

**Figure 3:**
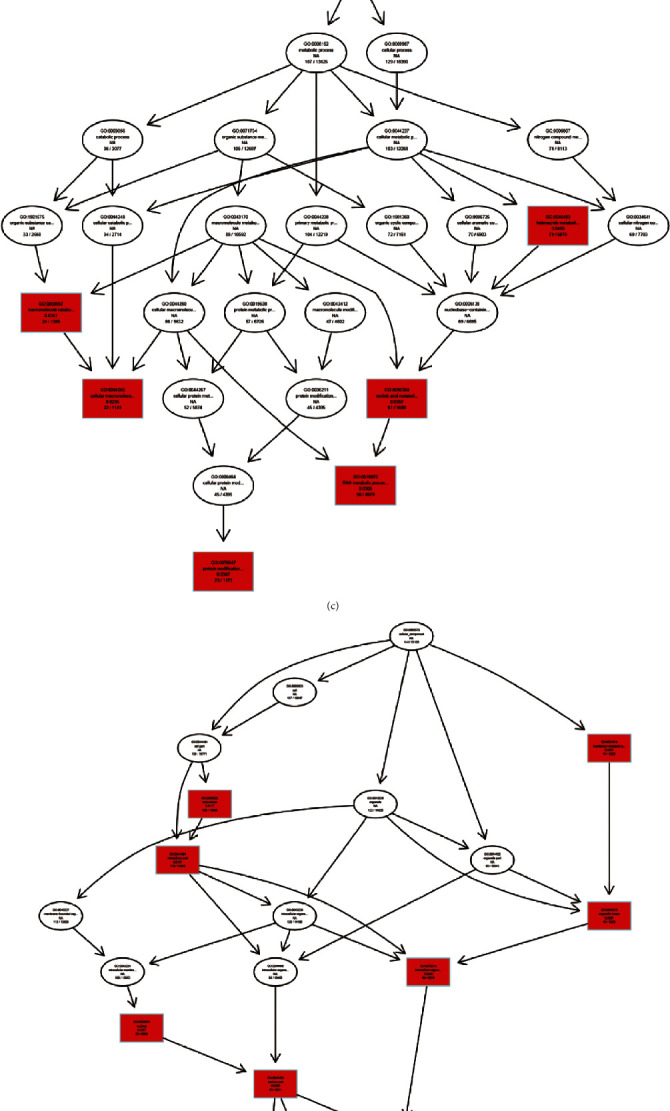
Enrichment analyses of all differentially expressed circRNAs. (a) Biological process (BP), cell component (CC), and molecular factor (MF) of GO terms. (b)–(d) Directed acyclic graph (DAG) of BP, CC, and MF. (e) Top 20 KEGG pathways of host genes of all circular RNAs.

In order to reveal the biological functions of differentially expressed circRNAs, GO assignments were exploited to categorize the functions of the host genes of differentially expressed circRNAs. GO functional classification was based on three different categories, namely, cellular components, molecular function, and biological processes. The top 20 significant enrichment GO terms for CVB5-infected cells are shown in Figure 3(a). Our results showed that most circRNA-host genes of the cellular component category were “intracellular”. Meanwhile, circRNA-host genes of the molecular function category were mainly of “organic cyclic compound binding” and “heterocyclic compound binding”. Lastly, circRNA-host genes of the biological process category were mainly involved in the “heterocycle metabolic process”. The directed acyclic graph (DAG) was constructed to display the GO structure results. Our analysis showed that enriched GO terms in the DAG of BP, CC, and MF were 6, 10, and 5, respectively (Figure 3(b)–3(d)). Additionally, KEGG pathway analysis indicated that differentially expressed circRNAs may also affect important pathways, five pathways were significantly enriched, such as ubiquitin-mediated proteolysis, tight junction, TNF signaling pathway, mRNA surveillance pathway, and MAPK signaling pathway (Figure 3(e)).

### 3.4. Validation of Selected circRNAs

**Figure 4:**
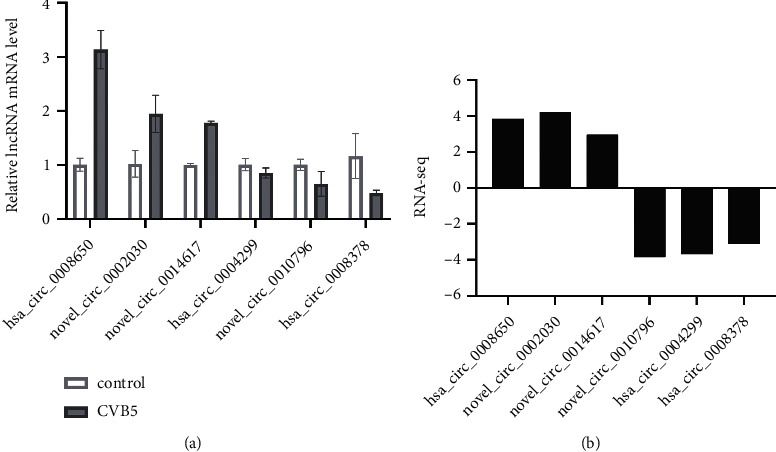
Validation of differentially expressed circRNAs by qRT-PCR. (a) Expression levels of six circRNAs validated by qRT-PCR. (b) Expression levels of six circRNA validated by RNA-seq.

To validate the reliability of the RNA-seq results, we performed RT-qPCR to detect the expression of differentially expressed circRNAs in SH-SY5Y cells infected by CVB5. Six typically differentially expressed circRNAs were selected for validation experiments. As shown in Figure 4(a), these results indicated that the hsa_circ_0008650, hsa_circ_0002030, and hsa_circ_0014617 were upregulated; hsa_circ_0004299, hsa_circ_0010796, and hsa_circ_0008378 were downregulated, which were consistent with our data analyzed by RNA-seq (Figure 4(b)).

### 3.5. Construction of the circRNA–miRNA–mRNA Interaction Network

**Figure 5:**
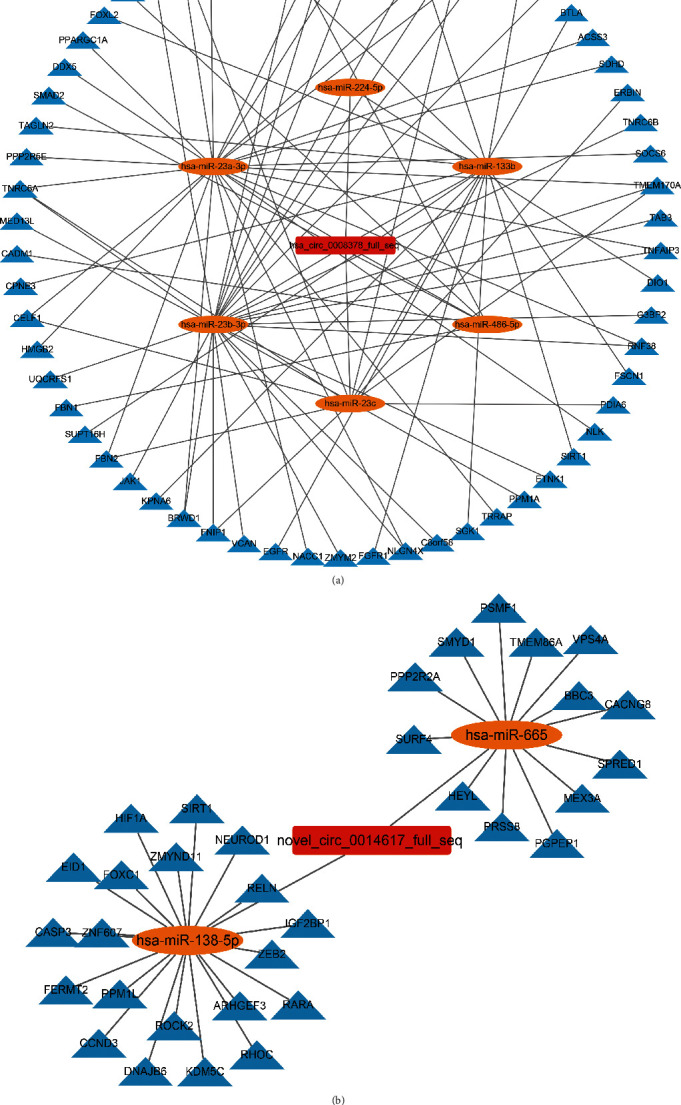
Construction of the circRNA–miRNA–mRNA interaction network. (a) Network of circRNA hsa_circ_0008378. (b) Network of circRNA novel_circ_0014617.

CircRNAs are known to offset miRNA-mediated gene regulation by acting as miRNA sponges [8]. To identify the critical roles of expressed circRNAs in CVB5-infected SH-SY5Y cells, a circRNA–miRNA–mRNA interaction network was established. According to the KEGG pathway, we selected differentially expressed circRNAs that regulated the immune system process. As shown in Figure 5, a total of two circRNAs, namely, hsa_circ_0008378 and novel_circ_0014617 were associated with 114 miRNAs; however, only 8 miRNAs have potential interactions with 112 mRNAs, while the rest (92.92% of associated miRNAs) have no mRNA binding sites (Figure 5). Both circRNAs have putative binding sites for hsa-miR-29a-5p and hsa-miR-4535 (Supplementary Table 5). Hsa_circ_0008378 was associated with the JAK-STAT signaling pathway-related network involving 6 miRNAs and 62 target genes (Figure 5(a)). Meanwhile, novel_circ_0014617 was involved in the RIG-I signaling pathway-related network and is associated with 2 miRNAs and 33 target genes (Figure 5(b)). Above all, the circRNA–miRNA–mRNA axes may form a complex interaction network and play a role in CVB5 infection.

### 3.6. Verification of circRNA hsa_circ_0008378 and novel_circ_0014617

**Figure 6:**
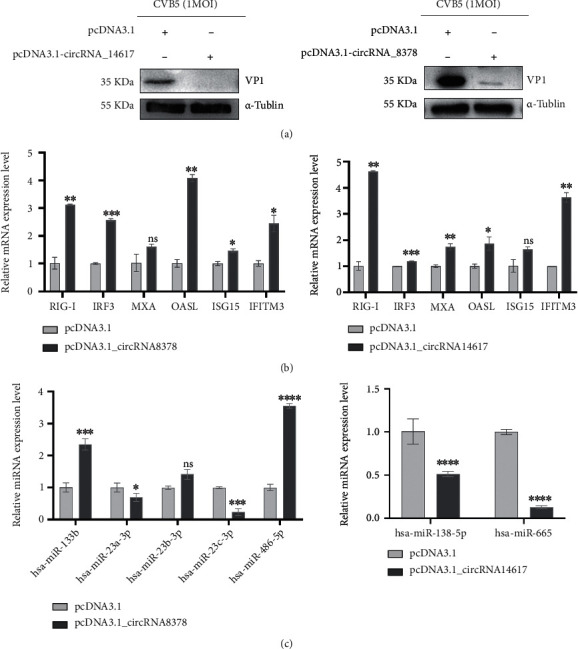
Verification of circRNA hsa_circ_0008378 and novel_circ_0014617. (a) Western blot analysis of CVB5 specific protein VP1 expression transfected with pcDNA3.1-circRNAs. (b) RT-qPCR detected the expression of genes in IFN-I pathways (RIG-I, IRF3, MxA, OSAL, ISG15, and IFITM3). (c) RT-qPCR detected the expression of target miRNA according to the network. Data are representative of three independent experiments with similar results. Data are shown as mean ± SD (b) and (c). Ns, not significant, ^∗^*p* < 0.05, ^∗∗^*p* < 0.01, ^∗∗∗^*p* < 0.001, and ^∗∗∗∗^*p* < 0.0001, two-tailed unpaired Student's *t* test.

We selected the circRNA hsa_circ_0008378 and novel_circ_0014617 from the network. Both the two circRNAs inhibited the CVB5 replication by promoting the key factors in the IFN-I signaling pathway (Figures 6(a) and (b)). Hsa_circ_0008378 was positively correlated with six targeted miRNAs, and novel_circ_0014617 was negatively correlated with two targeted miRNAs (Figure 6(c)).

## 4. Discussion

The prevalence of CVB5 has been increasing every year, causing considerable threats to children under the age of five. It also has been reported that adults infected with CVB5 are at risk of developing severe clinical complications [20]. Moreover, central nervous system (CNS) pathology and neurological sequelae often occur after CVB5 infection, which increases risk of mortality [21, 22]. In this study, the human neuroblastoma cell line, SH-SY5Y, was adopted for studying neurological damages of CVB5 infection. CircRNA, a special class of endogenous noncoding RNA that plays an important role in the process of immune regulation has been shown to be involved in the development of a number of diseases [23]. Therefore, studies of circRNA in virus-infected cells may provide an important theoretical basis for viral disease treatment. In this study, RNA-seq was applied to investigate the circRNA expression profile of SH-SY5Y cells infected with CVB5. The mechanisms of circRNAs identified to be involved in innate immune signaling pathway were further analyzed.

A total of 5,665 circRNAs identified to be present in SH-SY5Y cells were mainly derived from exons that were located on every chromosome except the *Y* chromosome. Only 3,167 circRNAs were found to be matched in the CircBase database; hence, the rest were classified as newly discovered circRNAs. Moreover, 163 differentially expressed circRNAs were identified in CVB5-infected cells. There were 26 differentially expressed circRNAs identified in both mock-infected cells and CVB5-infected cells. We speculated that these 26 differentially expressed circRNAs may be cell-specific and unrelated to virus infection. Enrichment analysis showed that enriched GO-BP terms mainly focused on the “metabolic process” and “protein modification,” which was largely consistent with EV71 or CV-A16 virus infection. However, enrichment analysis of SH-SY5Y cells infected with EV71 or CA-16 indicated association with the “gonadotropin-releasing hormone receptor pathway,” “Wnt signaling pathway,” “angiogenesis,” and “p53 pathway”, which were not present in our analysis with CVB5 infection [24, 25]. In our study, host genes that correspond to the differentially expressed circRNAs were mainly involved in “ubiquitin mediated proteolysis”, “tight junction,” and the regulation of other signaling pathways, such as the MAPK pathway. Ubiquitination, the most prevalent protein post-translational modification in cells, was known to play an important role in the dynamic regulation of host defense against pathogenic microbial infection [26, 27]. Ubiquitin often conjugates to lysine residues in substrate proteins, thereby inhibiting the interaction of transcription factors with upper signal molecular. For example, STAT1 has linear ubiquitination which inhibits the binding of type-I interferon receptor IFNAR2, thereby restricting STAT1 phosphorylation and resulting in type-I interferon signaling, in order to improve interferon (IFN) antiviral efficacy [28]. In our study, the key junction molecules may undergo ubiquitination modification in order to regulate the antiviral immune response, although further validation was required. Tight junction may influence virus infection by blocking virus entry into the cells [29]. The MAPK signaling pathway has been shown to be involved in inflammatory response during IAV infection [30]. Hence, our results collectively indicated that the altered expression of circRNAs may be the key regulators that participate in the infectious process of CVB5.

An increasing number of studies have shown that the circRNA function as works as miRNA sponges. CIRS-7, the first circRNA discovered, has been shown to play a role as a tumor promoter by decreasing the regulation of miR-7 on central oncogenic factors via competitive interactions with miR-7 [31]. Subsequently, circRNA-miRNA interactions in viral infection were put forward. During the infection of Kaposis sarcoma-associated herpesvirus, hsa_CIRC_0001400 has been shown to inhibit the expression of latency-associated nuclear antigen or replication and transcription activators in order to decrease virus infectivity through miR-K12-7-5p targeting [32]. The same results have also been found in human cytomegalovirus and influenza A virus infection [6]. Thus, it is necessary to establish the differentially expressed circRNAs interaction network. In order to better understand the pathogenesis of CVB5 infection that may facilitate the development of new therapeutic target, we focused on the circRNAs interaction network in the IFN-I pathway. The host innate immune system was the first line defense against viral invasion or replication. The association between the IFN-I signaling pathway and the viral infection has been widely documented, such as HBV, HSV, and IAV [33, 34].

We found that hsa_circ_0008378 mainly affected the JAK-STAT/IFN signaling pathway and the related ceRNA network. A total of 62 mRNA were identified to compete for 6 miRNAs including “hsa-miR-23a-3p/hsa_circ_0008378/TNFAIP3,” “hsa-miR-23a-3p/hsa_circ_0008378/DDX5,” “hsa-miR-23c/hsa_circ_0008378/BTLA,” and “hsa-miR-23b-3p/hsa_circ_0008378/JAK1” from the IFN-I signaling pathway. TNFAIP3 (also known as A20), which was essential for maintaining immune homeostasis, is a key regulator of inflammatory, antiviral, and apoptotic signaling pathways [35]. DEAD-box polypeptide 5 (DDX5), also called p68, is an ATP-dependent RNA helicase. The roles of DDX5 in viral infection have been established. For instance, DDX5 has been shown as a host factor that exhibits antiviral activity during HBV infection. Furthermore, Japanese encephalitis virus (JEV) and hepatitis C virus (HCV) have been found to hijack DDX5 to facilitate various steps of their replication cycles [36]. B-and T-lymphocyte attenuator (BTLA), an immune-regulatory receptor, was known to promote HSV infection in host cells by forming an interactive network of CD160/BTLA/LIGHT/HVEM [37]. Deubiquitinating enzyme HFMD virus structural protein VP3 has been shown to degrade Janus kinase 1 (JAK1) to inhibit IFN-*γ* signal transduction pathways [38]. Also, we found that novel_circ_0014617 is enriched in the RIG-I-like receptor signaling pathway and that 33 mRNAs competitively bind to 2 miRNAs. Surprisingly, hsa-miR-138-5p was found to interact with lncRNA-GAS5, which has been shown to reverse cardiomyocyte injury by lower CYP11B2 expression [39]. Additionally, we showed that “hsa-miR-138-5p/novel_circ_0014617/DNAJB6” and “hsa-miR-665/novel_circ_0014617/VPS4A” were enriched in the RIG-I signaling pathway. DNAJB6 has also shown to actively impact viral infection. Direct interaction in the DNAJB6 and prion protein during viral infection has been shown to favor nuclear localization of the HIV-1 pre integration complex. In addition, the delivery of primase-UL70 into cell nuclei through DNAJB6 may promote the synthesis of viral DNA during HCMV infection [40]. Although not all miRNA in the two circRNA networks selected in this study have binding with mRNA, they may be involved in CVB5 infection because they were the target molecules of circRNA. These miRNAs should be studied further, and the findings may provide new insights into CVB5 infection.

In summary, our study revealed the characteristics and profiles of circRNAs in CVB5-infected SH-SY5Y cells by RNA-seq. Differentially expressed circRNAs were identified and their potential functions were predicted by GO and KEGG. Enrichment analysis demonstrated that circRNAs associated with CVB5 infection participate in the cell metabolic process and immune response. Moreover, a circRNA–miRNA–mRNA network was constructed, which is based on the six validated circRNAs. Our analysis indicated that the circRNA played critical roles in the regulation of CVB5 infection through the IFN-I signaling pathway. All the above results are the first to identify the circRNA expression profile and construct a circRNA-related regulatory network involved in interactions between CVB5 and SH-SY5Y cells, in particular, in the host innate immune defense to viral infection. Also, the findings may also facilitate the identification of novel molecular targets for the prevention and treatment of CVB5 infection.

## Figures and Tables

**Table 1:** Primers used for circRNA expression analysis.

circRNA	Forward primer 5′-3′	Reverse primer 5′-3′
ACTB	GCGTGACATTAAGGAGAAGC	CCACGTCACACTTCATGATGG
hsa_circ_0008650	ACAGCCTTTCCCACGACTTG	AATGGCAAAAGCAGCAAAGC
novel_circ_0002030	ACACGCAGAAGATCAGCACC	CAGTTCCCCAGTTAGCCAGC
hsa_circ_0014617	GAGTTTCCCCAGTGCCTTCG	GCCCTCGCCTATTTCTCCTTT
hsa_circ_0004299	GCACATCAAGAAGCCCATCC	CGCAGGTCACAATACGGTTAC
hsa_circ_0010796	CCAAATTTAGTGCAGAAAAGGGC	CCACACGGCTCTGGATGG
hsa_circ_0008378	TAAGAAAGCGACCCAGCCG	GCTGACTGTTGTCTGATGTCTTCC

**Table 2:** Primers used for miRNA expression analysis.

miRNA	Reverse transcription primer 5′-3′	Forward primer 5′-3′	Reverse primer 5′-3′
miR-133b	GTCGTATCGACTGCAGGGTCCGAGGTATTCGCAGTCGATACGACTAGCTG	CGGCTTTGGTCCCCTTCAAC	ACTGCAGGGTCCGAGGTATT
miR-23a-3p	GTCGTATCGACTGCAGGGTCCGAGGTATTCGCAGTCGATACGACGGAAAT	CGGCATCACATTGCCAGGG	ACTGCAGGGTCCGAGGTATT
miR-23b-3p	GTCGTATCGACTGCAGGGTCCGAGGTATTCGCAGTCGATACGACGTGGTA	CGGCATCACATTGCCAGGGAT	ACTGCAGGGTCCGAGGTATT
miR-23c-3p	GTCGTATCGACTGCAGGGTCCGAGGTATTCGCAGTCGATACGACGGGTAA	CGGCATCACATTGCCAGTGA	ACTGCAGGGTCCGAGGTATT
miR-486-5p	GTCGTATCGACTGCAGGGTCCGAGGTATTCGCAGTCGATACGACCACGGG	CGGCTCCTGTACTGAGCTGC	ACTGCAGGGTCCGAGGTATT
miR-138-5p	GTCGTATCGACTGCAGGGTCCGAGGTATTCGCAGTCGATACGACCGGCCT	CGGCAGCTGGTGTGTGAATC	ACTGCAGGGTCCGAGGTATT
miR-665	GTCGTATCGACTGCAGGGTCCGAGGTATTCGCAGTCGATACGACAGGGGC	CGGCACCAGGAGGCTGAG	ACTGCAGGGTCCGAGGTATT
U6	—	CTCGCTTCGGCAGCACA	AACGCTTCACGAATTTGCGT

## Data Availability

The genetic data presented in this paper are publicly available in the GenBank database under accession no. GEO180816.

## Supplementary Materials

Supplementary 1 Supplementary Table 1 Identification of total circRNAs.

Supplementary 2 Supplementary Table 2 Feature of the identified circRNA genome.

Supplementary 3 Supplementary Table 3 Summary of sequencing data quality.

Supplementary 4 Supplementary Table 4 Distribution of TPM values of circRNAs.

Supplementary 5 Supplementary Table 5 miRNA targets of circRNAs.
