# Supplementary material for: Expression Profiles of Differentially Expressed Circular RNAs and circRNA–miRNA–mRNA Regulatory Networks in SH-SY5Y Cells Infected with Coxsackievirus B5
Source: Int J Genomics. 2022 Oct 10;2022:9298149. doi: 10.1155/2022/9298149 (PMC9577011; doi:10.1155/2022/9298149)
Supplement: Supplementary 1 — Supplementary Table 1 Identification of total circRNAs. [file 9298149.f1.pdf]

Table S1. Identifie of total circRNAs

circRNA_ID	strand	gene_id	samples
hsa_circ_0004663	+	ENSG00000047644;	Con_5Y1
hsa_circ_0006727	+	ENSG00000077713;	Con_5Y1
hsa_circ_0091506	+	ENSG00000156697;	Con_5Y1
hsa_circ_0007717	+	ENSG00000176896;	Con_5Y1
hsa_circ_0090739	-	ENSG00000086758;	Con_5Y1
hsa_circ_0002227	-	ENSG00000085224;	Con_5Y1
hsa_circ_0030130	+	ENSG00000102780;	Con_5Y1
hsa_circ_0030567	-	ENSG00000125257;	Con_5Y1
hsa_circ_0030722	+	ENSG00000134882;	Con_5Y1
hsa_circ_0027861	+	ENSG00000136021;	Con_5Y1
hsa_circ_0028021	+	ENSG00000111785;	Con_5Y1
hsa_circ_0008081	-	ENSG00000110880;	Con_5Y1
hsa_circ_0028146	+	ENSG00000139437;	Con_5Y1
hsa_circ_0028319	-	ENSG00000198270;	Con_5Y1
hsa_circ_0029051	-	ENSG00000284934;ENSG0	Con_5Y1
hsa_circ_0025506	+	ENSG00000013588;	Con_5Y1
hsa_circ_0029553	-	ENSG00000176915;	Con_5Y1
hsa_circ_0000385	+	ENSG00000123106;	Con_5Y1
hsa_circ_0025785	-	ENSG00000110888;	Con_5Y1
hsa_circ_0026616	-	ENSG00000094914;	Con_5Y1
hsa_circ_0002419	+	ENSG00000067798;	Con_5Y1
hsa_circ_0027707	-	ENSG00000070961;	Con_5Y1
hsa_circ_0005127	+	ENSG00000076043;	Con_5Y1
hsa_circ_0024473	+	ENSG00000095139;	Con_5Y1
hsa_circ_0008960	+	ENSG00000172273;	Con_5Y1
hsa_circ_0021500	+	ENSG00000185238;	Con_5Y1
hsa_circ_0021728	+	ENSG00000026508;	Con_5Y1
hsa_circ_0020915	-	ENSG00000110713;	Con_5Y1
hsa_circ_0002464	-	ENSG00000110713;	Con_5Y1
hsa_circ_0004899	+	ENSG00000198561;	Con_5Y1
hsa_circ_0002440	-	ENSG00000175376;	Con_5Y1
hsa_circ_0023031	+	ENSG00000173715;	Con_5Y1
hsa_circ_0001968	+	ENSG00000110075;	Con_5Y1
hsa_circ_0023555	-	ENSG00000168014;	Con_5Y1
hsa_circ_0023940	-	ENSG00000073921;	Con_5Y1
hsa_circ_0024016	+	ENSG00000166004;	Con_5Y1
hsa_circ_0019777	+	ENSG00000148842;	Con_5Y1
hsa_circ_0017469	+	ENSG00000047056;	Con_5Y1
hsa_circ_0020078	+	ENSG00000151553;	Con_5Y1
hsa_circ_0017709	-	ENSG00000151461;	Con_5Y1
hsa_circ_0020336	+	ENSG00000019995;	Con_5Y1
hsa_circ_0008885	-	ENSG00000175029;	Con_5Y1
hsa_circ_0006279	+	ENSG00000120539;	Con_5Y1
hsa_circ_0007403	-	ENSG00000134470;	Con_5Y1
hsa_circ_0003658	+	ENSG00000138311;	Con_5Y1
hsa_circ_0018544	+	ENSG00000060339;	Con_5Y1
hsa_circ_0018557	+	ENSG00000060339;	Con_5Y1
hsa_circ_0018659	+	ENSG00000107719;	Con_5Y1
hsa_circ_0007797	+	ENSG00000035403;	Con_5Y1
hsa_circ_0019116	+	ENSG00000107854;	Con_5Y1
hsa_circ_0008332	+	ENSG00000107854;	Con_5Y1
hsa_circ_0019120	+	ENSG00000095564;	Con_5Y1
hsa_circ_0004896	-	ENSG00000171311;	Con_5Y1
hsa_circ_0019421	+	ENSG00000198018;	Con_5Y1
hsa_circ_0007871	-	ENSG00000167193;	Con_5Y1
hsa_circ_0042268	-	ENSG00000171953;	Con_5Y1

hsa_circ_0042418	-	ENSG00000108599;	Con_5Y1
hsa_circ_0004719	-	ENSG00000108599;	Con_5Y1
hsa_circ_0041147	-	ENSG00000181031;	Con_5Y1
hsa_circ_0003481	-	ENSG00000109118;	Con_5Y1
hsa_circ_0004329	-	ENSG00000108578;	Con_5Y1
hsa_circ_0008283	+	ENSG00000276234;	Con_5Y1
hsa_circ_0043379	-	ENSG00000276293;	Con_5Y1
hsa_circ_0043975	+	ENSG00000188554;	Con_5Y1
hsa_circ_0000785	-	ENSG00000008294;	Con_5Y1
hsa_circ_0044699	+	ENSG00000166263;	Con_5Y1
hsa_circ_0044792	+	ENSG00000108384;	Con_5Y1
hsa_circ_0044812	-	ENSG00000108395;	Con_5Y1
hsa_circ_0044875	+	ENSG00000062716;	Con_5Y1
hsa_circ_0000798	+	ENSG00000171634;	Con_5Y1
hsa_circ_0045834	+	ENSG00000092931;	Con_5Y1
hsa_circ_0005294	+	ENSG00000170037;	Con_5Y1
hsa_circ_0046178	-	ENSG00000182446;	Con_5Y1
hsa_circ_0005221	-	ENSG00000182446;	Con_5Y1
hsa_circ_0046200	-	ENSG00000182446;	Con_5Y1
hsa_circ_0046496	+	ENSG00000141556;	Con_5Y1
hsa_circ_0002078	+	ENSG00000103222;	Con_5Y1
hsa_circ_0037353	-	ENSG00000063854;	Con_5Y1
hsa_circ_0006315	+	ENSG00000205629;	Con_5Y1
hsa_circ_0037558	+	ENSG00000167978;	Con_5Y1
hsa_circ_0007175	+	ENSG00000213918;	Con_5Y1
hsa_circ_0039245	-	ENSG00000129636;	Con_5Y1
hsa_circ_0039353	+	ENSG00000177200;	Con_5Y1
hsa_circ_0004656	+	ENSG00000103064;	Con_5Y1
hsa_circ_0040132	+	ENSG00000090857;	Con_5Y1
hsa_circ_0040937	+	ENSG00000197912;	Con_5Y1
hsa_circ_0007550	+	ENSG00000183475;	Con_5Y1
hsa_circ_0008425	-	ENSG00000185418;	Con_5Y1
hsa_circ_0035058	+	ENSG00000137770;	Con_5Y1
hsa_circ_0035060	+	ENSG00000137770;	Con_5Y1
hsa_circ_0035086	-	ENSG00000104133;	Con_5Y1
hsa_circ_0035209	-	ENSG00000104064;	Con_5Y1
hsa_circ_0004432	-	ENSG00000157483;	Con_5Y1
hsa_circ_0000615	+	ENSG00000180357;	Con_5Y1
hsa_circ_0036103	-	ENSG00000137831;	Con_5Y1
hsa_circ_0036295	-	ENSG00000179151;	Con_5Y1
hsa_circ_0036587	-	ENSG00000064726;	Con_5Y1
hsa_circ_0036998	+	ENSG00000173575;	Con_5Y1
hsa_circ_0006592	+	ENSG00000022976;	Con_5Y1
hsa_circ_0008754	-	ENSG00000185024;	Con_5Y1
hsa_circ_0031113	-	ENSG00000129566;	Con_5Y1
hsa_circ_0031427	+	ENSG00000092108;	Con_5Y1
hsa_circ_0031871	-	ENSG00000151748;	Con_5Y1
hsa_circ_0032115	+	ENSG00000020426;	Con_5Y1
hsa_circ_0049260	+	ENSG00000065989;	Con_5Y1
hsa_circ_0049265	+	ENSG00000065989;	Con_5Y1
hsa_circ_0004853	+	ENSG00000123146;	Con_5Y1
hsa_circ_0049874	+	ENSG00000196684;	Con_5Y1
hsa_circ_0008942	+	ENSG00000099331;	Con_5Y1
hsa_circ_0050534	+	ENSG00000126261;	Con_5Y1
hsa_circ_0051526	-	ENSG00000125746;	Con_5Y1
hsa_circ_0052233	+	ENSG00000133247;	Con_5Y1
hsa_circ_0048800	-	ENSG00000031823;	Con_5Y1
hsa_circ_0047016	+	ENSG00000101639;	Con_5Y1

hsa_circ_0046657	+	ENSG00000101557;	Con_5Y1
hsa_circ_0002608	+	ENSG00000101752;	Con_5Y1
hsa_circ_0046704	-	ENSG00000101574;	Con_5Y1
hsa_circ_0047690	+	ENSG00000082212;	Con_5Y1
hsa_circ_0000850	+	ENSG00000082212;	Con_5Y1
hsa_circ_0047719	+	ENSG00000101751;	Con_5Y1
hsa_circ_0047719	+	ENSG00000101751;	Con_5Y1
hsa_circ_0047723	+	ENSG00000101751;	Con_5Y1
hsa_circ_0005140	-	ENSG00000101493;	Con_5Y1
hsa_circ_0048011	+	ENSG00000166377;	Con_5Y1
hsa_circ_0048021	+	ENSG00000166377;	Con_5Y1
hsa_circ_0046841	+	ENSG00000101745;	Con_5Y1
hsa_circ_0046883	-	ENSG00000154845;	Con_5Y1
hsa_circ_0062220	-	ENSG00000070371;	Con_5Y1
hsa_circ_0062279	+	ENSG00000093010;	Con_5Y1
hsa_circ_0062547	-	ENSG00000228315;ENSG0	Con_5Y1
hsa_circ_0062926	-	ENSG00000241878;	Con_5Y1
hsa_circ_0063681	-	ENSG00000100271;	Con_5Y1
hsa_circ_0063776	+	ENSG00000197182;	Con_5Y1
hsa_circ_0060237	-	ENSG00000080839;	Con_5Y1
hsa_circ_0002182	+	ENSG00000124198;	Con_5Y1
hsa_circ_0006359	+	ENSG00000197818;	Con_5Y1
hsa_circ_0059402	+	ENSG00000286235;ENSG0	Con_5Y1
hsa_circ_0061694	+	ENSG00000157540;	Con_5Y1
hsa_circ_0061740	-	ENSG00000185658;	Con_5Y1
hsa_circ_0081749	-	ENSG00000105821;	Con_5Y1
hsa_circ_0003331	-	ENSG00000091127;	Con_5Y1
hsa_circ_0081892	-	ENSG00000164597;	Con_5Y1
hsa_circ_0082335	+	ENSG00000128607;	Con_5Y1
hsa_circ_0002014	-	ENSG00000146842;	Con_5Y1
hsa_circ_0003746	+	ENSG00000122786;	Con_5Y1
hsa_circ_0079126	-	ENSG00000164880;	Con_5Y1
hsa_circ_0083055	-	ENSG00000055609;	Con_5Y1
hsa_circ_0002097	-	ENSG00000105928;	Con_5Y1
hsa_circ_0006365	+	ENSG00000169902;	Con_5Y1
hsa_circ_0080496	-	ENSG00000106635;	Con_5Y1
hsa_circ_0077483	-	ENSG00000112249;	Con_5Y1
hsa_circ_0007138	-	ENSG00000152894;	Con_5Y1
hsa_circ_0077824	-	ENSG00000146376;	Con_5Y1
hsa_circ_0077824	-	ENSG00000146376;	Con_5Y1
hsa_circ_0006407	-	ENSG00000164483;	Con_5Y1
hsa_circ_0077957	-	ENSG00000135525;	Con_5Y1
hsa_circ_0078226	-	ENSG00000131023;	Con_5Y1
hsa_circ_0078765	+	ENSG00000130023;	Con_5Y1
hsa_circ_0075740	-	ENSG00000124789;	Con_5Y1
hsa_circ_0075748	-	ENSG00000137177;	Con_5Y1
hsa_circ_0075878	+	ENSG00000079691;	Con_5Y1
hsa_circ_0075496	+	ENSG00000137275;	Con_5Y1
hsa_circ_0077082	+	ENSG00000112701;	Con_5Y1
hsa_circ_0077183	-	ENSG00000005700;	Con_5Y1
hsa_circ_0007806	-	ENSG00000072364;	Con_5Y1
hsa_circ_0074102	+	ENSG00000120733;	Con_5Y1
hsa_circ_0074863	+	ENSG00000113645;	Con_5Y1
hsa_circ_0007958	+	ENSG00000113643;	Con_5Y1
hsa_circ_0074881	+	ENSG00000113643;	Con_5Y1
hsa_circ_0075071	+	ENSG00000164466;	Con_5Y1
hsa_circ_0008631	-	ENSG00000087206;	Con_5Y1
hsa_circ_0004138	-	ENSG00000246596;	Con_5Y1

hsa_circ_0072275	-	ENSG00000113569;	Con_5Y1
hsa_circ_0001483	-	ENSG00000067248;	Con_5Y1
hsa_circ_0071660	+	ENSG00000112877;	Con_5Y1
hsa_circ_0001498	-	ENSG00000164253;	Con_5Y1
hsa_circ_0073163	+	ENSG00000039319;	Con_5Y1
hsa_circ_0005187	+	n/a	Con_5Y1
hsa_circ_0003875	+	ENSG00000138802;	Con_5Y1
hsa_circ_0070648	+	ENSG00000138802;	Con_5Y1
hsa_circ_0068839	-	ENSG00000159692;	Con_5Y1
hsa_circ_0071206	-	ENSG00000198589;	Con_5Y1
hsa_circ_0069031	-	ENSG00000132406;	Con_5Y1
hsa_circ_0070482	+	ENSG00000138698;	Con_5Y1
hsa_circ_0003640	+	ENSG00000164024;	Con_5Y1
hsa_circ_0064306	+	ENSG00000197548;	Con_5Y1
hsa_circ_0066826	+	ENSG00000114573;	Con_5Y1
hsa_circ_0002243	-	ENSG00000163785;	Con_5Y1
hsa_circ_0001341	+	ENSG00000114054;	Con_5Y1
hsa_circ_0067492	+	ENSG00000174564;	Con_5Y1
hsa_circ_0067621	-	ENSG00000114127;	Con_5Y1
hsa_circ_0067689	+	ENSG00000163754;	Con_5Y1
hsa_circ_0008929	+	ENSG00000163655;	Con_5Y1
hsa_circ_0006614	+	ENSG00000163872;	Con_5Y1
hsa_circ_0001374	+	ENSG00000152492;	Con_5Y1
hsa_circ_0004555	+	n/a	Con_5Y1
hsa_circ_0064576	+	ENSG00000174738;	Con_5Y1
hsa_circ_0066078	-	ENSG00000163939;	Con_5Y1
hsa_circ_0066414	+	ENSG00000144724;	Con_5Y1
hsa_circ_0007176	-	ENSG00000144231;	Con_5Y1
hsa_circ_0003525	-	ENSG00000136715;	Con_5Y1
hsa_circ_0056433	+	ENSG00000136731;	Con_5Y1
hsa_circ_0056734	+	ENSG00000157827;	Con_5Y1
hsa_circ_0001081	+	ENSG00000115827;	Con_5Y1
hsa_circ_0057128	-	ENSG00000172845;	Con_5Y1
hsa_circ_0057301	+	ENSG00000163002;	Con_5Y1
hsa_circ_0002541	-	ENSG00000162971;	Con_5Y1
hsa_circ_0003337	+	ENSG00000055044;	Con_5Y1
hsa_circ_0058213	+	ENSG00000163466;	Con_5Y1
hsa_circ_0001102	-	ENSG00000286239;ENSG0	Con_5Y1
hsa_circ_0053882	+	ENSG00000018699;	Con_5Y1
hsa_circ_0054086	-	ENSG00000008869;	Con_5Y1
hsa_circ_0054190	-	ENSG00000011566;	Con_5Y1
hsa_circ_0054214	-	ENSG00000011566;	Con_5Y1
hsa_circ_0004447	+	ENSG00000171132;	Con_5Y1
hsa_circ_0054608	-	ENSG00000085760;	Con_5Y1
hsa_circ_0054626	-	ENSG00000275052;	Con_5Y1
hsa_circ_0054895	-	ENSG00000082898;	Con_5Y1
hsa_circ_0005504	-	ENSG00000198380;	Con_5Y1
hsa_circ_0055412	-	ENSG00000042493;	Con_5Y1
hsa_circ_0001052	-	ENSG00000135945;	Con_5Y1
hsa_circ_0002623	+	ENSG00000173218;	Con_5Y1
hsa_circ_0009995	+	ENSG00000048707;	Con_5Y1
hsa_circ_0014151	-	ENSG00000143393;	Con_5Y1
hsa_circ_0014644	-	ENSG00000132680;	Con_5Y1
hsa_circ_0000020	+	ENSG00000197312;	Con_5Y1
hsa_circ_0010109	+	ENSG00000065526;	Con_5Y1
hsa_circ_0015444	+	ENSG00000057252;	Con_5Y1
hsa_circ_0015491	+	ENSG00000135837;	Con_5Y1
hsa_circ_0008071	-	ENSG00000075151;	Con_5Y1

hsa_circ_0016414	+	ENSG00000162769;	Con_5Y1
hsa_circ_0016707	-	ENSG00000183814;	Con_5Y1
hsa_circ_0016873	-	ENSG00000119280;	Con_5Y1
hsa_circ_0017160	+	ENSG00000116984;	Con_5Y1
hsa_circ_0009376	+	ENSG00000157916;	Con_5Y1
hsa_circ_0017263	-	ENSG00000035687;	Con_5Y1
hsa_circ_0010972	+	ENSG00000130695;	Con_5Y1
hsa_circ_0011120	-	ENSG00000158161;	Con_5Y1
hsa_circ_0004320	+	ENSG00000197056;	Con_5Y1
hsa_circ_0006595	+	ENSG00000126091;ENSG0	Con_5Y1
hsa_circ_0012550	-	ENSG00000134744;	Con_5Y1
hsa_circ_0088404	+	ENSG00000011454;	Con_5Y1
hsa_circ_0088417	-	ENSG00000165209;	Con_5Y1
hsa_circ_0088530	-	ENSG00000173611;	Con_5Y1
hsa_circ_0088600	-	ENSG00000119487;	Con_5Y1
hsa_circ_0004403	+	ENSG00000136828;	Con_5Y1
hsa_circ_0088661	+	ENSG00000148356;	Con_5Y1
hsa_circ_0089721	+	ENSG00000181090;	Con_5Y1
hsa_circ_0007784	-	ENSG00000171889;	Con_5Y1
hsa_circ_0087024	-	ENSG00000137075;	Con_5Y1
hsa_circ_0002921	-	ENSG00000107362;	Con_5Y1
hsa_circ_0087232	+	ENSG00000099139;	Con_5Y1
hsa_circ_0004725	-	ENSG00000135018;	Con_5Y1
hsa_circ_0087396	+	ENSG00000135040;	Con_5Y1
hsa_circ_0008171	-	ENSG00000104517;	Con_5Y1
hsa_circ_0007374	-	ENSG00000155096;	Con_5Y1
hsa_circ_0002330	-	ENSG00000153317;	Con_5Y1
hsa_circ_0085719	-	ENSG00000123908;	Con_5Y1
hsa_circ_0085778	+	ENSG00000105339;	Con_5Y1
hsa_circ_0083458	+	ENSG00000078674;	Con_5Y1
hsa_circ_0083263	+	ENSG00000283239;ENSG0	Con_5Y1
hsa_circ_0083826	+	ENSG00000147421;	Con_5Y1
hsa_circ_0083995	-	ENSG00000147548;	Con_5Y1
hsa_circ_0084266	-	ENSG00000253729;	Con_5Y1
hsa_circ_0084501	-	ENSG00000023287;	Con_5Y1
hsa_circ_0084677	+	ENSG00000104218;	Con_5Y1
hsa_circ_0084868	-	ENSG00000104320;	Con_5Y1
hsa_circ_0084935	+	ENSG00000156162;	Con_5Y1
hsa_circ_0001814	+	ENSG00000164941;	Con_5Y1
hsa_circ_0085081	+	ENSG00000132549;	Con_5Y1
novel_circ_0018625	-	n/a	Con_5Y1
novel_circ_0018665	+	ENSG00000101911;	Con_5Y1
novel_circ_0018687	-	ENSG00000213468;	Con_5Y1
novel_circ_0018791	+	ENSG00000130741;	Con_5Y1
novel_circ_0002827	+	ENSG00000068650;	Con_5Y1
novel_circ_0002899	-	ENSG00000132953;	Con_5Y1
novel_circ_0002962	-	ENSG00000139514;	Con_5Y1
novel_circ_0003015	-	ENSG00000120690;	Con_5Y1
novel_circ_0003185	-	ENSG00000005810;	Con_5Y1
novel_circ_0003211	-	ENSG00000125257;	Con_5Y1
novel_circ_0001827	-	ENSG00000111670;	Con_5Y1
novel_circ_0001888	-	ENSG00000075856;	Con_5Y1
novel_circ_0001912	+	ENSG00000122970;	Con_5Y1
novel_circ_0001914	+	ENSG00000122970;	Con_5Y1
novel_circ_0001938	+	ENSG00000204852;	Con_5Y1
novel_circ_0001941	+	ENSG00000082805;	Con_5Y1
novel_circ_0002057	-	ENSG00000089154;	Con_5Y1
novel_circ_0002104	-	ENSG00000111011;	Con_5Y1

novel_circ_0002118	+	ENSG00000184445;	Con_5Y1
novel_circ_0002148	-	ENSG00000139697;	Con_5Y1
novel_circ_0002184	-	ENSG00000196498;	Con_5Y1
novel_circ_0002193	-	ENSG00000150990;	Con_5Y1
novel_circ_0002207	-	ENSG00000111450;	Con_5Y1
novel_circ_0002215	+	ENSG00000183495;	Con_5Y1
novel_circ_0002242	+	ENSG00000171681;	Con_5Y1
novel_circ_0002245	+	ENSG00000023697;	Con_5Y1
novel_circ_0002260	+	ENSG00000052126;	Con_5Y1
novel_circ_0002279	+	ENSG00000139163;	Con_5Y1
novel_circ_0002355	+	ENSG00000013573;	Con_5Y1
novel_circ_0002410	-	ENSG00000129317;	Con_5Y1
novel_circ_0002433	+	ENSG00000257261;	Con_5Y1
novel_circ_0002461	+	ENSG00000123352;	Con_5Y1
novel_circ_0002492	+	ENSG00000050426;	Con_5Y1
novel_circ_0002615	+	ENSG00000111581;	Con_5Y1
novel_circ_0002630	+	ENSG00000135679;	Con_5Y1
novel_circ_0002658	-	ENSG00000133858;	Con_5Y1
novel_circ_0002705	-	ENSG00000058272;	Con_5Y1
novel_circ_0000996	-	ENSG00000133812;	Con_5Y1
novel_circ_0001003	+	ENSG00000137693;	Con_5Y1
novel_circ_0001035	-	ENSG00000152404;	Con_5Y1
novel_circ_0001055	-	ENSG00000149308;	Con_5Y1
novel_circ_0001094	-	ENSG00000086848;	Con_5Y1
novel_circ_0001111	-	ENSG00000110328;	Con_5Y1
novel_circ_0001124	+	ENSG00000118058;	Con_5Y1
novel_circ_0001180	+	ENSG00000245498;	Con_5Y1
novel_circ_0001241	-	ENSG00000011405;	Con_5Y1
novel_circ_0001333	+	ENSG00000135387;	Con_5Y1
novel_circ_0001358	+	ENSG00000026508;	Con_5Y1
novel_circ_0001406	+	ENSG00000151348;	Con_5Y1
novel_circ_0001423	+	ENSG00000175224;	Con_5Y1
novel_circ_0001484	+	ENSG00000256591;	Con_5Y1
novel_circ_0001501	-	ENSG00000255717;	Con_5Y1
novel_circ_0001515	-	ENSG00000110076;	Con_5Y1
novel_circ_0001790	+	ENSG00000166037;	Con_5Y1
novel_circ_0000051	-	ENSG00000120029;	Con_5Y1
novel_circ_0000080	+	ENSG00000148843;	Con_5Y1
novel_circ_0000103	+	ENSG00000108055;	Con_5Y1
novel_circ_0000121	+	ENSG00000151532;	Con_5Y1
novel_circ_0000133	+	n/a	Con_5Y1
novel_circ_0000190	-	ENSG00000107581;	Con_5Y1
novel_circ_0000198	-	ENSG00000151461;	Con_5Y1
novel_circ_0000309	+	ENSG00000203780;	Con_5Y1
novel_circ_0000352	+	ENSG00000065328;	Con_5Y1
novel_circ_0000425	+	ENSG00000269897;ENSG0	Con_5Y1
novel_circ_0000482	+	ENSG00000095787;	Con_5Y1
novel_circ_0000483	+	ENSG00000095787;	Con_5Y1
novel_circ_0000501	-	ENSG00000165322;	Con_5Y1
novel_circ_0000518	+	ENSG00000216937;	Con_5Y1
novel_circ_0000533	-	ENSG00000148498;	Con_5Y1
novel_circ_0000567	-	ENSG00000165406;	Con_5Y1
novel_circ_0000645	-	ENSG00000057608;	Con_5Y1
novel_circ_0000701	-	ENSG00000138346;	Con_5Y1
novel_circ_0000707	-	ENSG00000138346;	Con_5Y1
novel_circ_0000714	+	ENSG00000138336;	Con_5Y1
novel_circ_0000778	-	ENSG00000122882;	Con_5Y1
novel_circ_0000833	-	ENSG00000107929;	Con_5Y1

novel_circ_0000845	-	ENSG00000182771;	Con_5Y1
novel_circ_0000856	-	ENSG00000062650;	Con_5Y1
novel_circ_0000915	+	ENSG00000138160;	Con_5Y1
novel_circ_0000950	-	ENSG00000059573;	Con_5Y1
novel_circ_0005327	+	ENSG00000132383;	Con_5Y1
novel_circ_0005393	-	ENSG00000007202;	Con_5Y1
novel_circ_0005454	+	ENSG00000196712;	Con_5Y1
novel_circ_0005523	+	ENSG00000276234;	Con_5Y1
novel_circ_0005566	-	ENSG00000074755;	Con_5Y1
novel_circ_0005598	-	ENSG00000108799;	Con_5Y1
novel_circ_0005748	+	ENSG00000011260;	Con_5Y1
novel_circ_0005925	+	ENSG00000198909;	Con_5Y1
novel_circ_0005977	-	ENSG00000154240;	Con_5Y1
novel_circ_0006053	+	ENSG00000073350;	Con_5Y1
novel_circ_0006087	+	ENSG00000078687;	Con_5Y1
novel_circ_0004630	+	ENSG00000186260;	Con_5Y1
novel_circ_0004644	-	ENSG00000166783;	Con_5Y1
novel_circ_0004681	-	ENSG00000063854;	Con_5Y1
novel_circ_0004699	+	ENSG00000103528;	Con_5Y1
novel_circ_0004888	+	ENSG00000102910;	Con_5Y1
novel_circ_0004894	+	ENSG00000102910;	Con_5Y1
novel_circ_0004903	+	ENSG00000155393;	Con_5Y1
novel_circ_0004930	+	ENSG00000087253;	Con_5Y1
novel_circ_0004941	-	ENSG00000135736;	Con_5Y1
novel_circ_0004950	-	ENSG00000125107;	Con_5Y1
novel_circ_0004966	-	ENSG00000159720;	Con_5Y1
novel_circ_0005153	-	ENSG00000103248;	Con_5Y1
novel_circ_0005154	-	ENSG00000103248;	Con_5Y1
novel_circ_0005165	-	ENSG00000103257;	Con_5Y1
novel_circ_0005189	+	ENSG00000158545;	Con_5Y1
novel_circ_0005223	+	ENSG00000197912;	Con_5Y1
novel_circ_0003954	+	ENSG00000184254;	Con_5Y1
novel_circ_0003969	-	ENSG00000273749;	Con_5Y1
novel_circ_0004072	+	ENSG00000137815;	Con_5Y1
novel_circ_0004080	-	ENSG00000166887;	Con_5Y1
novel_circ_0004132	-	ENSG00000104133;	Con_5Y1
novel_circ_0004143	-	ENSG00000166200;	Con_5Y1
novel_circ_0004153	-	ENSG00000166262;	Con_5Y1
novel_circ_0004203	-	ENSG00000285253;ENSG0	Con_5Y1
novel_circ_0004310	-	ENSG00000075131;	Con_5Y1
novel_circ_0004316	+	ENSG00000174442;	Con_5Y1
novel_circ_0004479	+	ENSG00000086666;	Con_5Y1
novel_circ_0004498	-	ENSG00000064726;	Con_5Y1
novel_circ_0004521	+	ENSG00000170776;	Con_5Y1
novel_circ_0004546	+	ENSG00000166965;	Con_5Y1
novel_circ_0004558	+	ENSG00000173575;	Con_5Y1
novel_circ_0003351	+	ENSG00000100664;	Con_5Y1
novel_circ_0003393	-	ENSG00000165819;	Con_5Y1
novel_circ_0003408	-	ENSG00000100802;	Con_5Y1
novel_circ_0003453	-	ENSG00000092148;	Con_5Y1
novel_circ_0003481	-	ENSG00000129515;	Con_5Y1
novel_circ_0003501	-	ENSG00000151332;	Con_5Y1
novel_circ_0003577	-	ENSG00000087299;	Con_5Y1
novel_circ_0003687	+	ENSG00000054654;	Con_5Y1
novel_circ_0003774	+	ENSG00000100767;	Con_5Y1
novel_circ_0003785	+	ENSG00000258653;ENSG0	Con_5Y1
novel_circ_0003792	+	ENSG00000119596;	Con_5Y1
novel_circ_0003878	-	ENSG00000100796;	Con_5Y1

novel_circ_0003888	+	ENSG00000165934;	Con_5Y1
novel_circ_0006735	-	ENSG00000267059;ENSG0	Con_5Y1
novel_circ_0006851	-	ENSG00000121289;	Con_5Y1
novel_circ_0007015	+	ENSG00000105464;	Con_5Y1
novel_circ_0007100	+	n/a	Con_5Y1
novel_circ_0007137	-	ENSG00000070423;	Con_5Y1
novel_circ_0007160	-	ENSG00000167785;	Con_5Y1
novel_circ_0006298	+	ENSG00000141449;	Con_5Y1
novel_circ_0006309	+	ENSG00000167088;	Con_5Y1
novel_circ_0006338	+	ENSG00000101773;	Con_5Y1
novel_circ_0006378	+	ENSG00000101596;	Con_5Y1
novel_circ_0006484	+	ENSG00000082212;	Con_5Y1
novel_circ_0009712	+	ENSG00000099940;	Con_5Y1
novel_circ_0009835	-	ENSG00000233080;	Con_5Y1
novel_circ_0009931	-	ENSG00000100271;	Con_5Y1
novel_circ_0010001	-	ENSG00000128159;	Con_5Y1
novel_circ_0010003	+	ENSG00000025770;	Con_5Y1
novel_circ_0009159	-	ENSG00000101391;	Con_5Y1
novel_circ_0009232	+	ENSG00000025293;	Con_5Y1
novel_circ_0009273	+	ENSG00000088888;	Con_5Y1
novel_circ_0009383	+	ENSG00000197818;	Con_5Y1
novel_circ_0009385	+	ENSG00000197818;	Con_5Y1
novel_circ_0009530	-	ENSG00000156299;	Con_5Y1
novel_circ_0009548	+	ENSG00000142166;	Con_5Y1
novel_circ_0009651	+	ENSG00000160218;	Con_5Y1
novel_circ_0009683	+	ENSG00000160299;	Con_5Y1
novel_circ_0015819	-	ENSG00000239521;	Con_5Y1
novel_circ_0015878	+	ENSG00000005483;	Con_5Y1
novel_circ_0016053	+	ENSG00000146963;ENSG0	Con_5Y1
novel_circ_0016054	+	ENSG00000146963;ENSG0	Con_5Y1
novel_circ_0016185	-	ENSG00000146918;	Con_5Y1
novel_circ_0016215	+	ENSG00000136261;	Con_5Y1
novel_circ_0016273	-	ENSG00000164548;	Con_5Y1
novel_circ_0016307	-	ENSG00000070882;	Con_5Y1
novel_circ_0016393	+	ENSG00000011426;	Con_5Y1
novel_circ_0016464	+	ENSG00000075618;	Con_5Y1
novel_circ_0016599	-	ENSG00000009954;	Con_5Y1
novel_circ_0016645	+	ENSG00000187257;	Con_5Y1
novel_circ_0014938	-	ENSG00000112249;	Con_5Y1
novel_circ_0014978	+	ENSG00000183137;	Con_5Y1
novel_circ_0014979	-	ENSG00000135596;	Con_5Y1
novel_circ_0015072	-	ENSG00000152894;	Con_5Y1
novel_circ_0015146	+	ENSG00000112149;	Con_5Y1
novel_circ_0015227	+	ENSG00000008083;	Con_5Y1
novel_circ_0015286	+	ENSG00000164674;	Con_5Y1
novel_circ_0015308	+	ENSG00000085511;	Con_5Y1
novel_circ_0015448	-	n/a	Con_5Y1
novel_circ_0015452	+	ENSG00000237649;	Con_5Y1
novel_circ_0015466	+	ENSG00000065060;	Con_5Y1
novel_circ_0015468	+	ENSG00000023892;	Con_5Y1
novel_circ_0015544	-	ENSG00000124571;	Con_5Y1
novel_circ_0015575	-	ENSG00000001084;	Con_5Y1
novel_circ_0015685	-	n/a	Con_5Y1
novel_circ_0015698	+	ENSG00000083123;	Con_5Y1
novel_circ_0013968	+	ENSG00000145495;	Con_5Y1
novel_circ_0013971	+	ENSG00000145495;	Con_5Y1
novel_circ_0014004	-	ENSG00000129595;	Con_5Y1
novel_circ_0014019	+	ENSG00000172795;	Con_5Y1

novel_circ_0014077	+	ENSG00000164902;	Con_5Y1
novel_circ_0014286	-	ENSG00000133706;	Con_5Y1
novel_circ_0014355	+	ENSG00000249738;	Con_5Y1
novel_circ_0014356	+	ENSG00000113312;	Con_5Y1
novel_circ_0014367	+	ENSG00000038274;	Con_5Y1
novel_circ_0014411	+	ENSG00000113194;	Con_5Y1
novel_circ_0014427	+	ENSG00000197451;	Con_5Y1
novel_circ_0014453	+	ENSG00000161011;	Con_5Y1
novel_circ_0014489	-	ENSG00000113360;	Con_5Y1
novel_circ_0014601	-	ENSG00000067248;	Con_5Y1
novel_circ_0014622	+	ENSG00000062194;	Con_5Y1
novel_circ_0014721	+	ENSG00000145734;	Con_5Y1
novel_circ_0014771	+	ENSG00000250802;	Con_5Y1
novel_circ_0014867	+	ENSG00000071539;	Con_5Y1
novel_circ_0013140	+	ENSG00000138792;	Con_5Y1
novel_circ_0013199	-	ENSG00000164073;	Con_5Y1
novel_circ_0013205	+	ENSG00000138709;	Con_5Y1
novel_circ_0013265	-	ENSG00000109452;	Con_5Y1
novel_circ_0013398	+	ENSG00000109466;	Con_5Y1
novel_circ_0013487	+	ENSG00000109794;	Con_5Y1
novel_circ_0013597	+	ENSG00000197386;	Con_5Y1
novel_circ_0013608	-	ENSG00000181826;	Con_5Y1
novel_circ_0013628	-	ENSG00000121892;	Con_5Y1
novel_circ_0013645	+	ENSG00000078177;	Con_5Y1
novel_circ_0013763	-	ENSG00000084092;	Con_5Y1
novel_circ_0013882	+	ENSG00000138678;	Con_5Y1
novel_circ_0013908	+	ENSG00000170502;	Con_5Y1
novel_circ_0013911	+	ENSG00000118762;	Con_5Y1
novel_circ_0011780	-	ENSG00000066422;	Con_5Y1
novel_circ_0011843	-	ENSG00000144559;	Con_5Y1
novel_circ_0011900	-	ENSG00000169087;	Con_5Y1
novel_circ_0011923	-	ENSG00000082781;	Con_5Y1
novel_circ_0012077	-	ENSG00000051382;	Con_5Y1
novel_circ_0012103	-	ENSG00000175054;	Con_5Y1
novel_circ_0012105	+	ENSG00000120756;	Con_5Y1
novel_circ_0012212	+	ENSG00000113810;	Con_5Y1
novel_circ_0012255	-	ENSG00000154310;	Con_5Y1
novel_circ_0012261	-	ENSG00000131374;	Con_5Y1
novel_circ_0012296	-	ENSG00000131374;	Con_5Y1
novel_circ_0012308	+	ENSG00000171109;	Con_5Y1
novel_circ_0012334	-	ENSG00000078070;	Con_5Y1
novel_circ_0012365	+	ENSG00000156931;	Con_5Y1
novel_circ_0012389	+	ENSG00000163904;	Con_5Y1
novel_circ_0012447	-	ENSG00000273331;ENSG0	Con_5Y1
novel_circ_0012523	-	ENSG00000077097;	Con_5Y1
novel_circ_0012535	-	ENSG00000033867;	Con_5Y1
novel_circ_0012546	-	ENSG00000235493;	Con_5Y1
novel_circ_0012602	+	ENSG00000182973;	Con_5Y1
novel_circ_0012688	+	ENSG00000114857;	Con_5Y1
novel_circ_0012741	+	ENSG00000227398;	Con_5Y1
novel_circ_0012796	-	ENSG00000047849;	Con_5Y1
novel_circ_0012852	+	ENSG00000164080;	Con_5Y1
novel_circ_0012944	-	ENSG00000189283;	Con_5Y1
novel_circ_0013006	-	ENSG00000144736;	Con_5Y1
novel_circ_0013027	+	ENSG00000134077;	Con_5Y1
novel_circ_0013036	+	ENSG00000168137;	Con_5Y1
novel_circ_0013043	+	ENSG00000114026;	Con_5Y1
novel_circ_0013044	-	ENSG00000134072;	Con_5Y1

novel_circ_0013059	-	ENSG00000057019;	Con_5Y1
novel_circ_0010026	+	ENSG00000071054;	Con_5Y1
novel_circ_0010032	+	ENSG00000135974;	Con_5Y1
novel_circ_0010056	+	ENSG00000163006;	Con_5Y1
novel_circ_0010179	+	ENSG00000136731;	Con_5Y1
novel_circ_0010248	+	ENSG00000115705;	Con_5Y1
novel_circ_0010342	-	ENSG00000196151;	Con_5Y1
novel_circ_0010345	-	ENSG00000123636;	Con_5Y1
novel_circ_0010359	-	ENSG00000115290;	Con_5Y1
novel_circ_0010468	-	ENSG00000115966;	Con_5Y1
novel_circ_0010528	+	ENSG00000138448;	Con_5Y1
novel_circ_0010541	+	ENSG00000064933;	Con_5Y1
novel_circ_0010669	+	ENSG00000119004;	Con_5Y1
novel_circ_0010736	+	n/a	Con_5Y1
novel_circ_0010738	+	ENSG00000079246;	Con_5Y1
novel_circ_0010788	+	n/a	Con_5Y1
novel_circ_0010898	+	ENSG00000204120;	Con_5Y1
novel_circ_0011157	+	ENSG00000171853;	Con_5Y1
novel_circ_0011365	-	ENSG00000068878;	Con_5Y1
novel_circ_0011500	+	ENSG00000115504;	Con_5Y1
novel_circ_0011540	-	ENSG00000243667;ENSG0	Con_5Y1
novel_circ_0011613	+	ENSG00000116127;	Con_5Y1
novel_circ_0011642	+	ENSG00000115459;	Con_5Y1
novel_circ_0011702	-	ENSG00000151694;	Con_5Y1
novel_circ_0007176	-	ENSG00000156876;	Con_5Y1
novel_circ_0007217	+	ENSG00000031698;	Con_5Y1
novel_circ_0007309	+	ENSG00000048707;	Con_5Y1
novel_circ_0007310	+	ENSG00000048707;	Con_5Y1
novel_circ_0007369	-	ENSG00000143418;	Con_5Y1
novel_circ_0007400	+	ENSG00000142621;	Con_5Y1
novel_circ_0007584	+	ENSG00000117523;	Con_5Y1
novel_circ_0007696	+	ENSG00000135837;	Con_5Y1
novel_circ_0007733	+	ENSG00000116668;	Con_5Y1
novel_circ_0007868	-	ENSG00000127483;	Con_5Y1
novel_circ_0007970	-	ENSG00000136628;	Con_5Y1
novel_circ_0007983	+	ENSG00000117791;	Con_5Y1
novel_circ_0008096	-	ENSG00000069248;	Con_5Y1
novel_circ_0008123	-	ENSG00000135766;	Con_5Y1
novel_circ_0008186	-	ENSG00000162885;	Con_5Y1
novel_circ_0008198	-	ENSG00000119285;	Con_5Y1
novel_circ_0008223	+	n/a	Con_5Y1
novel_circ_0008275	-	ENSG00000162851;	Con_5Y1
novel_circ_0008302	+	ENSG00000117682;	Con_5Y1
novel_circ_0008445	-	ENSG00000092853;	Con_5Y1
novel_circ_0008591	+	ENSG00000162368;	Con_5Y1
novel_circ_0008640	-	ENSG00000078618;	Con_5Y1
novel_circ_0008654	-	ENSG00000134744;	Con_5Y1
novel_circ_0008754	+	ENSG00000162607;	Con_5Y1
novel_circ_0008815	+	ENSG00000116754;	Con_5Y1
novel_circ_0008854	-	ENSG00000077254;	Con_5Y1
novel_circ_0008957	-	ENSG00000122482;	Con_5Y1
novel_circ_0008960	+	ENSG00000189195;	Con_5Y1
novel_circ_0008993	+	ENSG00000122483;	Con_5Y1
novel_circ_0017752	+	ENSG00000119509;	Con_5Y1
novel_circ_0017814	+	ENSG00000157654;ENSG0	Con_5Y1
novel_circ_0017953	-	ENSG00000119414;	Con_5Y1
novel_circ_0017957	+	ENSG00000165219;	Con_5Y1
novel_circ_0018041	-	ENSG00000107290;	Con_5Y1

novel_circ_0018212	+	n/a	Con_5Y1
novel_circ_0018302	+	ENSG00000165304;	Con_5Y1
novel_circ_0018457	-	ENSG00000135052;	Con_5Y1
novel_circ_0016992	-	ENSG00000154359;	Con_5Y1
novel_circ_0017309	-	ENSG00000129696;	Con_5Y1
novel_circ_0017389	+	ENSG00000164808;	Con_5Y1
novel_circ_0017392	-	ENSG00000253729;	Con_5Y1
novel_circ_0017570	-	ENSG00000276418;	Con_5Y1
novel_circ_0017607	-	ENSG00000251136;	Con_5Y1
novel_circ_0017666	-	ENSG00000164944;	Con_5Y1
novel_circ_0017699	+	ENSG00000173273;	Con_5Y1
novel_circ_0017703	+	ENSG00000147649;	Con_5Y1
novel_circ_0017705	+	ENSG00000147649;	Con_5Y1
novel_circ_0017720	-	ENSG00000104375;	Con_5Y1
novel_circ_0017744	+	ENSG00000132549;	Con_5Y1
hsa_circ_0003450	-	ENSG00000123562;	Con_5Y1,Con_5Y2
hsa_circ_0001913	-	ENSG00000147010;	Con_5Y1,Con_5Y2
hsa_circ_0000509	+	ENSG00000185896;	Con_5Y1,Con_5Y2
hsa_circ_0025002	+	ENSG00000006831;	Con_5Y1,Con_5Y2
hsa_circ_0026153	+	ENSG00000123352;	Con_5Y1,Con_5Y2
hsa_circ_0025219	-	ENSG00000285238;ENSG0	Con_5Y1,Con_5Y2
hsa_circ_0025359	+	ENSG00000065970;	Con_5Y1,Con_5Y2
hsa_circ_0020875	-	ENSG00000110713;	Con_5Y1,Con_5Y2
hsa_circ_0004410	-	ENSG00000110713;	Con_5Y1,Con_5Y2
hsa_circ_0023229	+	ENSG00000110075;	Con_5Y1,Con_5Y2
hsa_circ_0023655	+	ENSG00000158636;	Con_5Y1,Con_5Y2
hsa_circ_0003546	-	ENSG00000184014;	Con_5Y1,Con_5Y2
hsa_circ_0006820	+	ENSG00000204152;ENSG0	Con_5Y1,Con_5Y2
hsa_circ_0003168	+	ENSG00000155256;	Con_5Y1,Con_5Y2
hsa_circ_0042835	+	ENSG00000108582;	Con_5Y1,Con_5Y2
hsa_circ_0007990	-	ENSG00000161395;	Con_5Y1,Con_5Y2
hsa_circ_0043816	-	ENSG00000168610;	Con_5Y1,Con_5Y2
hsa_circ_0044273	+	ENSG00000141279;	Con_5Y1,Con_5Y2
hsa_circ_0045440	+	ENSG00000171634;	Con_5Y1,Con_5Y2
hsa_circ_0041872	+	ENSG00000181222;	Con_5Y1,Con_5Y2
hsa_circ_0045788	-	ENSG00000129646;	Con_5Y1,Con_5Y2
hsa_circ_0002988	+	ENSG00000038532;	Con_5Y1,Con_5Y2
hsa_circ_0037875	-	ENSG00000153066;	Con_5Y1,Con_5Y2
hsa_circ_0003645	+	ENSG00000103544;	Con_5Y1,Con_5Y2
hsa_circ_0038737	-	ENSG00000077235;	Con_5Y1,Con_5Y2
hsa_circ_0040078	+	ENSG00000102908;	Con_5Y1,Con_5Y2
hsa_circ_0003398	-	ENSG00000128731;	Con_5Y1,Con_5Y2
hsa_circ_0000606	-	ENSG00000128915;	Con_5Y1,Con_5Y2
hsa_circ_0000614	+	ENSG00000028528;	Con_5Y1,Con_5Y2
hsa_circ_0035897	-	ENSG00000074603;	Con_5Y1,Con_5Y2
hsa_circ_0007837	+	ENSG00000140525;	Con_5Y1,Con_5Y2
hsa_circ_0031194	-	ENSG00000100888;	Con_5Y1,Con_5Y2
hsa_circ_0008568	-	ENSG00000258704;	Con_5Y1,Con_5Y2
hsa_circ_0007976	+	ENSG00000100644;	Con_5Y1,Con_5Y2
hsa_circ_0032384	+	ENSG00000100731;	Con_5Y1,Con_5Y2
hsa_circ_0008762	+	ENSG00000105364;	Con_5Y1,Con_5Y2
hsa_circ_0006392	+	ENSG00000141905;	Con_5Y1,Con_5Y2
hsa_circ_0050532	+	ENSG00000126261;	Con_5Y1,Con_5Y2
hsa_circ_0051123	+	ENSG00000090006;	Con_5Y1,Con_5Y2
hsa_circ_0005800	+	ENSG00000104880;ENSG0	Con_5Y1,Con_5Y2
hsa_circ_0047803	+	ENSG00000172175;	Con_5Y1,Con_5Y2
hsa_circ_0048027	+	ENSG00000060069;	Con_5Y1,Con_5Y2
hsa_circ_0059334	+	ENSG00000088888;	Con_5Y1,Con_5Y2

hsa_circ_0060517	+	ENSG00000101104;	Con_5Y1,Con_5Y2
hsa_circ_0060937	-	ENSG00000019186;	Con_5Y1,Con_5Y2
hsa_circ_0001740	+	ENSG00000128534;	Con_5Y1,Con_5Y2
hsa_circ_0079546	+	ENSG00000105866;	Con_5Y1,Con_5Y2
hsa_circ_0001717	+	ENSG00000106665;	Con_5Y1,Con_5Y2
hsa_circ_0008163	+	ENSG00000130429;ENSG0	Con_5Y1,Con_5Y2
hsa_circ_0002037	-	ENSG00000124571;	Con_5Y1,Con_5Y2
hsa_circ_0073942	-	ENSG00000113575;	Con_5Y1,Con_5Y2
hsa_circ_0007402	+	ENSG00000131503;ENSG0	Con_5Y1,Con_5Y2
hsa_circ_0074332	-	ENSG00000171720;	Con_5Y1,Con_5Y2
hsa_circ_0074704	+	ENSG00000155506;	Con_5Y1,Con_5Y2
hsa_circ_0072892	+	ENSG00000145734;	Con_5Y1,Con_5Y2
hsa_circ_0007860	+	ENSG00000138780;	Con_5Y1,Con_5Y2
hsa_circ_0069977	+	ENSG00000163738;	Con_5Y1,Con_5Y2
hsa_circ_0007096	+	ENSG00000138768;	Con_5Y1,Con_5Y2
hsa_circ_0070253	-	ENSG00000138674;	Con_5Y1,Con_5Y2
hsa_circ_0003626	-	ENSG00000118007;	Con_5Y1,Con_5Y2
hsa_circ_0068142	+	ENSG00000058063;	Con_5Y1,Con_5Y2
hsa_circ_0064557	-	ENSG00000182568;	Con_5Y1,Con_5Y2
hsa_circ_0068647	-	ENSG00000163960;	Con_5Y1,Con_5Y2
hsa_circ_0002639	-	ENSG00000144635;	Con_5Y1,Con_5Y2
hsa_circ_0065378	-	ENSG00000164050;	Con_5Y1,Con_5Y2
hsa_circ_0066271	+	ENSG00000163681;	Con_5Y1,Con_5Y2
hsa_circ_0066406	+	ENSG00000144724;	Con_5Y1,Con_5Y2
hsa_circ_0056244	+	ENSG00000088179;	Con_5Y1,Con_5Y2
hsa_circ_0002428	+	ENSG00000115419;	Con_5Y1,Con_5Y2
hsa_circ_0057908	-	ENSG00000023228;	Con_5Y1,Con_5Y2
hsa_circ_0058923	-	ENSG00000130414;	Con_5Y1,Con_5Y2
hsa_circ_0053439	+	ENSG00000115760;	Con_5Y1,Con_5Y2
hsa_circ_0054990	-	ENSG00000198369;	Con_5Y1,Con_5Y2
hsa_circ_0002863	-	ENSG00000198380;	Con_5Y1,Con_5Y2
hsa_circ_0006955	+	ENSG00000130939;	Con_5Y1,Con_5Y2
hsa_circ_0000017	-	ENSG00000171824;	Con_5Y1,Con_5Y2
hsa_circ_0002233	-	ENSG00000196505;	Con_5Y1,Con_5Y2
hsa_circ_0014022	+	ENSG00000143374;	Con_5Y1,Con_5Y2
hsa_circ_0006352	+	ENSG00000143379;	Con_5Y1,Con_5Y2
hsa_circ_0000130	+	ENSG00000143376;	Con_5Y1,Con_5Y2
hsa_circ_0010113	+	ENSG00000065526;	Con_5Y1,Con_5Y2
hsa_circ_0016408	+	ENSG00000162769;	Con_5Y1,Con_5Y2
hsa_circ_0011162	-	ENSG00000120656;	Con_5Y1,Con_5Y2
hsa_circ_0003099	+	ENSG00000066135;	Con_5Y1,Con_5Y2
hsa_circ_0009135	-	ENSG00000131697;	Con_5Y1,Con_5Y2
hsa_circ_0004607	-	ENSG00000153904;	Con_5Y1,Con_5Y2
hsa_circ_0088284	-	ENSG00000136861;	Con_5Y1,Con_5Y2
hsa_circ_0086296	+	ENSG00000147854;	Con_5Y1,Con_5Y2
hsa_circ_0009125	+	ENSG00000130956;	Con_5Y1,Con_5Y2
hsa_circ_0008921	-	ENSG00000155096;	Con_5Y1,Con_5Y2
hsa_circ_0085447	+	ENSG00000156787;	Con_5Y1,Con_5Y2
hsa_circ_0007779	-	ENSG00000189376;ENSG0	Con_5Y1,Con_5Y2
hsa_circ_0085694	-	ENSG00000167632;	Con_5Y1,Con_5Y2
hsa_circ_0084941	+	ENSG00000164941;	Con_5Y1,Con_5Y2
novel_circ_0018765	-	ENSG00000102098;	Con_5Y1,Con_5Y2
novel_circ_0018869	+	ENSG00000165591;	Con_5Y1,Con_5Y2
novel_circ_0002012	-	ENSG00000089060;	Con_5Y1,Con_5Y2
novel_circ_0002105	-	ENSG00000111011;	Con_5Y1,Con_5Y2
novel_circ_0002765	-	ENSG00000180263;	Con_5Y1,Con_5Y2
novel_circ_0001103	-	ENSG00000110328;	Con_5Y1,Con_5Y2
novel_circ_0001149	+	ENSG00000110395;	Con_5Y1,Con_5Y2

novel_circ_0001482	-	ENSG00000149532;	Con_5Y1,Con_5Y2
novel_circ_0000428	-	ENSG00000150867;	Con_5Y1,Con_5Y2
novel_circ_0006163	-	ENSG00000176155;	Con_5Y1,Con_5Y2
novel_circ_0005183	+	ENSG00000158545;	Con_5Y1,Con_5Y2
novel_circ_0003939	-	ENSG00000140470;	Con_5Y1,Con_5Y2
novel_circ_0003953	+	n/a	Con_5Y1,Con_5Y2
novel_circ_0004003	+	ENSG00000285077;ENSG0	Con_5Y1,Con_5Y2
novel_circ_0004064	-	ENSG00000128908;	Con_5Y1,Con_5Y2
novel_circ_0004073	+	ENSG00000137815;	Con_5Y1,Con_5Y2
novel_circ_0004126	+	ENSG00000104131;	Con_5Y1,Con_5Y2
novel_circ_0004190	-	ENSG00000128833;	Con_5Y1,Con_5Y2
novel_circ_0004246	+	ENSG00000140455;	Con_5Y1,Con_5Y2
novel_circ_0004465	+	ENSG00000140403;	Con_5Y1,Con_5Y2
novel_circ_0007058	+	n/a	Con_5Y1,Con_5Y2
novel_circ_0006475	-	ENSG00000172361;	Con_5Y1,Con_5Y2
novel_circ_0006595	+	ENSG00000166377;	Con_5Y1,Con_5Y2
novel_circ_0009774	-	ENSG00000180957;	Con_5Y1,Con_5Y2
novel_circ_0009943	+	ENSG00000248405;ENSG0	Con_5Y1,Con_5Y2
novel_circ_0009071	-	ENSG00000089048;	Con_5Y1,Con_5Y2
novel_circ_0015866	-	ENSG00000164815;	Con_5Y1,Con_5Y2
novel_circ_0016176	+	ENSG00000009335;	Con_5Y1,Con_5Y2
novel_circ_0016701	+	ENSG00000105793;	Con_5Y1,Con_5Y2
novel_circ_0015097	+	ENSG00000118507;	Con_5Y1,Con_5Y2
novel_circ_0015386	-	ENSG00000124795;	Con_5Y1,Con_5Y2
novel_circ_0015788	-	ENSG00000146263;	Con_5Y1,Con_5Y2
novel_circ_0014921	-	ENSG00000153922;	Con_5Y1,Con_5Y2
novel_circ_0013318	-	ENSG00000198589;	Con_5Y1,Con_5Y2
novel_circ_0013632	-	ENSG00000121892;	Con_5Y1,Con_5Y2
novel_circ_0011804	+	ENSG00000177707;	Con_5Y1,Con_5Y2
novel_circ_0011921	-	ENSG00000082781;	Con_5Y1,Con_5Y2
novel_circ_0012324	+	ENSG00000131374;	Con_5Y1,Con_5Y2
novel_circ_0012702	+	ENSG00000179152;	Con_5Y1,Con_5Y2
novel_circ_0012855	+	ENSG00000164081;	Con_5Y1,Con_5Y2
novel_circ_0010266	+	ENSG00000080345;	Con_5Y1,Con_5Y2
novel_circ_0010839	+	ENSG00000144468;	Con_5Y1,Con_5Y2
novel_circ_0011014	-	ENSG00000138101;	Con_5Y1,Con_5Y2
novel_circ_0011183	-	ENSG00000171055;	Con_5Y1,Con_5Y2
novel_circ_0011221	-	ENSG00000115904;	Con_5Y1,Con_5Y2
novel_circ_0011430	-	ENSG00000115464;	Con_5Y1,Con_5Y2
novel_circ_0011624	-	ENSG00000159374;	Con_5Y1,Con_5Y2
novel_circ_0011647	+	ENSG00000168883;	Con_5Y1,Con_5Y2
novel_circ_0007351	-	ENSG00000143401;	Con_5Y1,Con_5Y2
novel_circ_0007355	+	ENSG00000117360;	Con_5Y1,Con_5Y2
novel_circ_0007547	-	ENSG00000152382;	Con_5Y1,Con_5Y2
novel_circ_0007686	+	ENSG00000162782;	Con_5Y1,Con_5Y2
novel_circ_0007764	+	ENSG00000116747;	Con_5Y1,Con_5Y2
novel_circ_0007869	-	ENSG00000127483;	Con_5Y1,Con_5Y2
novel_circ_0008222	+	ENSG00000174371;	Con_5Y1,Con_5Y2
novel_circ_0008663	-	ENSG00000134744;	Con_5Y1,Con_5Y2
novel_circ_0008998	+	ENSG00000122483;	Con_5Y1,Con_5Y2
novel_circ_0018094	+	ENSG00000177239;	Con_5Y1,Con_5Y2
novel_circ_0018185	+	ENSG00000099810;	Con_5Y1,Con_5Y2
novel_circ_0016825	-	ENSG00000104517;	Con_5Y1,Con_5Y2
novel_circ_0016842	-	ENSG00000155096;	Con_5Y1,Con_5Y2
novel_circ_0016872	+	ENSG00000164830;	Con_5Y1,Con_5Y2
novel_circ_0017357	-	ENSG00000120925;	Con_5Y1,Con_5Y2
novel_circ_0017506	+	ENSG00000104218;	Con_5Y1,Con_5Y2
novel_circ_0017533	-	ENSG00000066777;	Con_5Y1,Con_5Y2

novel_circ_0017735	+	ENSG00000132549;	Con_5Y1,Con_5Y2
hsa_circ_0030012	-	ENSG00000102710;	Con_5Y1,Con_5Y2,C
hsa_circ_0006620	+	ENSG00000136156;	Con_5Y1,Con_5Y2,C
hsa_circ_0008803	+	ENSG00000022840;	Con_5Y1,Con_5Y2,C
hsa_circ_0002770	+	ENSG00000135679;	Con_5Y1,Con_5Y2,C
hsa_circ_0003541	+	ENSG00000149289;	Con_5Y1,Con_5Y2,C
hsa_circ_0022614	+	ENSG00000072518;	Con_5Y1,Con_5Y2,C
hsa_circ_0005611	+	ENSG00000173715;	Con_5Y1,Con_5Y2,C
hsa_circ_0000214	+	ENSG00000181192;	Con_5Y1,Con_5Y2,C
hsa_circ_0042231	+	ENSG00000133030;	Con_5Y1,Con_5Y2,C
hsa_circ_0042493	+	ENSG00000109046;	Con_5Y1,Con_5Y2,C
hsa_circ_0004621	+	ENSG00000029725;	Con_5Y1,Con_5Y2,C
hsa_circ_0000678	+	ENSG00000103222;	Con_5Y1,Con_5Y2,C
hsa_circ_0038374	+	ENSG00000174628;	Con_5Y1,Con_5Y2,C
hsa_circ_0034982	-	ENSG00000067369;	Con_5Y1,Con_5Y2,C
hsa_circ_0003867	+	ENSG00000028528;	Con_5Y1,Con_5Y2,C
hsa_circ_0004008	+	ENSG00000100731;	Con_5Y1,Con_5Y2,C
hsa_circ_0008187	+	ENSG00000119685;	Con_5Y1,Con_5Y2,C
hsa_circ_0032939	-	ENSG00000100784;	Con_5Y1,Con_5Y2,C
hsa_circ_0008287	+	ENSG00000076650;	Con_5Y1,Con_5Y2,C
hsa_circ_0047964	+	ENSG00000215421;	Con_5Y1,Con_5Y2,C
hsa_circ_0059545	+	ENSG00000101310;	Con_5Y1,Con_5Y2,C
hsa_circ_0008117	+	ENSG00000124191;	Con_5Y1,Con_5Y2,C
hsa_circ_0005113	+	ENSG00000009335;	Con_5Y1,Con_5Y2,C
hsa_circ_0004299	+	ENSG00000284292;ENSG0	Con_5Y1,Con_5Y2,C
hsa_circ_0077520	-	ENSG00000085382;	Con_5Y1,Con_5Y2,C
hsa_circ_0003105	-	ENSG00000152894;	Con_5Y1,Con_5Y2,C
hsa_circ_0003016	+	ENSG00000112584;	Con_5Y1,Con_5Y2,C
hsa_circ_0074323	-	ENSG00000131504;	Con_5Y1,Con_5Y2,C
hsa_circ_0001097	+	ENSG00000115020;	Con_5Y1,Con_5Y2,C
hsa_circ_0003990	+	ENSG00000057935;	Con_5Y1,Con_5Y2,C
hsa_circ_0011245	-	ENSG00000134644;	Con_5Y1,Con_5Y2,C
hsa_circ_0002467	+	ENSG00000121774;	Con_5Y1,Con_5Y2,C
hsa_circ_0004073	+	ENSG00000181090;	Con_5Y1,Con_5Y2,C
hsa_circ_0084665	+	ENSG00000104218;	Con_5Y1,Con_5Y2,C
novel_circ_0018874	-	ENSG00000131089;	Con_5Y1,Con_5Y2,C
novel_circ_0000101	+	ENSG00000138166;	Con_5Y1,Con_5Y2,C
novel_circ_0000361	-	ENSG00000165752;	Con_5Y1,Con_5Y2,C
novel_circ_0006182	+	ENSG00000141568;	Con_5Y1,Con_5Y2,C
novel_circ_0004659	-	ENSG00000187535;	Con_5Y1,Con_5Y2,C
novel_circ_0004760	+	ENSG00000090905;	Con_5Y1,Con_5Y2,C
novel_circ_0004400	+	ENSG00000067141;	Con_5Y1,Con_5Y2,C
novel_circ_0006726	+	ENSG00000123143;	Con_5Y1,Con_5Y2,C
novel_circ_0006823	-	ENSG00000104969;	Con_5Y1,Con_5Y2,C
novel_circ_0006362	-	ENSG00000141447;	Con_5Y1,Con_5Y2,C
novel_circ_0006617	+	ENSG00000168502;	Con_5Y1,Con_5Y2,C
novel_circ_0009711	+	ENSG00000099917;	Con_5Y1,Con_5Y2,C
novel_circ_0015874	+	ENSG00000005483;	Con_5Y1,Con_5Y2,C
novel_circ_0016480	-	ENSG00000188191;	Con_5Y1,Con_5Y2,C
novel_circ_0014210	-	ENSG00000120725;	Con_5Y1,Con_5Y2,C
novel_circ_0014436	+	ENSG00000176783;	Con_5Y1,Con_5Y2,C
novel_circ_0013461	-	n/a	Con_5Y1,Con_5Y2,C
novel_circ_0012013	-	ENSG00000274810;	Con_5Y1,Con_5Y2,C
novel_circ_0012700	-	ENSG00000144455;	Con_5Y1,Con_5Y2,C
novel_circ_0010796	+	ENSG00000239498;	Con_5Y1,Con_5Y2,C
novel_circ_0008738	+	ENSG00000132849;	Con_5Y1,Con_5Y2,C
hsa_circ_0091546	-	ENSG00000213468;	Con_5Y1,Con_5Y3
hsa_circ_0008760	+	ENSG00000152520;	Con_5Y1,Con_5Y3

hsa_circ_0002748	-	ENSG00000123066;	Con_5Y1,Con_5Y3
hsa_circ_0028587	-	ENSG00000123066;	Con_5Y1,Con_5Y3
hsa_circ_0008087	+	ENSG00000153179;	Con_5Y1,Con_5Y3
hsa_circ_0024236	+	ENSG00000149311;	Con_5Y1,Con_5Y3
hsa_circ_0003900	-	ENSG00000167986;	Con_5Y1,Con_5Y3
hsa_circ_0005282	-	ENSG00000110090;	Con_5Y1,Con_5Y3
hsa_circ_0006534	-	ENSG00000136758;	Con_5Y1,Con_5Y3
hsa_circ_0002498	-	ENSG00000107959;	Con_5Y1,Con_5Y3
hsa_circ_0002487	-	ENSG00000148498;	Con_5Y1,Con_5Y3
hsa_circ_0003023	-	ENSG00000151240;	Con_5Y1,Con_5Y3
hsa_circ_0006148	+	ENSG00000156671;	Con_5Y1,Con_5Y3
hsa_circ_0000744	-	ENSG00000141027;	Con_5Y1,Con_5Y3
hsa_circ_0042977	+	ENSG00000178691;	Con_5Y1,Con_5Y3
hsa_circ_0043282	+	ENSG00000276234;	Con_5Y1,Con_5Y3
hsa_circ_0045218	-	ENSG00000108588;	Con_5Y1,Con_5Y3
hsa_circ_0006264	+	ENSG00000173821;	Con_5Y1,Con_5Y3
hsa_circ_0040528	-	ENSG00000103091;	Con_5Y1,Con_5Y3
hsa_circ_0040905	+	ENSG00000176715;	Con_5Y1,Con_5Y3
hsa_circ_0041011	-	ENSG00000187741;	Con_5Y1,Con_5Y3
hsa_circ_0034694	+	ENSG00000285920;ENSG0	Con_5Y1,Con_5Y3
hsa_circ_0035929	-	ENSG00000174485;	Con_5Y1,Con_5Y3
hsa_circ_0007836	+	ENSG00000132003;	Con_5Y1,Con_5Y3
hsa_circ_0005617	+	ENSG00000198089;	Con_5Y1,Con_5Y3
hsa_circ_0007130	+	ENSG00000025293;	Con_5Y1,Con_5Y3
hsa_circ_0002660	+	ENSG00000159128;	Con_5Y1,Con_5Y3
hsa_circ_0008795	-	ENSG00000159131;	Con_5Y1,Con_5Y3
hsa_circ_0061722	-	ENSG00000185658;	Con_5Y1,Con_5Y3
hsa_circ_0079628	+	ENSG00000105926;	Con_5Y1,Con_5Y3
hsa_circ_0077535	-	ENSG00000057663;	Con_5Y1,Con_5Y3
hsa_circ_0001647	+	ENSG00000152818;	Con_5Y1,Con_5Y3
hsa_circ_0076558	-	ENSG00000124571;	Con_5Y1,Con_5Y3
hsa_circ_0073904	-	ENSG00000072364;	Con_5Y1,Con_5Y3
hsa_circ_0006694	+	ENSG00000145907;	Con_5Y1,Con_5Y3
hsa_circ_0003868	-	ENSG00000135074;	Con_5Y1,Con_5Y3
hsa_circ_0006970	+	ENSG00000113460;	Con_5Y1,Con_5Y3
hsa_circ_0072758	+	ENSG00000134057;	Con_5Y1,Con_5Y3
hsa_circ_0007080	+	ENSG00000158234;	Con_5Y1,Con_5Y3
hsa_circ_0068435	-	ENSG00000244405;	Con_5Y1,Con_5Y3
hsa_circ_0066434	+	ENSG00000163635;ENSG0	Con_5Y1,Con_5Y3
hsa_circ_0005995	+	ENSG00000143379;	Con_5Y1,Con_5Y3
hsa_circ_0016905	+	ENSG00000270106;	Con_5Y1,Con_5Y3
hsa_circ_0005057	-	ENSG00000116514;	Con_5Y1,Con_5Y3
hsa_circ_0004877	-	ENSG00000085832;	Con_5Y1,Con_5Y3
hsa_circ_0012742	-	ENSG00000162601;	Con_5Y1,Con_5Y3
hsa_circ_0012942	-	ENSG00000118454;	Con_5Y1,Con_5Y3
hsa_circ_0003762	+	ENSG00000086061;	Con_5Y1,Con_5Y3
hsa_circ_0087248	+	ENSG00000197969;	Con_5Y1,Con_5Y3
hsa_circ_0008577	+	ENSG00000158079;	Con_5Y1,Con_5Y3
novel_circ_0002873	+	ENSG00000121741;	Con_5Y1,Con_5Y3
novel_circ_0002640	+	ENSG00000166226;	Con_5Y1,Con_5Y3
novel_circ_0002775	+	ENSG00000139350;	Con_5Y1,Con_5Y3
novel_circ_0001315	+	ENSG00000149100;	Con_5Y1,Con_5Y3
novel_circ_0001670	-	ENSG00000048649;	Con_5Y1,Con_5Y3
novel_circ_0001766	+	ENSG00000182919;ENSG0	Con_5Y1,Con_5Y3
novel_circ_0000404	-	ENSG00000241058;	Con_5Y1,Con_5Y3
novel_circ_0000432	-	n/a	Con_5Y1,Con_5Y3
novel_circ_0000914	+	ENSG00000138160;	Con_5Y1,Con_5Y3
novel_circ_0000992	-	ENSG00000107554;	Con_5Y1,Con_5Y3

novel_circ_0005377	+	ENSG00000007168;	Con_5Y1,Con_5Y3
novel_circ_0005751	+	ENSG00000261879;	Con_5Y1,Con_5Y3
novel_circ_0006011	+	ENSG00000141337;	Con_5Y1,Con_5Y3
novel_circ_0005113	-	ENSG00000153774;	Con_5Y1,Con_5Y3
novel_circ_0005114	-	ENSG00000153774;	Con_5Y1,Con_5Y3
novel_circ_0003526	+	ENSG00000258526;	Con_5Y1,Con_5Y3
novel_circ_0003889	+	ENSG00000066455;	Con_5Y1,Con_5Y3
novel_circ_0009389	-	ENSG00000101126;	Con_5Y1,Con_5Y3
novel_circ_0016102	-	ENSG00000133624;	Con_5Y1,Con_5Y3
novel_circ_0016674	-	ENSG00000003147;	Con_5Y1,Con_5Y3
novel_circ_0014116	+	ENSG00000113522;ENSG0	Con_5Y1,Con_5Y3
novel_circ_0014153	+	ENSG00000145833;	Con_5Y1,Con_5Y3
novel_circ_0013627	-	ENSG00000121892;	Con_5Y1,Con_5Y3
novel_circ_0013675	-	ENSG00000145220;	Con_5Y1,Con_5Y3
novel_circ_0013730	+	ENSG00000145216;ENSG0	Con_5Y1,Con_5Y3
novel_circ_0011906	+	ENSG00000065485;	Con_5Y1,Con_5Y3
novel_circ_0012254	-	ENSG00000154310;	Con_5Y1,Con_5Y3
novel_circ_0010028	+	ENSG00000170417;	Con_5Y1,Con_5Y3
novel_circ_0010170	-	ENSG00000144233;	Con_5Y1,Con_5Y3
novel_circ_0010231	+	ENSG00000144224;	Con_5Y1,Con_5Y3
novel_circ_0010666	+	ENSG00000144426;	Con_5Y1,Con_5Y3
novel_circ_0010897	+	ENSG00000204120;	Con_5Y1,Con_5Y3
novel_circ_0007300	+	ENSG00000083444;	Con_5Y1,Con_5Y3
novel_circ_0007437	-	ENSG00000116539;	Con_5Y1,Con_5Y3
novel_circ_0007542	-	ENSG00000215908;	Con_5Y1,Con_5Y3
novel_circ_0007739	+	n/a	Con_5Y1,Con_5Y3
novel_circ_0008742	+	ENSG00000132849;	Con_5Y1,Con_5Y3
novel_circ_0016946	+	ENSG00000156787;	Con_5Y1,Con_5Y3
novel_circ_0016971	+	ENSG00000104549;	Con_5Y1,Con_5Y3
novel_circ_0017216	+	ENSG00000104611;	Con_5Y1,Con_5Y3
novel_circ_0017628	+	ENSG00000214954;	Con_5Y1,Con_5Y3
hsa_circ_0002724	-	ENSG00000086758;	Con_5Y2
hsa_circ_0091092	-	ENSG00000085224;	Con_5Y2
hsa_circ_0004790	-	ENSG00000185989;	Con_5Y2
hsa_circ_0000465	+	ENSG00000121741;	Con_5Y2
hsa_circ_0008007	-	ENSG00000132953;	Con_5Y2
hsa_circ_0029684	-	ENSG00000132953;	Con_5Y2
hsa_circ_0005874	+	ENSG00000132952;	Con_5Y2
hsa_circ_0029929	+	ENSG00000139618;	Con_5Y2
hsa_circ_0030200	+	ENSG00000136141;	Con_5Y2
hsa_circ_0030340	-	ENSG00000136100;	Con_5Y2
hsa_circ_0030425	-	ENSG00000136111;	Con_5Y2
hsa_circ_0030547	+	ENSG00000102471;	Con_5Y2
hsa_circ_0027905	-	ENSG00000111670;	Con_5Y2
hsa_circ_0028048	-	ENSG00000110851;	Con_5Y2
hsa_circ_0028608	+	ENSG00000174989;	Con_5Y2
hsa_circ_0003902	-	ENSG00000122966;	Con_5Y2
hsa_circ_0006615	-	ENSG00000051825;	Con_5Y2
hsa_circ_0029320	-	ENSG00000196498;	Con_5Y2
hsa_circ_0024996	+	ENSG00000082805;	Con_5Y2
hsa_circ_0004021	+	ENSG00000185684;	Con_5Y2
hsa_circ_0025850	+	ENSG00000087470;	Con_5Y2
hsa_circ_0006018	+	ENSG00000189079;	Con_5Y2
hsa_circ_0027471	+	ENSG00000111581;	Con_5Y2
hsa_circ_0027478	+	ENSG00000111581;	Con_5Y2
hsa_circ_0027479	+	ENSG00000111581;	Con_5Y2
hsa_circ_0000424	-	ENSG00000058272;	Con_5Y2
hsa_circ_0003251	+	ENSG00000060237;	Con_5Y2

hsa_circ_0024986	-	ENSG00000002016;	Con_5Y2
hsa_circ_0024477	+	ENSG00000095139;	Con_5Y2
hsa_circ_0003015	+	ENSG00000187079;	Con_5Y2
hsa_circ_0024917	-	ENSG00000151503;	Con_5Y2
hsa_circ_0021386	-	ENSG00000011405;	Con_5Y2
hsa_circ_0000279	-	ENSG00000110756;	Con_5Y2
hsa_circ_0021572	+	ENSG00000060749;	Con_5Y2
hsa_circ_0020917	-	ENSG00000110713;	Con_5Y2
hsa_circ_0021929	-	ENSG00000175216;	Con_5Y2
hsa_circ_0022306	-	ENSG00000167986;	Con_5Y2
hsa_circ_0022378	-	ENSG00000149485;	Con_5Y2
hsa_circ_0009159	+	ENSG00000168005;	Con_5Y2
hsa_circ_0023232	+	ENSG00000110075;	Con_5Y2
hsa_circ_0004411	-	ENSG00000110090;	Con_5Y2
hsa_circ_0007722	-	ENSG00000186635;	Con_5Y2
hsa_circ_0007169	-	ENSG00000168014;	Con_5Y2
hsa_circ_0000342	-	ENSG00000149269;	Con_5Y2
hsa_circ_0007767	-	ENSG00000159063;	Con_5Y2
hsa_circ_0007455	+	ENSG00000074266;	Con_5Y2
hsa_circ_0020052	+	ENSG00000148737;	Con_5Y2
hsa_circ_0020250	-	ENSG00000107669;	Con_5Y2
hsa_circ_0020310	+	ENSG00000107902;	Con_5Y2
hsa_circ_0020311	+	ENSG00000107902;	Con_5Y2
hsa_circ_0007825	-	ENSG00000258539;	Con_5Y2
hsa_circ_0020460	+	ENSG00000150760;	Con_5Y2
hsa_circ_0001963	+	ENSG00000148459;	Con_5Y2
hsa_circ_0018019	-	ENSG00000107890;	Con_5Y2
hsa_circ_0018038	+	ENSG00000120539;	Con_5Y2
hsa_circ_0005144	-	ENSG00000171988;	Con_5Y2
hsa_circ_0018761	+	ENSG00000156026;	Con_5Y2
hsa_circ_0018887	+	ENSG00000035403;	Con_5Y2
hsa_circ_0019003	-	ENSG00000062650;	Con_5Y2
hsa_circ_0006293	+	ENSG00000198060;	Con_5Y2
hsa_circ_0019208	-	ENSG00000138119;	Con_5Y2
hsa_circ_0000732	-	ENSG00000074660;	Con_5Y2
hsa_circ_0042419	-	ENSG00000108599;	Con_5Y2
hsa_circ_0042426	+	ENSG00000128487;	Con_5Y2
hsa_circ_0004192	-	ENSG00000070366;	Con_5Y2
hsa_circ_0042838	+	ENSG00000108582;	Con_5Y2
hsa_circ_0042888	+	ENSG00000196712;	Con_5Y2
hsa_circ_0043333	+	ENSG00000274211;	Con_5Y2
hsa_circ_0043462	+	ENSG00000141736;	Con_5Y2
hsa_circ_0002899	-	ENSG00000074755;	Con_5Y2
hsa_circ_0041551	-	ENSG00000185722;	Con_5Y2
hsa_circ_0008601	-	ENSG00000108883;	Con_5Y2
hsa_circ_0044836	+	ENSG00000153982;	Con_5Y2
hsa_circ_0045047	-	ENSG00000108506;	Con_5Y2
hsa_circ_0045409	-	ENSG00000198265;	Con_5Y2
hsa_circ_0045462	+	ENSG00000171634;	Con_5Y2
hsa_circ_0008557	-	ENSG00000161533;	Con_5Y2
hsa_circ_0008891	+	ENSG00000163597;	Con_5Y2
hsa_circ_0006122	+	ENSG00000141519;	Con_5Y2
hsa_circ_0046131	-	ENSG00000157637;	Con_5Y2
hsa_circ_0006462	-	ENSG00000182446;	Con_5Y2
hsa_circ_0002445	-	ENSG00000059145;	Con_5Y2
hsa_circ_0038063	+	ENSG00000072864;	Con_5Y2
hsa_circ_0038247	-	ENSG00000157106;	Con_5Y2
hsa_circ_0038748	-	ENSG00000077235;	Con_5Y2

hsa_circ_0037563	+	ENSG00000167978;	Con_5Y2
hsa_circ_0006982	-	ENSG00000125107;	Con_5Y2
hsa_circ_0039914	+	ENSG00000167264;	Con_5Y2
hsa_circ_0005615	+	ENSG00000072736;	Con_5Y2
hsa_circ_0039945	+	ENSG00000103064;	Con_5Y2
hsa_circ_0040082	-	ENSG00000181019;	Con_5Y2
hsa_circ_0040191	-	ENSG00000090861;	Con_5Y2
hsa_circ_0002505	-	ENSG00000166747;	Con_5Y2
hsa_circ_0040498	-	ENSG00000168411;	Con_5Y2
hsa_circ_0006418	-	ENSG00000185418;	Con_5Y2
hsa_circ_0034065	+	ENSG00000273749;	Con_5Y2
hsa_circ_0007659	+	ENSG00000166922;	Con_5Y2
hsa_circ_0034557	+	ENSG00000128829;	Con_5Y2
hsa_circ_0034604	+	ENSG00000137812;	Con_5Y2
hsa_circ_0034967	+	ENSG00000137822;	Con_5Y2
hsa_circ_0035055	+	ENSG00000137770;	Con_5Y2
hsa_circ_0035572	-	ENSG00000128915;	Con_5Y2
hsa_circ_0006487	-	ENSG00000074603;	Con_5Y2
hsa_circ_0000640	-	ENSG00000140386;	Con_5Y2
hsa_circ_0005889	+	ENSG00000073417;	Con_5Y2
hsa_circ_0003679	+	ENSG00000140526;	Con_5Y2
hsa_circ_0036926	-	ENSG00000284946;ENSG0	Con_5Y2
hsa_circ_0000654	+	ENSG00000173575;ENSG0	Con_5Y2
hsa_circ_0003831	+	ENSG00000078304;	Con_5Y2
hsa_circ_0003589	+	ENSG00000197102;	Con_5Y2
hsa_circ_0004399	-	ENSG00000070367;	Con_5Y2
hsa_circ_0032125	+	ENSG00000027075;	Con_5Y2
hsa_circ_0032248	+	ENSG00000033170;	Con_5Y2
hsa_circ_0032349	+	ENSG00000029364;	Con_5Y2
hsa_circ_0032708	+	ENSG00000119685;	Con_5Y2
hsa_circ_0032931	-	ENSG00000165914;	Con_5Y2
hsa_circ_0033155	+	ENSG00000066629;	Con_5Y2
hsa_circ_0049899	+	ENSG00000127511;	Con_5Y2
hsa_circ_0000909	+	ENSG00000099331;	Con_5Y2
hsa_circ_0002600	+	ENSG00000105700;	Con_5Y2
hsa_circ_0008135	-	ENSG00000051128;	Con_5Y2
hsa_circ_0005126	-	ENSG00000105717;	Con_5Y2
hsa_circ_0008186	+	ENSG00000104885;	Con_5Y2
hsa_circ_0050462	+	ENSG00000257103;	Con_5Y2
hsa_circ_0008033	+	ENSG00000105220;	Con_5Y2
hsa_circ_0003090	-	ENSG00000011422;	Con_5Y2
hsa_circ_0051427	+	ENSG00000104856;	Con_5Y2
hsa_circ_0051493	-	ENSG00000012061;	Con_5Y2
hsa_circ_0048987	+	ENSG00000032444;	Con_5Y2
hsa_circ_0008741	+	ENSG00000101752;	Con_5Y2
hsa_circ_0047348	-	ENSG00000118276;	Con_5Y2
hsa_circ_0047891	+	ENSG00000081913;	Con_5Y2
hsa_circ_0005815	-	ENSG00000141759;	Con_5Y2
hsa_circ_0046843	+	ENSG00000101745;	Con_5Y2
hsa_circ_0006707	-	ENSG00000154845;	Con_5Y2
hsa_circ_0001212	-	ENSG00000228315;ENSG0	Con_5Y2
hsa_circ_0004208	+	ENSG00000099956;	Con_5Y2
hsa_circ_0062608	+	ENSG00000100014;ENSG0	Con_5Y2
hsa_circ_0062638	-	ENSG00000100068;	Con_5Y2
hsa_circ_0062656	+	ENSG00000100077;	Con_5Y2
hsa_circ_0062675	-	ENSG00000100109;	Con_5Y2
hsa_circ_0006303	+	ENSG00000100330;	Con_5Y2
hsa_circ_0006331	-	ENSG00000184708;	Con_5Y2

hsa_circ_0063153	-	ENSG00000100345;	Con_5Y2
hsa_circ_0001230	-	ENSG00000100201;	Con_5Y2
hsa_circ_0063605	+	ENSG00000100147;	Con_5Y2
hsa_circ_0001243	-	n/a	Con_5Y2
hsa_circ_0059304	+	ENSG00000088812;	Con_5Y2
hsa_circ_0005163	+	ENSG00000025293;	Con_5Y2
hsa_circ_0007050	+	ENSG00000132792;	Con_5Y2
hsa_circ_0060456	+	ENSG00000101057;	Con_5Y2
hsa_circ_0002615	-	ENSG00000132824;	Con_5Y2
hsa_circ_0060516	+	ENSG00000101104;	Con_5Y2
hsa_circ_0059365	-	ENSG00000089057;	Con_5Y2
hsa_circ_0060730	+	ENSG00000124207;	Con_5Y2
hsa_circ_0002536	+	ENSG00000124207;	Con_5Y2
hsa_circ_0009105	+	ENSG00000125885;ENSG0	Con_5Y2
hsa_circ_0006854	-	ENSG00000130699;	Con_5Y2
hsa_circ_0008725	+	ENSG00000159128;	Con_5Y2
hsa_circ_0061757	-	ENSG00000185658;	Con_5Y2
hsa_circ_0007126	-	ENSG00000160294;	Con_5Y2
hsa_circ_0081536	+	ENSG00000146828;	Con_5Y2
hsa_circ_0007459	-	ENSG00000091127;	Con_5Y2
hsa_circ_0081891	-	ENSG00000164597;	Con_5Y2
hsa_circ_0082648	+	ENSG00000157741;	Con_5Y2
hsa_circ_0082922	+	ENSG00000133612;	Con_5Y2
hsa_circ_0005860	-	ENSG00000286192;ENSG0	Con_5Y2
hsa_circ_0079572	+	ENSG00000122550;	Con_5Y2
hsa_circ_0004869	+	ENSG00000106263;	Con_5Y2
hsa_circ_0001956	+	ENSG00000122547;	Con_5Y2
hsa_circ_0079336	+	ENSG00000106346;	Con_5Y2
hsa_circ_0005619	+	ENSG00000263001;	Con_5Y2
hsa_circ_0079074	+	ENSG00000164828;	Con_5Y2
hsa_circ_0006991	+	ENSG00000164465;	Con_5Y2
hsa_circ_0077825	-	ENSG00000146376;	Con_5Y2
hsa_circ_0077851	-	ENSG00000112282;	Con_5Y2
hsa_circ_0005753	+	ENSG00000055208;	Con_5Y2
hsa_circ_0078208	-	ENSG00000131013;	Con_5Y2
hsa_circ_0078297	+	ENSG00000120254;	Con_5Y2
hsa_circ_0005472	-	ENSG00000285441;ENSG0	Con_5Y2
hsa_circ_0004662	-	ENSG00000285441;ENSG0	Con_5Y2
hsa_circ_0075805	+	ENSG00000112242;	Con_5Y2
hsa_circ_0076055	+	ENSG00000065060;	Con_5Y2
hsa_circ_0076056	+	ENSG00000065060;	Con_5Y2
hsa_circ_0076150	-	ENSG00000096060;	Con_5Y2
hsa_circ_0004035	+	ENSG00000170734;	Con_5Y2
hsa_circ_0075522	+	ENSG00000153046;	Con_5Y2
hsa_circ_0077416	-	ENSG00000112234;	Con_5Y2
hsa_circ_0006072	+	ENSG00000151422;	Con_5Y2
hsa_circ_0005631	+	ENSG00000038382;	Con_5Y2
hsa_circ_0071903	+	ENSG00000038382;	Con_5Y2
hsa_circ_0074665	+	ENSG00000145907;	Con_5Y2
hsa_circ_0074744	-	ENSG00000082516;	Con_5Y2
hsa_circ_0006386	-	ENSG00000120137;	Con_5Y2
hsa_circ_0075026	-	ENSG00000072803;	Con_5Y2
hsa_circ_0072056	-	ENSG00000113360;	Con_5Y2
hsa_circ_0072263	-	ENSG00000113569;	Con_5Y2
hsa_circ_0072629	+	ENSG00000086200;	Con_5Y2
hsa_circ_0072756	+	ENSG00000145740;	Con_5Y2
hsa_circ_0073131	-	ENSG00000152413;	Con_5Y2
hsa_circ_0073329	+	ENSG00000164199;	Con_5Y2

hsa_circ_0007668	-	ENSG00000138785;	Con_5Y2
hsa_circ_0070574	-	ENSG00000138785;	Con_5Y2
hsa_circ_0070649	+	ENSG00000138802;	Con_5Y2
hsa_circ_0070916	+	ENSG00000142731;	Con_5Y2
hsa_circ_0068842	+	ENSG00000090316;	Con_5Y2
hsa_circ_0071198	-	ENSG00000198589;	Con_5Y2
hsa_circ_0071228	-	ENSG00000198589;	Con_5Y2
hsa_circ_0069397	-	ENSG00000047365;	Con_5Y2
hsa_circ_0069032	-	ENSG00000145220;	Con_5Y2
hsa_circ_0069651	-	ENSG00000170448;	Con_5Y2
hsa_circ_0070111	+	ENSG00000138759;	Con_5Y2
hsa_circ_0070132	+	ENSG00000138759;	Con_5Y2
hsa_circ_0070303	-	ENSG00000163625;	Con_5Y2
hsa_circ_0070388	-	ENSG00000145332;	Con_5Y2
hsa_circ_0006387	+	ENSG00000138641;	Con_5Y2
hsa_circ_0070440	-	ENSG00000138640;	Con_5Y2
hsa_circ_0069191	-	ENSG00000109667;	Con_5Y2
hsa_circ_0066701	+	ENSG00000182504;	Con_5Y2
hsa_circ_0066986	-	ENSG00000114030;	Con_5Y2
hsa_circ_0064360	-	ENSG00000132155;	Con_5Y2
hsa_circ_0067347	+	ENSG00000114670;	Con_5Y2
hsa_circ_0067900	-	ENSG00000173889;	Con_5Y2
hsa_circ_0068355	+	ENSG00000156931;	Con_5Y2
hsa_circ_0005286	-	ENSG00000244405;	Con_5Y2
hsa_circ_0002381	+	ENSG00000076242;	Con_5Y2
hsa_circ_0065140	-	ENSG00000181555;	Con_5Y2
hsa_circ_0003311	+	ENSG00000135968;	Con_5Y2
hsa_circ_0056199	+	ENSG00000115091;	Con_5Y2
hsa_circ_0056709	+	ENSG00000080345;	Con_5Y2
hsa_circ_0056714	+	ENSG00000080345;	Con_5Y2
hsa_circ_0057050	+	ENSG00000115827;	Con_5Y2
hsa_circ_0057510	+	ENSG00000138386;	Con_5Y2
hsa_circ_0005395	+	ENSG00000144426;	Con_5Y2
hsa_circ_0006734	+	ENSG00000144426;	Con_5Y2
hsa_circ_0058175	+	ENSG00000079246;	Con_5Y2
hsa_circ_0004783	-	ENSG00000135913;	Con_5Y2
hsa_circ_0005355	-	ENSG00000085449;	Con_5Y2
hsa_circ_0058518	+	ENSG00000173744;	Con_5Y2
hsa_circ_0058691	+	ENSG00000144535;	Con_5Y2
hsa_circ_0058699	+	ENSG00000144535;	Con_5Y2
hsa_circ_0003585	+	ENSG00000085978;	Con_5Y2
hsa_circ_0006014	-	ENSG00000132323;	Con_5Y2
hsa_circ_0004461	+	ENSG00000065802;	Con_5Y2
hsa_circ_0007159	+	ENSG00000168397;	Con_5Y2
hsa_circ_0053297	+	ENSG00000243943;ENSG0	Con_5Y2
hsa_circ_0053860	+	ENSG00000018699;	Con_5Y2
hsa_circ_0052471	+	ENSG00000171853;	Con_5Y2
hsa_circ_0054278	+	ENSG00000057935;	Con_5Y2
hsa_circ_0054338	-	ENSG00000138095;	Con_5Y2
hsa_circ_0054472	+	ENSG00000095002;	Con_5Y2
hsa_circ_0006587	-	ENSG00000115464;	Con_5Y2
hsa_circ_0054915	+	ENSG00000115504;	Con_5Y2
hsa_circ_0008804	+	ENSG00000115548;	Con_5Y2
hsa_circ_0013587	+	ENSG00000198799;	Con_5Y2
hsa_circ_0003247	-	ENSG00000116539;	Con_5Y2
hsa_circ_0014737	-	ENSG00000116604;	Con_5Y2
hsa_circ_0006507	-	ENSG00000116406;	Con_5Y2
hsa_circ_0016049	-	ENSG00000117139;	Con_5Y2

hsa_circ_0003527	-	ENSG00000117222;	Con_5Y2
hsa_circ_0000180	+	ENSG00000143476;	Con_5Y2
hsa_circ_0010530	-	ENSG00000090686;	Con_5Y2
hsa_circ_0016759	+	ENSG00000143740;	Con_5Y2
hsa_circ_0016991	-	ENSG00000135749;	Con_5Y2
hsa_circ_0002135	+	ENSG00000130695;	Con_5Y2
hsa_circ_0002868	+	ENSG00000130695;	Con_5Y2
hsa_circ_0008614	+	ENSG00000117682;	Con_5Y2
hsa_circ_0011153	+	ENSG00000180098;	Con_5Y2
hsa_circ_0011192	+	ENSG00000060656;	Con_5Y2
hsa_circ_0009057	+	ENSG00000121766;	Con_5Y2
hsa_circ_0003880	+	ENSG00000025800;	Con_5Y2
hsa_circ_0011537	+	ENSG00000146463;	Con_5Y2
hsa_circ_0003986	+	ENSG00000134698;	Con_5Y2
hsa_circ_0004093	+	ENSG00000116871;	Con_5Y2
hsa_circ_0003529	+	ENSG00000174574;	Con_5Y2
hsa_circ_0008844	+	ENSG00000168389;	Con_5Y2
hsa_circ_0012360	-	ENSG00000159658;	Con_5Y2
hsa_circ_0012494	+	ENSG00000157077;	Con_5Y2
hsa_circ_0007301	+	ENSG00000172456;	Con_5Y2
hsa_circ_0009531	-	ENSG00000162408;	Con_5Y2
hsa_circ_0013007	+	ENSG00000180488;	Con_5Y2
hsa_circ_0000088	+	ENSG00000180488;	Con_5Y2
hsa_circ_0009624	-	ENSG00000074800;	Con_5Y2
hsa_circ_0013213	-	ENSG00000137936;	Con_5Y2
hsa_circ_0004504	-	ENSG00000106771;	Con_5Y2
hsa_circ_0088062	-	ENSG00000106868;	Con_5Y2
hsa_circ_0088190	+	ENSG00000157693;	Con_5Y2
hsa_circ_0088296	-	ENSG00000095261;	Con_5Y2
hsa_circ_0088549	-	ENSG00000119414;	Con_5Y2
hsa_circ_0088708	-	ENSG00000160408;ENSG0	Con_5Y2
hsa_circ_0089196	+	ENSG00000130723;	Con_5Y2
hsa_circ_0089237	+	ENSG00000130714;	Con_5Y2
hsa_circ_0089252	-	ENSG00000107263;	Con_5Y2
hsa_circ_0089606	+	ENSG00000127191;	Con_5Y2
hsa_circ_0086226	-	ENSG00000080608;	Con_5Y2
hsa_circ_0086709	-	ENSG00000137073;	Con_5Y2
hsa_circ_0086739	-	ENSG00000137073;	Con_5Y2
hsa_circ_0086754	+	ENSG00000164978;	Con_5Y2
hsa_circ_0087009	-	ENSG00000159921;	Con_5Y2
hsa_circ_0087023	-	ENSG00000137075;	Con_5Y2
hsa_circ_0003486	+	ENSG00000147854;	Con_5Y2
hsa_circ_0087427	+	ENSG00000106723;	Con_5Y2
hsa_circ_0007416	-	ENSG00000127081;	Con_5Y2
hsa_circ_0087601	+	ENSG00000182150;	Con_5Y2
hsa_circ_0009164	+	ENSG00000164934;	Con_5Y2
hsa_circ_0007116	+	ENSG00000105339;	Con_5Y2
hsa_circ_0005256	-	ENSG00000171045;	Con_5Y2
hsa_circ_0005042	+	ENSG00000147459;	Con_5Y2
hsa_circ_0083772	+	ENSG00000168077;	Con_5Y2
hsa_circ_0006175	+	ENSG00000147421;	Con_5Y2
hsa_circ_0084304	-	ENSG00000253729;	Con_5Y2
novel_circ_0018630	+	ENSG00000147224;	Con_5Y2
novel_circ_0018730	-	ENSG00000181544;	Con_5Y2
novel_circ_0018761	+	ENSG00000169891;	Con_5Y2
novel_circ_0018796	+	ENSG00000101868;	Con_5Y2
novel_circ_0018861	-	ENSG00000086758;	Con_5Y2
novel_circ_0018867	+	ENSG00000227486;	Con_5Y2

novel_circ_0002803	+	ENSG00000134900;	Con_5Y2
novel_circ_0002828	+	ENSG00000068650;	Con_5Y2
novel_circ_0002869	-	ENSG00000132950;	Con_5Y2
novel_circ_0002893	+	ENSG00000032742;	Con_5Y2
novel_circ_0002970	-	ENSG00000120694;	Con_5Y2
novel_circ_0003013	-	ENSG00000120690;	Con_5Y2
novel_circ_0003051	+	ENSG00000179630;	Con_5Y2
novel_circ_0003064	-	ENSG00000123200;	Con_5Y2
novel_circ_0003096	+	ENSG00000102531;	Con_5Y2
novel_circ_0003151	-	ENSG00000139734;	Con_5Y2
novel_circ_0003172	-	ENSG00000136111;	Con_5Y2
novel_circ_0003233	+	ENSG00000102580;	Con_5Y2
novel_circ_0003269	+	ENSG00000125304;	Con_5Y2
novel_circ_0001821	+	ENSG00000120800;	Con_5Y2
novel_circ_0001858	+	ENSG00000198431;	Con_5Y2
novel_circ_0001903	+	ENSG00000076555;	Con_5Y2
novel_circ_0001905	+	ENSG00000151148;	Con_5Y2
novel_circ_0001917	+	ENSG00000122970;	Con_5Y2
novel_circ_0001936	-	ENSG00000196850;	Con_5Y2
novel_circ_0002025	-	ENSG00000123066;	Con_5Y2
novel_circ_0002025	-	ENSG00000123066;	Con_5Y2
novel_circ_0002029	-	ENSG00000123066;	Con_5Y2
novel_circ_0002073	+	ENSG00000175970;	Con_5Y2
novel_circ_0002090	+	ENSG00000256546;	Con_5Y2
novel_circ_0002216	+	ENSG00000183495;	Con_5Y2
novel_circ_0002239	-	ENSG00000273079;	Con_5Y2
novel_circ_0002276	-	ENSG00000111731;	Con_5Y2
novel_circ_0002281	-	ENSG00000133703;	Con_5Y2
novel_circ_0002310	+	ENSG00000111203;	Con_5Y2
novel_circ_0002380	-	ENSG00000073614;	Con_5Y2
novel_circ_0002456	+	ENSG00000123352;	Con_5Y2
novel_circ_0002526	+	ENSG00000111540;	Con_5Y2
novel_circ_0002643	+	ENSG00000127328;	Con_5Y2
novel_circ_0002657	-	ENSG00000133858;	Con_5Y2
novel_circ_0002726	-	ENSG00000198707;	Con_5Y2
novel_circ_0001067	+	ENSG00000149311;	Con_5Y2
novel_circ_0001098	+	ENSG00000150768;	Con_5Y2
novel_circ_0001121	+	ENSG00000167257;	Con_5Y2
novel_circ_0001160	+	ENSG00000196914;	Con_5Y2
novel_circ_0001161	+	ENSG00000196914;	Con_5Y2
novel_circ_0001167	+	ENSG00000149403;	Con_5Y2
novel_circ_0001175	+	ENSG00000285509;ENSG0	Con_5Y2
novel_circ_0001240	-	ENSG00000011405;	Con_5Y2
novel_circ_0001244	-	ENSG00000011405;	Con_5Y2
novel_circ_0001246	+	ENSG00000070081;	Con_5Y2
novel_circ_0001271	+	ENSG00000151117;	Con_5Y2
novel_circ_0001291	+	ENSG00000053918;	Con_5Y2
novel_circ_0001314	+	ENSG00000149100;	Con_5Y2
novel_circ_0001323	+	ENSG00000176148;	Con_5Y2
novel_circ_0001326	-	ENSG00000176102;	Con_5Y2
novel_circ_0001373	-	ENSG00000110713;	Con_5Y2
novel_circ_0001380	-	ENSG00000110713;	Con_5Y2
novel_circ_0001411	+	ENSG00000151348;	Con_5Y2
novel_circ_0001425	-	ENSG00000175216;	Con_5Y2
novel_circ_0001442	-	ENSG00000109920;	Con_5Y2
novel_circ_0001475	-	ENSG00000167992;	Con_5Y2
novel_circ_0001518	+	ENSG00000014216;	Con_5Y2
novel_circ_0001535	+	ENSG00000173933;ENSG0	Con_5Y2

novel_circ_0001574	+	ENSG00000110075;	Con_5Y2
novel_circ_0001590	+	ENSG00000162341;	Con_5Y2
novel_circ_0001695	+	ENSG00000165494;	Con_5Y2
novel_circ_0001735	-	ENSG00000166436;	Con_5Y2
novel_circ_0001765	+	ENSG00000182919;ENSG0	Con_5Y2
novel_circ_0000093	+	ENSG00000148700;	Con_5Y2
novel_circ_0000144	-	ENSG00000099204;	Con_5Y2
novel_circ_0000163	+	ENSG00000107518;	Con_5Y2
novel_circ_0000175	-	ENSG00000107560;	Con_5Y2
novel_circ_0000189	+	ENSG00000107581;	Con_5Y2
novel_circ_0000192	-	ENSG00000107581;	Con_5Y2
novel_circ_0000214	-	ENSG00000197771;	Con_5Y2
novel_circ_0000351	+	ENSG00000065328;	Con_5Y2
novel_circ_0000379	-	ENSG00000152464;ENSG0	Con_5Y2
novel_circ_0000381	-	ENSG00000152465;	Con_5Y2
novel_circ_0000419	-	ENSG00000136770;	Con_5Y2
novel_circ_0000438	+	ENSG00000148459;	Con_5Y2
novel_circ_0000534	-	ENSG00000148498;	Con_5Y2
novel_circ_0000540	-	ENSG00000148498;	Con_5Y2
novel_circ_0000656	-	ENSG00000108091;	Con_5Y2
novel_circ_0000689	+	ENSG00000096746;	Con_5Y2
novel_circ_0000693	-	ENSG00000204130;	Con_5Y2
novel_circ_0000698	-	ENSG00000204130;	Con_5Y2
novel_circ_0000713	+	ENSG00000138336;	Con_5Y2
novel_circ_0000740	+	ENSG00000165732;	Con_5Y2
novel_circ_0000753	-	ENSG00000079332;	Con_5Y2
novel_circ_0000780	-	ENSG00000156042;	Con_5Y2
novel_circ_0000864	-	ENSG00000138138;	Con_5Y2
novel_circ_0000908	-	ENSG00000119912;	Con_5Y2
novel_circ_0000913	+	ENSG00000138160;	Con_5Y2
novel_circ_0000930	-	ENSG00000148690;	Con_5Y2
novel_circ_0000937	+	ENSG00000108239;	Con_5Y2
novel_circ_0000962	-	ENSG00000077147;	Con_5Y2
novel_circ_0000987	+	ENSG00000023839;	Con_5Y2
novel_circ_0005339	-	ENSG00000108599;	Con_5Y2
novel_circ_0005369	-	ENSG00000070366;	Con_5Y2
novel_circ_0005390	-	ENSG00000004142;	Con_5Y2
novel_circ_0005480	+	ENSG00000006125;	Con_5Y2
novel_circ_0005506	-	ENSG00000278540;	Con_5Y2
novel_circ_0005554	+	ENSG00000094804;	Con_5Y2
novel_circ_0005555	+	n/a	Con_5Y2
novel_circ_0005563	-	ENSG00000131747;	Con_5Y2
novel_circ_0005586	-	ENSG00000168610;	Con_5Y2
novel_circ_0005607	-	ENSG00000132388;	Con_5Y2
novel_circ_0005632	-	ENSG00000182963;	Con_5Y2
novel_circ_0005707	-	ENSG00000198740;	Con_5Y2
novel_circ_0005787	-	ENSG00000121058;	Con_5Y2
novel_circ_0005812	-	ENSG00000108395;	Con_5Y2
novel_circ_0005881	-	ENSG00000136492;	Con_5Y2
novel_circ_0005935	-	ENSG00000136478;	Con_5Y2
novel_circ_0005982	-	ENSG00000154240;	Con_5Y2
novel_circ_0006012	+	ENSG00000141337;	Con_5Y2
novel_circ_0006016	+	ENSG00000108946;	Con_5Y2
novel_circ_0006021	-	ENSG00000227036;	Con_5Y2
novel_circ_0006030	-	ENSG00000133195;	Con_5Y2
novel_circ_0006032	-	ENSG00000133195;	Con_5Y2
novel_circ_0006061	-	ENSG00000167699;	Con_5Y2
novel_circ_0006063	-	ENSG00000167881;	Con_5Y2

novel_circ_0006070	-	ENSG00000129667;	Con_5Y2
novel_circ_0006111	+	ENSG00000173821;	Con_5Y2
novel_circ_0006167	-	ENSG00000176155;	Con_5Y2
novel_circ_0004625	-	ENSG00000059145;	Con_5Y2
novel_circ_0004697	+	ENSG00000167186;	Con_5Y2
novel_circ_0004701	+	ENSG00000103534;	Con_5Y2
novel_circ_0004706	-	ENSG00000103550;	Con_5Y2
novel_circ_0004716	+	ENSG00000103197;	Con_5Y2
novel_circ_0004762	+	ENSG00000090905;	Con_5Y2
novel_circ_0004800	-	n/a	Con_5Y2
novel_circ_0004830	+	ENSG00000103351;	Con_5Y2
novel_circ_0004865	-	ENSG00000069329;	Con_5Y2
novel_circ_0004919	+	ENSG00000103479;	Con_5Y2
novel_circ_0004929	+	ENSG00000140718;	Con_5Y2
novel_circ_0005004	+	ENSG00000141076;	Con_5Y2
novel_circ_0005012	-	ENSG00000132604;	Con_5Y2
novel_circ_0005022	-	ENSG00000127585;	Con_5Y2
novel_circ_0005056	+	ENSG00000189091;	Con_5Y2
novel_circ_0005057	+	ENSG00000189091;	Con_5Y2
novel_circ_0005084	-	ENSG00000090863;	Con_5Y2
novel_circ_0005136	+	ENSG00000103150;	Con_5Y2
novel_circ_0003937	-	ENSG00000140470;	Con_5Y2
novel_circ_0003950	-	ENSG00000140471;	Con_5Y2
novel_circ_0003990	-	ENSG00000128731;	Con_5Y2
novel_circ_0004019	-	ENSG00000021776;	Con_5Y2
novel_circ_0004051	+	ENSG00000137812;	Con_5Y2
novel_circ_0004129	-	ENSG00000104133;	Con_5Y2
novel_circ_0004144	-	ENSG00000166200;	Con_5Y2
novel_circ_0004173	-	ENSG00000138600;	Con_5Y2
novel_circ_0004193	-	ENSG00000047346;	Con_5Y2
novel_circ_0004220	-	ENSG00000140299;	Con_5Y2
novel_circ_0004265	-	ENSG00000169118;ENSG0	Con_5Y2
novel_circ_0004283	-	ENSG00000074603;	Con_5Y2
novel_circ_0004473	+	ENSG00000169684;	Con_5Y2
novel_circ_0004562	+	ENSG00000173575;	Con_5Y2
novel_circ_0004564	+	ENSG00000173575;	Con_5Y2
novel_circ_0004573	+	ENSG00000140563;	Con_5Y2
novel_circ_0004582	-	ENSG00000251209;	Con_5Y2
novel_circ_0004586	-	ENSG00000103852;	Con_5Y2
novel_circ_0003322	+	ENSG00000197102;	Con_5Y2
novel_circ_0003332	+	ENSG00000196663;	Con_5Y2
novel_circ_0003381	+	ENSG00000129484;	Con_5Y2
novel_circ_0003382	+	ENSG00000129484;	Con_5Y2
novel_circ_0003406	-	ENSG00000092036;	Con_5Y2
novel_circ_0003449	-	ENSG00000092148;	Con_5Y2
novel_circ_0003536	+	ENSG00000187790;	Con_5Y2
novel_circ_0003538	-	ENSG00000129534;	Con_5Y2
novel_circ_0003539	-	ENSG00000129534;	Con_5Y2
novel_circ_0003540	-	ENSG00000129534;	Con_5Y2
novel_circ_0003567	-	ENSG00000100485;	Con_5Y2
novel_circ_0003590	-	ENSG00000100503;	Con_5Y2
novel_circ_0003683	-	n/a	Con_5Y2
novel_circ_0003695	-	ENSG00000125952;	Con_5Y2
novel_circ_0003718	+	ENSG00000182185;	Con_5Y2
novel_circ_0003724	+	ENSG00000100626;	Con_5Y2
novel_circ_0003864	-	ENSG00000165914;	Con_5Y2
novel_circ_0003882	-	ENSG00000100815;	Con_5Y2
novel_circ_0006725	-	ENSG00000123136;	Con_5Y2

novel_circ_0006736	-	ENSG00000171903;	Con_5Y2
novel_circ_0006757	+	ENSG00000099331;	Con_5Y2
novel_circ_0006786	+	ENSG00000167491;	Con_5Y2
novel_circ_0006844	-	ENSG00000105186;	Con_5Y2
novel_circ_0006854	-	ENSG00000121289;	Con_5Y2
novel_circ_0006912	+	ENSG00000105738;	Con_5Y2
novel_circ_0006938	-	ENSG00000123815;	Con_5Y2
novel_circ_0006943	+	ENSG00000167600;	Con_5Y2
novel_circ_0007095	-	ENSG00000080031;	Con_5Y2
novel_circ_0007101	-	ENSG00000130254;	Con_5Y2
novel_circ_0007110	+	ENSG00000160633;	Con_5Y2
novel_circ_0006232	+	n/a	Con_5Y2
novel_circ_0006241	-	ENSG00000134278;	Con_5Y2
novel_circ_0006310	-	ENSG00000079134;	Con_5Y2
novel_circ_0006340	+	ENSG00000101773;	Con_5Y2
novel_circ_0006381	+	ENSG00000101596;	Con_5Y2
novel_circ_0006428	-	ENSG00000152234;	Con_5Y2
novel_circ_0006453	-	ENSG00000141627;	Con_5Y2
novel_circ_0006473	-	ENSG00000167306;	Con_5Y2
novel_circ_0006495	+	ENSG00000101751;	Con_5Y2
novel_circ_0006517	-	ENSG00000066926;	Con_5Y2
novel_circ_0009741	+	ENSG00000100014;ENSG0	Con_5Y2
novel_circ_0009749	+	ENSG00000100028;ENSG0	Con_5Y2
novel_circ_0009837	-	ENSG00000233080;	Con_5Y2
novel_circ_0009892	+	ENSG00000196236;	Con_5Y2
novel_circ_0009907	+	ENSG00000100393;	Con_5Y2
novel_circ_0009945	+	ENSG00000248405;ENSG0	Con_5Y2
novel_circ_0009974	-	ENSG00000075275;	Con_5Y2
novel_circ_0009086	+	ENSG00000149474;	Con_5Y2
novel_circ_0009202	+	ENSG00000126001;	Con_5Y2
novel_circ_0009262	+	ENSG00000101413;	Con_5Y2
novel_circ_0009330	-	ENSG00000101040;	Con_5Y2
novel_circ_0009334	-	ENSG00000101040;	Con_5Y2
novel_circ_0009377	-	ENSG00000124201;	Con_5Y2
novel_circ_0009419	-	ENSG00000101144;	Con_5Y2
novel_circ_0009434	+	ENSG00000286235;ENSG0	Con_5Y2
novel_circ_0009536	-	ENSG00000142207;	Con_5Y2
novel_circ_0009578	+	ENSG00000159259;	Con_5Y2
novel_circ_0015823	+	ENSG00000272752;	Con_5Y2
novel_circ_0015881	-	ENSG00000135250;	Con_5Y2
novel_circ_0015937	-	ENSG00000184903;	Con_5Y2
novel_circ_0016032	+	ENSG00000155561;	Con_5Y2
novel_circ_0016034	+	ENSG00000155561;	Con_5Y2
novel_circ_0016170	+	ENSG00000009335;	Con_5Y2
novel_circ_0016308	-	ENSG00000070882;	Con_5Y2
novel_circ_0016310	-	ENSG00000070882;	Con_5Y2
novel_circ_0016399	+	ENSG00000065883;	Con_5Y2
novel_circ_0016628	+	ENSG00000135205;	Con_5Y2
novel_circ_0016640	+	ENSG00000127947;	Con_5Y2
novel_circ_0016648	+	ENSG00000187257;	Con_5Y2
novel_circ_0016653	+	ENSG00000006576;	Con_5Y2
novel_circ_0016700	+	ENSG00000105792;	Con_5Y2
novel_circ_0016717	+	ENSG00000127914;	Con_5Y2
novel_circ_0016720	+	ENSG00000127914;	Con_5Y2
novel_circ_0016781	-	ENSG00000198742;	Con_5Y2
novel_circ_0015051	+	ENSG00000111907;	Con_5Y2
novel_circ_0015151	+	ENSG00000112419;	Con_5Y2
novel_circ_0015152	+	ENSG00000135521;	Con_5Y2

novel_circ_0015198	+	ENSG00000120254;	Con_5Y2
novel_circ_0015251	+	ENSG00000175048;	Con_5Y2
novel_circ_0015294	-	ENSG00000092820;	Con_5Y2
novel_circ_0015327	+	ENSG00000130396;	Con_5Y2
novel_circ_0015335	+	ENSG00000130396;	Con_5Y2
novel_circ_0015402	+	ENSG00000250903;	Con_5Y2
novel_circ_0015437	-	ENSG00000204463;	Con_5Y2
novel_circ_0015447	+	ENSG00000258388;ENSG0	Con_5Y2
novel_circ_0015449	-	ENSG00000137266;	Con_5Y2
novel_circ_0015450	-	ENSG00000137266;	Con_5Y2
novel_circ_0015477	-	ENSG00000096063;	Con_5Y2
novel_circ_0015492	-	ENSG00000112079;	Con_5Y2
novel_circ_0015499	+	ENSG00000156639;	Con_5Y2
novel_circ_0015602	+	ENSG00000112200;	Con_5Y2
novel_circ_0015636	+	ENSG00000082269;	Con_5Y2
novel_circ_0015761	+	ENSG00000146281;	Con_5Y2
novel_circ_0014002	-	ENSG00000186952;	Con_5Y2
novel_circ_0014068	+	ENSG00000151292;	Con_5Y2
novel_circ_0014185	-	ENSG00000094880;	Con_5Y2
novel_circ_0014233	+	ENSG00000254996;ENSG0	Con_5Y2
novel_circ_0014291	+	ENSG00000091009;ENSG0	Con_5Y2
novel_circ_0014298	+	ENSG00000154124;	Con_5Y2
novel_circ_0014334	+	ENSG00000155506;	Con_5Y2
novel_circ_0014336	+	ENSG00000155508;	Con_5Y2
novel_circ_0014370	-	ENSG00000145555;	Con_5Y2
novel_circ_0014553	+	ENSG00000063438;ENSG0	Con_5Y2
novel_circ_0014687	+	ENSG00000112851;	Con_5Y2
novel_circ_0014810	-	ENSG00000164300;	Con_5Y2
novel_circ_0013088	-	ENSG00000109323;	Con_5Y2
novel_circ_0013117	-	ENSG00000138801;	Con_5Y2
novel_circ_0013201	+	ENSG00000164074;	Con_5Y2
novel_circ_0013231	-	n/a	Con_5Y2
novel_circ_0013252	+	ENSG00000164134;	Con_5Y2
novel_circ_0013437	-	ENSG00000137601;	Con_5Y2
novel_circ_0013550	-	ENSG00000091490;	Con_5Y2
novel_circ_0013592	+	ENSG00000169851;	Con_5Y2
novel_circ_0013758	-	ENSG00000128059;	Con_5Y2
novel_circ_0013761	+	ENSG00000174780;	Con_5Y2
novel_circ_0013770	+	ENSG00000248049;	Con_5Y2
novel_circ_0013773	+	ENSG00000170871;	Con_5Y2
novel_circ_0013879	-	ENSG00000163312;	Con_5Y2
novel_circ_0013925	+	ENSG00000163104;	Con_5Y2
novel_circ_0011770	+	ENSG00000114354;	Con_5Y2
novel_circ_0011862	-	n/a	Con_5Y2
novel_circ_0011884	-	ENSG00000051341;	Con_5Y2
novel_circ_0011898	-	ENSG00000114030;	Con_5Y2
novel_circ_0011966	-	ENSG00000175792;	Con_5Y2
novel_circ_0012018	-	ENSG00000163781;	Con_5Y2
novel_circ_0012024	-	ENSG00000163785;	Con_5Y2
novel_circ_0012034	-	ENSG00000118007;	Con_5Y2
novel_circ_0012122	-	ENSG00000071794;	Con_5Y2
novel_circ_0012123	-	ENSG00000071794;	Con_5Y2
novel_circ_0012124	-	ENSG00000071794;	Con_5Y2
novel_circ_0012149	+	ENSG00000144895;	Con_5Y2
novel_circ_0012225	-	n/a	Con_5Y2
novel_circ_0012251	-	ENSG00000173889;	Con_5Y2
novel_circ_0012320	+	ENSG00000058056;	Con_5Y2
novel_circ_0012330	+	ENSG00000058063;	Con_5Y2

novel_circ_0012337	-	ENSG00000053524;	Con_5Y2
novel_circ_0012364	+	ENSG00000114867;	Con_5Y2
novel_circ_0012383	+	ENSG00000073803;	Con_5Y2
novel_circ_0012420	-	ENSG00000133657;	Con_5Y2
novel_circ_0012436	-	ENSG00000061938;	Con_5Y2
novel_circ_0012446	+	ENSG00000273331;ENSG0	Con_5Y2
novel_circ_0012534	-	ENSG00000033867;	Con_5Y2
novel_circ_0012554	+	ENSG00000163513;	Con_5Y2
novel_circ_0012576	+	ENSG00000152642;	Con_5Y2
novel_circ_0012587	+	ENSG00000153551;	Con_5Y2
novel_circ_0012687	+	ENSG00000114857;	Con_5Y2
novel_circ_0012774	-	ENSG00000173473;	Con_5Y2
novel_circ_0012777	-	ENSG00000173473;	Con_5Y2
novel_circ_0012841	+	ENSG00000114353;	Con_5Y2
novel_circ_0012975	-	ENSG00000151276;	Con_5Y2
novel_circ_0013025	-	ENSG00000196220;ENSG0	Con_5Y2
novel_circ_0013046	+	ENSG00000241553;	Con_5Y2
novel_circ_0010015	-	ENSG00000204634;	Con_5Y2
novel_circ_0010088	-	ENSG00000153107;	Con_5Y2
novel_circ_0010093	+	ENSG00000144152;	Con_5Y2
novel_circ_0010120	+	n/a	Con_5Y2
novel_circ_0010121	+	ENSG00000088179;	Con_5Y2
novel_circ_0010258	+	ENSG00000080345;	Con_5Y2
novel_circ_0010270	+	ENSG00000080345;	Con_5Y2
novel_circ_0010357	+	ENSG00000136560;	Con_5Y2
novel_circ_0010432	+	ENSG00000115827;	Con_5Y2
novel_circ_0010493	+	ENSG00000138434;	Con_5Y2
novel_circ_0010502	-	ENSG00000061676;	Con_5Y2
novel_circ_0010508	-	ENSG00000061676;	Con_5Y2
novel_circ_0010610	-	ENSG00000115942;	Con_5Y2
novel_circ_0010678	+	ENSG00000138443;	Con_5Y2
novel_circ_0010795	+	ENSG00000239498;	Con_5Y2
novel_circ_0010826	-	ENSG00000036257;	Con_5Y2
novel_circ_0010876	+	ENSG00000135931;	Con_5Y2
novel_circ_0010892	+	ENSG00000204120;	Con_5Y2
novel_circ_0010913	+	ENSG00000119771;	Con_5Y2
novel_circ_0010966	-	ENSG00000122085;	Con_5Y2
novel_circ_0011031	+	ENSG00000115163;	Con_5Y2
novel_circ_0011055	-	ENSG00000171103;	Con_5Y2
novel_circ_0011147	+	ENSG00000115760;	Con_5Y2
novel_circ_0011184	-	ENSG00000115808;	Con_5Y2
novel_circ_0011191	-	ENSG00000055332;	Con_5Y2
novel_circ_0011206	+	ENSG00000232973;	Con_5Y2
novel_circ_0011227	-	ENSG00000011566;	Con_5Y2
novel_circ_0011400	+	ENSG00000162929;	Con_5Y2
novel_circ_0011412	+	ENSG00000173209;	Con_5Y2
novel_circ_0011425	-	ENSG00000115464;	Con_5Y2
novel_circ_0011453	-	ENSG00000082898;	Con_5Y2
novel_circ_0011474	-	ENSG00000082898;	Con_5Y2
novel_circ_0011564	-	ENSG00000115998;	Con_5Y2
novel_circ_0011650	+	ENSG00000168883;	Con_5Y2
novel_circ_0007190	+	ENSG00000130939;	Con_5Y2
novel_circ_0007461	-	ENSG00000116580;	Con_5Y2
novel_circ_0007462	-	ENSG00000116580;	Con_5Y2
novel_circ_0007466	-	ENSG00000116580;	Con_5Y2
novel_circ_0007486	+	ENSG00000197312;	Con_5Y2
novel_circ_0007531	-	ENSG00000248333;	Con_5Y2
novel_circ_0007535	-	ENSG00000143183;	Con_5Y2

novel_circ_0007586	+	ENSG00000117523;	Con_5Y2
novel_circ_0007619	-	ENSG00000135870;	Con_5Y2
novel_circ_0007663	+	ENSG00000074964;	Con_5Y2
novel_circ_0007669	+	ENSG00000186283;	Con_5Y2
novel_circ_0007802	-	ENSG00000118193;	Con_5Y2
novel_circ_0007805	-	ENSG00000118193;	Con_5Y2
novel_circ_0007920	+	ENSG00000162769;	Con_5Y2
novel_circ_0007956	-	ENSG00000090686;	Con_5Y2
novel_circ_0008000	-	ENSG00000162923;	Con_5Y2
novel_circ_0008095	-	ENSG00000069248;	Con_5Y2
novel_circ_0008177	-	ENSG00000054267;	Con_5Y2
novel_circ_0008199	+	ENSG00000116984;	Con_5Y2
novel_circ_0008413	+	ENSG00000160094;	Con_5Y2
novel_circ_0008464	-	ENSG00000163877;	Con_5Y2
novel_circ_0008653	-	ENSG00000134744;	Con_5Y2
novel_circ_0008721	+	ENSG00000134709;	Con_5Y2
novel_circ_0008748	-	ENSG00000116237;	Con_5Y2
novel_circ_0008758	-	ENSG00000116641;	Con_5Y2
novel_circ_0008797	+	ENSG00000198160;	Con_5Y2
novel_circ_0008848	-	ENSG00000077254;	Con_5Y2
novel_circ_0008994	+	ENSG00000122483;	Con_5Y2
novel_circ_0017784	+	ENSG00000148143;	Con_5Y2
novel_circ_0017792	+	ENSG00000119318;	Con_5Y2
novel_circ_0017855	-	ENSG00000119314;	Con_5Y2
novel_circ_0017911	+	ENSG00000011454;	Con_5Y2
novel_circ_0017926	-	ENSG00000165209;	Con_5Y2
novel_circ_0017947	-	ENSG00000173611;	Con_5Y2
novel_circ_0018002	+	ENSG00000095319;	Con_5Y2
novel_circ_0018025	+	ENSG00000130723;	Con_5Y2
novel_circ_0018043	-	ENSG00000107290;	Con_5Y2
novel_circ_0018053	-	ENSG00000148300;	Con_5Y2
novel_circ_0018064	+	ENSG00000186350;	Con_5Y2
novel_circ_0018087	-	ENSG00000148396;	Con_5Y2
novel_circ_0018090	-	ENSG00000148400;	Con_5Y2
novel_circ_0018104	+	ENSG00000181090;	Con_5Y2
novel_circ_0018199	+	ENSG00000096872;	Con_5Y2
novel_circ_0018305	+	ENSG00000165304;	Con_5Y2
novel_circ_0018343	+	ENSG00000120158;	Con_5Y2
novel_circ_0018365	+	ENSG00000165072;	Con_5Y2
novel_circ_0018368	+	ENSG00000165072;	Con_5Y2
novel_circ_0018369	+	ENSG00000198887;	Con_5Y2
novel_circ_0018406	+	ENSG00000172159;	Con_5Y2
novel_circ_0018496	+	ENSG00000165238;	Con_5Y2
novel_circ_0018504	+	ENSG00000048828;	Con_5Y2
novel_circ_0018545	+	ENSG00000182150;	Con_5Y2
novel_circ_0018583	+	ENSG00000197816;ENSG0	Con_5Y2
novel_circ_0016800	-	ENSG00000034677;	Con_5Y2
novel_circ_0016921	-	ENSG00000136982;	Con_5Y2
novel_circ_0017036	+	ENSG00000129292;	Con_5Y2
novel_circ_0017071	-	ENSG00000123908;	Con_5Y2
novel_circ_0017132	-	ENSG00000171045;	Con_5Y2
novel_circ_0017144	+	ENSG00000179832;	Con_5Y2
novel_circ_0017205	+	ENSG00000104728;ENSG0	Con_5Y2
novel_circ_0017215	+	ENSG00000104611;	Con_5Y2
novel_circ_0017245	+	ENSG00000221914;	Con_5Y2
novel_circ_0017266	-	ENSG00000104299;	Con_5Y2
novel_circ_0017277	-	ENSG00000251191;	Con_5Y2
novel_circ_0017313	-	ENSG00000104221;	Con_5Y2

novel_circ_0017435	-	ENSG00000023287;	Con_5Y2
novel_circ_0017457	-	n/a	Con_5Y2
novel_circ_0017490	+	ENSG00000066855;	Con_5Y2
novel_circ_0017521	+	ENSG00000104218;	Con_5Y2
novel_circ_0017557	+	ENSG00000121039;	Con_5Y2
novel_circ_0017565	+	ENSG00000104381;	Con_5Y2
novel_circ_0017599	+	ENSG00000085719;	Con_5Y2
novel_circ_0017658	-	ENSG00000265817;ENSG0	Con_5Y2
novel_circ_0017659	-	ENSG00000265817;ENSG0	Con_5Y2
novel_circ_0017672	+	ENSG00000104413;	Con_5Y2
novel_circ_0017684	+	ENSG00000156170;	Con_5Y2
hsa_circ_0000503	+	ENSG00000102606;	Con_5Y2,Con_5Y3
hsa_circ_0003852	-	ENSG00000150456;	Con_5Y2,Con_5Y3
hsa_circ_0004627	+	ENSG00000120800;	Con_5Y2,Con_5Y3
hsa_circ_0005824	-	ENSG00000064115;	Con_5Y2,Con_5Y3
hsa_circ_0024470	-	ENSG00000118096;	Con_5Y2,Con_5Y3
hsa_circ_0002294	-	ENSG00000110367;	Con_5Y2,Con_5Y3
hsa_circ_0002332	+	ENSG00000149554;	Con_5Y2,Con_5Y3
hsa_circ_0002038	+	ENSG00000110075;	Con_5Y2,Con_5Y3
hsa_circ_0021155	-	ENSG00000166471;	Con_5Y2,Con_5Y3
hsa_circ_0008115	+	ENSG00000148459;	Con_5Y2,Con_5Y3
hsa_circ_0017476	+	ENSG00000067057;	Con_5Y2,Con_5Y3
hsa_circ_0018600	+	ENSG00000156502;	Con_5Y2,Con_5Y3
hsa_circ_0000247	+	ENSG00000156026;	Con_5Y2,Con_5Y3
hsa_circ_0005392	-	ENSG00000138119;	Con_5Y2,Con_5Y3
hsa_circ_0006371	+	ENSG00000196233;	Con_5Y2,Con_5Y3
hsa_circ_0042253	-	ENSG00000141030;	Con_5Y2,Con_5Y3
hsa_circ_0006964	-	ENSG00000131747;	Con_5Y2,Con_5Y3
hsa_circ_0004006	+	ENSG00000170921;	Con_5Y2,Con_5Y3
hsa_circ_0006761	+	ENSG00000173821;	Con_5Y2,Con_5Y3
hsa_circ_0046598	-	ENSG00000175711;	Con_5Y2,Con_5Y3
hsa_circ_0000676	+	ENSG00000103222;	Con_5Y2,Con_5Y3
hsa_circ_0038872	+	ENSG00000196296;	Con_5Y2,Con_5Y3
hsa_circ_0002970	+	ENSG00000103888;	Con_5Y2,Con_5Y3
hsa_circ_0004200	-	ENSG00000247809;	Con_5Y2,Con_5Y3
hsa_circ_0005670	+	ENSG00000151413;	Con_5Y2,Con_5Y3
hsa_circ_0007695	-	ENSG00000100485;	Con_5Y2,Con_5Y3
hsa_circ_0049370	+	ENSG00000127616;	Con_5Y2,Con_5Y3
hsa_circ_0000881	+	ENSG00000160633;	Con_5Y2,Con_5Y3
hsa_circ_0049024	-	ENSG00000104980;	Con_5Y2,Con_5Y3
hsa_circ_0062802	-	ENSG00000100325;	Con_5Y2,Con_5Y3
hsa_circ_0060068	-	ENSG00000101019;	Con_5Y2,Con_5Y3
hsa_circ_0060300	+	ENSG00000197122;	Con_5Y2,Con_5Y3
hsa_circ_0007207	-	ENSG00000156304;	Con_5Y2,Con_5Y3
hsa_circ_0061687	-	ENSG00000157538;	Con_5Y2,Con_5Y3
hsa_circ_0005419	+	ENSG00000228409;	Con_5Y2,Con_5Y3
hsa_circ_0075551	+	ENSG00000124782;	Con_5Y2,Con_5Y3
hsa_circ_0077101	+	ENSG00000196586;	Con_5Y2,Con_5Y3
hsa_circ_0071224	-	ENSG00000198589;	Con_5Y2,Con_5Y3
hsa_circ_0069420	+	ENSG00000065882;	Con_5Y2,Con_5Y3
hsa_circ_0069649	-	ENSG00000170448;	Con_5Y2,Con_5Y3
hsa_circ_0070040	-	ENSG00000138750;	Con_5Y2,Con_5Y3
hsa_circ_0007358	-	ENSG00000074416;	Con_5Y2,Con_5Y3
hsa_circ_0007818	-	ENSG00000240303;ENSG0	Con_5Y2,Con_5Y3
hsa_circ_0064460	+	ENSG00000177463;	Con_5Y2,Con_5Y3
hsa_circ_0002622	-	ENSG00000173889;	Con_5Y2,Con_5Y3
hsa_circ_0003975	+	ENSG00000114346;	Con_5Y2,Con_5Y3
hsa_circ_0008997	-	ENSG00000173473;	Con_5Y2,Con_5Y3

hsa_circ_0066535	-	ENSG00000285708;ENSG0	Con_5Y2,Con_5Y3
hsa_circ_0056469	+	ENSG00000136002;	Con_5Y2,Con_5Y3
hsa_circ_0056586	+	ENSG00000048991;	Con_5Y2,Con_5Y3
hsa_circ_0058055	-	ENSG00000138376;	Con_5Y2,Con_5Y3
hsa_circ_0053083	+	ENSG00000138029;	Con_5Y2,Con_5Y3
hsa_circ_0008559	+	ENSG00000171132;	Con_5Y2,Con_5Y3
hsa_circ_0054547	-	ENSG00000068878;	Con_5Y2,Con_5Y3
hsa_circ_0014132	+	ENSG00000143398;	Con_5Y2,Con_5Y3
hsa_circ_0000162	+	ENSG00000116191;	Con_5Y2,Con_5Y3
hsa_circ_0015928	+	ENSG00000198700;	Con_5Y2,Con_5Y3
hsa_circ_0011173	+	ENSG00000159023;	Con_5Y2,Con_5Y3
hsa_circ_0012152	+	ENSG00000187147;	Con_5Y2,Con_5Y3
hsa_circ_0012779	+	ENSG00000132849;	Con_5Y2,Con_5Y3
hsa_circ_0003697	-	ENSG00000136813;	Con_5Y2,Con_5Y3
hsa_circ_0085311	+	ENSG00000164830;	Con_5Y2,Con_5Y3
hsa_circ_0003183	+	ENSG00000104388;	Con_5Y2,Con_5Y3
novel_circ_0018866	+	n/a	Con_5Y2,Con_5Y3
novel_circ_0002113	+	ENSG00000184445;	Con_5Y2,Con_5Y3
novel_circ_0002735	-	ENSG00000049130;	Con_5Y2,Con_5Y3
novel_circ_0002746	-	ENSG00000139318;	Con_5Y2,Con_5Y3
novel_circ_0002777	+	ENSG00000120802;	Con_5Y2,Con_5Y3
novel_circ_0001042	-	ENSG00000110660;	Con_5Y2,Con_5Y3
novel_circ_0001105	-	ENSG00000048028;	Con_5Y2,Con_5Y3
novel_circ_0001119	+	ENSG00000167257;	Con_5Y2,Con_5Y3
novel_circ_0001512	+	ENSG00000168439;	Con_5Y2,Con_5Y3
novel_circ_0005975	-	ENSG00000154240;	Con_5Y2,Con_5Y3
novel_circ_0006015	+	ENSG00000108946;	Con_5Y2,Con_5Y3
novel_circ_0006044	+	ENSG00000170190;	Con_5Y2,Con_5Y3
novel_circ_0004703	+	ENSG00000103534;	Con_5Y2,Con_5Y3
novel_circ_0005009	+	ENSG00000168807;	Con_5Y2,Con_5Y3
novel_circ_0004058	-	ENSG00000128908;	Con_5Y2,Con_5Y3
novel_circ_0006906	-	ENSG00000245680;ENSG0	Con_5Y2,Con_5Y3
novel_circ_0006400	-	ENSG00000153339;	Con_5Y2,Con_5Y3
novel_circ_0009349	+	ENSG00000124198;	Con_5Y2,Con_5Y3
novel_circ_0009443	-	ENSG00000130699;	Con_5Y2,Con_5Y3
novel_circ_0009682	+	ENSG00000160299;	Con_5Y2,Con_5Y3
novel_circ_0015737	-	ENSG00000135316;	Con_5Y2,Con_5Y3
novel_circ_0013150	-	ENSG00000145349;	Con_5Y2,Con_5Y3
novel_circ_0013661	-	ENSG00000163697;	Con_5Y2,Con_5Y3
novel_circ_0013870	-	ENSG00000189308;	Con_5Y2,Con_5Y3
novel_circ_0012915	-	ENSG00000174839;	Con_5Y2,Con_5Y3
novel_circ_0010188	-	ENSG00000152102;	Con_5Y2,Con_5Y3
novel_circ_0011622	-	ENSG00000114993;	Con_5Y2,Con_5Y3
novel_circ_0011714	+	ENSG00000121152;	Con_5Y2,Con_5Y3
novel_circ_0011758	-	ENSG00000135945;	Con_5Y2,Con_5Y3
novel_circ_0007359	+	ENSG00000163125;	Con_5Y2,Con_5Y3
novel_circ_0007384	+	ENSG00000159352;	Con_5Y2,Con_5Y3
novel_circ_0007419	+	ENSG00000116138;	Con_5Y2,Con_5Y3
novel_circ_0008323	+	ENSG00000130766;	Con_5Y2,Con_5Y3
novel_circ_0018062	+	ENSG00000186350;	Con_5Y2,Con_5Y3
novel_circ_0018262	-	ENSG00000137073;	Con_5Y2,Con_5Y3
novel_circ_0018274	-	ENSG00000186638;	Con_5Y2,Con_5Y3
novel_circ_0016813	-	ENSG00000070756;	Con_5Y2,Con_5Y3
novel_circ_0017496	-	ENSG00000185697;	Con_5Y2,Con_5Y3
novel_circ_0017502	+	ENSG00000104218;	Con_5Y2,Con_5Y3
novel_circ_0017662	-	ENSG00000164944;	Con_5Y2,Con_5Y3
hsa_circ_0091366	+	ENSG00000131725;	Con_5Y3
hsa_circ_0089902	+	ENSG00000046651;	Con_5Y3

hsa_circ_0090360	-	ENSG00000065923;	Con_5Y3
hsa_circ_0002532	+	n/a	Con_5Y3
hsa_circ_0008420	+	ENSG00000175198;	Con_5Y3
hsa_circ_0029617	-	ENSG00000121390;	Con_5Y3
hsa_circ_0030585	-	ENSG00000125257;	Con_5Y3
hsa_circ_0002349	-	ENSG00000120860;	Con_5Y3
hsa_circ_0002957	-	ENSG00000084112;	Con_5Y3
hsa_circ_0029146	+	ENSG00000130783;	Con_5Y3
hsa_circ_0029160	-	ENSG00000150967;	Con_5Y3
hsa_circ_0029463	+	ENSG00000183495;	Con_5Y3
hsa_circ_0025582	+	ENSG00000257046;ENSG0	Con_5Y3
hsa_circ_0025705	-	ENSG00000064115;	Con_5Y3
hsa_circ_0025034	-	ENSG00000111206;	Con_5Y3
hsa_circ_0025783	-	ENSG00000110888;	Con_5Y3
hsa_circ_0026276	+	ENSG00000066084;	Con_5Y3
hsa_circ_0027352	+	ENSG00000135655;	Con_5Y3
hsa_circ_0020914	-	ENSG00000110713;	Con_5Y3
hsa_circ_0004637	-	ENSG00000110713;	Con_5Y3
hsa_circ_0003555	-	ENSG00000175216;	Con_5Y3
hsa_circ_0004988	-	ENSG00000166889;	Con_5Y3
hsa_circ_0023059	+	ENSG00000173120;	Con_5Y3
hsa_circ_0008648	+	ENSG00000166025;	Con_5Y3
hsa_circ_0019614	-	ENSG00000198408;	Con_5Y3
hsa_circ_0019764	-	ENSG00000138175;	Con_5Y3
hsa_circ_0020150	-	ENSG00000107581;	Con_5Y3
hsa_circ_0000267	-	ENSG00000189319;ENSG0	Con_5Y3
hsa_circ_0004583	-	ENSG00000175029;	Con_5Y3
hsa_circ_0017555	-	ENSG00000196372;	Con_5Y3
hsa_circ_0017599	+	ENSG00000134452;	Con_5Y3
hsa_circ_0017605	+	ENSG00000134453;	Con_5Y3
hsa_circ_0008629	+	ENSG00000138134;	Con_5Y3
hsa_circ_0003505	-	ENSG00000124422;	Con_5Y3
hsa_circ_0043427	-	ENSG00000125686;	Con_5Y3
hsa_circ_0043509	+	ENSG00000108349;	Con_5Y3
hsa_circ_0041539	-	ENSG00000185722;	Con_5Y3
hsa_circ_0003899	+	ENSG00000067596;	Con_5Y3
hsa_circ_0044796	+	ENSG00000108384;	Con_5Y3
hsa_circ_0045012	-	ENSG00000136492;	Con_5Y3
hsa_circ_0007534	+	ENSG00000198231;	Con_5Y3
hsa_circ_0041822	-	ENSG00000215041;	Con_5Y3
hsa_circ_0045559	+	ENSG00000109062;	Con_5Y3
hsa_circ_0045774	-	ENSG00000182473;	Con_5Y3
hsa_circ_0046395	-	ENSG00000141551;	Con_5Y3
hsa_circ_0046521	+	ENSG00000141556;	Con_5Y3
hsa_circ_0046596	+	ENSG00000141556;	Con_5Y3
hsa_circ_0042072	+	ENSG00000154914;	Con_5Y3
hsa_circ_0037830	+	ENSG00000038532;	Con_5Y3
hsa_circ_0007865	-	ENSG00000063854;	Con_5Y3
hsa_circ_0004049	+	ENSG00000174628;	Con_5Y3
hsa_circ_0038929	+	ENSG00000079616;	Con_5Y3
hsa_circ_0003617	-	ENSG00000103126;	Con_5Y3
hsa_circ_0039402	+	ENSG00000140718;	Con_5Y3
hsa_circ_0039921	+	ENSG00000167264;	Con_5Y3
hsa_circ_0040480	-	ENSG00000168411;	Con_5Y3
hsa_circ_0040919	+	ENSG00000170100;	Con_5Y3
hsa_circ_0003784	-	ENSG00000104133;	Con_5Y3
hsa_circ_0003879	+	ENSG00000138592;	Con_5Y3
hsa_circ_0003916	+	ENSG00000033800;	Con_5Y3

hsa_circ_0000641	-	ENSG00000140391;	Con_5Y3
hsa_circ_0003590	+	ENSG00000041357;	Con_5Y3
hsa_circ_0036984	+	ENSG00000173575;	Con_5Y3
hsa_circ_0004110	+	ENSG00000126777;	Con_5Y3
hsa_circ_0005205	+	ENSG00000100644;	Con_5Y3
hsa_circ_0032135	+	ENSG00000100644;	Con_5Y3
hsa_circ_0032201	+	ENSG00000100714;	Con_5Y3
hsa_circ_0032529	+	ENSG00000258653;ENSG0	Con_5Y3
hsa_circ_0006706	-	ENSG00000100596;	Con_5Y3
hsa_circ_0005682	-	ENSG00000011114;	Con_5Y3
hsa_circ_0049267	-	ENSG00000079999;	Con_5Y3
hsa_circ_0000910	+	ENSG00000099331;	Con_5Y3
hsa_circ_0005063	-	ENSG00000064607;	Con_5Y3
hsa_circ_0050590	+	ENSG00000105699;	Con_5Y3
hsa_circ_0050803	+	ENSG00000105738;	Con_5Y3
hsa_circ_0050899	+	ENSG00000130402;	Con_5Y3
hsa_circ_0002837	-	ENSG00000105329;	Con_5Y3
hsa_circ_0006240	+	ENSG00000167670;	Con_5Y3
hsa_circ_0007568	+	ENSG00000104805;	Con_5Y3
hsa_circ_0048113	-	ENSG00000175221;	Con_5Y3
hsa_circ_0046941	+	ENSG00000141404;	Con_5Y3
hsa_circ_0006937	-	ENSG00000141385;	Con_5Y3
hsa_circ_0046977	-	ENSG00000101624;	Con_5Y3
hsa_circ_0047133	+	ENSG00000101773;	Con_5Y3
hsa_circ_0047288	-	ENSG00000141447;	Con_5Y3
hsa_circ_0047315	-	ENSG00000141380;	Con_5Y3
hsa_circ_0046733	+	ENSG00000101596;	Con_5Y3
hsa_circ_0003552	+	ENSG00000075643;	Con_5Y3
hsa_circ_0062685	-	ENSG00000180957;	Con_5Y3
hsa_circ_0003468	-	ENSG00000100325;	Con_5Y3
hsa_circ_0062983	+	ENSG00000100225;	Con_5Y3
hsa_circ_0063206	+	ENSG00000100083;	Con_5Y3
hsa_circ_0002811	+	ENSG00000239900;	Con_5Y3
hsa_circ_0002610	-	ENSG00000100266;	Con_5Y3
hsa_circ_0002246	+	ENSG00000130638;	Con_5Y3
hsa_circ_0063781	+	ENSG00000186951;	Con_5Y3
hsa_circ_0059660	-	ENSG00000100997;	Con_5Y3
hsa_circ_0060518	+	ENSG00000101104;	Con_5Y3
hsa_circ_0060816	+	ENSG00000197818;	Con_5Y3
hsa_circ_0004871	+	ENSG00000149657;	Con_5Y3
hsa_circ_0061570	+	ENSG00000205726;	Con_5Y3
hsa_circ_0008021	+	ENSG00000160209;	Con_5Y3
hsa_circ_0008254	-	ENSG00000135250;	Con_5Y3
hsa_circ_0081861	+	ENSG00000135249;	Con_5Y3
hsa_circ_0082490	-	ENSG00000080802;	Con_5Y3
hsa_circ_0082707	-	ENSG00000157800;	Con_5Y3
hsa_circ_0007123	-	ENSG00000055609;	Con_5Y3
hsa_circ_0002852	+	ENSG00000136261;	Con_5Y3
hsa_circ_0001688	+	ENSG00000105926;	Con_5Y3
hsa_circ_0079739	+	ENSG00000254959;ENSG0	Con_5Y3
hsa_circ_0004150	+	ENSG00000146757;	Con_5Y3
hsa_circ_0005493	+	ENSG00000196367;	Con_5Y3
hsa_circ_0075617	+	ENSG00000153157;ENSG0	Con_5Y3
hsa_circ_0077817	-	ENSG00000152894;	Con_5Y3
hsa_circ_0078017	-	ENSG00000135597;	Con_5Y3
hsa_circ_0078367	+	ENSG00000213079;	Con_5Y3
hsa_circ_0005685	+	ENSG00000146426;	Con_5Y3
hsa_circ_0002971	+	ENSG00000130340;	Con_5Y3

hsa_circ_0075705	-	ENSG00000124789;	Con_5Y3
hsa_circ_0075456	+	ENSG00000124535;	Con_5Y3
hsa_circ_0003886	+	ENSG00000137275;	Con_5Y3
hsa_circ_0077141	-	ENSG00000146247;	Con_5Y3
hsa_circ_0077237	-	ENSG00000135317;	Con_5Y3
hsa_circ_0075356	+	ENSG00000113300;	Con_5Y3
hsa_circ_0072282	-	ENSG00000113569;	Con_5Y3
hsa_circ_0002121	-	ENSG00000083720;	Con_5Y3
hsa_circ_0004230	+	ENSG00000086200;	Con_5Y3
hsa_circ_0072925	+	ENSG00000083312;	Con_5Y3
hsa_circ_0007662	+	ENSG00000164134;	Con_5Y3
hsa_circ_0071279	+	ENSG00000164144;	Con_5Y3
hsa_circ_0071311	+	ENSG00000121210;	Con_5Y3
hsa_circ_0006234	+	ENSG00000109762;	Con_5Y3
hsa_circ_0068890	+	ENSG00000109685;	Con_5Y3
hsa_circ_0068793	+	ENSG00000174227;	Con_5Y3
hsa_circ_0069117	+	ENSG00000132405;	Con_5Y3
hsa_circ_0066636	+	ENSG00000036054;	Con_5Y3
hsa_circ_0066970	+	ENSG00000145088;	Con_5Y3
hsa_circ_0005028	+	ENSG00000154743;	Con_5Y3
hsa_circ_0004046	+	ENSG00000177463;	Con_5Y3
hsa_circ_0064491	-	ENSG00000131373;	Con_5Y3
hsa_circ_0067835	-	ENSG00000068885;	Con_5Y3
hsa_circ_0067841	+	ENSG00000113810;	Con_5Y3
hsa_circ_0065263	-	ENSG00000173473;	Con_5Y3
hsa_circ_0065932	+	ENSG00000164080;	Con_5Y3
hsa_circ_0055934	-	ENSG00000115652;	Con_5Y3
hsa_circ_0001075	+	ENSG00000135999;	Con_5Y3
hsa_circ_0056731	-	ENSG00000283228;ENSG0	Con_5Y3
hsa_circ_0056957	-	ENSG00000138399;	Con_5Y3
hsa_circ_0057551	+	ENSG00000196950;	Con_5Y3
hsa_circ_0057741	-	ENSG00000155744;	Con_5Y3
hsa_circ_0003747	+	ENSG00000138380;	Con_5Y3
hsa_circ_0054428	-	ENSG00000068784;	Con_5Y3
hsa_circ_0054963	+	ENSG00000119844;	Con_5Y3
hsa_circ_0055055	-	ENSG00000198380;	Con_5Y3
hsa_circ_0000970	+	ENSG00000151692;	Con_5Y3
hsa_circ_0055820	-	ENSG00000115514;	Con_5Y3
hsa_circ_0004437	+	ENSG00000163125;	Con_5Y3
hsa_circ_0007277	+	ENSG00000143164;	Con_5Y3
hsa_circ_0015157	-	ENSG00000143156;	Con_5Y3
hsa_circ_0005794	+	ENSG00000076321;	Con_5Y3
hsa_circ_0015285	+	ENSG00000117593;	Con_5Y3
hsa_circ_0003777	-	ENSG00000127481;	Con_5Y3
hsa_circ_0002815	+	ENSG00000058668;	Con_5Y3
hsa_circ_0010466	-	ENSG00000075151;	Con_5Y3
hsa_circ_0006962	-	ENSG00000162923;	Con_5Y3
hsa_circ_0002577	-	ENSG00000162923;	Con_5Y3
hsa_circ_0016750	-	ENSG00000143776;	Con_5Y3
hsa_circ_0016960	-	ENSG00000135749;	Con_5Y3
hsa_circ_0011401	+	ENSG00000116478;	Con_5Y3
hsa_circ_0004013	-	ENSG00000116514;	Con_5Y3
hsa_circ_0004660	-	ENSG00000142687;	Con_5Y3
hsa_circ_0003494	-	ENSG00000085840;	Con_5Y3
hsa_circ_0009530	-	ENSG00000162408;	Con_5Y3
hsa_circ_0003114	-	ENSG00000162408;	Con_5Y3
hsa_circ_0088041	-	ENSG00000106868;	Con_5Y3
hsa_circ_0088087	+	ENSG00000119471;	Con_5Y3

hsa_circ_0086645	+	ENSG00000086102;	Con_5Y3
hsa_circ_0005739	+	ENSG00000136938;	Con_5Y3
hsa_circ_0001818	-	ENSG00000104517;	Con_5Y3
hsa_circ_0001825	-	ENSG00000066827;	Con_5Y3
hsa_circ_0085703	-	ENSG00000167632;	Con_5Y3
hsa_circ_0083258	+	ENSG00000283239;ENSG0	Con_5Y3
novel_circ_0018624	-	ENSG00000133131;	Con_5Y3
novel_circ_0018757	+	ENSG00000086712;	Con_5Y3
novel_circ_0018832	-	ENSG00000069509;	Con_5Y3
novel_circ_0018855	-	ENSG00000072501;	Con_5Y3
novel_circ_0003110	-	ENSG00000102753;	Con_5Y3
novel_circ_0001805	-	n/a	Con_5Y3
novel_circ_0001830	+	ENSG00000136048;	Con_5Y3
novel_circ_0001995	-	ENSG00000173064;	Con_5Y3
novel_circ_0002031	-	ENSG00000111412;	Con_5Y3
novel_circ_0002079	+	ENSG00000170633;	Con_5Y3
novel_circ_0002128	+	ENSG00000130783;	Con_5Y3
novel_circ_0002129	+	ENSG00000130783;	Con_5Y3
novel_circ_0002130	+	ENSG00000130783;	Con_5Y3
novel_circ_0002134	-	n/a	Con_5Y3
novel_circ_0002272	+	ENSG00000111726;	Con_5Y3
novel_circ_0002443	+	ENSG00000152556;	Con_5Y3
novel_circ_0002446	-	ENSG00000174233;	Con_5Y3
novel_circ_0002484	+	ENSG00000161813;	Con_5Y3
novel_circ_0002519	+	ENSG00000256417;	Con_5Y3
novel_circ_0002610	+	n/a	Con_5Y3
novel_circ_0001043	-	ENSG00000110321;	Con_5Y3
novel_circ_0001091	+	ENSG00000170145;	Con_5Y3
novel_circ_0001270	-	ENSG00000151116;	Con_5Y3
novel_circ_0001292	-	ENSG00000121621;	Con_5Y3
novel_circ_0001308	-	ENSG00000205531;	Con_5Y3
novel_circ_0001359	+	ENSG00000026508;	Con_5Y3
novel_circ_0001471	-	ENSG00000167987;	Con_5Y3
novel_circ_0001548	+	ENSG00000110717;	Con_5Y3
novel_circ_0000022	-	ENSG00000186862;	Con_5Y3
novel_circ_0000044	-	ENSG00000198408;	Con_5Y3
novel_circ_0000168	-	ENSG00000187164;	Con_5Y3
novel_circ_0000308	-	ENSG00000089876;	Con_5Y3
novel_circ_0000340	+	ENSG00000132334;	Con_5Y3
novel_circ_0000368	+	ENSG00000152455;	Con_5Y3
novel_circ_0000466	-	ENSG00000169126;	Con_5Y3
novel_circ_0000728	+	ENSG00000060339;	Con_5Y3
novel_circ_0000730	+	ENSG00000060339;	Con_5Y3
novel_circ_0000779	-	ENSG00000122882;	Con_5Y3
novel_circ_0000804	+	ENSG00000156110;	Con_5Y3
novel_circ_0000876	+	ENSG00000138182;	Con_5Y3
novel_circ_0000890	+	ENSG00000095564;	Con_5Y3
novel_circ_0005273	-	ENSG00000272636;	Con_5Y3
novel_circ_0005516	-	ENSG00000278540;	Con_5Y3
novel_circ_0005591	+	ENSG00000033627;	Con_5Y3
novel_circ_0005824	+	ENSG00000108406;	Con_5Y3
novel_circ_0005903	-	ENSG00000108510;	Con_5Y3
novel_circ_0005911	-	ENSG00000141252;	Con_5Y3
novel_circ_0005944	+	ENSG00000258890;	Con_5Y3
novel_circ_0006171	-	ENSG00000141551;	Con_5Y3
novel_circ_0004890	+	ENSG00000102910;	Con_5Y3
novel_circ_0004936	-	ENSG00000125124;	Con_5Y3
novel_circ_0005150	-	ENSG00000131148;	Con_5Y3

novel_circ_0005222	+	ENSG00000197912;	Con_5Y3
novel_circ_0005225	+	ENSG00000185324;	Con_5Y3
novel_circ_0004074	+	ENSG00000137815;	Con_5Y3
novel_circ_0004084	+	ENSG00000159433;	Con_5Y3
novel_circ_0004106	+	ENSG00000092470;	Con_5Y3
novel_circ_0004131	-	ENSG00000104133;	Con_5Y3
novel_circ_0004360	+	ENSG00000137807;	Con_5Y3
novel_circ_0004416	-	ENSG00000169375;	Con_5Y3
novel_circ_0004468	+	ENSG00000136381;	Con_5Y3
novel_circ_0003333	+	ENSG00000089902;	Con_5Y3
novel_circ_0003616	-	ENSG00000237356;	Con_5Y3
novel_circ_0003623	-	ENSG00000198554;	Con_5Y3
novel_circ_0003686	+	ENSG00000054654;	Con_5Y3
novel_circ_0003802	+	ENSG00000119685;	Con_5Y3
novel_circ_0003907	+	ENSG00000090060;	Con_5Y3
novel_circ_0003932	-	ENSG00000205476;	Con_5Y3
novel_circ_0006748	+	ENSG00000127511;	Con_5Y3
novel_circ_0006777	-	ENSG00000064607;	Con_5Y3
novel_circ_0006826	+	ENSG00000172009;	Con_5Y3
novel_circ_0006893	-	n/a	Con_5Y3
novel_circ_0006913	-	ENSG00000011332;	Con_5Y3
novel_circ_0007038	-	ENSG00000074219;	Con_5Y3
novel_circ_0007045	-	ENSG00000268006;	Con_5Y3
novel_circ_0007130	-	ENSG00000031823;	Con_5Y3
novel_circ_0006236	-	ENSG00000141385;	Con_5Y3
novel_circ_0006437	-	ENSG00000078043;	Con_5Y3
novel_circ_0006543	+	ENSG00000141664;	Con_5Y3
novel_circ_0006625	+	ENSG00000017797;	Con_5Y3
novel_circ_0009805	+	ENSG00000100330;	Con_5Y3
novel_circ_0009904	+	ENSG00000100393;	Con_5Y3
novel_circ_0009910	-	ENSG00000100401;	Con_5Y3
novel_circ_0009950	+	ENSG00000100376;	Con_5Y3
novel_circ_0009426	-	ENSG00000101160;	Con_5Y3
novel_circ_0009556	-	ENSG00000142188;	Con_5Y3
novel_circ_0015828	-	ENSG00000196411;	Con_5Y3
novel_circ_0015902	-	ENSG00000091127;	Con_5Y3
novel_circ_0016192	-	ENSG00000117868;	Con_5Y3
novel_circ_0016322	-	ENSG00000078399;ENSG0	Con_5Y3
novel_circ_0016388	-	ENSG00000228878;	Con_5Y3
novel_circ_0016630	+	ENSG00000135205;	Con_5Y3
novel_circ_0016646	+	ENSG00000187257;	Con_5Y3
novel_circ_0016654	+	ENSG00000006576;	Con_5Y3
novel_circ_0016718	+	ENSG00000127914;	Con_5Y3
novel_circ_0016725	-	ENSG00000001631;ENSG0	Con_5Y3
novel_circ_0014953	+	ENSG00000112297;	Con_5Y3
novel_circ_0015011	-	ENSG00000153975;	Con_5Y3
novel_circ_0015406	+	ENSG00000112312;	Con_5Y3
novel_circ_0015518	+	ENSG00000024048;	Con_5Y3
novel_circ_0015765	-	ENSG00000112159;	Con_5Y3
novel_circ_0013972	+	ENSG00000145495;	Con_5Y3
novel_circ_0014155	+	ENSG00000145833;	Con_5Y3
novel_circ_0014189	+	ENSG00000120733;	Con_5Y3
novel_circ_0014214	+	ENSG00000015479;ENSG0	Con_5Y3
novel_circ_0014235	-	ENSG00000131504;	Con_5Y3
novel_circ_0014275	-	n/a	Con_5Y3
novel_circ_0014321	+	ENSG00000145907;	Con_5Y3
novel_circ_0014329	+	ENSG00000155506;	Con_5Y3
novel_circ_0014343	+	ENSG00000055163;	Con_5Y3

novel_circ_0014365	+	ENSG00000113328;	Con_5Y3
novel_circ_0014490	-	ENSG00000113360;	Con_5Y3
novel_circ_0014610	+	ENSG00000039123;	Con_5Y3
novel_circ_0014615	-	ENSG00000134352;	Con_5Y3
novel_circ_0014659	+	ENSG00000112877;	Con_5Y3
novel_circ_0014673	+	ENSG00000113597;	Con_5Y3
novel_circ_0014754	-	ENSG00000198780;	Con_5Y3
novel_circ_0014785	-	ENSG00000132842;	Con_5Y3
novel_circ_0014797	-	ENSG00000132837;	Con_5Y3
novel_circ_0014828	+	ENSG00000113318;	Con_5Y3
novel_circ_0014876	-	ENSG00000113391;	Con_5Y3
novel_circ_0013087	-	ENSG00000109323;	Con_5Y3
novel_circ_0013198	-	ENSG00000164073;	Con_5Y3
novel_circ_0013239	+	ENSG00000163945;	Con_5Y3
novel_circ_0013392	-	ENSG00000170088;	Con_5Y3
novel_circ_0013478	-	ENSG00000164323;	Con_5Y3
novel_circ_0013551	-	ENSG00000091490;	Con_5Y3
novel_circ_0013830	+	ENSG00000138758;	Con_5Y3
novel_circ_0013838	-	ENSG00000118816;	Con_5Y3
novel_circ_0011771	+	ENSG00000114354;	Con_5Y3
novel_circ_0011817	+	ENSG00000197548;	Con_5Y3
novel_circ_0011824	-	ENSG00000206530;ENSG0	Con_5Y3
novel_circ_0011885	-	ENSG00000051341;	Con_5Y3
novel_circ_0012070	-	ENSG00000114107;	Con_5Y3
novel_circ_0012205	+	ENSG00000168827;	Con_5Y3
novel_circ_0012214	+	ENSG00000113810;	Con_5Y3
novel_circ_0012240	-	ENSG00000173889;	Con_5Y3
novel_circ_0012462	+	ENSG00000180370;	Con_5Y3
novel_circ_0012600	+	ENSG00000182973;	Con_5Y3
novel_circ_0012670	+	ENSG00000114742;	Con_5Y3
novel_circ_0012683	-	ENSG00000168038;	Con_5Y3
novel_circ_0012911	+	n/a	Con_5Y3
novel_circ_0012976	-	ENSG00000151276;	Con_5Y3
novel_circ_0013032	+	ENSG00000169379;	Con_5Y3
novel_circ_0013040	+	ENSG00000163719;	Con_5Y3
novel_circ_0010147	-	ENSG00000074054;	Con_5Y3
novel_circ_0010317	+	ENSG00000115159;	Con_5Y3
novel_circ_0010631	+	ENSG00000055044;	Con_5Y3
novel_circ_0010781	+	n/a	Con_5Y3
novel_circ_0010832	-	ENSG00000036257;	Con_5Y3
novel_circ_0010852	-	ENSG00000153827;	Con_5Y3
novel_circ_0010921	-	ENSG00000119778;	Con_5Y3
novel_circ_0010996	+	ENSG00000084676;	Con_5Y3
novel_circ_0011010	-	ENSG00000119772;	Con_5Y3
novel_circ_0011242	+	ENSG00000057935;	Con_5Y3
novel_circ_0011354	+	ENSG00000162869;	Con_5Y3
novel_circ_0011521	+	ENSG00000169764;	Con_5Y3
novel_circ_0011659	-	ENSG00000132305;	Con_5Y3
novel_circ_0011740	-	ENSG00000135951;	Con_5Y3
novel_circ_0007396	+	ENSG00000142621;	Con_5Y3
novel_circ_0007628	+	ENSG00000152061;	Con_5Y3
novel_circ_0007678	+	ENSG00000057252;	Con_5Y3
novel_circ_0007765	+	ENSG00000116747;	Con_5Y3
novel_circ_0007769	+	ENSG00000134371;	Con_5Y3
novel_circ_0007828	+	ENSG00000077157;	Con_5Y3
novel_circ_0008011	+	ENSG00000185842;	Con_5Y3
novel_circ_0008104	+	ENSG00000004487;ENSG0	Con_5Y3
novel_circ_0008293	-	ENSG00000117614;	Con_5Y3

novel_circ_0008327	+	ENSG00000204138;	Con_5Y3
novel_circ_0008368	-	ENSG00000134644;	Con_5Y3
novel_circ_0008446	-	ENSG00000162591;	Con_5Y3
novel_circ_0008497	+	ENSG00000084073;	Con_5Y3
novel_circ_0008794	+	ENSG00000198160;	Con_5Y3
novel_circ_0008987	-	ENSG00000067208;	Con_5Y3
novel_circ_0017888	-	ENSG00000119396;	Con_5Y3
novel_circ_0017913	+	ENSG00000011454;	Con_5Y3
novel_circ_0018088	-	ENSG00000148396;	Con_5Y3
novel_circ_0018312	+	ENSG00000165304;	Con_5Y3
novel_circ_0016847	-	ENSG00000155096;	Con_5Y3
novel_circ_0016947	+	ENSG00000156787;	Con_5Y3
novel_circ_0017138	-	ENSG00000254812;	Con_5Y3
novel_circ_0017176	+	ENSG00000155975;	Con_5Y3
novel_circ_0017232	+	ENSG00000147454;	Con_5Y3
novel_circ_0017270	+	ENSG00000147421;	Con_5Y3
novel_circ_0017424	+	ENSG00000169139;	Con_5Y3
novel_circ_0017529	-	ENSG00000066777;	Con_5Y3
novel_circ_0017543	-	ENSG00000140396;	Con_5Y3
novel_circ_0017586	+	ENSG00000133739;	Con_5Y3
novel_circ_0017588	+	ENSG00000185015;	Con_5Y3
hsa_circ_0089978	-	ENSG00000102098;	CVB5_5Y1
hsa_circ_0090183	+	ENSG00000250349;ENSG0	CVB5_5Y1
hsa_circ_0091113	-	ENSG00000102158;	CVB5_5Y1
hsa_circ_0008744	+	ENSG00000134900;	CVB5_5Y1
hsa_circ_0031011	-	ENSG00000150401;	CVB5_5Y1
hsa_circ_0030065	+	ENSG00000172766;	CVB5_5Y1
hsa_circ_0030409	+	ENSG00000083535;	CVB5_5Y1
hsa_circ_0030741	+	ENSG00000125304;	CVB5_5Y1
hsa_circ_0029222	-	ENSG00000139697;	CVB5_5Y1
hsa_circ_0000460	+	ENSG00000185344;	CVB5_5Y1
hsa_circ_0029568	-	ENSG00000090615;	CVB5_5Y1
hsa_circ_0026326	+	ENSG00000196876;	CVB5_5Y1
hsa_circ_0004296	-	ENSG00000134954;	CVB5_5Y1
hsa_circ_0020766	-	ENSG00000078902;	CVB5_5Y1
hsa_circ_0004960	-	ENSG00000110713;	CVB5_5Y1
hsa_circ_0022054	-	ENSG00000109920;	CVB5_5Y1
hsa_circ_0023180	+	ENSG00000162337;	CVB5_5Y1
hsa_circ_0008391	+	ENSG00000110075;	CVB5_5Y1
hsa_circ_0023559	-	ENSG00000168014;	CVB5_5Y1
hsa_circ_0023928	-	ENSG00000073921;	CVB5_5Y1
hsa_circ_0020022	+	ENSG00000108055;	CVB5_5Y1
hsa_circ_0004676	+	ENSG00000181192;	CVB5_5Y1
hsa_circ_0018440	-	ENSG00000151150;	CVB5_5Y1
hsa_circ_0018581	+	ENSG00000165732;	CVB5_5Y1
hsa_circ_0018620	+	ENSG00000156515;	CVB5_5Y1
hsa_circ_0005828	-	ENSG00000062650;	CVB5_5Y1
hsa_circ_0004202	-	ENSG00000107929;	CVB5_5Y1
hsa_circ_0004224	-	ENSG00000107864;	CVB5_5Y1
hsa_circ_0004445	-	ENSG00000167193;	CVB5_5Y1
hsa_circ_0044767	-	ENSG00000011143;	CVB5_5Y1
hsa_circ_0044956	-	ENSG00000170832;	CVB5_5Y1
hsa_circ_0045309	+	ENSG00000258890;	CVB5_5Y1
hsa_circ_0003646	+	ENSG00000167889;	CVB5_5Y1
hsa_circ_0045938	-	ENSG00000055483;	CVB5_5Y1
hsa_circ_0039635	-	ENSG00000125107;	CVB5_5Y1
hsa_circ_0040364	-	ENSG00000040199;	CVB5_5Y1
hsa_circ_0034699	-	ENSG00000137806;	CVB5_5Y1

hsa_circ_0002891	+	ENSG00000167004;	CVB5_5Y1
hsa_circ_0008693	-	ENSG00000092439;	CVB5_5Y1
hsa_circ_0004456	-	ENSG00000129003;	CVB5_5Y1
hsa_circ_0037007	+	ENSG00000173575;	CVB5_5Y1
hsa_circ_0031638	-	ENSG00000198604;	CVB5_5Y1
hsa_circ_0049475	+	ENSG00000130175;	CVB5_5Y1
hsa_circ_0049891	+	ENSG00000105072;	CVB5_5Y1
hsa_circ_0000928	+	ENSG00000167595;	CVB5_5Y1
hsa_circ_0051220	+	ENSG00000142046;ENSG0	CVB5_5Y1
hsa_circ_0003999	+	ENSG00000008382;	CVB5_5Y1
hsa_circ_0002979	+	ENSG00000213892;	CVB5_5Y1
hsa_circ_0004551	-	ENSG00000105355;	CVB5_5Y1
hsa_circ_0052189	-	ENSG00000105048;	CVB5_5Y1
hsa_circ_0048780	-	ENSG00000196365;	CVB5_5Y1
hsa_circ_0048965	-	ENSG00000171105;	CVB5_5Y1
hsa_circ_0047748	+	ENSG00000091157;	CVB5_5Y1
hsa_circ_0062937	-	ENSG00000183530;	CVB5_5Y1
hsa_circ_0063328	-	ENSG00000100201;	CVB5_5Y1
hsa_circ_0002406	+	ENSG00000054611;	CVB5_5Y1
hsa_circ_0059577	+	ENSG00000088970;	CVB5_5Y1
hsa_circ_0006922	+	ENSG00000171456;	CVB5_5Y1
hsa_circ_0007051	+	ENSG00000101447;	CVB5_5Y1
hsa_circ_0060896	-	ENSG00000054793;	CVB5_5Y1
hsa_circ_0061025	-	ENSG00000130699;	CVB5_5Y1
hsa_circ_0081856	-	ENSG00000091127;	CVB5_5Y1
hsa_circ_0082447	+	ENSG00000131558;	CVB5_5Y1
hsa_circ_0082865	+	ENSG00000204946;	CVB5_5Y1
hsa_circ_0005096	+	ENSG00000136243;	CVB5_5Y1
hsa_circ_0005366	+	ENSG00000164543;	CVB5_5Y1
hsa_circ_0078143	-	ENSG00000146414;	CVB5_5Y1
hsa_circ_0075680	-	ENSG00000047579;	CVB5_5Y1
hsa_circ_0075728	-	ENSG00000124789;	CVB5_5Y1
hsa_circ_0004209	-	ENSG00000213676;	CVB5_5Y1
hsa_circ_0074893	+	ENSG00000113643;	CVB5_5Y1
hsa_circ_0075332	-	ENSG00000197226;	CVB5_5Y1
hsa_circ_0075388	-	ENSG00000204628;	CVB5_5Y1
hsa_circ_0070605	+	ENSG00000164022;	CVB5_5Y1
hsa_circ_0005669	+	ENSG00000109466;	CVB5_5Y1
hsa_circ_0068896	+	ENSG00000109685;	CVB5_5Y1
hsa_circ_0070051	+	ENSG00000138771;	CVB5_5Y1
hsa_circ_0070057	+	ENSG00000138758;	CVB5_5Y1
hsa_circ_0002515	-	ENSG00000196526;	CVB5_5Y1
hsa_circ_0070261	-	ENSG00000189308;	CVB5_5Y1
hsa_circ_0004577	+	ENSG00000118762;	CVB5_5Y1
hsa_circ_0001425	-	ENSG00000138642;	CVB5_5Y1
hsa_circ_0006132	+	ENSG00000173193;	CVB5_5Y1
hsa_circ_0064346	+	ENSG00000154743;	CVB5_5Y1
hsa_circ_0007210	-	ENSG00000241288;	CVB5_5Y1
hsa_circ_0068255	-	ENSG00000175193;ENSG0	CVB5_5Y1
hsa_circ_0068259	-	ENSG00000114770;	CVB5_5Y1
hsa_circ_0003636	-	ENSG00000160746;	CVB5_5Y1
hsa_circ_0065926	+	ENSG00000164080;	CVB5_5Y1
hsa_circ_0056290	-	ENSG00000074054;	CVB5_5Y1
hsa_circ_0007814	-	ENSG00000121988;	CVB5_5Y1
hsa_circ_0008459	-	ENSG00000144445;	CVB5_5Y1
hsa_circ_0004526	+	ENSG00000162804;	CVB5_5Y1
hsa_circ_0054499	-	ENSG00000138081;	CVB5_5Y1
hsa_circ_0055471	-	ENSG00000068654;	CVB5_5Y1

hsa_circ_0009689	+	ENSG00000130939;	CVB5_5Y1
hsa_circ_0013646	-	ENSG00000009307;	CVB5_5Y1
hsa_circ_0004222	+	ENSG00000048707;	CVB5_5Y1
hsa_circ_0010039	-	ENSG00000132906;	CVB5_5Y1
hsa_circ_0014684	+	ENSG00000260238;ENSG0	CVB5_5Y1
hsa_circ_0007921	+	ENSG00000197965;	CVB5_5Y1
hsa_circ_0006924	+	ENSG00000135837;	CVB5_5Y1
hsa_circ_0011361	+	ENSG00000084652;	CVB5_5Y1
hsa_circ_0011692	-	ENSG00000196182;	CVB5_5Y1
hsa_circ_0000082	+	ENSG00000185483;	CVB5_5Y1
hsa_circ_0013107	+	ENSG00000065243;	CVB5_5Y1
hsa_circ_0013200	+	ENSG00000122483;	CVB5_5Y1
hsa_circ_0088613	+	ENSG00000196814;	CVB5_5Y1
hsa_circ_0086397	+	ENSG00000164975;	CVB5_5Y1
hsa_circ_0086241	-	ENSG00000080298;	CVB5_5Y1
hsa_circ_0003624	+	ENSG00000107341;	CVB5_5Y1
hsa_circ_0003084	+	ENSG00000107036;	CVB5_5Y1
hsa_circ_0087400	-	ENSG00000135052;	CVB5_5Y1
hsa_circ_0005411	+	ENSG00000012232;	CVB5_5Y1
hsa_circ_0001803	+	ENSG00000254087;	CVB5_5Y1
hsa_circ_0084867	-	ENSG00000104320;	CVB5_5Y1
novel_circ_0018775	-	ENSG00000147010;	CVB5_5Y1
novel_circ_0018805	+	ENSG00000101868;	CVB5_5Y1
novel_circ_0018895	-	ENSG00000085224;	CVB5_5Y1
novel_circ_0018932	+	ENSG00000146950;	CVB5_5Y1
novel_circ_0002850	-	ENSG00000183087;	CVB5_5Y1
novel_circ_0002971	-	ENSG00000120694;	CVB5_5Y1
novel_circ_0002973	-	ENSG00000120694;	CVB5_5Y1
novel_circ_0003073	-	ENSG00000123200;	CVB5_5Y1
novel_circ_0003120	+	ENSG00000176124;	CVB5_5Y1
novel_circ_0002058	-	ENSG00000089154;	CVB5_5Y1
novel_circ_0002425	-	ENSG00000111371;	CVB5_5Y1
novel_circ_0002451	-	ENSG00000139636;	CVB5_5Y1
novel_circ_0002517	+	ENSG00000197111;ENSG0	CVB5_5Y1
novel_circ_0002654	+	ENSG00000127329;	CVB5_5Y1
novel_circ_0002727	-	ENSG00000198707;	CVB5_5Y1
novel_circ_0002743	-	ENSG00000049130;	CVB5_5Y1
novel_circ_0001026	+	ENSG00000133805;	CVB5_5Y1
novel_circ_0001037	-	ENSG00000152404;	CVB5_5Y1
novel_circ_0001269	-	ENSG00000151116;	CVB5_5Y1
novel_circ_0001414	-	ENSG00000135365;	CVB5_5Y1
novel_circ_0001593	+	ENSG00000110092;	CVB5_5Y1
novel_circ_0001607	-	ENSG00000162105;	CVB5_5Y1
novel_circ_0001656	-	ENSG00000149269;	CVB5_5Y1
novel_circ_0001758	+	ENSG00000166004;	CVB5_5Y1
novel_circ_0001771	+	ENSG00000284057;ENSG0	CVB5_5Y1
novel_circ_0000380	-	ENSG00000152465;	CVB5_5Y1
novel_circ_0000678	+	ENSG00000096717;	CVB5_5Y1
novel_circ_0000963	-	ENSG00000077147;	CVB5_5Y1
novel_circ_0005355	-	ENSG00000124422;	CVB5_5Y1
novel_circ_0005438	+	ENSG00000176208;	CVB5_5Y1
novel_circ_0005553	-	ENSG00000074755;	CVB5_5Y1
novel_circ_0005699	+	ENSG00000082641;	CVB5_5Y1
novel_circ_0005744	-	ENSG00000011258;	CVB5_5Y1
novel_circ_0005761	+	ENSG00000029725;	CVB5_5Y1
novel_circ_0005797	+	ENSG00000108384;	CVB5_5Y1
novel_circ_0005818	-	ENSG00000108395;	CVB5_5Y1
novel_circ_0005971	-	ENSG00000198920;	CVB5_5Y1

novel_circ_0005991	-	ENSG00000198265;	CVB5_5Y1
novel_circ_0006048	+	ENSG00000125450;	CVB5_5Y1
novel_circ_0006051	+	ENSG00000073350;	CVB5_5Y1
novel_circ_0006054	-	ENSG00000108469;	CVB5_5Y1
novel_circ_0006149	+	ENSG00000185298;	CVB5_5Y1
novel_circ_0006168	-	ENSG00000176155;	CVB5_5Y1
novel_circ_0004627	-	ENSG00000059145;	CVB5_5Y1
novel_circ_0004639	+	ENSG00000103429;	CVB5_5Y1
novel_circ_0004715	-	ENSG00000007392;	CVB5_5Y1
novel_circ_0004720	+	ENSG00000102897;	CVB5_5Y1
novel_circ_0004753	-	ENSG00000083093;	CVB5_5Y1
novel_circ_0004866	-	ENSG00000069345;	CVB5_5Y1
novel_circ_0005152	-	ENSG00000103248;	CVB5_5Y1
novel_circ_0005198	-	ENSG00000187555;	CVB5_5Y1
novel_circ_0004125	+	ENSG00000104131;	CVB5_5Y1
novel_circ_0004135	+	ENSG00000171763;	CVB5_5Y1
novel_circ_0004192	-	ENSG00000047346;	CVB5_5Y1
novel_circ_0004422	-	ENSG00000260269;	CVB5_5Y1
novel_circ_0004477	+	ENSG00000166557;	CVB5_5Y1
novel_circ_0003357	+	ENSG00000126214;	CVB5_5Y1
novel_circ_0003615	-	ENSG00000237356;	CVB5_5Y1
novel_circ_0003762	+	ENSG00000119707;	CVB5_5Y1
novel_circ_0003851	+	ENSG00000100722;	CVB5_5Y1
novel_circ_0006689	-	ENSG00000197933;	CVB5_5Y1
novel_circ_0006712	+	ENSG00000104957;	CVB5_5Y1
novel_circ_0006731	-	ENSG00000141867;	CVB5_5Y1
novel_circ_0006820	-	ENSG00000176619;	CVB5_5Y1
novel_circ_0007070	-	ENSG00000105426;	CVB5_5Y1
novel_circ_0007092	-	ENSG00000105048;	CVB5_5Y1
novel_circ_0007148	+	n/a	CVB5_5Y1
novel_circ_0007149	-	ENSG00000142449;	CVB5_5Y1
novel_circ_0006581	+	ENSG00000130856;	CVB5_5Y1
novel_circ_0009735	+	ENSG00000099991;	CVB5_5Y1
novel_circ_0009836	-	ENSG00000233080;	CVB5_5Y1
novel_circ_0009847	-	ENSG00000100065;	CVB5_5Y1
novel_circ_0009873	+	ENSG00000100226;	CVB5_5Y1
novel_circ_0009884	+	ENSG00000284431;ENSG0	CVB5_5Y1
novel_circ_0009989	+	ENSG00000224271;	CVB5_5Y1
novel_circ_0009128	-	ENSG00000282826;	CVB5_5Y1
novel_circ_0009459	+	ENSG00000101187;	CVB5_5Y1
novel_circ_0009470	-	ENSG00000101210;	CVB5_5Y1
novel_circ_0009490	+	ENSG00000155313;	CVB5_5Y1
novel_circ_0009673	-	ENSG00000160294;	CVB5_5Y1
novel_circ_0015832	-	ENSG00000106397;	CVB5_5Y1
novel_circ_0015851	-	ENSG00000161040;	CVB5_5Y1
novel_circ_0016035	+	ENSG00000155561;	CVB5_5Y1
novel_circ_0016087	+	ENSG00000055130;	CVB5_5Y1
novel_circ_0016245	-	ENSG00000286192;ENSG0	CVB5_5Y1
novel_circ_0016620	-	ENSG00000127952;	CVB5_5Y1
novel_circ_0014964	-	ENSG00000025796;	CVB5_5Y1
novel_circ_0014974	+	ENSG00000153157;ENSG0	CVB5_5Y1
novel_circ_0015073	-	ENSG00000152894;	CVB5_5Y1
novel_circ_0015218	-	ENSG00000155906;	CVB5_5Y1
novel_circ_0015290	+	ENSG00000164674;	CVB5_5Y1
novel_circ_0015329	+	ENSG00000130396;	CVB5_5Y1
novel_circ_0015357	+	ENSG00000112584;	CVB5_5Y1
novel_circ_0015485	-	ENSG00000246982;	CVB5_5Y1
novel_circ_0015488	+	ENSG00000112078;	CVB5_5Y1

novel_circ_0015576	-	ENSG00000001084;	CVB5_5Y1
novel_circ_0015606	+	ENSG00000112208;	CVB5_5Y1
novel_circ_0015616	+	ENSG00000145982;	CVB5_5Y1
novel_circ_0015655	-	ENSG00000119899;	CVB5_5Y1
novel_circ_0015799	-	ENSG00000132423;	CVB5_5Y1
novel_circ_0013979	+	ENSG00000164236;	CVB5_5Y1
novel_circ_0014024	+	ENSG00000047188;	CVB5_5Y1
novel_circ_0014152	+	ENSG00000145833;	CVB5_5Y1
novel_circ_0014156	+	ENSG00000145833;	CVB5_5Y1
novel_circ_0014158	+	ENSG00000132570;	CVB5_5Y1
novel_circ_0014160	+	ENSG00000132570;	CVB5_5Y1
novel_circ_0014423	+	ENSG00000165671;	CVB5_5Y1
novel_circ_0014428	+	ENSG00000197451;	CVB5_5Y1
novel_circ_0014566	+	ENSG00000172262;	CVB5_5Y1
novel_circ_0014648	+	ENSG00000086200;	CVB5_5Y1
novel_circ_0014847	+	ENSG00000152348;	CVB5_5Y1
novel_circ_0014866	+	ENSG00000071539;	CVB5_5Y1
novel_circ_0013082	-	ENSG00000109323;	CVB5_5Y1
novel_circ_0013090	-	ENSG00000164038;	CVB5_5Y1
novel_circ_0013343	-	ENSG00000198589;	CVB5_5Y1
novel_circ_0013623	-	ENSG00000109814;	CVB5_5Y1
novel_circ_0013785	-	ENSG00000132463;	CVB5_5Y1
novel_circ_0013839	-	ENSG00000196526;	CVB5_5Y1
novel_circ_0011825	-	ENSG00000285943;ENSG0	CVB5_5Y1
novel_circ_0012126	-	ENSG00000018408;	CVB5_5Y1
novel_circ_0012235	+	ENSG00000008952;	CVB5_5Y1
novel_circ_0012276	+	ENSG00000114346;	CVB5_5Y1
novel_circ_0012287	+	ENSG00000177694;	CVB5_5Y1
novel_circ_0012288	-	ENSG00000131374;	CVB5_5Y1
novel_circ_0012435	+	ENSG00000242086;	CVB5_5Y1
novel_circ_0012520	+	ENSG00000174738;	CVB5_5Y1
novel_circ_0012639	+	ENSG00000170248;	CVB5_5Y1
novel_circ_0012980	+	ENSG00000144741;	CVB5_5Y1
novel_circ_0013045	+	ENSG00000080200;	CVB5_5Y1
novel_circ_0010239	-	ENSG00000115866;	CVB5_5Y1
novel_circ_0010346	+	ENSG00000136536;	CVB5_5Y1
novel_circ_0010578	-	ENSG00000197121;	CVB5_5Y1
novel_circ_0010605	-	ENSG00000013441;	CVB5_5Y1
novel_circ_0010765	+	ENSG00000135924;	CVB5_5Y1
novel_circ_0010871	+	ENSG00000173692;	CVB5_5Y1
novel_circ_0010873	+	ENSG00000173692;	CVB5_5Y1
novel_circ_0010886	+	ENSG00000144535;	CVB5_5Y1
novel_circ_0010938	-	ENSG00000068024;	CVB5_5Y1
novel_circ_0011162	+	ENSG00000234171;	CVB5_5Y1
novel_circ_0011210	-	ENSG00000119787;	CVB5_5Y1
novel_circ_0011317	-	ENSG00000068784;	CVB5_5Y1
novel_circ_0011376	-	ENSG00000085760;	CVB5_5Y1
novel_circ_0011426	-	ENSG00000115464;	CVB5_5Y1
novel_circ_0011440	-	ENSG00000115464;	CVB5_5Y1
novel_circ_0011463	-	ENSG00000082898;	CVB5_5Y1
novel_circ_0007204	-	ENSG00000085491;	CVB5_5Y1
novel_circ_0007210	+	ENSG00000116266;	CVB5_5Y1
novel_circ_0007327	-	ENSG00000221978;	CVB5_5Y1
novel_circ_0007360	+	ENSG00000163125;	CVB5_5Y1
novel_circ_0007463	-	ENSG00000116580;	CVB5_5Y1
novel_circ_0007623	-	ENSG00000179051;	CVB5_5Y1
novel_circ_0007735	-	ENSG00000047410;	CVB5_5Y1
novel_circ_0007976	+	ENSG00000067704;	CVB5_5Y1

novel_circ_0007980	+	ENSG00000116141;	CVB5_5Y1
novel_circ_0008044	+	ENSG00000185842;	CVB5_5Y1
novel_circ_0008085	-	ENSG00000154370;	CVB5_5Y1
novel_circ_0008094	-	ENSG00000069248;	CVB5_5Y1
novel_circ_0008161	-	ENSG00000135749;	CVB5_5Y1
novel_circ_0008166	+	ENSG00000143674;	CVB5_5Y1
novel_circ_0008192	-	ENSG00000162885;	CVB5_5Y1
novel_circ_0008219	-	ENSG00000091483;	CVB5_5Y1
novel_circ_0008465	-	ENSG00000134697;	CVB5_5Y1
novel_circ_0008575	-	ENSG00000159658;	CVB5_5Y1
novel_circ_0008730	+	ENSG00000132849;	CVB5_5Y1
novel_circ_0008871	+	ENSG00000116288;	CVB5_5Y1
novel_circ_0009037	-	ENSG00000171603;	CVB5_5Y1
novel_circ_0017867	+	ENSG00000157693;	CVB5_5Y1
novel_circ_0017928	-	ENSG00000119522;	CVB5_5Y1
novel_circ_0018093	-	ENSG00000127191;	CVB5_5Y1
novel_circ_0018148	+	ENSG00000137145;	CVB5_5Y1
novel_circ_0018450	+	ENSG00000135040;	CVB5_5Y1
novel_circ_0018528	-	ENSG00000158169;	CVB5_5Y1
novel_circ_0018547	+	ENSG00000285269;ENSG0	CVB5_5Y1
novel_circ_0018559	+	ENSG00000130956;	CVB5_5Y1
novel_circ_0017042	-	ENSG00000066827;	CVB5_5Y1
novel_circ_0017119	-	ENSG00000169398;	CVB5_5Y1
novel_circ_0017279	+	ENSG00000104660;	CVB5_5Y1
novel_circ_0017294	+	ENSG00000104691;	CVB5_5Y1
novel_circ_0017504	+	ENSG00000104218;	CVB5_5Y1
novel_circ_0017595	-	ENSG00000176623;	CVB5_5Y1
novel_circ_0017619	+	n/a	CVB5_5Y1
novel_circ_0017646	+	ENSG00000164953;	CVB5_5Y1
hsa_circ_0026285	+	ENSG00000123268;	CVB5_5Y1,Con_5Y1
hsa_circ_0007689	+	ENSG00000166224;	CVB5_5Y1,Con_5Y1
hsa_circ_0041411	-	ENSG00000127804;	CVB5_5Y1,Con_5Y1
hsa_circ_0041694	-	ENSG00000108559;	CVB5_5Y1,Con_5Y1
hsa_circ_0044709	+	ENSG00000153933;	CVB5_5Y1,Con_5Y1
hsa_circ_0038696	+	ENSG00000205629;	CVB5_5Y1,Con_5Y1
hsa_circ_0037561	+	ENSG00000167978;	CVB5_5Y1,Con_5Y1
hsa_circ_0003564	+	ENSG00000103351;	CVB5_5Y1,Con_5Y1
hsa_circ_0005559	-	ENSG00000169410;	CVB5_5Y1,Con_5Y1
hsa_circ_0003512	+	ENSG00000140153;	CVB5_5Y1,Con_5Y1
hsa_circ_0051428	+	ENSG00000104856;	CVB5_5Y1,Con_5Y1
hsa_circ_0082304	+	ENSG00000106459;	CVB5_5Y1,Con_5Y1
hsa_circ_0003649	+	ENSG00000009335;	CVB5_5Y1,Con_5Y1
hsa_circ_0079607	-	ENSG00000164548;	CVB5_5Y1,Con_5Y1
hsa_circ_0005004	+	ENSG00000105953;	CVB5_5Y1,Con_5Y1
hsa_circ_0004826	+	ENSG00000152818;	CVB5_5Y1,Con_5Y1
hsa_circ_0075504	-	ENSG00000137266;	CVB5_5Y1,Con_5Y1
hsa_circ_0074736	-	ENSG00000082516;	CVB5_5Y1,Con_5Y1
hsa_circ_0004473	+	ENSG00000165671;	CVB5_5Y1,Con_5Y1
hsa_circ_0066814	-	ENSG00000163611;ENSG0	CVB5_5Y1,Con_5Y1
hsa_circ_0064340	+	ENSG00000154743;	CVB5_5Y1,Con_5Y1
hsa_circ_0057130	-	ENSG00000138430;	CVB5_5Y1,Con_5Y1
hsa_circ_0054178	-	ENSG00000115904;	CVB5_5Y1,Con_5Y1
hsa_circ_0015980	+	ENSG00000077157;	CVB5_5Y1,Con_5Y1
hsa_circ_0088474	-	ENSG00000119522;	CVB5_5Y1,Con_5Y1
hsa_circ_0002883	-	ENSG00000136935;	CVB5_5Y1,Con_5Y1
hsa_circ_0089195	+	ENSG00000130723;	CVB5_5Y1,Con_5Y1
hsa_circ_0089372	+	ENSG00000160323;	CVB5_5Y1,Con_5Y1
hsa_circ_0084193	+	ENSG00000164808;	CVB5_5Y1,Con_5Y1

novel_circ_0002891	+	ENSG00000032742;	CVB5_5Y1,Con_5Y1
novel_circ_0003213	-	ENSG00000125257;	CVB5_5Y1,Con_5Y1
novel_circ_0002127	+	ENSG00000130783;	CVB5_5Y1,Con_5Y1
novel_circ_0001342	-	ENSG00000166016;	CVB5_5Y1,Con_5Y1
novel_circ_0001447	-	ENSG00000030066;	CVB5_5Y1,Con_5Y1
novel_circ_0001537	+	ENSG00000173715;	CVB5_5Y1,Con_5Y1
novel_circ_0000509	-	ENSG00000165322;	CVB5_5Y1,Con_5Y1
novel_circ_0000514	-	ENSG00000170759;	CVB5_5Y1,Con_5Y1
novel_circ_0000924	-	ENSG00000138119;	CVB5_5Y1,Con_5Y1
novel_circ_0004737	-	ENSG00000167969;	CVB5_5Y1,Con_5Y1
novel_circ_0006650	+	ENSG00000065989;	CVB5_5Y1,Con_5Y1
novel_circ_0007024	-	ENSG00000105355;	CVB5_5Y1,Con_5Y1
novel_circ_0009479	+	ENSG00000101161;	CVB5_5Y1,Con_5Y1
novel_circ_0016651	+	ENSG00000187257;	CVB5_5Y1,Con_5Y1
novel_circ_0013579	+	n/a	CVB5_5Y1,Con_5Y1
novel_circ_0012456	-	ENSG00000163961;	CVB5_5Y1,Con_5Y1
novel_circ_0012714	-	ENSG00000163814;	CVB5_5Y1,Con_5Y1
novel_circ_0010820	-	ENSG00000152056;ENSG0	CVB5_5Y1,Con_5Y1
novel_circ_0011118	+	ENSG00000115760;	CVB5_5Y1,Con_5Y1
novel_circ_0011258	-	ENSG00000115970;	CVB5_5Y1,Con_5Y1
novel_circ_0011265	+	ENSG00000152527;	CVB5_5Y1,Con_5Y1
novel_circ_0007287	+	ENSG00000198162;	CVB5_5Y1,Con_5Y1
novel_circ_0008251	-	ENSG00000153187;	CVB5_5Y1,Con_5Y1
novel_circ_0008795	+	ENSG00000198160;	CVB5_5Y1,Con_5Y1
novel_circ_0017890	+	ENSG00000136848;	CVB5_5Y1,Con_5Y1
novel_circ_0018139	+	ENSG00000044459;	CVB5_5Y1,Con_5Y1
novel_circ_0018322	+	ENSG00000137054;	CVB5_5Y1,Con_5Y1
novel_circ_0016858	-	ENSG00000164933;ENSG0	CVB5_5Y1,Con_5Y1
novel_circ_0017307	-	ENSG00000172728;	CVB5_5Y1,Con_5Y1
hsa_circ_0005534	+	ENSG00000082805;	CVB5_5Y1,Con_5Y1,
hsa_circ_0024251	+	ENSG00000178105;	CVB5_5Y1,Con_5Y1,
hsa_circ_0002778	-	ENSG00000166788;	CVB5_5Y1,Con_5Y1,
hsa_circ_0003970	+	ENSG00000150760;	CVB5_5Y1,Con_5Y1,
hsa_circ_0007503	+	ENSG00000095787;	CVB5_5Y1,Con_5Y1,
hsa_circ_0002637	-	ENSG00000197879;	CVB5_5Y1,Con_5Y1,
hsa_circ_0043898	-	ENSG00000108799;	CVB5_5Y1,Con_5Y1,
hsa_circ_0000786	+	ENSG00000153933;	CVB5_5Y1,Con_5Y1,
hsa_circ_0051401	+	ENSG00000142273;	CVB5_5Y1,Con_5Y1,
hsa_circ_0003912	-	ENSG00000105516;	CVB5_5Y1,Con_5Y1,
hsa_circ_0061776	+	ENSG00000182240;	CVB5_5Y1,Con_5Y1,
hsa_circ_0079431	+	ENSG00000106443;	CVB5_5Y1,Con_5Y1,
hsa_circ_0003027	+	ENSG00000133612;	CVB5_5Y1,Con_5Y1,
hsa_circ_0078373	+	ENSG00000213079;	CVB5_5Y1,Con_5Y1,
hsa_circ_0002906	+	ENSG00000024048;	CVB5_5Y1,Con_5Y1,
hsa_circ_0001610	-	ENSG00000146072;	CVB5_5Y1,Con_5Y1,
hsa_circ_0074104	+	ENSG00000120733;	CVB5_5Y1,Con_5Y1,
hsa_circ_0058988	-	ENSG00000115687;	CVB5_5Y1,Con_5Y1,
hsa_circ_0012344	-	ENSG00000079277;	CVB5_5Y1,Con_5Y1,
hsa_circ_0001856	-	ENSG00000137075;	CVB5_5Y1,Con_5Y1,
hsa_circ_0008842	-	ENSG00000165244;	CVB5_5Y1,Con_5Y1,
hsa_circ_0006863	+	ENSG00000104671;	CVB5_5Y1,Con_5Y1,
novel_circ_0002389	+	ENSG00000120647;	CVB5_5Y1,Con_5Y1,
novel_circ_0002599	-	ENSG00000285238;ENSG0	CVB5_5Y1,Con_5Y1,
novel_circ_0001135	+	ENSG00000170242;	CVB5_5Y1,Con_5Y1,
novel_circ_0000970	-	ENSG00000052749;	CVB5_5Y1,Con_5Y1,
novel_circ_0006907	-	ENSG00000245680;	CVB5_5Y1,Con_5Y1,
novel_circ_0011541	-	ENSG00000273398;ENSG0	CVB5_5Y1,Con_5Y1,
novel_circ_0007684	+	ENSG00000162779;	CVB5_5Y1,Con_5Y1,

novel_circ_0018480	-	ENSG00000169071;	CVB5_5Y1,Con_5Y1,
novel_circ_0016855	-	ENSG00000247081;	CVB5_5Y1,Con_5Y1,
hsa_circ_0006128	+	ENSG00000139154;	CVB5_5Y1,Con_5Y1,
hsa_circ_0006599	-	ENSG00000073614;	CVB5_5Y1,Con_5Y1,
hsa_circ_0008802	-	ENSG00000110911;	CVB5_5Y1,Con_5Y1,
hsa_circ_0004282	-	ENSG00000094916;	CVB5_5Y1,Con_5Y1,
hsa_circ_0027261	+	ENSG00000166986;	CVB5_5Y1,Con_5Y1,
hsa_circ_0020203	+	ENSG00000107651;	CVB5_5Y1,Con_5Y1,
hsa_circ_0018054	+	ENSG00000095787;	CVB5_5Y1,Con_5Y1,
hsa_circ_0000706	-	ENSG00000135720;	CVB5_5Y1,Con_5Y1,
hsa_circ_0008926	+	ENSG00000285920;ENSG0	CVB5_5Y1,Con_5Y1,
hsa_circ_0003448	+	ENSG00000258653;ENSG0	CVB5_5Y1,Con_5Y1,
hsa_circ_0049613	+	ENSG00000105613;	CVB5_5Y1,Con_5Y1,
hsa_circ_0008174	+	ENSG00000100393;	CVB5_5Y1,Con_5Y1,
hsa_circ_0003359	-	ENSG00000100425;	CVB5_5Y1,Con_5Y1,
hsa_circ_0082633	-	ENSG00000105939;	CVB5_5Y1,Con_5Y1,
hsa_circ_0081258	+	ENSG00000196367;	CVB5_5Y1,Con_5Y1,
hsa_circ_0077084	+	ENSG00000112701;	CVB5_5Y1,Con_5Y1,
hsa_circ_0072264	-	ENSG00000113569;	CVB5_5Y1,Con_5Y1,
hsa_circ_0070959	-	ENSG00000151466;	CVB5_5Y1,Con_5Y1,
hsa_circ_0001356	+	ENSG00000113810;	CVB5_5Y1,Con_5Y1,
hsa_circ_0005093	-	n/a	CVB5_5Y1,Con_5Y1,
hsa_circ_0065252	-	ENSG00000173473;	CVB5_5Y1,Con_5Y1,
hsa_circ_0066178	-	ENSG00000163931;	CVB5_5Y1,Con_5Y1,
hsa_circ_0058764	+	ENSG00000077044;	CVB5_5Y1,Con_5Y1,
hsa_circ_0004964	+	ENSG00000176407;	CVB5_5Y1,Con_5Y1,
hsa_circ_0008660	+	ENSG00000120948;ENSG0	CVB5_5Y1,Con_5Y1,
hsa_circ_0007167	+	ENSG00000058668;	CVB5_5Y1,Con_5Y1,
hsa_circ_0005886	-	ENSG00000067208;	CVB5_5Y1,Con_5Y1,
hsa_circ_0088095	+	ENSG00000119471;	CVB5_5Y1,Con_5Y1,
hsa_circ_0088425	-	ENSG00000165209;	CVB5_5Y1,Con_5Y1,
hsa_circ_0086536	+	ENSG00000188352;	CVB5_5Y1,Con_5Y1,
novel_circ_0006091	+	ENSG00000087157;	CVB5_5Y1,Con_5Y1,
novel_circ_0006150	+	ENSG00000185298;	CVB5_5Y1,Con_5Y1,
novel_circ_0004680	-	ENSG00000063854;	CVB5_5Y1,Con_5Y1,
novel_circ_0004840	-	ENSG00000005339;	CVB5_5Y1,Con_5Y1,
novel_circ_0005207	+	ENSG00000170100;	CVB5_5Y1,Con_5Y1,
novel_circ_0006764	+	ENSG00000099308;	CVB5_5Y1,Con_5Y1,
novel_circ_0006802	+	ENSG00000160352;	CVB5_5Y1,Con_5Y1,
novel_circ_0009930	-	ENSG00000100271;	CVB5_5Y1,Con_5Y1,
novel_circ_0014165	-	ENSG00000113648;	CVB5_5Y1,Con_5Y1,
novel_circ_0012739	-	ENSG00000181555;	CVB5_5Y1,Con_5Y1,
novel_circ_0010312	-	ENSG00000226383;	CVB5_5Y1,Con_5Y1,
novel_circ_0007747	-	ENSG00000255275;ENSG0	CVB5_5Y1,Con_5Y1,
novel_circ_0008765	-	ENSG00000142856;	CVB5_5Y1,Con_5Y1,
hsa_circ_0030408	+	ENSG00000083535;	CVB5_5Y1,Con_5Y1,
hsa_circ_0008940	+	ENSG00000196935;	CVB5_5Y1,Con_5Y1,
hsa_circ_0000298	-	ENSG00000149182;	CVB5_5Y1,Con_5Y1,
hsa_circ_0022025	-	ENSG00000109919;	CVB5_5Y1,Con_5Y1,
hsa_circ_0008856	+	ENSG00000108219;	CVB5_5Y1,Con_5Y1,
hsa_circ_0036158	-	ENSG00000066933;	CVB5_5Y1,Con_5Y1,
hsa_circ_0049457	-	ENSG00000205517;	CVB5_5Y1,Con_5Y1,
hsa_circ_0007749	+	ENSG00000101773;	CVB5_5Y1,Con_5Y1,
hsa_circ_0008212	+	ENSG00000124226;	CVB5_5Y1,Con_5Y1,
hsa_circ_0077083	+	ENSG00000112701;	CVB5_5Y1,Con_5Y1,
hsa_circ_0003506	+	ENSG00000055163;	CVB5_5Y1,Con_5Y1,
hsa_circ_0067620	-	ENSG00000114127;	CVB5_5Y1,Con_5Y1,
hsa_circ_0006905	+	ENSG00000251503;	CVB5_5Y1,Con_5Y1,

hsa_circ_0002263	+	ENSG00000104218;	CVB5_5Y1,Con_5Y1,
novel_circ_0002097	-	ENSG00000130779;	CVB5_5Y1,Con_5Y1,
novel_circ_0000843	+	ENSG00000107771;	CVB5_5Y1,Con_5Y1,
novel_circ_0009695	+	ENSG00000099968;	CVB5_5Y1,Con_5Y1,
novel_circ_0009757	+	ENSG00000100077;	CVB5_5Y1,Con_5Y1,
novel_circ_0013388	+	ENSG00000109756;	CVB5_5Y1,Con_5Y1,
novel_circ_0013497	-	ENSG00000083857;	CVB5_5Y1,Con_5Y1,
novel_circ_0018456	-	ENSG00000135052;	CVB5_5Y1,Con_5Y1,
hsa_circ_0091427	-	ENSG00000101882;	CVB5_5Y1,Con_5Y2
hsa_circ_0008579	+	ENSG00000198689;	CVB5_5Y1,Con_5Y2
hsa_circ_0089772	+	ENSG00000182378;	CVB5_5Y1,Con_5Y2
hsa_circ_0000495	-	ENSG00000005810;	CVB5_5Y1,Con_5Y2
hsa_circ_0007885	-	ENSG00000089234;	CVB5_5Y1,Con_5Y2
hsa_circ_0025721	+	ENSG00000029153;	CVB5_5Y1,Con_5Y2
hsa_circ_0027629	+	ENSG00000067798;	CVB5_5Y1,Con_5Y2
hsa_circ_0022058	-	ENSG00000030066;	CVB5_5Y1,Con_5Y2
hsa_circ_0022983	-	ENSG00000174669;	CVB5_5Y1,Con_5Y2
hsa_circ_0003206	+	ENSG00000166439;	CVB5_5Y1,Con_5Y2
hsa_circ_0005321	+	ENSG00000065613;	CVB5_5Y1,Con_5Y2
hsa_circ_0002298	+	ENSG00000107651;	CVB5_5Y1,Con_5Y2
hsa_circ_0000236	+	ENSG00000169826;	CVB5_5Y1,Con_5Y2
hsa_circ_0018736	-	ENSG00000138303;	CVB5_5Y1,Con_5Y2
hsa_circ_0004954	-	ENSG00000174238;	CVB5_5Y1,Con_5Y2
hsa_circ_0042474	-	ENSG00000124422;	CVB5_5Y1,Con_5Y2
hsa_circ_0002404	-	ENSG00000278540;	CVB5_5Y1,Con_5Y2
hsa_circ_0043428	-	ENSG00000125686;	CVB5_5Y1,Con_5Y2
hsa_circ_0000679	+	ENSG00000103222;	CVB5_5Y1,Con_5Y2
hsa_circ_0002902	-	ENSG00000168434;	CVB5_5Y1,Con_5Y2
hsa_circ_0040792	-	ENSG00000104731;	CVB5_5Y1,Con_5Y2
hsa_circ_0035294	+	ENSG00000138594;	CVB5_5Y1,Con_5Y2
hsa_circ_0004047	-	ENSG00000198604;	CVB5_5Y1,Con_5Y2
hsa_circ_0002395	-	ENSG00000182400;	CVB5_5Y1,Con_5Y2
hsa_circ_0003819	+	ENSG00000089916;	CVB5_5Y1,Con_5Y2
hsa_circ_0006698	+	ENSG00000007047;	CVB5_5Y1,Con_5Y2
hsa_circ_0002560	+	ENSG00000078142;	CVB5_5Y1,Con_5Y2
hsa_circ_0062157	-	ENSG00000069998;	CVB5_5Y1,Con_5Y2
hsa_circ_0002035	+	ENSG00000101294;	CVB5_5Y1,Con_5Y2
hsa_circ_0060530	+	ENSG00000101109;	CVB5_5Y1,Con_5Y2
hsa_circ_0060922	-	ENSG00000019186;	CVB5_5Y1,Con_5Y2
hsa_circ_0002197	-	ENSG00000159131;	CVB5_5Y1,Con_5Y2
hsa_circ_0003476	+	ENSG00000160218;	CVB5_5Y1,Con_5Y2
hsa_circ_0007639	+	ENSG00000146828;	CVB5_5Y1,Con_5Y2
hsa_circ_0008441	-	ENSG00000243797;	CVB5_5Y1,Con_5Y2
hsa_circ_0082439	+	ENSG00000131558;	CVB5_5Y1,Con_5Y2
hsa_circ_0007762	+	ENSG00000164506;	CVB5_5Y1,Con_5Y2
hsa_circ_0003130	-	ENSG00000184465;	CVB5_5Y1,Con_5Y2
hsa_circ_0075487	+	ENSG00000124588;	CVB5_5Y1,Con_5Y2
hsa_circ_0005913	+	ENSG00000137221;	CVB5_5Y1,Con_5Y2
hsa_circ_0071261	-	ENSG00000109686;	CVB5_5Y1,Con_5Y2
hsa_circ_0003148	-	ENSG00000189308;	CVB5_5Y1,Con_5Y2
hsa_circ_0064388	-	ENSG00000144711;	CVB5_5Y1,Con_5Y2
hsa_circ_0002761	-	ENSG00000073792;	CVB5_5Y1,Con_5Y2
hsa_circ_0006039	-	ENSG00000151276;	CVB5_5Y1,Con_5Y2
hsa_circ_0002401	+	ENSG00000158435;	CVB5_5Y1,Con_5Y2
hsa_circ_0058874	-	ENSG00000132323;	CVB5_5Y1,Con_5Y2
hsa_circ_0058920	-	ENSG00000130414;	CVB5_5Y1,Con_5Y2
hsa_circ_0053786	+	ENSG00000115760;	CVB5_5Y1,Con_5Y2
hsa_circ_0015529	-	ENSG00000135823;	CVB5_5Y1,Con_5Y2

hsa_circ_0000089	+	ENSG00000065243;	CVB5_5Y1,Con_5Y2
hsa_circ_0087932	-	ENSG00000106771;	CVB5_5Y1,Con_5Y2
hsa_circ_0088088	+	ENSG00000119471;	CVB5_5Y1,Con_5Y2
hsa_circ_0005432	+	ENSG00000187742;	CVB5_5Y1,Con_5Y2
hsa_circ_0085187	-	ENSG00000104517;	CVB5_5Y1,Con_5Y2
hsa_circ_0002887	+	ENSG00000078674;	CVB5_5Y1,Con_5Y2
hsa_circ_0005847	+	ENSG00000254673;ENSG0	CVB5_5Y1,Con_5Y2
novel_circ_0018703	+	ENSG00000156531;	CVB5_5Y1,Con_5Y2
novel_circ_0002925	-	ENSG00000027001;	CVB5_5Y1,Con_5Y2
novel_circ_0003261	-	ENSG00000088387;	CVB5_5Y1,Con_5Y2
novel_circ_0002485	+	ENSG00000066084;	CVB5_5Y1,Con_5Y2
novel_circ_0001221	-	ENSG00000080854;	CVB5_5Y1,Con_5Y2
novel_circ_0001549	-	ENSG00000110066;	CVB5_5Y1,Con_5Y2
novel_circ_0001768	+	ENSG00000284057;ENSG0	CVB5_5Y1,Con_5Y2
novel_circ_0005394	-	ENSG00000007202;	CVB5_5Y1,Con_5Y2
novel_circ_0005482	+	ENSG00000270647;	CVB5_5Y1,Con_5Y2
novel_circ_0005697	+	ENSG00000108424;	CVB5_5Y1,Con_5Y2
novel_circ_0005806	-	ENSG00000108395;	CVB5_5Y1,Con_5Y2
novel_circ_0005954	-	ENSG00000108854;	CVB5_5Y1,Con_5Y2
novel_circ_0006018	+	ENSG00000262943;	CVB5_5Y1,Con_5Y2
novel_circ_0005139	-	ENSG00000140943;	CVB5_5Y1,Con_5Y2
novel_circ_0004137	+	ENSG00000171763;	CVB5_5Y1,Con_5Y2
novel_circ_0004160	+	ENSG00000138592;	CVB5_5Y1,Con_5Y2
novel_circ_0004402	+	n/a	CVB5_5Y1,Con_5Y2
novel_circ_0004413	-	ENSG00000169375;	CVB5_5Y1,Con_5Y2
novel_circ_0006715	+	ENSG00000132024;	CVB5_5Y1,Con_5Y2
novel_circ_0009709	+	ENSG00000099917;	CVB5_5Y1,Con_5Y2
novel_circ_0009495	+	ENSG00000155313;	CVB5_5Y1,Con_5Y2
novel_circ_0015822	-	ENSG00000078319;	CVB5_5Y1,Con_5Y2
novel_circ_0016515	-	ENSG00000272693;	CVB5_5Y1,Con_5Y2
novel_circ_0015112	-	ENSG00000135541;	CVB5_5Y1,Con_5Y2
novel_circ_0015337	+	ENSG00000130396;	CVB5_5Y1,Con_5Y2
novel_circ_0015540	-	ENSG00000124571;	CVB5_5Y1,Con_5Y2
novel_circ_0011775	-	ENSG00000138468;	CVB5_5Y1,Con_5Y2
novel_circ_0012222	+	ENSG00000154814;	CVB5_5Y1,Con_5Y2
novel_circ_0012467	-	ENSG00000163975;	CVB5_5Y1,Con_5Y2
novel_circ_0012653	+	ENSG00000144674;	CVB5_5Y1,Con_5Y2
novel_circ_0007530	-	ENSG00000248333;	CVB5_5Y1,Con_5Y2
novel_circ_0007953	-	ENSG00000090686;	CVB5_5Y1,Con_5Y2
novel_circ_0008093	-	ENSG00000069248;	CVB5_5Y1,Con_5Y2
novel_circ_0017500	+	ENSG00000104218;	CVB5_5Y1,Con_5Y2
hsa_circ_0026154	+	ENSG00000123352;	CVB5_5Y1,Con_5Y2,
hsa_circ_0006286	+	ENSG00000137693;	CVB5_5Y1,Con_5Y2,
hsa_circ_0007626	+	ENSG00000165476;	CVB5_5Y1,Con_5Y2,
hsa_circ_0019122	+	ENSG00000095564;	CVB5_5Y1,Con_5Y2,
hsa_circ_0008605	-	ENSG00000070366;	CVB5_5Y1,Con_5Y2,
hsa_circ_0044776	-	ENSG00000285897;ENSG0	CVB5_5Y1,Con_5Y2,
hsa_circ_0045045	-	ENSG00000108506;	CVB5_5Y1,Con_5Y2,
hsa_circ_0008700	+	ENSG00000141076;	CVB5_5Y1,Con_5Y2,
hsa_circ_0002466	-	ENSG00000128881;	CVB5_5Y1,Con_5Y2,
hsa_circ_0000572	-	ENSG00000088808;	CVB5_5Y1,Con_5Y2,
hsa_circ_0032673	-	ENSG00000119638;	CVB5_5Y1,Con_5Y2,
hsa_circ_0008615	-	ENSG00000104881;	CVB5_5Y1,Con_5Y2,
hsa_circ_0048704	+	ENSG00000127663;	CVB5_5Y1,Con_5Y2,
hsa_circ_0003805	-	ENSG00000153339;	CVB5_5Y1,Con_5Y2,
hsa_circ_0062176	-	ENSG00000243156;	CVB5_5Y1,Con_5Y2,
hsa_circ_0001195	-	ENSG00000185658;	CVB5_5Y1,Con_5Y2,
hsa_circ_0005038	-	ENSG00000124587;	CVB5_5Y1,Con_5Y2,

hsa_circ_0005888	+	ENSG00000116731;	CVB5_5Y1,Con_5Y2,
hsa_circ_0083455	+	ENSG00000078674;	CVB5_5Y1,Con_5Y2,
hsa_circ_0002537	+	ENSG00000169139;	CVB5_5Y1,Con_5Y2,
novel_circ_0018931	+	ENSG00000146950;	CVB5_5Y1,Con_5Y2,
novel_circ_0002211	+	ENSG00000061936;	CVB5_5Y1,Con_5Y2,
novel_circ_0005558	-	ENSG00000131747;	CVB5_5Y1,Con_5Y2,
novel_circ_0005801	+	ENSG00000108384;	CVB5_5Y1,Con_5Y2,
novel_circ_0004898	-	ENSG00000102921;	CVB5_5Y1,Con_5Y2,
novel_circ_0005020	+	ENSG00000102908;	CVB5_5Y1,Con_5Y2,
novel_circ_0006645	-	ENSG00000130816;	CVB5_5Y1,Con_5Y2,
novel_circ_0006733	-	ENSG00000141867;	CVB5_5Y1,Con_5Y2,
novel_circ_0013782	+	ENSG00000132405;	CVB5_5Y1,Con_5Y2,
novel_circ_0011950	-	ENSG00000070476;	CVB5_5Y1,Con_5Y2,
novel_circ_0012322	+	ENSG00000274840;	CVB5_5Y1,Con_5Y2,
novel_circ_0011634	+	ENSG00000176407;	CVB5_5Y1,Con_5Y2,
novel_circ_0007607	-	ENSG00000224228;	CVB5_5Y1,Con_5Y2,
novel_circ_0018233	-	ENSG00000137073;	CVB5_5Y1,Con_5Y2,
hsa_circ_0007100	+	ENSG00000130826;	CVB5_5Y1,Con_5Y3
hsa_circ_0006390	+	ENSG00000022840;	CVB5_5Y1,Con_5Y3
hsa_circ_0042330	-	ENSG00000177302;	CVB5_5Y1,Con_5Y3
hsa_circ_0006690	-	ENSG00000108588;	CVB5_5Y1,Con_5Y3
hsa_circ_0037753	-	ENSG00000140632;	CVB5_5Y1,Con_5Y3
hsa_circ_0002604	-	ENSG00000159593;	CVB5_5Y1,Con_5Y3
hsa_circ_0034088	+	ENSG00000273749;	CVB5_5Y1,Con_5Y3
hsa_circ_0004180	+	ENSG00000275835;	CVB5_5Y1,Con_5Y3
hsa_circ_0002551	-	ENSG00000104064;	CVB5_5Y1,Con_5Y3
hsa_circ_0036927	-	ENSG00000198901;ENSG0	CVB5_5Y1,Con_5Y3
hsa_circ_0048171	-	ENSG00000099817;	CVB5_5Y1,Con_5Y3
hsa_circ_0062595	+	ENSG00000099991;	CVB5_5Y1,Con_5Y3
hsa_circ_0006704	-	ENSG00000089006;	CVB5_5Y1,Con_5Y3
hsa_circ_0007544	-	ENSG00000146833;	CVB5_5Y1,Con_5Y3
hsa_circ_0002958	+	ENSG00000145982;	CVB5_5Y1,Con_5Y3
hsa_circ_0008823	-	ENSG00000122203;	CVB5_5Y1,Con_5Y3
hsa_circ_0073127	-	ENSG00000152413;	CVB5_5Y1,Con_5Y3
hsa_circ_0006517	+	ENSG00000145012;	CVB5_5Y1,Con_5Y3
hsa_circ_0054309	-	ENSG00000115970;	CVB5_5Y1,Con_5Y3
hsa_circ_0009205	-	ENSG00000188976;	CVB5_5Y1,Con_5Y3
hsa_circ_0086648	+	ENSG00000086102;	CVB5_5Y1,Con_5Y3
hsa_circ_0005376	+	ENSG00000168172;	CVB5_5Y1,Con_5Y3
novel_circ_0018752	-	ENSG00000224533;ENSG0	CVB5_5Y1,Con_5Y3
novel_circ_0003192	-	ENSG00000005810;	CVB5_5Y1,Con_5Y3
novel_circ_0002117	+	ENSG00000184445;	CVB5_5Y1,Con_5Y3
novel_circ_0002251	+	ENSG00000006831;	CVB5_5Y1,Con_5Y3
novel_circ_0001299	+	ENSG00000169519;	CVB5_5Y1,Con_5Y3
novel_circ_0000942	+	ENSG00000108239;	CVB5_5Y1,Con_5Y3
novel_circ_0004559	+	ENSG00000173575;	CVB5_5Y1,Con_5Y3
novel_circ_0003345	+	ENSG00000131323;	CVB5_5Y1,Con_5Y3
novel_circ_0009756	+	ENSG00000100077;	CVB5_5Y1,Con_5Y3
novel_circ_0015079	-	ENSG00000146376;	CVB5_5Y1,Con_5Y3
novel_circ_0012734	-	ENSG00000181555;	CVB5_5Y1,Con_5Y3
novel_circ_0007777	-	ENSG00000077549;	CVB5_5Y1,Con_5Y3
novel_circ_0017053	-	ENSG00000167632;	CVB5_5Y1,Con_5Y3
novel_circ_0017330	+	ENSG00000168615;	CVB5_5Y1,Con_5Y3
novel_circ_0017606	-	n/a	CVB5_5Y1,Con_5Y3
hsa_circ_0005990	+	ENSG00000137693;	CVB5_5Y1,CVB5_5Y
hsa_circ_0045768	-	ENSG00000167881;	CVB5_5Y1,CVB5_5Y
hsa_circ_0046509	+	ENSG00000141556;	CVB5_5Y1,CVB5_5Y
hsa_circ_0008860	+	ENSG00000138834;	CVB5_5Y1,CVB5_5Y

hsa_circ_0007593	-	ENSG00000064607;	CVB5_5Y1,CVB5_5Y
hsa_circ_0061721	-	ENSG00000185658;	CVB5_5Y1,CVB5_5Y
hsa_circ_0002708	+	ENSG00000006451;	CVB5_5Y1,CVB5_5Y
hsa_circ_0073036	+	ENSG00000113161;	CVB5_5Y1,CVB5_5Y
hsa_circ_0065242	-	ENSG00000173473;	CVB5_5Y1,CVB5_5Y
hsa_circ_0002377	-	ENSG00000138399;	CVB5_5Y1,CVB5_5Y
hsa_circ_0008141	+	ENSG00000152061;	CVB5_5Y1,CVB5_5Y
hsa_circ_0004406	-	ENSG00000078369;	CVB5_5Y1,CVB5_5Y
hsa_circ_0011191	-	ENSG00000116353;	CVB5_5Y1,CVB5_5Y
hsa_circ_0085257	-	ENSG00000104517;	CVB5_5Y1,CVB5_5Y
novel_circ_0000815	-	ENSG00000151657;	CVB5_5Y1,CVB5_5Y
novel_circ_0005293	-	ENSG00000141027;	CVB5_5Y1,CVB5_5Y
novel_circ_0005089	-	ENSG00000090863;	CVB5_5Y1,CVB5_5Y
novel_circ_0009767	+	n/a	CVB5_5Y1,CVB5_5Y
novel_circ_0009478	+	ENSG00000101161;	CVB5_5Y1,CVB5_5Y
novel_circ_0016062	-	ENSG00000006459;	CVB5_5Y1,CVB5_5Y
novel_circ_0015680	+	ENSG00000196586;	CVB5_5Y1,CVB5_5Y
novel_circ_0014617	+	ENSG00000095015;	CVB5_5Y1,CVB5_5Y
novel_circ_0011829	-	ENSG00000285943;ENSG0	CVB5_5Y1,CVB5_5Y
novel_circ_0012163	+	ENSG00000152601;	CVB5_5Y1,CVB5_5Y
novel_circ_0012204	+	ENSG00000174891;	CVB5_5Y1,CVB5_5Y
novel_circ_0012947	+	n/a	CVB5_5Y1,CVB5_5Y
novel_circ_0010356	+	ENSG00000136560;	CVB5_5Y1,CVB5_5Y
novel_circ_0010628	+	ENSG00000055044;	CVB5_5Y1,CVB5_5Y
novel_circ_0007237	-	ENSG00000162777;	CVB5_5Y1,CVB5_5Y
novel_circ_0007528	+	ENSG00000157191;	CVB5_5Y1,CVB5_5Y
novel_circ_0007722	+	ENSG00000135829;	CVB5_5Y1,CVB5_5Y
novel_circ_0008528	-	ENSG00000198815;	CVB5_5Y1,CVB5_5Y
hsa_circ_0001921	-	ENSG00000072501;	CVB5_5Y1,CVB5_5Y
hsa_circ_0002435	-	ENSG00000107864;	CVB5_5Y1,CVB5_5Y
hsa_circ_0007637	-	ENSG00000005339;	CVB5_5Y1,CVB5_5Y
hsa_circ_0006590	+	ENSG00000099331;	CVB5_5Y1,CVB5_5Y
hsa_circ_0048775	-	ENSG00000196365;	CVB5_5Y1,CVB5_5Y
hsa_circ_0060461	+	ENSG00000101057;	CVB5_5Y1,CVB5_5Y
hsa_circ_0004254	-	ENSG00000124201;	CVB5_5Y1,CVB5_5Y
hsa_circ_0004342	+	ENSG00000130396;	CVB5_5Y1,CVB5_5Y
hsa_circ_0075158	+	ENSG00000165671;	CVB5_5Y1,CVB5_5Y
hsa_circ_0002239	+	ENSG00000116288;	CVB5_5Y1,CVB5_5Y
novel_circ_0001424	-	ENSG00000175216;	CVB5_5Y1,CVB5_5Y
novel_circ_0006693	-	ENSG00000196826;	CVB5_5Y1,CVB5_5Y
novel_circ_0009529	-	ENSG00000156299;	CVB5_5Y1,CVB5_5Y
novel_circ_0011413	+	ENSG00000173209;	CVB5_5Y1,CVB5_5Y
novel_circ_0007548	+	ENSG00000143190;	CVB5_5Y1,CVB5_5Y
novel_circ_0008371	-	ENSG00000134644;	CVB5_5Y1,CVB5_5Y
novel_circ_0008997	+	ENSG00000122483;	CVB5_5Y1,CVB5_5Y
novel_circ_0017312	-	ENSG00000104221;	CVB5_5Y1,CVB5_5Y
novel_circ_0017604	-	n/a	CVB5_5Y1,CVB5_5Y
hsa_circ_0005161	+	ENSG00000150403;	CVB5_5Y1,CVB5_5Y
hsa_circ_0022989	+	ENSG00000254986;	CVB5_5Y1,CVB5_5Y
hsa_circ_0019609	-	ENSG00000198408;	CVB5_5Y1,CVB5_5Y
hsa_circ_0006608	+	ENSG00000067057;	CVB5_5Y1,CVB5_5Y
hsa_circ_0008662	-	ENSG00000148719;	CVB5_5Y1,CVB5_5Y
hsa_circ_0044839	+	ENSG00000108406;	CVB5_5Y1,CVB5_5Y
hsa_circ_0007669	+	ENSG00000103064;	CVB5_5Y1,CVB5_5Y
hsa_circ_0003438	-	ENSG00000090861;	CVB5_5Y1,CVB5_5Y
hsa_circ_0006153	-	ENSG00000185024;	CVB5_5Y1,CVB5_5Y
hsa_circ_0031738	-	ENSG00000182400;	CVB5_5Y1,CVB5_5Y
hsa_circ_0031977	-	ENSG00000100523;	CVB5_5Y1,CVB5_5Y

hsa_circ_0005746	-	ENSG00000105063;	CVB5_5Y1,CVB5_5Y
hsa_circ_0003630	-	ENSG00000184708;	CVB5_5Y1,CVB5_5Y
hsa_circ_0003342	+	ENSG00000160218;	CVB5_5Y1,CVB5_5Y
hsa_circ_0079534	-	ENSG00000183742;	CVB5_5Y1,CVB5_5Y
hsa_circ_0008336	+	ENSG00000122507;	CVB5_5Y1,CVB5_5Y
hsa_circ_0004331	+	ENSG00000127955;	CVB5_5Y1,CVB5_5Y
hsa_circ_0077492	-	ENSG00000112249;	CVB5_5Y1,CVB5_5Y
hsa_circ_0076742	-	ENSG00000112118;	CVB5_5Y1,CVB5_5Y
hsa_circ_0002594	-	ENSG00000109381;	CVB5_5Y1,CVB5_5Y
hsa_circ_0001395	+	ENSG00000109805;	CVB5_5Y1,CVB5_5Y
hsa_circ_0065052	-	ENSG00000163812;	CVB5_5Y1,CVB5_5Y
hsa_circ_0008343	+	ENSG00000177479;	CVB5_5Y1,CVB5_5Y
hsa_circ_0004001	-	ENSG00000013441;	CVB5_5Y1,CVB5_5Y
hsa_circ_0053829	+	ENSG00000115760;	CVB5_5Y1,CVB5_5Y
hsa_circ_0000025	-	ENSG00000075151;	CVB5_5Y1,CVB5_5Y
hsa_circ_0011450	+	ENSG00000116497;	CVB5_5Y1,CVB5_5Y
hsa_circ_0088602	-	ENSG00000119487;	CVB5_5Y1,CVB5_5Y
hsa_circ_0008599	-	ENSG00000171889;	CVB5_5Y1,CVB5_5Y
hsa_circ_0085800	-	ENSG00000171045;	CVB5_5Y1,CVB5_5Y
novel_circ_0000877	+	ENSG00000138182;	CVB5_5Y1,CVB5_5Y
novel_circ_0006745	+	ENSG00000072954;	CVB5_5Y1,CVB5_5Y
novel_circ_0006821	-	ENSG00000176619;	CVB5_5Y1,CVB5_5Y
novel_circ_0016434	-	ENSG00000164744;	CVB5_5Y1,CVB5_5Y
novel_circ_0015463	-	ENSG00000196821;	CVB5_5Y1,CVB5_5Y
novel_circ_0014442	+	ENSG00000127022;	CVB5_5Y1,CVB5_5Y
novel_circ_0013552	-	ENSG00000091490;	CVB5_5Y1,CVB5_5Y
novel_circ_0012592	-	ENSG00000144635;	CVB5_5Y1,CVB5_5Y
novel_circ_0012969	-	ENSG00000151276;	CVB5_5Y1,CVB5_5Y
novel_circ_0017241	+	ENSG00000221914;	CVB5_5Y1,CVB5_5Y
hsa_circ_0004372	+	ENSG00000152520;	CVB5_5Y1,CVB5_5Y
hsa_circ_0030253	+	ENSG00000136169;	CVB5_5Y1,CVB5_5Y
hsa_circ_0027700	-	ENSG00000049130;	CVB5_5Y1,CVB5_5Y
hsa_circ_0024231	+	ENSG00000149311;	CVB5_5Y1,CVB5_5Y
hsa_circ_0004075	-	ENSG00000109920;	CVB5_5Y1,CVB5_5Y
hsa_circ_0004162	-	ENSG00000166889;	CVB5_5Y1,CVB5_5Y
hsa_circ_0024043	-	ENSG00000020922;	CVB5_5Y1,CVB5_5Y
hsa_circ_0000258	+	ENSG00000148843;	CVB5_5Y1,CVB5_5Y
hsa_circ_0008311	+	ENSG00000136738;	CVB5_5Y1,CVB5_5Y
hsa_circ_0008655	-	ENSG00000151240;	CVB5_5Y1,CVB5_5Y
hsa_circ_0041252	-	ENSG00000174238;	CVB5_5Y1,CVB5_5Y
hsa_circ_0007736	-	ENSG00000141252;	CVB5_5Y1,CVB5_5Y
hsa_circ_0008099	-	ENSG00000108854;	CVB5_5Y1,CVB5_5Y
hsa_circ_0045526	-	ENSG00000133195;	CVB5_5Y1,CVB5_5Y
hsa_circ_0006719	-	ENSG00000255439;	CVB5_5Y1,CVB5_5Y
hsa_circ_0040188	-	ENSG00000090861;	CVB5_5Y1,CVB5_5Y
hsa_circ_0031485	-	ENSG00000092148;	CVB5_5Y1,CVB5_5Y
hsa_circ_0032406	+	ENSG00000100731;	CVB5_5Y1,CVB5_5Y
hsa_circ_0008521	+	ENSG00000080815;	CVB5_5Y1,CVB5_5Y
hsa_circ_0006446	+	ENSG00000257103;	CVB5_5Y1,CVB5_5Y
hsa_circ_0000942	+	ENSG00000125753;	CVB5_5Y1,CVB5_5Y
hsa_circ_0051749	+	ENSG00000105464;	CVB5_5Y1,CVB5_5Y
hsa_circ_0006400	-	ENSG00000141385;	CVB5_5Y1,CVB5_5Y
hsa_circ_0006209	+	ENSG00000060069;	CVB5_5Y1,CVB5_5Y
hsa_circ_0004547	+	ENSG00000183762;	CVB5_5Y1,CVB5_5Y
hsa_circ_0007513	+	ENSG00000100116;	CVB5_5Y1,CVB5_5Y
hsa_circ_0008324	-	ENSG00000106462;	CVB5_5Y1,CVB5_5Y
hsa_circ_0083196	-	ENSG00000117868;	CVB5_5Y1,CVB5_5Y
hsa_circ_0081024	-	ENSG00000127980;	CVB5_5Y1,CVB5_5Y

hsa_circ_0078224	-	ENSG00000131023;	CVB5_5Y1,CVB5_5Y
hsa_circ_0008403	+	ENSG00000231185;	CVB5_5Y1,CVB5_5Y
hsa_circ_0004098	-	ENSG00000072803;	CVB5_5Y1,CVB5_5Y
hsa_circ_0072380	+	ENSG00000172262;	CVB5_5Y1,CVB5_5Y
hsa_circ_0072546	+	ENSG00000062194;	CVB5_5Y1,CVB5_5Y
hsa_circ_0007692	-	ENSG00000071127;	CVB5_5Y1,CVB5_5Y
hsa_circ_0003673	-	ENSG00000145348;	CVB5_5Y1,CVB5_5Y
hsa_circ_0070421	+	ENSG00000138646;	CVB5_5Y1,CVB5_5Y
hsa_circ_0003113	-	ENSG00000163785;	CVB5_5Y1,CVB5_5Y
hsa_circ_0001349	+	ENSG00000152601;	CVB5_5Y1,CVB5_5Y
hsa_circ_0001274	+	ENSG00000154822;	CVB5_5Y1,CVB5_5Y
hsa_circ_0001965	-	ENSG00000173889;	CVB5_5Y1,CVB5_5Y
hsa_circ_0002569	-	ENSG00000181555;	CVB5_5Y1,CVB5_5Y
hsa_circ_0066452	-	ENSG00000151276;	CVB5_5Y1,CVB5_5Y
hsa_circ_0003789	+	ENSG00000211460;	CVB5_5Y1,CVB5_5Y
hsa_circ_0003994	+	ENSG00000196141;	CVB5_5Y1,CVB5_5Y
hsa_circ_0007381	-	ENSG00000153827;	CVB5_5Y1,CVB5_5Y
hsa_circ_0008944	+	ENSG00000213064;	CVB5_5Y1,CVB5_5Y
hsa_circ_0000196	-	ENSG00000135766;	CVB5_5Y1,CVB5_5Y
hsa_circ_0000042	+	ENSG00000159023;	CVB5_5Y1,CVB5_5Y
hsa_circ_0000076	+	ENSG00000172456;	CVB5_5Y1,CVB5_5Y
hsa_circ_0012782	+	ENSG00000132849;	CVB5_5Y1,CVB5_5Y
hsa_circ_0009007	+	ENSG00000136933;	CVB5_5Y1,CVB5_5Y
hsa_circ_0001897	+	ENSG00000130714;	CVB5_5Y1,CVB5_5Y
hsa_circ_0006459	-	ENSG00000156052;	CVB5_5Y1,CVB5_5Y
hsa_circ_0085434	-	ENSG00000136986;	CVB5_5Y1,CVB5_5Y
hsa_circ_0085459	-	ENSG00000156802;	CVB5_5Y1,CVB5_5Y
hsa_circ_0084746	-	ENSG00000067167;	CVB5_5Y1,CVB5_5Y
novel_circ_0002930	-	ENSG00000102699;	CVB5_5Y1,CVB5_5Y
novel_circ_0002780	+	ENSG00000120802;	CVB5_5Y1,CVB5_5Y
novel_circ_0001460	+	ENSG00000070047;	CVB5_5Y1,CVB5_5Y
novel_circ_0003948	-	ENSG00000140470;	CVB5_5Y1,CVB5_5Y
novel_circ_0006830	+	ENSG00000105176;	CVB5_5Y1,CVB5_5Y
novel_circ_0016381	+	n/a	CVB5_5Y1,CVB5_5Y
novel_circ_0016768	+	ENSG00000164715;	CVB5_5Y1,CVB5_5Y
novel_circ_0015555	+	ENSG00000198087;	CVB5_5Y1,CVB5_5Y
novel_circ_0012466	-	ENSG00000163975;	CVB5_5Y1,CVB5_5Y
novel_circ_0008406	-	ENSG00000134684;	CVB5_5Y1,CVB5_5Y
novel_circ_0018277	-	ENSG00000080298;	CVB5_5Y1,CVB5_5Y
novel_circ_0017553	-	ENSG00000147592;	CVB5_5Y1,CVB5_5Y
hsa_circ_0006903	-	ENSG00000176915;	CVB5_5Y1,CVB5_5Y
hsa_circ_0044968	+	ENSG00000170836;	CVB5_5Y1,CVB5_5Y
hsa_circ_0032047	-	ENSG00000070367;	CVB5_5Y1,CVB5_5Y
hsa_circ_0003271	+	ENSG00000167601;	CVB5_5Y1,CVB5_5Y
hsa_circ_0059802	+	ENSG00000088305;	CVB5_5Y1,CVB5_5Y
hsa_circ_0010402	-	ENSG00000077549;	CVB5_5Y1,CVB5_5Y
hsa_circ_0002318	+	ENSG00000157693;	CVB5_5Y1,CVB5_5Y
novel_circ_0002602	-	ENSG00000111653;	CVB5_5Y1,CVB5_5Y
novel_circ_0009269	+	n/a	CVB5_5Y1,CVB5_5Y
novel_circ_0015498	+	ENSG00000156639;	CVB5_5Y1,CVB5_5Y
novel_circ_0011159	-	ENSG00000171865;	CVB5_5Y1,CVB5_5Y
novel_circ_0011625	-	ENSG00000159374;	CVB5_5Y1,CVB5_5Y
hsa_circ_0030051	-	ENSG00000120690;	CVB5_5Y1,CVB5_5Y
hsa_circ_0007458	+	ENSG00000158636;	CVB5_5Y1,CVB5_5Y
hsa_circ_0004376	-	ENSG00000005100;	CVB5_5Y1,CVB5_5Y
hsa_circ_0007066	-	ENSG00000215041;	CVB5_5Y1,CVB5_5Y
hsa_circ_0045758	-	ENSG00000167880;	CVB5_5Y1,CVB5_5Y
hsa_circ_0046435	+	ENSG00000141568;	CVB5_5Y1,CVB5_5Y

hsa_circ_0004315	-	ENSG00000090863;	CVB5_5Y1,CVB5_5Y
hsa_circ_0007206	-	ENSG00000092439;	CVB5_5Y1,CVB5_5Y
hsa_circ_0008636	-	ENSG00000103591;	CVB5_5Y1,CVB5_5Y
hsa_circ_0004794	-	ENSG00000151748;	CVB5_5Y1,CVB5_5Y
hsa_circ_0047292	-	ENSG00000141447;	CVB5_5Y1,CVB5_5Y
hsa_circ_0004812	-	ENSG00000101004;	CVB5_5Y1,CVB5_5Y
hsa_circ_0008540	+	ENSG00000105819;	CVB5_5Y1,CVB5_5Y
hsa_circ_0006920	+	n/a	CVB5_5Y1,CVB5_5Y
hsa_circ_0073057	-	ENSG00000152359;	CVB5_5Y1,CVB5_5Y
hsa_circ_0065898	-	ENSG00000145041;	CVB5_5Y1,CVB5_5Y
hsa_circ_0064139	+	ENSG00000168137;	CVB5_5Y1,CVB5_5Y
hsa_circ_0003071	+	ENSG00000196141;	CVB5_5Y1,CVB5_5Y
hsa_circ_0012275	-	ENSG00000197429;	CVB5_5Y1,CVB5_5Y
hsa_circ_0006906	-	ENSG00000116171;ENSG0	CVB5_5Y1,CVB5_5Y
hsa_circ_0009143	+	ENSG00000249859;	CVB5_5Y1,CVB5_5Y
hsa_circ_0003134	+	ENSG00000104213;	CVB5_5Y1,CVB5_5Y
novel_circ_0018789	+	ENSG00000130741;	CVB5_5Y1,CVB5_5Y
novel_circ_0002612	+	ENSG00000111581;	CVB5_5Y1,CVB5_5Y
novel_circ_0001606	-	ENSG00000162105;	CVB5_5Y1,CVB5_5Y
novel_circ_0003881	-	ENSG00000165929;	CVB5_5Y1,CVB5_5Y
novel_circ_0014304	-	ENSG00000153395;	CVB5_5Y1,CVB5_5Y
novel_circ_0012628	-	ENSG00000163539;	CVB5_5Y1,CVB5_5Y
hsa_circ_0000345	-	ENSG00000048649;	CVB5_5Y1,CVB5_5Y
hsa_circ_0000345	-	ENSG00000048649;	CVB5_5Y1,CVB5_5Y
hsa_circ_0005637	-	ENSG00000108854;	CVB5_5Y1,CVB5_5Y
hsa_circ_0045534	+	ENSG00000166685;	CVB5_5Y1,CVB5_5Y
hsa_circ_0046447	+	ENSG00000141556;	CVB5_5Y1,CVB5_5Y
hsa_circ_0002255	-	ENSG00000117899;	CVB5_5Y1,CVB5_5Y
hsa_circ_0037002	+	ENSG00000173575;	CVB5_5Y1,CVB5_5Y
hsa_circ_0000926	+	ENSG00000257103;	CVB5_5Y1,CVB5_5Y
hsa_circ_0007940	+	ENSG00000130429;ENSG0	CVB5_5Y1,CVB5_5Y
hsa_circ_0003131	-	ENSG00000112079;	CVB5_5Y1,CVB5_5Y
hsa_circ_0008646	+	ENSG00000038382;	CVB5_5Y1,CVB5_5Y
hsa_circ_0007184	+	ENSG00000138802;	CVB5_5Y1,CVB5_5Y
hsa_circ_0067528	-	ENSG00000051382;	CVB5_5Y1,CVB5_5Y
hsa_circ_0007900	-	ENSG00000135945;	CVB5_5Y1,CVB5_5Y
hsa_circ_0004380	-	ENSG00000167632;	CVB5_5Y1,CVB5_5Y
hsa_circ_0005296	+	ENSG00000168615;	CVB5_5Y1,CVB5_5Y
novel_circ_0001051	+	n/a	CVB5_5Y1,CVB5_5Y
novel_circ_0000729	+	ENSG00000060339;	CVB5_5Y1,CVB5_5Y
novel_circ_0005290	-	ENSG00000141027;	CVB5_5Y1,CVB5_5Y
novel_circ_0005790	-	ENSG00000136450;	CVB5_5Y1,CVB5_5Y
novel_circ_0006071	-	ENSG00000129667;	CVB5_5Y1,CVB5_5Y
novel_circ_0006122	+	ENSG00000141564;	CVB5_5Y1,CVB5_5Y
novel_circ_0004119	+	ENSG00000137770;	CVB5_5Y1,CVB5_5Y
novel_circ_0009925	-	ENSG00000182841;	CVB5_5Y1,CVB5_5Y
novel_circ_0013691	-	ENSG00000145244;	CVB5_5Y1,CVB5_5Y
novel_circ_0013923	+	ENSG00000184305;	CVB5_5Y1,CVB5_5Y
novel_circ_0018521	+	ENSG00000148120;	CVB5_5Y1,CVB5_5Y
novel_circ_0018543	+	ENSG00000182150;	CVB5_5Y1,CVB5_5Y
hsa_circ_0008131	-	ENSG00000167393;	CVB5_5Y1,CVB5_5Y
hsa_circ_0029170	-	ENSG00000090975;	CVB5_5Y1,CVB5_5Y
hsa_circ_0004596	-	ENSG00000175582;	CVB5_5Y1,CVB5_5Y
hsa_circ_0000228	+	ENSG00000148516;	CVB5_5Y1,CVB5_5Y
hsa_circ_0006285	+	ENSG00000138160;	CVB5_5Y1,CVB5_5Y
hsa_circ_0005508	-	ENSG00000174238;	CVB5_5Y1,CVB5_5Y
hsa_circ_0008866	+	ENSG00000160551;	CVB5_5Y1,CVB5_5Y
hsa_circ_0003127	+	ENSG00000062716;	CVB5_5Y1,CVB5_5Y

hsa_circ_0047096	+	ENSG00000101752;	CVB5_5Y1,CVB5_5Y
hsa_circ_0001581	+	ENSG00000165097;	CVB5_5Y1,CVB5_5Y
hsa_circ_0003373	-	ENSG00000118816;	CVB5_5Y1,CVB5_5Y
hsa_circ_0007841	+	ENSG00000058262;	CVB5_5Y1,CVB5_5Y
hsa_circ_0003693	-	ENSG00000186141;	CVB5_5Y1,CVB5_5Y
hsa_circ_0011163	+	ENSG00000162419;	CVB5_5Y1,CVB5_5Y
hsa_circ_0008129	+	ENSG00000117054;	CVB5_5Y1,CVB5_5Y
hsa_circ_0001807	-	ENSG00000066777;	CVB5_5Y1,CVB5_5Y
novel_circ_0013484	+	ENSG00000109762;	CVB5_5Y1,CVB5_5Y
novel_circ_0012830	+	ENSG00000004534;	CVB5_5Y1,CVB5_5Y
novel_circ_0012887	-	ENSG00000163935;	CVB5_5Y1,CVB5_5Y
novel_circ_0013057	-	ENSG00000057019;	CVB5_5Y1,CVB5_5Y
hsa_circ_0006238	+	ENSG00000123352;	CVB5_5Y1,CVB5_5Y
hsa_circ_0005589	+	ENSG00000095139;	CVB5_5Y1,CVB5_5Y
hsa_circ_0002124	+	ENSG00000137804;ENSG0	CVB5_5Y1,CVB5_5Y
hsa_circ_0000604	-	ENSG00000137776;	CVB5_5Y1,CVB5_5Y
hsa_circ_0036287	+	ENSG00000179335;	CVB5_5Y1,CVB5_5Y
hsa_circ_0072482	+	ENSG00000039123;	CVB5_5Y1,CVB5_5Y
hsa_circ_0066459	-	ENSG00000151276;	CVB5_5Y1,CVB5_5Y
hsa_circ_0058182	+	ENSG00000079246;	CVB5_5Y1,CVB5_5Y
hsa_circ_0010444	-	ENSG00000127483;	CVB5_5Y1,CVB5_5Y
hsa_circ_0008650	-	ENSG00000185420;	CVB5_5Y1,CVB5_5Y
hsa_circ_0004005	-	ENSG00000162851;	CVB5_5Y1,CVB5_5Y
hsa_circ_0005249	+	ENSG00000165219;	CVB5_5Y1,CVB5_5Y
hsa_circ_0086748	-	ENSG00000186638;	CVB5_5Y1,CVB5_5Y
novel_circ_0002030	+	n/a	CVB5_5Y1,CVB5_5Y
novel_circ_0001329	-	ENSG00000176102;	CVB5_5Y1,CVB5_5Y
novel_circ_0000235	-	ENSG00000066468;	CVB5_5Y1,CVB5_5Y
novel_circ_0016657	+	ENSG00000127955;	CVB5_5Y1,CVB5_5Y
novel_circ_0017078	-	ENSG00000169398;	CVB5_5Y1,CVB5_5Y
hsa_circ_0028270	-	ENSG00000204842;	CVB5_5Y1,CVB5_5Y
hsa_circ_0005082	-	ENSG00000196498;	CVB5_5Y1,CVB5_5Y
hsa_circ_0005701	+	ENSG00000110108;	CVB5_5Y1,CVB5_5Y
hsa_circ_0002383	-	ENSG00000214530;	CVB5_5Y1,CVB5_5Y
hsa_circ_0019975	+	ENSG00000065621;	CVB5_5Y1,CVB5_5Y
hsa_circ_0040987	+	ENSG00000158805;	CVB5_5Y1,CVB5_5Y
hsa_circ_0007737	+	ENSG00000126214;ENSG0	CVB5_5Y1,CVB5_5Y
hsa_circ_0003312	+	ENSG00000104856;	CVB5_5Y1,CVB5_5Y
hsa_circ_0068843	+	ENSG00000090316;	CVB5_5Y1,CVB5_5Y
hsa_circ_0001846	-	ENSG00000137073;	CVB5_5Y1,CVB5_5Y
hsa_circ_0001866	-	ENSG00000135018;	CVB5_5Y1,CVB5_5Y
hsa_circ_0084051	+	ENSG00000168615;	CVB5_5Y1,CVB5_5Y
novel_circ_0004101	-	ENSG00000067369;	CVB5_5Y1,CVB5_5Y
novel_circ_0003834	-	ENSG00000100629;	CVB5_5Y1,CVB5_5Y
novel_circ_0006718	+	ENSG00000104998;	CVB5_5Y1,CVB5_5Y
novel_circ_0012267	+	ENSG00000075420;	CVB5_5Y1,CVB5_5Y
hsa_circ_0006541	+	ENSG00000132964;	CVB5_5Y1,CVB5_5Y
hsa_circ_0008670	+	ENSG00000256950;ENSG0	CVB5_5Y1,CVB5_5Y
hsa_circ_0029308	-	ENSG00000196498;	CVB5_5Y1,CVB5_5Y
hsa_circ_0000392	-	ENSG00000015153;	CVB5_5Y1,CVB5_5Y
hsa_circ_0006535	+	ENSG00000066117;	CVB5_5Y1,CVB5_5Y
hsa_circ_0008992	-	ENSG00000149269;	CVB5_5Y1,CVB5_5Y
hsa_circ_0020181	-	ENSG00000151923;	CVB5_5Y1,CVB5_5Y
hsa_circ_0004451	+	ENSG00000019995;	CVB5_5Y1,CVB5_5Y
hsa_circ_0003459	-	ENSG00000122912;	CVB5_5Y1,CVB5_5Y
hsa_circ_0003584	+	ENSG00000095564;	CVB5_5Y1,CVB5_5Y
hsa_circ_0042458	-	ENSG00000124422;	CVB5_5Y1,CVB5_5Y
hsa_circ_0041388	-	ENSG00000070366;	CVB5_5Y1,CVB5_5Y

hsa_circ_0038487	+	ENSG00000103319;	CVB5_5Y1,CVB5_5Y
hsa_circ_0006008	+	ENSG00000090905;	CVB5_5Y1,CVB5_5Y
hsa_circ_0008223	-	ENSG00000169180;	CVB5_5Y1,CVB5_5Y
hsa_circ_0039076	+	ENSG00000080603;	CVB5_5Y1,CVB5_5Y
hsa_circ_0006482	+	ENSG00000067955;	CVB5_5Y1,CVB5_5Y
hsa_circ_0007361	-	ENSG00000140948;	CVB5_5Y1,CVB5_5Y
hsa_circ_0037060	-	ENSG00000140470;	CVB5_5Y1,CVB5_5Y
hsa_circ_0008488	+	ENSG00000156958;	CVB5_5Y1,CVB5_5Y
hsa_circ_0004252	+	ENSG00000137807;	CVB5_5Y1,CVB5_5Y
hsa_circ_0004266	+	ENSG00000137807;	CVB5_5Y1,CVB5_5Y
hsa_circ_0036354	-	ENSG00000169375;	CVB5_5Y1,CVB5_5Y
hsa_circ_0036763	+	ENSG00000185033;	CVB5_5Y1,CVB5_5Y
hsa_circ_0000660	+	ENSG00000140563;	CVB5_5Y1,CVB5_5Y
hsa_circ_0000557	-	ENSG00000119638;	CVB5_5Y1,CVB5_5Y
hsa_circ_0049356	+	ENSG00000142453;	CVB5_5Y1,CVB5_5Y
hsa_circ_0050336	+	ENSG00000105176;	CVB5_5Y1,CVB5_5Y
hsa_circ_0003356	+	ENSG00000011485;	CVB5_5Y1,CVB5_5Y
hsa_circ_0005175	-	ENSG00000100099;	CVB5_5Y1,CVB5_5Y
hsa_circ_0001235	-	ENSG00000100401;	CVB5_5Y1,CVB5_5Y
hsa_circ_0004580	-	ENSG00000089123;	CVB5_5Y1,CVB5_5Y
hsa_circ_0009098	+	ENSG00000088325;	CVB5_5Y1,CVB5_5Y
hsa_circ_0002715	+	ENSG00000160299;	CVB5_5Y1,CVB5_5Y
hsa_circ_0007518	+	ENSG00000090266;	CVB5_5Y1,CVB5_5Y
hsa_circ_0003203	-	ENSG00000126524;	CVB5_5Y1,CVB5_5Y
hsa_circ_0005214	-	ENSG00000135541;	CVB5_5Y1,CVB5_5Y
hsa_circ_0006835	-	ENSG00000135540;	CVB5_5Y1,CVB5_5Y
hsa_circ_0076125	+	ENSG00000023892;	CVB5_5Y1,CVB5_5Y
hsa_circ_0001599	-	ENSG00000096060;	CVB5_5Y1,CVB5_5Y
hsa_circ_0077078	+	ENSG00000112701;	CVB5_5Y1,CVB5_5Y
hsa_circ_0075157	+	ENSG00000165671;	CVB5_5Y1,CVB5_5Y
hsa_circ_0073027	-	ENSG00000198780;	CVB5_5Y1,CVB5_5Y
hsa_circ_0006916	-	ENSG00000152413;	CVB5_5Y1,CVB5_5Y
hsa_circ_0073276	+	ENSG00000113356;	CVB5_5Y1,CVB5_5Y
hsa_circ_0009039	-	ENSG00000083857;	CVB5_5Y1,CVB5_5Y
hsa_circ_0002194	-	ENSG00000181826;	CVB5_5Y1,CVB5_5Y
hsa_circ_0006884	+	ENSG00000113845;	CVB5_5Y1,CVB5_5Y
hsa_circ_0006619	+	ENSG00000075975;	CVB5_5Y1,CVB5_5Y
hsa_circ_0005414	+	ENSG00000153551;	CVB5_5Y1,CVB5_5Y
hsa_circ_0001308	-	ENSG00000114904;	CVB5_5Y1,CVB5_5Y
hsa_circ_0008027	+	ENSG00000114026;	CVB5_5Y1,CVB5_5Y
hsa_circ_0066608	+	ENSG00000064225;	CVB5_5Y1,CVB5_5Y
hsa_circ_0004476	-	ENSG00000143970;	CVB5_5Y1,CVB5_5Y
hsa_circ_0007793	+	ENSG00000162929;	CVB5_5Y1,CVB5_5Y
hsa_circ_0015211	+	ENSG00000117523;	CVB5_5Y1,CVB5_5Y
hsa_circ_0015211	+	ENSG00000117523;	CVB5_5Y1,CVB5_5Y
hsa_circ_0003914	-	ENSG00000143514;	CVB5_5Y1,CVB5_5Y
hsa_circ_0017041	-	ENSG00000054267;ENSG0	CVB5_5Y1,CVB5_5Y
hsa_circ_0003757	-	ENSG00000117305;	CVB5_5Y1,CVB5_5Y
hsa_circ_0000073	-	ENSG00000162600;	CVB5_5Y1,CVB5_5Y
hsa_circ_0087960	-	ENSG00000198121;	CVB5_5Y1,CVB5_5Y
hsa_circ_0003496	-	ENSG00000137073;	CVB5_5Y1,CVB5_5Y
novel_circ_0018839	+	n/a	CVB5_5Y1,CVB5_5Y
novel_circ_0002972	-	ENSG00000120694;	CVB5_5Y1,CVB5_5Y
novel_circ_0002192	-	ENSG00000150990;	CVB5_5Y1,CVB5_5Y
novel_circ_0002530	-	ENSG00000139613;	CVB5_5Y1,CVB5_5Y
novel_circ_0005542	+	ENSG00000167258;	CVB5_5Y1,CVB5_5Y
novel_circ_0005742	-	ENSG00000011258;	CVB5_5Y1,CVB5_5Y
novel_circ_0005181	+	ENSG00000179588;	CVB5_5Y1,CVB5_5Y

novel_circ_0006708	-	ENSG00000179271;	CVB5_5Y1,CVB5_5Y
novel_circ_0006835	-	ENSG00000105556;	CVB5_5Y1,CVB5_5Y
novel_circ_0006283	-	ENSG00000177150;	CVB5_5Y1,CVB5_5Y
novel_circ_0009814	-	ENSG00000241878;	CVB5_5Y1,CVB5_5Y
novel_circ_0009973	-	ENSG00000075275;	CVB5_5Y1,CVB5_5Y
novel_circ_0009271	+	n/a	CVB5_5Y1,CVB5_5Y
novel_circ_0013250	+	ENSG00000164134;	CVB5_5Y1,CVB5_5Y
novel_circ_0012386	+	ENSG00000163904;	CVB5_5Y1,CVB5_5Y
novel_circ_0010546	+	ENSG00000064933;	CVB5_5Y1,CVB5_5Y
novel_circ_0018282	+	ENSG00000198722;	CVB5_5Y1,CVB5_5Y
novel_circ_0016990	-	ENSG00000254166;	CVB5_5Y1,CVB5_5Y
hsa_circ_0006020	+	ENSG00000086712;	CVB5_5Y1,CVB5_5Y
hsa_circ_0008077	+	ENSG00000130962;	CVB5_5Y1,CVB5_5Y
hsa_circ_0007290	-	ENSG00000069509;	CVB5_5Y1,CVB5_5Y
hsa_circ_0007817	-	ENSG00000086758;	CVB5_5Y1,CVB5_5Y
hsa_circ_0091178	-	ENSG00000165288;	CVB5_5Y1,CVB5_5Y
hsa_circ_0029614	-	ENSG00000121390;	CVB5_5Y1,CVB5_5Y
hsa_circ_0003285	-	ENSG00000150456;	CVB5_5Y1,CVB5_5Y
hsa_circ_0003489	+	ENSG00000132964;	CVB5_5Y1,CVB5_5Y
hsa_circ_0006597	+	ENSG00000152520;	CVB5_5Y1,CVB5_5Y
hsa_circ_0030720	+	ENSG00000134882;	CVB5_5Y1,CVB5_5Y
hsa_circ_0002601	-	ENSG00000204842;	CVB5_5Y1,CVB5_5Y
hsa_circ_0028601	+	ENSG00000135119;	CVB5_5Y1,CVB5_5Y
hsa_circ_0028899	+	ENSG00000022840;	CVB5_5Y1,CVB5_5Y
hsa_circ_0000450	-	ENSG00000139725;	CVB5_5Y1,CVB5_5Y
hsa_circ_0007708	+	ENSG00000130783;	CVB5_5Y1,CVB5_5Y
hsa_circ_0007552	-	ENSG00000188026;	CVB5_5Y1,CVB5_5Y
hsa_circ_0000458	+	ENSG00000086598;	CVB5_5Y1,CVB5_5Y
hsa_circ_0000462	-	ENSG00000139370;	CVB5_5Y1,CVB5_5Y
hsa_circ_0004602	-	ENSG00000139636;	CVB5_5Y1,CVB5_5Y
hsa_circ_0026230	+	ENSG00000161813;	CVB5_5Y1,CVB5_5Y
hsa_circ_0000408	-	ENSG00000111602;	CVB5_5Y1,CVB5_5Y
hsa_circ_0007845	-	ENSG00000089693;	CVB5_5Y1,CVB5_5Y
hsa_circ_0027492	+	ENSG00000135679;	CVB5_5Y1,CVB5_5Y
hsa_circ_0027699	-	ENSG00000049130;	CVB5_5Y1,CVB5_5Y
hsa_circ_0006868	-	ENSG00000152404;	CVB5_5Y1,CVB5_5Y
hsa_circ_0024169	+	ENSG00000166266;	CVB5_5Y1,CVB5_5Y
hsa_circ_0004962	+	ENSG00000168092;	CVB5_5Y1,CVB5_5Y
hsa_circ_0020976	-	ENSG00000167355;	CVB5_5Y1,CVB5_5Y
hsa_circ_0008338	+	ENSG00000149054;	CVB5_5Y1,CVB5_5Y
hsa_circ_0006705	+	ENSG00000166439;	CVB5_5Y1,CVB5_5Y
hsa_circ_0000343	-	ENSG00000074201;	CVB5_5Y1,CVB5_5Y
hsa_circ_0023942	-	ENSG00000073921;	CVB5_5Y1,CVB5_5Y
hsa_circ_0024037	-	ENSG00000020922;	CVB5_5Y1,CVB5_5Y
hsa_circ_0024067	+	ENSG00000166037;	CVB5_5Y1,CVB5_5Y
hsa_circ_0000255	-	ENSG00000107829;	CVB5_5Y1,CVB5_5Y
hsa_circ_0000257	+	ENSG00000166197;	CVB5_5Y1,CVB5_5Y
hsa_circ_0003366	+	ENSG00000148843;	CVB5_5Y1,CVB5_5Y
hsa_circ_0000259	+	ENSG00000065613;	CVB5_5Y1,CVB5_5Y
hsa_circ_0002533	+	ENSG00000151532;	CVB5_5Y1,CVB5_5Y
hsa_circ_0020050	+	ENSG00000148737;	CVB5_5Y1,CVB5_5Y
hsa_circ_0020072	-	ENSG00000099204;	CVB5_5Y1,CVB5_5Y
hsa_circ_0020134	-	ENSG00000151893;	CVB5_5Y1,CVB5_5Y
hsa_circ_0004498	-	ENSG00000151923;	CVB5_5Y1,CVB5_5Y
hsa_circ_0007448	-	ENSG00000151461;	CVB5_5Y1,CVB5_5Y
hsa_circ_0020238	-	ENSG00000066468;	CVB5_5Y1,CVB5_5Y
hsa_circ_0005734	-	ENSG00000258539;ENSG0	CVB5_5Y1,CVB5_5Y
hsa_circ_0008969	-	ENSG00000203791;ENSG0	CVB5_5Y1,CVB5_5Y

hsa_circ_0004477	+	ENSG00000065328;	CVB5_5Y1,CVB5_5Y
hsa_circ_0002618	-	ENSG00000136770;	CVB5_5Y1,CVB5_5Y
hsa_circ_0004306	-	ENSG00000136770;	CVB5_5Y1,CVB5_5Y
hsa_circ_0003583	-	ENSG00000150867;	CVB5_5Y1,CVB5_5Y
hsa_circ_0018009	-	ENSG00000107890;	CVB5_5Y1,CVB5_5Y
hsa_circ_0018168	-	ENSG00000148498;	CVB5_5Y1,CVB5_5Y
hsa_circ_0006272	+	ENSG00000060339;	CVB5_5Y1,CVB5_5Y
hsa_circ_0008865	+	ENSG00000165732;	CVB5_5Y1,CVB5_5Y
hsa_circ_0017412	-	ENSG00000107929;	CVB5_5Y1,CVB5_5Y
hsa_circ_0019079	+	ENSG00000138182;	CVB5_5Y1,CVB5_5Y
hsa_circ_0005801	-	ENSG00000077147;	CVB5_5Y1,CVB5_5Y
hsa_circ_0005887	-	ENSG00000171311;	CVB5_5Y1,CVB5_5Y
hsa_circ_0008996	+	ENSG00000065559;	CVB5_5Y1,CVB5_5Y
hsa_circ_0004142	+	ENSG00000091542;	CVB5_5Y1,CVB5_5Y
hsa_circ_0005946	+	ENSG00000132383;	CVB5_5Y1,CVB5_5Y
hsa_circ_0002167	+	ENSG00000108587;	CVB5_5Y1,CVB5_5Y
hsa_circ_0004751	+	ENSG00000275700;	CVB5_5Y1,CVB5_5Y
hsa_circ_0000759	-	ENSG00000278540;	CVB5_5Y1,CVB5_5Y
hsa_circ_0007635	+	ENSG00000131467;	CVB5_5Y1,CVB5_5Y
hsa_circ_0004622	+	ENSG00000141279;	CVB5_5Y1,CVB5_5Y
hsa_circ_0005600	+	ENSG00000175155;	CVB5_5Y1,CVB5_5Y
hsa_circ_0004958	+	ENSG00000170836;	CVB5_5Y1,CVB5_5Y
hsa_circ_0007682	+	ENSG00000141376;	CVB5_5Y1,CVB5_5Y
hsa_circ_0004913	-	ENSG00000136478;	CVB5_5Y1,CVB5_5Y
hsa_circ_0005347	+	ENSG00000171634;	CVB5_5Y1,CVB5_5Y
hsa_circ_0000741	+	ENSG00000181222;	CVB5_5Y1,CVB5_5Y
hsa_circ_0003684	-	ENSG00000125447;	CVB5_5Y1,CVB5_5Y
hsa_circ_0006799	-	ENSG00000129646;	CVB5_5Y1,CVB5_5Y
hsa_circ_0008114	+	ENSG00000167280;	CVB5_5Y1,CVB5_5Y
hsa_circ_0041986	+	ENSG00000170037;	CVB5_5Y1,CVB5_5Y
hsa_circ_0008310	+	ENSG00000170037;	CVB5_5Y1,CVB5_5Y
hsa_circ_0000813	+	ENSG00000141564;	CVB5_5Y1,CVB5_5Y
hsa_circ_0046123	-	ENSG00000157637;	CVB5_5Y1,CVB5_5Y
hsa_circ_0046209	-	ENSG00000182446;	CVB5_5Y1,CVB5_5Y
hsa_circ_0000673	-	ENSG00000171490;	CVB5_5Y1,CVB5_5Y
hsa_circ_0008120	+	ENSG00000103222;	CVB5_5Y1,CVB5_5Y
hsa_circ_0000688	+	ENSG00000013364;	CVB5_5Y1,CVB5_5Y
hsa_circ_0008535	-	ENSG00000174938;	CVB5_5Y1,CVB5_5Y
hsa_circ_0037158	-	ENSG00000103126;	CVB5_5Y1,CVB5_5Y
hsa_circ_0007788	-	ENSG00000153406;	CVB5_5Y1,CVB5_5Y
hsa_circ_0005813	+	ENSG00000102910;	CVB5_5Y1,CVB5_5Y
hsa_circ_0000705	-	ENSG00000125107;	CVB5_5Y1,CVB5_5Y
hsa_circ_0002122	+	ENSG00000102974;	CVB5_5Y1,CVB5_5Y
hsa_circ_0006845	+	ENSG00000102908;	CVB5_5Y1,CVB5_5Y
hsa_circ_0004983	-	ENSG00000181019;	CVB5_5Y1,CVB5_5Y
hsa_circ_0009163	+	ENSG00000189091;	CVB5_5Y1,CVB5_5Y
hsa_circ_0006173	+	n/a	CVB5_5Y1,CVB5_5Y
hsa_circ_0003315	-	ENSG00000090863;	CVB5_5Y1,CVB5_5Y
hsa_circ_0004910	-	ENSG00000103091;	CVB5_5Y1,CVB5_5Y
hsa_circ_0000669	-	ENSG00000153048;	CVB5_5Y1,CVB5_5Y
hsa_circ_0004640	+	ENSG00000158545;	CVB5_5Y1,CVB5_5Y
hsa_circ_0040831	+	ENSG00000158545;	CVB5_5Y1,CVB5_5Y
hsa_circ_0005152	-	ENSG00000187555;	CVB5_5Y1,CVB5_5Y
hsa_circ_0004344	-	ENSG00000187555;	CVB5_5Y1,CVB5_5Y
hsa_circ_0003846	-	ENSG00000167522;	CVB5_5Y1,CVB5_5Y
hsa_circ_0040929	-	ENSG00000167522;	CVB5_5Y1,CVB5_5Y
hsa_circ_0004945	+	ENSG00000197912;	CVB5_5Y1,CVB5_5Y
hsa_circ_0037096	-	ENSG00000140479;	CVB5_5Y1,CVB5_5Y

hsa_circ_0034294	-	ENSG00000104067;	CVB5_5Y1,CVB5_5Y
hsa_circ_0004942	-	ENSG00000137824;	CVB5_5Y1,CVB5_5Y
hsa_circ_0004279	-	ENSG00000128908;	CVB5_5Y1,CVB5_5Y
hsa_circ_0008346	+	ENSG00000285920;ENSG0	CVB5_5Y1,CVB5_5Y
hsa_circ_0000596	+	ENSG00000166734;	CVB5_5Y1,CVB5_5Y
hsa_circ_0035292	+	ENSG00000138594;	CVB5_5Y1,CVB5_5Y
hsa_circ_0003713	-	ENSG00000137776;	CVB5_5Y1,CVB5_5Y
hsa_circ_0008104	+	ENSG00000140455;	CVB5_5Y1,CVB5_5Y
hsa_circ_0000612	+	ENSG00000140455;	CVB5_5Y1,CVB5_5Y
hsa_circ_0035796	-	ENSG00000103657;	CVB5_5Y1,CVB5_5Y
hsa_circ_0004374	-	ENSG00000166855;	CVB5_5Y1,CVB5_5Y
hsa_circ_0004401	-	ENSG00000179151;	CVB5_5Y1,CVB5_5Y
hsa_circ_0003620	-	ENSG00000140374;	CVB5_5Y1,CVB5_5Y
hsa_circ_0005558	-	ENSG00000117899;	CVB5_5Y1,CVB5_5Y
hsa_circ_0036599	-	ENSG00000140612;	CVB5_5Y1,CVB5_5Y
hsa_circ_0007262	+	ENSG00000173575;ENSG0	CVB5_5Y1,CVB5_5Y
hsa_circ_0000655	+	ENSG00000173575;	CVB5_5Y1,CVB5_5Y
hsa_circ_0036997	+	ENSG00000173575;	CVB5_5Y1,CVB5_5Y
hsa_circ_0037000	+	ENSG00000173575;	CVB5_5Y1,CVB5_5Y
hsa_circ_0006809	-	ENSG00000198752;	CVB5_5Y1,CVB5_5Y
hsa_circ_0005721	-	ENSG00000129566;	CVB5_5Y1,CVB5_5Y
hsa_circ_0031241	-	ENSG00000100461;	CVB5_5Y1,CVB5_5Y
hsa_circ_0000524	-	ENSG00000100461;	CVB5_5Y1,CVB5_5Y
hsa_circ_0031447	-	ENSG00000196792;	CVB5_5Y1,CVB5_5Y
hsa_circ_0000530	+	ENSG00000150527;	CVB5_5Y1,CVB5_5Y
hsa_circ_0000539	-	ENSG00000126787;	CVB5_5Y1,CVB5_5Y
hsa_circ_0032825	-	ENSG00000100629;	CVB5_5Y1,CVB5_5Y
hsa_circ_0007715	+	ENSG00000099622;	CVB5_5Y1,CVB5_5Y
hsa_circ_0000915	-	ENSG00000105701;	CVB5_5Y1,CVB5_5Y
hsa_circ_0006804	+	ENSG00000167491;	CVB5_5Y1,CVB5_5Y
hsa_circ_0007328	+	ENSG00000104885;	CVB5_5Y1,CVB5_5Y
hsa_circ_0000921	+	ENSG00000105176;	CVB5_5Y1,CVB5_5Y
hsa_circ_0002661	-	ENSG00000105298;	CVB5_5Y1,CVB5_5Y
hsa_circ_0000871	-	ENSG00000186111;	CVB5_5Y1,CVB5_5Y
hsa_circ_0006670	+	ENSG00000105738;	CVB5_5Y1,CVB5_5Y
hsa_circ_0000936	+	ENSG00000160410;	CVB5_5Y1,CVB5_5Y
hsa_circ_0000944	+	ENSG00000105321;	CVB5_5Y1,CVB5_5Y
hsa_circ_0002084	+	ENSG00000104805;	CVB5_5Y1,CVB5_5Y
hsa_circ_0052095	+	ENSG00000196214;	CVB5_5Y1,CVB5_5Y
hsa_circ_0000880	-	ENSG00000130254;	CVB5_5Y1,CVB5_5Y
hsa_circ_0005104	+	ENSG00000160633;	CVB5_5Y1,CVB5_5Y
hsa_circ_0006382	+	ENSG00000099783;	CVB5_5Y1,CVB5_5Y
hsa_circ_0046702	-	ENSG00000176105;	CVB5_5Y1,CVB5_5Y
hsa_circ_0008435	+	ENSG00000060069;	CVB5_5Y1,CVB5_5Y
hsa_circ_0005353	+	ENSG00000206418;	CVB5_5Y1,CVB5_5Y
hsa_circ_0008870	-	ENSG00000100030;	CVB5_5Y1,CVB5_5Y
hsa_circ_0007312	+	ENSG00000100014;ENSG0	CVB5_5Y1,CVB5_5Y
hsa_circ_0002931	-	ENSG00000183765;	CVB5_5Y1,CVB5_5Y
hsa_circ_0006752	+	ENSG00000186575;	CVB5_5Y1,CVB5_5Y
hsa_circ_0063534	-	ENSG00000100401;	CVB5_5Y1,CVB5_5Y
hsa_circ_0063716	+	ENSG00000241484;ENSG0	CVB5_5Y1,CVB5_5Y
hsa_circ_0006117	+	ENSG00000132670;	CVB5_5Y1,CVB5_5Y
hsa_circ_0001137	+	ENSG00000088305;	CVB5_5Y1,CVB5_5Y
hsa_circ_0008817	-	ENSG00000131051;	CVB5_5Y1,CVB5_5Y
hsa_circ_0060420	-	ENSG00000124177;	CVB5_5Y1,CVB5_5Y
hsa_circ_0008253	-	ENSG00000054793;	CVB5_5Y1,CVB5_5Y
hsa_circ_0060927	-	ENSG00000019186;	CVB5_5Y1,CVB5_5Y
hsa_circ_0061342	-	ENSG00000142192;	CVB5_5Y1,CVB5_5Y

hsa_circ_0001187	+	ENSG00000142197;	CVB5_5Y1,CVB5_5Y
hsa_circ_0061774	+	ENSG00000182240;	CVB5_5Y1,CVB5_5Y
hsa_circ_0001200	-	ENSG00000183255;	CVB5_5Y1,CVB5_5Y
hsa_circ_0005037	+	ENSG00000197381;	CVB5_5Y1,CVB5_5Y
hsa_circ_0002903	+	ENSG00000160299;	CVB5_5Y1,CVB5_5Y
hsa_circ_0005925	+	ENSG00000121716;	CVB5_5Y1,CVB5_5Y
hsa_circ_0006501	+	ENSG00000257923;	CVB5_5Y1,CVB5_5Y
hsa_circ_0081751	-	ENSG00000105821;	CVB5_5Y1,CVB5_5Y
hsa_circ_0079440	+	ENSG00000106443;	CVB5_5Y1,CVB5_5Y
hsa_circ_0002699	+	ENSG00000105976;	CVB5_5Y1,CVB5_5Y
hsa_circ_0003655	+	ENSG00000197157;	CVB5_5Y1,CVB5_5Y
hsa_circ_0082444	+	ENSG00000131558;	CVB5_5Y1,CVB5_5Y
hsa_circ_0006357	-	ENSG00000106462;	CVB5_5Y1,CVB5_5Y
hsa_circ_0001766	-	ENSG00000155660;	CVB5_5Y1,CVB5_5Y
hsa_circ_0004351	+	ENSG00000197024;	CVB5_5Y1,CVB5_5Y
hsa_circ_0083176	+	ENSG00000105993;	CVB5_5Y1,CVB5_5Y
hsa_circ_0003162	+	ENSG00000122507;	CVB5_5Y1,CVB5_5Y
hsa_circ_0007501	-	ENSG00000011275;	CVB5_5Y1,CVB5_5Y
hsa_circ_0007417	-	ENSG00000229180;	CVB5_5Y1,CVB5_5Y
hsa_circ_0003866	-	ENSG00000009954;	CVB5_5Y1,CVB5_5Y
hsa_circ_0005588	+	ENSG00000071462;	CVB5_5Y1,CVB5_5Y
hsa_circ_0009012	-	ENSG00000106089;	CVB5_5Y1,CVB5_5Y
hsa_circ_0001638	+	ENSG00000173214;	CVB5_5Y1,CVB5_5Y
hsa_circ_0001641	-	ENSG00000112339;	CVB5_5Y1,CVB5_5Y
hsa_circ_0007218	+	ENSG00000124523;	CVB5_5Y1,CVB5_5Y
hsa_circ_0075796	-	ENSG00000124795;	CVB5_5Y1,CVB5_5Y
hsa_circ_0008846	-	ENSG00000124795;	CVB5_5Y1,CVB5_5Y
hsa_circ_0076178	-	ENSG00000096063;	CVB5_5Y1,CVB5_5Y
hsa_circ_0001608	-	ENSG00000112651;	CVB5_5Y1,CVB5_5Y
hsa_circ_0008285	+	ENSG00000153046;	CVB5_5Y1,CVB5_5Y
hsa_circ_0007874	+	ENSG00000135297;	CVB5_5Y1,CVB5_5Y
hsa_circ_0002465	+	ENSG00000156535;	CVB5_5Y1,CVB5_5Y
hsa_circ_0006131	-	ENSG00000135316;	CVB5_5Y1,CVB5_5Y
hsa_circ_0077292	-	ENSG00000111880;	CVB5_5Y1,CVB5_5Y
hsa_circ_0001619	-	ENSG00000112159;	CVB5_5Y1,CVB5_5Y
hsa_circ_0001529	-	ENSG00000072364;	CVB5_5Y1,CVB5_5Y
hsa_circ_0001531	+	ENSG00000170606;	CVB5_5Y1,CVB5_5Y
hsa_circ_0004854	-	ENSG00000113648;	CVB5_5Y1,CVB5_5Y
hsa_circ_0003280	-	ENSG00000113013;	CVB5_5Y1,CVB5_5Y
hsa_circ_0075341	-	ENSG00000050748;	CVB5_5Y1,CVB5_5Y
hsa_circ_0001470	-	ENSG00000113384;	CVB5_5Y1,CVB5_5Y
hsa_circ_0007071	+	ENSG00000172262;	CVB5_5Y1,CVB5_5Y
hsa_circ_0001479	+	ENSG00000172262;	CVB5_5Y1,CVB5_5Y
hsa_circ_0005730	+	ENSG00000134058;	CVB5_5Y1,CVB5_5Y
hsa_circ_0072954	+	ENSG00000157107;	CVB5_5Y1,CVB5_5Y
hsa_circ_0004405	-	ENSG00000198780;	CVB5_5Y1,CVB5_5Y
hsa_circ_0003550	-	ENSG00000071127;	CVB5_5Y1,CVB5_5Y
hsa_circ_0006935	-	ENSG00000138801;	CVB5_5Y1,CVB5_5Y
hsa_circ_0001436	+	ENSG00000138802;	CVB5_5Y1,CVB5_5Y
hsa_circ_0001438	+	ENSG00000138709;	CVB5_5Y1,CVB5_5Y
hsa_circ_0007619	+	ENSG00000138709;	CVB5_5Y1,CVB5_5Y
hsa_circ_0004731	-	ENSG00000157869;	CVB5_5Y1,CVB5_5Y
hsa_circ_0001445	+	ENSG00000153147;	CVB5_5Y1,CVB5_5Y
hsa_circ_0001447	-	ENSG00000151612;	CVB5_5Y1,CVB5_5Y
hsa_circ_0071422	-	ENSG00000154447;	CVB5_5Y1,CVB5_5Y
hsa_circ_0001459	+	ENSG00000109674;	CVB5_5Y1,CVB5_5Y
hsa_circ_0002212	-	ENSG00000129187;	CVB5_5Y1,CVB5_5Y
hsa_circ_0071615	-	ENSG00000083857;	CVB5_5Y1,CVB5_5Y

hsa_circ_0008872	+	ENSG00000063978;	CVB5_5Y1,CVB5_5Y
hsa_circ_0006737	-	ENSG00000087269;	CVB5_5Y1,CVB5_5Y
hsa_circ_0001400	-	ENSG00000181826;	CVB5_5Y1,CVB5_5Y
hsa_circ_0001403	-	ENSG00000035928;	CVB5_5Y1,CVB5_5Y
hsa_circ_0009154	+	ENSG00000078140;	CVB5_5Y1,CVB5_5Y
hsa_circ_0002590	+	ENSG00000078140;	CVB5_5Y1,CVB5_5Y
hsa_circ_0069492	+	ENSG00000078140;	CVB5_5Y1,CVB5_5Y
hsa_circ_0069559	-	ENSG00000163697;	CVB5_5Y1,CVB5_5Y
hsa_circ_0008453	-	ENSG00000163697;	CVB5_5Y1,CVB5_5Y
hsa_circ_0005912	+	ENSG00000145216;	CVB5_5Y1,CVB5_5Y
hsa_circ_0007476	+	ENSG00000145216;	CVB5_5Y1,CVB5_5Y
hsa_circ_0069748	+	ENSG00000145216;	CVB5_5Y1,CVB5_5Y
hsa_circ_0069987	+	ENSG00000163738;	CVB5_5Y1,CVB5_5Y
hsa_circ_0003451	+	ENSG00000145293;	CVB5_5Y1,CVB5_5Y
hsa_circ_0001421	-	ENSG00000138674;	CVB5_5Y1,CVB5_5Y
hsa_circ_0001324	+	ENSG00000114439;	CVB5_5Y1,CVB5_5Y
hsa_circ_0004639	+	ENSG00000177707;	CVB5_5Y1,CVB5_5Y
hsa_circ_0007315	+	ENSG00000177707;	CVB5_5Y1,CVB5_5Y
hsa_circ_0066783	+	ENSG00000144824;	CVB5_5Y1,CVB5_5Y
hsa_circ_0007986	-	ENSG00000082701;	CVB5_5Y1,CVB5_5Y
hsa_circ_0067103	-	ENSG00000163848;	CVB5_5Y1,CVB5_5Y
hsa_circ_0003181	+	ENSG00000159685;	CVB5_5Y1,CVB5_5Y
hsa_circ_0005768	-	ENSG00000163785;	CVB5_5Y1,CVB5_5Y
hsa_circ_0001359	-	ENSG00000173889;	CVB5_5Y1,CVB5_5Y
hsa_circ_0001360	-	ENSG00000173889;	CVB5_5Y1,CVB5_5Y
hsa_circ_0002387	-	ENSG00000154310;	CVB5_5Y1,CVB5_5Y
hsa_circ_0006248	-	ENSG00000136527;	CVB5_5Y1,CVB5_5Y
hsa_circ_0007203	-	ENSG00000075711;	CVB5_5Y1,CVB5_5Y
hsa_circ_0006215	-	ENSG00000033867;	CVB5_5Y1,CVB5_5Y
hsa_circ_0001277	-	ENSG00000033867;	CVB5_5Y1,CVB5_5Y
hsa_circ_0001294	-	ENSG00000173473;	CVB5_5Y1,CVB5_5Y
hsa_circ_0001296	-	ENSG00000173473;	CVB5_5Y1,CVB5_5Y
hsa_circ_0001307	+	ENSG00000164080;	CVB5_5Y1,CVB5_5Y
hsa_circ_0001309	-	ENSG00000114904;	CVB5_5Y1,CVB5_5Y
hsa_circ_0001312	+	ENSG00000180376;	CVB5_5Y1,CVB5_5Y
hsa_circ_0003540	-	ENSG00000151276;	CVB5_5Y1,CVB5_5Y
hsa_circ_0003101	+	ENSG00000144741;	CVB5_5Y1,CVB5_5Y
hsa_circ_0006135	+	ENSG00000169379;	CVB5_5Y1,CVB5_5Y
hsa_circ_0001062	+	ENSG00000188177;	CVB5_5Y1,CVB5_5Y
hsa_circ_0001070	+	ENSG00000048991;	CVB5_5Y1,CVB5_5Y
hsa_circ_0001074	-	ENSG00000115947;	CVB5_5Y1,CVB5_5Y
hsa_circ_0000978	-	ENSG00000151779;	CVB5_5Y1,CVB5_5Y
hsa_circ_0008010	+	ENSG00000115183;	CVB5_5Y1,CVB5_5Y
hsa_circ_0003296	-	ENSG00000198648;	CVB5_5Y1,CVB5_5Y
hsa_circ_0002141	+	ENSG00000204186;	CVB5_5Y1,CVB5_5Y
hsa_circ_0008544	+	n/a	CVB5_5Y1,CVB5_5Y
hsa_circ_0007648	-	ENSG00000115568;	CVB5_5Y1,CVB5_5Y
hsa_circ_0058456	-	ENSG00000286239;ENSG0	CVB5_5Y1,CVB5_5Y
hsa_circ_0008382	+	ENSG00000185404;	CVB5_5Y1,CVB5_5Y
hsa_circ_0003922	+	ENSG00000067066;	CVB5_5Y1,CVB5_5Y
hsa_circ_0007321	+	ENSG00000144535;	CVB5_5Y1,CVB5_5Y
hsa_circ_0003341	+	ENSG00000204120;	CVB5_5Y1,CVB5_5Y
hsa_circ_0001115	+	ENSG00000077044;	CVB5_5Y1,CVB5_5Y
hsa_circ_0005059	-	ENSG00000144504;	CVB5_5Y1,CVB5_5Y
hsa_circ_0000985	+	ENSG00000243943;ENSG0	CVB5_5Y1,CVB5_5Y
hsa_circ_0006794	-	ENSG00000138081;	CVB5_5Y1,CVB5_5Y
hsa_circ_0005552	+	ENSG00000115504;	CVB5_5Y1,CVB5_5Y
hsa_circ_0001023	+	ENSG00000169621;	CVB5_5Y1,CVB5_5Y

hsa_circ_0007092	-	ENSG00000135945;	CVB5_5Y1,CVB5_5Y
hsa_circ_0000116	+	ENSG00000198162;	CVB5_5Y1,CVB5_5Y
hsa_circ_0000117	+	ENSG00000198162;	CVB5_5Y1,CVB5_5Y
hsa_circ_0000120	+	ENSG00000198162;	CVB5_5Y1,CVB5_5Y
hsa_circ_0006470	+	ENSG00000116688;	CVB5_5Y1,CVB5_5Y
hsa_circ_0000002	-	ENSG00000078808;	CVB5_5Y1,CVB5_5Y
hsa_circ_0008063	-	ENSG00000160087;	CVB5_5Y1,CVB5_5Y
hsa_circ_0010023	-	ENSG00000162496;	CVB5_5Y1,CVB5_5Y
hsa_circ_0013953	+	ENSG00000131778;ENSG0	CVB5_5Y1,CVB5_5Y
hsa_circ_0000128	+	ENSG00000163156;	CVB5_5Y1,CVB5_5Y
hsa_circ_0014613	+	ENSG00000132676;	CVB5_5Y1,CVB5_5Y
hsa_circ_0000019	+	ENSG00000197312;	CVB5_5Y1,CVB5_5Y
hsa_circ_0006758	+	ENSG00000143179;	CVB5_5Y1,CVB5_5Y
hsa_circ_0004722	-	ENSG00000143207;	CVB5_5Y1,CVB5_5Y
hsa_circ_0005522	+	ENSG00000136643;	CVB5_5Y1,CVB5_5Y
hsa_circ_0000028	-	ENSG00000090686;	CVB5_5Y1,CVB5_5Y
hsa_circ_0002166	-	ENSG00000090686;	CVB5_5Y1,CVB5_5Y
hsa_circ_0007739	-	ENSG00000136628;	CVB5_5Y1,CVB5_5Y
hsa_circ_0000198	-	ENSG00000135749;	CVB5_5Y1,CVB5_5Y
hsa_circ_0003553	+	ENSG00000117602;	CVB5_5Y1,CVB5_5Y
hsa_circ_0003940	+	ENSG00000090273;	CVB5_5Y1,CVB5_5Y
hsa_circ_0005087	+	ENSG00000090273;	CVB5_5Y1,CVB5_5Y
hsa_circ_0008057	-	ENSG00000158161;	CVB5_5Y1,CVB5_5Y
hsa_circ_0007895	-	ENSG00000158161;	CVB5_5Y1,CVB5_5Y
hsa_circ_0006602	-	ENSG00000116350;	CVB5_5Y1,CVB5_5Y
hsa_circ_0000045	-	ENSG00000134644;	CVB5_5Y1,CVB5_5Y
hsa_circ_0007249	+	ENSG00000121766;	CVB5_5Y1,CVB5_5Y
hsa_circ_0003238	+	ENSG00000084652;	CVB5_5Y1,CVB5_5Y
hsa_circ_0009027	+	ENSG00000160094;	CVB5_5Y1,CVB5_5Y
hsa_circ_0000061	-	ENSG00000010803;	CVB5_5Y1,CVB5_5Y
hsa_circ_0008774	-	ENSG00000159592;	CVB5_5Y1,CVB5_5Y
hsa_circ_0012333	+	ENSG00000173660;	CVB5_5Y1,CVB5_5Y
hsa_circ_0008657	+	ENSG00000162607;	CVB5_5Y1,CVB5_5Y
hsa_circ_0006354	+	ENSG00000049245;	CVB5_5Y1,CVB5_5Y
hsa_circ_0009674	-	ENSG00000178585;	CVB5_5Y1,CVB5_5Y
hsa_circ_0087905	-	ENSG00000106771;	CVB5_5Y1,CVB5_5Y
hsa_circ_0002672	+	ENSG00000011454;	CVB5_5Y1,CVB5_5Y
hsa_circ_0088427	-	ENSG00000165209;	CVB5_5Y1,CVB5_5Y
hsa_circ_0088614	+	ENSG00000196814;	CVB5_5Y1,CVB5_5Y
hsa_circ_0005871	+	ENSG00000130723;	CVB5_5Y1,CVB5_5Y
hsa_circ_0089310	+	ENSG00000148308;	CVB5_5Y1,CVB5_5Y
hsa_circ_0089371	-	ENSG00000148300;	CVB5_5Y1,CVB5_5Y
hsa_circ_0006502	-	ENSG00000148399;	CVB5_5Y1,CVB5_5Y
hsa_circ_0001904	+	ENSG00000181090;	CVB5_5Y1,CVB5_5Y
hsa_circ_0089727	+	ENSG00000181090;	CVB5_5Y1,CVB5_5Y
hsa_circ_0001851	-	ENSG00000137073;	CVB5_5Y1,CVB5_5Y
hsa_circ_0086735	-	ENSG00000137073;	CVB5_5Y1,CVB5_5Y
hsa_circ_0001860	+	ENSG00000147905;	CVB5_5Y1,CVB5_5Y
hsa_circ_0006345	+	ENSG00000120158;	CVB5_5Y1,CVB5_5Y
hsa_circ_0087630	+	ENSG00000130956;	CVB5_5Y1,CVB5_5Y
hsa_circ_0087631	+	ENSG00000130956;	CVB5_5Y1,CVB5_5Y
hsa_circ_0006623	-	ENSG00000070756;	CVB5_5Y1,CVB5_5Y
hsa_circ_0085173	+	ENSG00000083307;	CVB5_5Y1,CVB5_5Y
hsa_circ_0008849	+	ENSG00000249859;	CVB5_5Y1,CVB5_5Y
hsa_circ_0002965	-	ENSG00000123908;	CVB5_5Y1,CVB5_5Y
hsa_circ_0007313	-	ENSG00000123908;	CVB5_5Y1,CVB5_5Y
hsa_circ_0086154	-	ENSG00000147799;	CVB5_5Y1,CVB5_5Y
hsa_circ_0083443	-	ENSG00000129422;	CVB5_5Y1,CVB5_5Y

hsa_circ_0007618	+	ENSG00000147459;	CVB5_5Y1,CVB5_5Y
hsa_circ_0007353	+	ENSG00000104660;	CVB5_5Y1,CVB5_5Y
hsa_circ_0006376	+	ENSG00000168172;	CVB5_5Y1,CVB5_5Y
hsa_circ_0007581	+	ENSG00000104388;	CVB5_5Y1,CVB5_5Y
hsa_circ_0003388	+	ENSG00000104218;	CVB5_5Y1,CVB5_5Y
hsa_circ_0084889	+	ENSG00000164953;	CVB5_5Y1,CVB5_5Y
hsa_circ_0084927	+	ENSG00000104413;	CVB5_5Y1,CVB5_5Y
novel_circ_0002806	-	ENSG00000134884;	CVB5_5Y1,CVB5_5Y
novel_circ_0002650	+	ENSG00000258168;	CVB5_5Y1,CVB5_5Y
novel_circ_0002663	-	ENSG00000251138;	CVB5_5Y1,CVB5_5Y
novel_circ_0001137	-	ENSG00000110367;	CVB5_5Y1,CVB5_5Y
novel_circ_0001430	-	ENSG00000175216;	CVB5_5Y1,CVB5_5Y
novel_circ_0001541	+	ENSG00000173715;	CVB5_5Y1,CVB5_5Y
novel_circ_0000124	+	n/a	CVB5_5Y1,CVB5_5Y
novel_circ_0000374	-	n/a	CVB5_5Y1,CVB5_5Y
novel_circ_0005636	+	ENSG00000136448;	CVB5_5Y1,CVB5_5Y
novel_circ_0005739	-	n/a	CVB5_5Y1,CVB5_5Y
novel_circ_0005779	+	ENSG00000166263;	CVB5_5Y1,CVB5_5Y
novel_circ_0006024	-	n/a	CVB5_5Y1,CVB5_5Y
novel_circ_0006109	+	ENSG00000173821;	CVB5_5Y1,CVB5_5Y
novel_circ_0006148	+	ENSG00000185298;	CVB5_5Y1,CVB5_5Y
novel_circ_0006197	+	ENSG00000141556;	CVB5_5Y1,CVB5_5Y
novel_circ_0005191	+	ENSG00000158545;	CVB5_5Y1,CVB5_5Y
novel_circ_0004099	-	ENSG00000067369;	CVB5_5Y1,CVB5_5Y
novel_circ_0004201	+	ENSG00000151575;	CVB5_5Y1,CVB5_5Y
novel_circ_0004328	+	ENSG00000166949;	CVB5_5Y1,CVB5_5Y
novel_circ_0004429	+	ENSG00000140367;	CVB5_5Y1,CVB5_5Y
novel_circ_0003518	+	ENSG00000100941;	CVB5_5Y1,CVB5_5Y
novel_circ_0003535	-	ENSG00000100442;	CVB5_5Y1,CVB5_5Y
novel_circ_0006759	-	n/a	CVB5_5Y1,CVB5_5Y
novel_circ_0006772	-	ENSG00000006016;	CVB5_5Y1,CVB5_5Y
novel_circ_0006852	-	ENSG00000121289;	CVB5_5Y1,CVB5_5Y
novel_circ_0006440	+	ENSG00000167216;	CVB5_5Y1,CVB5_5Y
novel_circ_0009963	+	ENSG00000130638;	CVB5_5Y1,CVB5_5Y
novel_circ_0009270	+	n/a	CVB5_5Y1,CVB5_5Y
novel_circ_0009272	+	n/a	CVB5_5Y1,CVB5_5Y
novel_circ_0009458	+	ENSG00000101187;	CVB5_5Y1,CVB5_5Y
novel_circ_0016055	+	ENSG00000146963;ENSG0	CVB5_5Y1,CVB5_5Y
novel_circ_0016269	-	ENSG00000136231;	CVB5_5Y1,CVB5_5Y
novel_circ_0016552	-	ENSG00000229180;	CVB5_5Y1,CVB5_5Y
novel_circ_0015128	-	ENSG00000135525;	CVB5_5Y1,CVB5_5Y
novel_circ_0015148	+	ENSG00000009844;	CVB5_5Y1,CVB5_5Y
novel_circ_0015330	+	ENSG00000130396;	CVB5_5Y1,CVB5_5Y
novel_circ_0015382	-	ENSG00000124795;	CVB5_5Y1,CVB5_5Y
novel_circ_0015427	-	ENSG00000204560;	CVB5_5Y1,CVB5_5Y
novel_circ_0015527	+	ENSG00000124541;	CVB5_5Y1,CVB5_5Y
novel_circ_0015556	+	ENSG00000198087;	CVB5_5Y1,CVB5_5Y
novel_circ_0015574	-	ENSG00000012660;	CVB5_5Y1,CVB5_5Y
novel_circ_0015621	+	ENSG00000285976;ENSG0	CVB5_5Y1,CVB5_5Y
novel_circ_0015686	-	n/a	CVB5_5Y1,CVB5_5Y
novel_circ_0013976	-	ENSG00000251574;	CVB5_5Y1,CVB5_5Y
novel_circ_0014037	+	ENSG00000177879;	CVB5_5Y1,CVB5_5Y
novel_circ_0014371	-	ENSG00000145555;	CVB5_5Y1,CVB5_5Y
novel_circ_0014433	+	n/a	CVB5_5Y1,CVB5_5Y
novel_circ_0014720	+	ENSG00000145734;	CVB5_5Y1,CVB5_5Y
novel_circ_0014922	-	ENSG00000153922;	CVB5_5Y1,CVB5_5Y
novel_circ_0013402	-	ENSG00000181381;	CVB5_5Y1,CVB5_5Y
novel_circ_0013492	-	ENSG00000083857;	CVB5_5Y1,CVB5_5Y

novel_circ_0011803	+	ENSG00000177707;	CVB5_5Y1,CVB5_5Y
novel_circ_0012459	-	ENSG00000163961;	CVB5_5Y1,CVB5_5Y
novel_circ_0012552	-	ENSG00000235493;	CVB5_5Y1,CVB5_5Y
novel_circ_0012593	-	ENSG00000144635;	CVB5_5Y1,CVB5_5Y
novel_circ_0012594	-	ENSG00000144635;	CVB5_5Y1,CVB5_5Y
novel_circ_0012603	+	ENSG00000182973;	CVB5_5Y1,CVB5_5Y
novel_circ_0012937	+	n/a	CVB5_5Y1,CVB5_5Y
novel_circ_0010775	+	n/a	CVB5_5Y1,CVB5_5Y
novel_circ_0010779	+	n/a	CVB5_5Y1,CVB5_5Y
novel_circ_0010783	+	n/a	CVB5_5Y1,CVB5_5Y
novel_circ_0010819	-	ENSG00000152056;ENSG0	CVB5_5Y1,CVB5_5Y
novel_circ_0010862	+	ENSG00000185404;	CVB5_5Y1,CVB5_5Y
novel_circ_0011032	-	ENSG00000138074;	CVB5_5Y1,CVB5_5Y
novel_circ_0011163	+	ENSG00000234171;	CVB5_5Y1,CVB5_5Y
novel_circ_0011329	+	ENSG00000119888;	CVB5_5Y1,CVB5_5Y
novel_circ_0011332	+	ENSG00000119888;	CVB5_5Y1,CVB5_5Y
novel_circ_0011411	+	ENSG00000173209;	CVB5_5Y1,CVB5_5Y
novel_circ_0011420	-	ENSG00000115464;	CVB5_5Y1,CVB5_5Y
novel_circ_0011475	-	ENSG00000082898;	CVB5_5Y1,CVB5_5Y
novel_circ_0007238	-	ENSG00000162777;	CVB5_5Y1,CVB5_5Y
novel_circ_0007543	-	ENSG00000215908;	CVB5_5Y1,CVB5_5Y
novel_circ_0007757	-	ENSG00000285280;	CVB5_5Y1,CVB5_5Y
novel_circ_0008295	+	ENSG00000157978;	CVB5_5Y1,CVB5_5Y
novel_circ_0008301	+	ENSG00000130695;	CVB5_5Y1,CVB5_5Y
novel_circ_0008468	-	ENSG00000134697;	CVB5_5Y1,CVB5_5Y
novel_circ_0008796	+	ENSG00000198160;	CVB5_5Y1,CVB5_5Y
novel_circ_0008931	-	ENSG00000142599;	CVB5_5Y1,CVB5_5Y
novel_circ_0018216	+	ENSG00000086061;	CVB5_5Y1,CVB5_5Y
novel_circ_0018254	-	ENSG00000137073;	CVB5_5Y1,CVB5_5Y
novel_circ_0018350	-	ENSG00000183354;	CVB5_5Y1,CVB5_5Y
novel_circ_0016887	+	ENSG00000104412;	CVB5_5Y1,CVB5_5Y
novel_circ_0017230	-	ENSG00000120889;	CVB5_5Y1,CVB5_5Y
novel_circ_0017461	+	ENSG00000171316;	CVB5_5Y1,CVB5_5Y
novel_circ_0017747	+	ENSG00000132549;	CVB5_5Y1,CVB5_5Y
hsa_circ_0007232	-	ENSG00000102753;	CVB5_5Y1,CVB5_5Y
hsa_circ_0000416	+	ENSG00000135679;	CVB5_5Y1,CVB5_5Y
hsa_circ_0017438	+	ENSG00000047056;	CVB5_5Y1,CVB5_5Y
hsa_circ_0005516	-	ENSG00000148908;	CVB5_5Y1,CVB5_5Y
hsa_circ_0007759	-	ENSG00000108506;	CVB5_5Y1,CVB5_5Y
hsa_circ_0000631	-	ENSG00000159322;	CVB5_5Y1,CVB5_5Y
hsa_circ_0050256	-	ENSG00000105726;	CVB5_5Y1,CVB5_5Y
hsa_circ_0075648	+	ENSG00000124523;	CVB5_5Y1,CVB5_5Y
hsa_circ_0078563	-	ENSG00000120438;	CVB5_5Y1,CVB5_5Y
hsa_circ_0072654	+	ENSG00000153015;	CVB5_5Y1,CVB5_5Y
hsa_circ_0001413	+	ENSG00000145216;	CVB5_5Y1,CVB5_5Y
hsa_circ_0001282	+	ENSG00000144674;	CVB5_5Y1,CVB5_5Y
hsa_circ_0058876	-	ENSG00000132323;	CVB5_5Y1,CVB5_5Y
hsa_circ_0009246	-	ENSG00000160087;	CVB5_5Y1,CVB5_5Y
hsa_circ_0014306	+	ENSG00000143624;	CVB5_5Y1,CVB5_5Y
hsa_circ_0008998	-	ENSG00000132680;	CVB5_5Y1,CVB5_5Y
hsa_circ_0009040	-	ENSG00000171603;	CVB5_5Y1,CVB5_5Y
hsa_circ_0007613	+	ENSG00000130723;	CVB5_5Y1,CVB5_5Y
hsa_circ_0002647	+	ENSG00000181090;	CVB5_5Y1,CVB5_5Y
hsa_circ_0085673	-	ENSG00000066827;	CVB5_5Y1,CVB5_5Y
hsa_circ_0001790	+	ENSG00000129691;	CVB5_5Y1,CVB5_5Y
novel_circ_0001462	+	ENSG00000070047;	CVB5_5Y1,CVB5_5Y
novel_circ_0006222	-	ENSG00000175711;	CVB5_5Y1,CVB5_5Y
novel_circ_0004952	-	n/a	CVB5_5Y1,CVB5_5Y

novel_circ_0004971	-	ENSG00000141098;	CVB5_5Y1,CVB5_5Y
novel_circ_0003600	+	ENSG00000258479;	CVB5_5Y1,CVB5_5Y
novel_circ_0015012	+	ENSG00000196911;	CVB5_5Y1,CVB5_5Y
novel_circ_0007915	+	ENSG00000162769;	CVB5_5Y1,CVB5_5Y
novel_circ_0009044	+	ENSG00000173614;	CVB5_5Y1,CVB5_5Y
novel_circ_0018592	+	ENSG00000095380;	CVB5_5Y1,CVB5_5Y
hsa_circ_0091223	-	ENSG00000188917;	CVB5_5Y1,CVB5_5Y
hsa_circ_0008539	+	ENSG00000150403;	CVB5_5Y1,CVB5_5Y
hsa_circ_0026238	+	ENSG00000161813;	CVB5_5Y1,CVB5_5Y
hsa_circ_0026627	+	ENSG00000185591;	CVB5_5Y1,CVB5_5Y
hsa_circ_0008194	-	ENSG00000124942;	CVB5_5Y1,CVB5_5Y
hsa_circ_0020197	+	ENSG00000107651;	CVB5_5Y1,CVB5_5Y
hsa_circ_0002050	+	ENSG00000150760;	CVB5_5Y1,CVB5_5Y
hsa_circ_0008883	+	ENSG00000103326;	CVB5_5Y1,CVB5_5Y
hsa_circ_0003848	+	ENSG00000080815;	CVB5_5Y1,CVB5_5Y
hsa_circ_0049700	+	ENSG00000132024;	CVB5_5Y1,CVB5_5Y
hsa_circ_0008214	-	ENSG00000123159;	CVB5_5Y1,CVB5_5Y
hsa_circ_0047650	-	ENSG00000141627;	CVB5_5Y1,CVB5_5Y
hsa_circ_0000852	+	ENSG00000091157;	CVB5_5Y1,CVB5_5Y
hsa_circ_0062156	-	ENSG00000069998;	CVB5_5Y1,CVB5_5Y
hsa_circ_0005917	+	ENSG00000111845;	CVB5_5Y1,CVB5_5Y
hsa_circ_0001640	-	ENSG00000079819;	CVB5_5Y1,CVB5_5Y
hsa_circ_0001574	+	ENSG00000168566;	CVB5_5Y1,CVB5_5Y
hsa_circ_0002972	-	ENSG00000146067;	CVB5_5Y1,CVB5_5Y
hsa_circ_0068957	-	ENSG00000087269;	CVB5_5Y1,CVB5_5Y
hsa_circ_0064802	+	ENSG00000170248;	CVB5_5Y1,CVB5_5Y
hsa_circ_0065251	-	ENSG00000173473;	CVB5_5Y1,CVB5_5Y
hsa_circ_0055019	+	ENSG00000169621;	CVB5_5Y1,CVB5_5Y
hsa_circ_0005986	+	ENSG00000116731;	CVB5_5Y1,CVB5_5Y
hsa_circ_0003952	+	ENSG00000143374;	CVB5_5Y1,CVB5_5Y
hsa_circ_0011585	-	ENSG00000092853;	CVB5_5Y1,CVB5_5Y
novel_circ_0005117	-	ENSG00000153774;	CVB5_5Y1,CVB5_5Y
novel_circ_0006788	+	ENSG00000167491;	CVB5_5Y1,CVB5_5Y
novel_circ_0006953	-	ENSG00000176531;	CVB5_5Y1,CVB5_5Y
novel_circ_0009100	+	ENSG00000088930;	CVB5_5Y1,CVB5_5Y
novel_circ_0016629	+	ENSG00000135205;	CVB5_5Y1,CVB5_5Y
novel_circ_0015457	+	ENSG00000137309;	CVB5_5Y1,CVB5_5Y
novel_circ_0014698	-	ENSG00000171368;	CVB5_5Y1,CVB5_5Y
novel_circ_0010211	-	ENSG00000224043;	CVB5_5Y1,CVB5_5Y
novel_circ_0010830	-	ENSG00000036257;	CVB5_5Y1,CVB5_5Y
hsa_circ_0000374	+	ENSG00000111203;	CVB5_5Y1,CVB5_5Y
hsa_circ_0025908	+	ENSG00000134283;	CVB5_5Y1,CVB5_5Y
hsa_circ_0027494	+	ENSG00000135679;	CVB5_5Y1,CVB5_5Y
hsa_circ_0021644	+	ENSG00000135387;	CVB5_5Y1,CVB5_5Y
hsa_circ_0042437	+	ENSG00000128487;	CVB5_5Y1,CVB5_5Y
hsa_circ_0046263	-	ENSG00000185624;	CVB5_5Y1,CVB5_5Y
hsa_circ_0005725	-	ENSG00000178741;	CVB5_5Y1,CVB5_5Y
hsa_circ_0006779	+	ENSG00000140525;	CVB5_5Y1,CVB5_5Y
hsa_circ_0052011	+	ENSG00000062822;	CVB5_5Y1,CVB5_5Y
hsa_circ_0000958	-	ENSG00000125503;	CVB5_5Y1,CVB5_5Y
hsa_circ_0000860	+	ENSG00000060069;	CVB5_5Y1,CVB5_5Y
hsa_circ_0062169	+	ENSG00000099968;	CVB5_5Y1,CVB5_5Y
hsa_circ_0007004	+	ENSG00000128563;	CVB5_5Y1,CVB5_5Y
hsa_circ_0001541	+	ENSG00000131503;ENSG0	CVB5_5Y1,CVB5_5Y
hsa_circ_0064871	+	ENSG00000172939;	CVB5_5Y1,CVB5_5Y
hsa_circ_0005520	+	ENSG00000171848;	CVB5_5Y1,CVB5_5Y
hsa_circ_0001124	+	ENSG00000168395;	CVB5_5Y1,CVB5_5Y
hsa_circ_0015733	-	ENSG00000116750;	CVB5_5Y1,CVB5_5Y

hsa_circ_0006415	-	ENSG00000135749;	CVB5_5Y1,CVB5_5Y
hsa_circ_0002043	+	ENSG00000146463;	CVB5_5Y1,CVB5_5Y
hsa_circ_0006388	+	ENSG00000066697;	CVB5_5Y1,CVB5_5Y
hsa_circ_0001789	-	ENSG00000156675;	CVB5_5Y1,CVB5_5Y
hsa_circ_0001796	+	ENSG00000168522;ENSG0	CVB5_5Y1,CVB5_5Y
novel_circ_0001087	-	ENSG00000137710;	CVB5_5Y1,CVB5_5Y
novel_circ_0003562	-	ENSG00000165525;	CVB5_5Y1,CVB5_5Y
novel_circ_0009274	+	n/a	CVB5_5Y1,CVB5_5Y
novel_circ_0016058	+	ENSG00000269955;ENSG0	CVB5_5Y1,CVB5_5Y
novel_circ_0016583	-	ENSG00000277149;	CVB5_5Y1,CVB5_5Y
novel_circ_0015332	+	ENSG00000130396;	CVB5_5Y1,CVB5_5Y
novel_circ_0014669	-	ENSG00000123219;	CVB5_5Y1,CVB5_5Y
novel_circ_0011883	-	ENSG00000051341;	CVB5_5Y1,CVB5_5Y
novel_circ_0011333	+	ENSG00000119888;	CVB5_5Y1,CVB5_5Y
novel_circ_0016868	-	ENSG00000251003;	CVB5_5Y1,CVB5_5Y
novel_circ_0016987	+	ENSG00000286122;	CVB5_5Y1,CVB5_5Y
hsa_circ_0029468	+	ENSG00000183495;	CVB5_5Y1,CVB5_5Y
hsa_circ_0018553	+	ENSG00000060339;	CVB5_5Y1,CVB5_5Y
hsa_circ_0003704	-	ENSG00000103404;	CVB5_5Y1,CVB5_5Y
hsa_circ_0036730	+	ENSG00000140534;	CVB5_5Y1,CVB5_5Y
hsa_circ_0008479	+	n/a	CVB5_5Y1,CVB5_5Y
hsa_circ_0007532	+	ENSG00000121152;	CVB5_5Y1,CVB5_5Y
hsa_circ_0083757	-	ENSG00000104228;	CVB5_5Y1,CVB5_5Y
novel_circ_0002516	+	ENSG00000185591;	CVB5_5Y1,CVB5_5Y
novel_circ_0003597	+	ENSG00000258479;	CVB5_5Y1,CVB5_5Y
novel_circ_0014022	-	ENSG00000171444;	CVB5_5Y1,CVB5_5Y
hsa_circ_0030883	+	ENSG00000213995;	CVB5_5Y1,CVB5_5Y
hsa_circ_0005086	+	ENSG00000022840;	CVB5_5Y1,CVB5_5Y
hsa_circ_0027058	-	ENSG00000076108;	CVB5_5Y1,CVB5_5Y
hsa_circ_0044224	-	ENSG00000004897;	CVB5_5Y1,CVB5_5Y
hsa_circ_0008740	-	ENSG00000141252;	CVB5_5Y1,CVB5_5Y
hsa_circ_0045319	-	ENSG00000108854;	CVB5_5Y1,CVB5_5Y
hsa_circ_0008255	+	ENSG00000103222;	CVB5_5Y1,CVB5_5Y
hsa_circ_0037748	-	ENSG00000103199;	CVB5_5Y1,CVB5_5Y
hsa_circ_0040569	+	ENSG00000186153;	CVB5_5Y1,CVB5_5Y
hsa_circ_0049459	-	ENSG00000205517;	CVB5_5Y1,CVB5_5Y
hsa_circ_0000932	+	ENSG00000196235;	CVB5_5Y1,CVB5_5Y
hsa_circ_0079385	+	ENSG00000136247;	CVB5_5Y1,CVB5_5Y
hsa_circ_0003418	-	ENSG00000204371;	CVB5_5Y1,CVB5_5Y
hsa_circ_0002102	+	ENSG00000188846;	CVB5_5Y1,CVB5_5Y
hsa_circ_0007198	-	ENSG00000173473;	CVB5_5Y1,CVB5_5Y
hsa_circ_0005315	-	ENSG00000134313;	CVB5_5Y1,CVB5_5Y
hsa_circ_0007615	-	ENSG00000152104;	CVB5_5Y1,CVB5_5Y
hsa_circ_0010850	-	ENSG00000125944;	CVB5_5Y1,CVB5_5Y
hsa_circ_0000057	-	ENSG00000043514;	CVB5_5Y1,CVB5_5Y
hsa_circ_0009076	-	ENSG00000078618;	CVB5_5Y1,CVB5_5Y
hsa_circ_0088398	-	ENSG00000056586;	CVB5_5Y1,CVB5_5Y
hsa_circ_0007230	-	ENSG00000119487;	CVB5_5Y1,CVB5_5Y
hsa_circ_0086708	-	ENSG00000137073;	CVB5_5Y1,CVB5_5Y
hsa_circ_0087606	+	ENSG00000182150;	CVB5_5Y1,CVB5_5Y
novel_circ_0002295	+	ENSG00000029153;	CVB5_5Y1,CVB5_5Y
novel_circ_0005902	-	ENSG00000108510;	CVB5_5Y1,CVB5_5Y
novel_circ_0006147	+	ENSG00000185298;	CVB5_5Y1,CVB5_5Y
novel_circ_0004434	+	ENSG00000167196;	CVB5_5Y1,CVB5_5Y
novel_circ_0009450	+	ENSG00000130703;	CVB5_5Y1,CVB5_5Y
novel_circ_0013977	-	ENSG00000113504;	CVB5_5Y1,CVB5_5Y
novel_circ_0011125	+	ENSG00000115760;	CVB5_5Y1,CVB5_5Y
novel_circ_0007348	-	ENSG00000264522;	CVB5_5Y1,CVB5_5Y

novel_circ_0007476	-	ENSG00000160075;	CVB5_5Y1,CVB5_5Y
novel_circ_0018097	+	ENSG00000188986;	CVB5_5Y1,CVB5_5Y
hsa_circ_0027621	+	ENSG00000186908;	CVB5_5Y1,CVB5_5Y
hsa_circ_0021727	+	ENSG00000026508;	CVB5_5Y1,CVB5_5Y
hsa_circ_0002677	+	ENSG00000170836;	CVB5_5Y1,CVB5_5Y
hsa_circ_0004440	+	ENSG00000007047;	CVB5_5Y1,CVB5_5Y
hsa_circ_0060746	+	ENSG00000124207;	CVB5_5Y1,CVB5_5Y
hsa_circ_0080026	-	ENSG00000015676;	CVB5_5Y1,CVB5_5Y
hsa_circ_0078673	+	ENSG00000130396;	CVB5_5Y1,CVB5_5Y
hsa_circ_0073585	+	ENSG00000134982;	CVB5_5Y1,CVB5_5Y
hsa_circ_0001456	-	ENSG00000137601;	CVB5_5Y1,CVB5_5Y
hsa_circ_0009134	+	ENSG00000181090;	CVB5_5Y1,CVB5_5Y
hsa_circ_0005062	+	ENSG00000107104;	CVB5_5Y1,CVB5_5Y
hsa_circ_0085268	-	ENSG00000104517;	CVB5_5Y1,CVB5_5Y
hsa_circ_0004686	-	ENSG00000169398;	CVB5_5Y1,CVB5_5Y
hsa_circ_0007545	-	ENSG00000104375;	CVB5_5Y1,CVB5_5Y
novel_circ_0018917	-	ENSG00000165288;	CVB5_5Y1,CVB5_5Y
novel_circ_0003074	-	ENSG00000123200;	CVB5_5Y1,CVB5_5Y
novel_circ_0012332	-	ENSG00000043093;	CVB5_5Y1,CVB5_5Y
novel_circ_0018138	+	ENSG00000044459;	CVB5_5Y1,CVB5_5Y
hsa_circ_0007401	-	ENSG00000110713;	CVB5_5Y1,CVB5_5Y
hsa_circ_0008145	-	ENSG00000138303;	CVB5_5Y1,CVB5_5Y
hsa_circ_0041443	+	ENSG00000132359;	CVB5_5Y1,CVB5_5Y
hsa_circ_0045202	+	ENSG00000136485;	CVB5_5Y1,CVB5_5Y
hsa_circ_0002879	-	ENSG00000141252;	CVB5_5Y1,CVB5_5Y
hsa_circ_0039844	+	ENSG00000102974;	CVB5_5Y1,CVB5_5Y
hsa_circ_0040321	-	ENSG00000103043;	CVB5_5Y1,CVB5_5Y
hsa_circ_0000613	+	ENSG00000140455;	CVB5_5Y1,CVB5_5Y
hsa_circ_0004624	+	ENSG00000257103;	CVB5_5Y1,CVB5_5Y
hsa_circ_0007022	+	ENSG00000142230;	CVB5_5Y1,CVB5_5Y
hsa_circ_0001966	-	ENSG00000142192;	CVB5_5Y1,CVB5_5Y
hsa_circ_0008466	-	ENSG00000185658;	CVB5_5Y1,CVB5_5Y
hsa_circ_0079668	+	ENSG00000106052;	CVB5_5Y1,CVB5_5Y
hsa_circ_0004119	-	ENSG00000112210;	CVB5_5Y1,CVB5_5Y
hsa_circ_0001528	+	ENSG00000113522;ENSG0	CVB5_5Y1,CVB5_5Y
hsa_circ_0072081	-	ENSG00000150712;	CVB5_5Y1,CVB5_5Y
hsa_circ_0070272	-	ENSG00000173085;	CVB5_5Y1,CVB5_5Y
hsa_circ_0007728	-	ENSG00000206560;	CVB5_5Y1,CVB5_5Y
hsa_circ_0008124	+	ENSG00000152492;	CVB5_5Y1,CVB5_5Y
hsa_circ_0054034	-	ENSG00000115808;	CVB5_5Y1,CVB5_5Y
hsa_circ_0054469	+	ENSG00000095002;	CVB5_5Y1,CVB5_5Y
hsa_circ_0005521	+	ENSG00000136636;	CVB5_5Y1,CVB5_5Y
hsa_circ_0011542	+	ENSG00000146463;	CVB5_5Y1,CVB5_5Y
hsa_circ_0009025	-	ENSG00000080608;	CVB5_5Y1,CVB5_5Y
hsa_circ_0005499	-	ENSG00000285982;ENSG0	CVB5_5Y1,CVB5_5Y
novel_circ_0003068	-	ENSG00000123200;	CVB5_5Y1,CVB5_5Y
novel_circ_0002391	+	ENSG00000120647;	CVB5_5Y1,CVB5_5Y
novel_circ_0000171	-	ENSG00000187164;	CVB5_5Y1,CVB5_5Y
novel_circ_0005907	-	ENSG00000108510;	CVB5_5Y1,CVB5_5Y
novel_circ_0015971	+	n/a	CVB5_5Y1,CVB5_5Y
novel_circ_0015063	+	ENSG00000066651;	CVB5_5Y1,CVB5_5Y
novel_circ_0013115	-	ENSG00000138801;	CVB5_5Y1,CVB5_5Y
novel_circ_0013412	-	ENSG00000145439;	CVB5_5Y1,CVB5_5Y
novel_circ_0013060	-	ENSG00000057019;	CVB5_5Y1,CVB5_5Y
novel_circ_0018569	-	ENSG00000196597;	CVB5_5Y1,CVB5_5Y
hsa_circ_0091508	+	ENSG00000085185;	CVB5_5Y1,CVB5_5Y
hsa_circ_0001917	-	ENSG00000147044;	CVB5_5Y1,CVB5_5Y
hsa_circ_0002628	+	ENSG00000174437;	CVB5_5Y1,CVB5_5Y

hsa_circ_0003641	+	ENSG00000149311;	CVB5_5Y1,CVB5_5Y
hsa_circ_0008542	-	ENSG00000109118;	CVB5_5Y1,CVB5_5Y
hsa_circ_0002496	-	ENSG00000062725;	CVB5_5Y1,CVB5_5Y
hsa_circ_0004241	-	ENSG00000161542;	CVB5_5Y1,CVB5_5Y
hsa_circ_0003592	+	ENSG00000007545;	CVB5_5Y1,CVB5_5Y
hsa_circ_0041103	+	ENSG00000141002;	CVB5_5Y1,CVB5_5Y
hsa_circ_0005387	-	ENSG00000128731;	CVB5_5Y1,CVB5_5Y
hsa_circ_0007349	+	ENSG00000140455;	CVB5_5Y1,CVB5_5Y
hsa_circ_0003651	-	ENSG00000103591;	CVB5_5Y1,CVB5_5Y
hsa_circ_0004965	+	ENSG00000256053;	CVB5_5Y1,CVB5_5Y
hsa_circ_0031433	-	ENSG00000196792;	CVB5_5Y1,CVB5_5Y
hsa_circ_0004066	-	ENSG00000105705;	CVB5_5Y1,CVB5_5Y
hsa_circ_0005188	+	ENSG00000134815;	CVB5_5Y1,CVB5_5Y
hsa_circ_0063411	+	ENSG00000100354;	CVB5_5Y1,CVB5_5Y
hsa_circ_0004052	+	ENSG00000101104;	CVB5_5Y1,CVB5_5Y
hsa_circ_0008628	-	ENSG00000091127;	CVB5_5Y1,CVB5_5Y
hsa_circ_0006565	+	ENSG00000055130;	CVB5_5Y1,CVB5_5Y
hsa_circ_0002808	-	ENSG00000006715;	CVB5_5Y1,CVB5_5Y
hsa_circ_0002512	-	ENSG00000035499;	CVB5_5Y1,CVB5_5Y
hsa_circ_0007747	-	ENSG00000237187;	CVB5_5Y1,CVB5_5Y
hsa_circ_0006007	-	ENSG00000109323;	CVB5_5Y1,CVB5_5Y
hsa_circ_0069396	-	ENSG00000047365;	CVB5_5Y1,CVB5_5Y
hsa_circ_0008547	-	ENSG00000163697;	CVB5_5Y1,CVB5_5Y
hsa_circ_0003036	+	ENSG00000180370;	CVB5_5Y1,CVB5_5Y
hsa_circ_0004609	-	ENSG00000114316;	CVB5_5Y1,CVB5_5Y
hsa_circ_0001316	-	ENSG00000163946;	CVB5_5Y1,CVB5_5Y
hsa_circ_0002193	-	ENSG00000198648;	CVB5_5Y1,CVB5_5Y
hsa_circ_0005952	-	ENSG00000055917;	CVB5_5Y1,CVB5_5Y
hsa_circ_0003926	-	ENSG00000221978;	CVB5_5Y1,CVB5_5Y
hsa_circ_0000008	-	ENSG00000078369;	CVB5_5Y1,CVB5_5Y
hsa_circ_0007120	+	ENSG00000157933;	CVB5_5Y1,CVB5_5Y
hsa_circ_0010906	-	ENSG00000185436;	CVB5_5Y1,CVB5_5Y
hsa_circ_0000034	+	ENSG00000117682;	CVB5_5Y1,CVB5_5Y
hsa_circ_0007356	-	ENSG00000186638;	CVB5_5Y1,CVB5_5Y
hsa_circ_0005460	+	ENSG00000165304;	CVB5_5Y1,CVB5_5Y
hsa_circ_0005517	+	ENSG00000165304;	CVB5_5Y1,CVB5_5Y
novel_circ_0018654	-	ENSG00000125676;	CVB5_5Y1,CVB5_5Y
novel_circ_0002949	+	ENSG00000122034;	CVB5_5Y1,CVB5_5Y
novel_circ_0003077	-	ENSG00000123200;	CVB5_5Y1,CVB5_5Y
novel_circ_0000445	-	ENSG00000107890;	CVB5_5Y1,CVB5_5Y
novel_circ_0004759	+	ENSG00000090905;	CVB5_5Y1,CVB5_5Y
novel_circ_0003412	-	ENSG00000254505;ENSG0	CVB5_5Y1,CVB5_5Y
novel_circ_0003685	+	ENSG00000054654;	CVB5_5Y1,CVB5_5Y
novel_circ_0006785	+	ENSG00000167491;	CVB5_5Y1,CVB5_5Y
novel_circ_0006470	-	ENSG00000167306;	CVB5_5Y1,CVB5_5Y
novel_circ_0009833	-	ENSG00000233080;	CVB5_5Y1,CVB5_5Y
novel_circ_0009617	-	ENSG00000185658;	CVB5_5Y1,CVB5_5Y
novel_circ_0015185	-	ENSG00000186625;	CVB5_5Y1,CVB5_5Y
novel_circ_0015778	+	ENSG00000118412;	CVB5_5Y1,CVB5_5Y
novel_circ_0010629	+	ENSG00000055044;	CVB5_5Y1,CVB5_5Y
novel_circ_0010787	+	n/a	CVB5_5Y1,CVB5_5Y
novel_circ_0007412	+	ENSG00000143569;	CVB5_5Y1,CVB5_5Y
novel_circ_0008377	+	ENSG00000142611;	CVB5_5Y1,CVB5_5Y
hsa_circ_0029898	+	ENSG00000132952;	CVB5_5Y1,CVB5_5Y
hsa_circ_0002485	+	ENSG00000166377;	CVB5_5Y1,CVB5_5Y
hsa_circ_0060238	-	ENSG00000080839;	CVB5_5Y1,CVB5_5Y
hsa_circ_0007507	+	ENSG00000145715;	CVB5_5Y1,CVB5_5Y
hsa_circ_0001337	+	ENSG00000075785;	CVB5_5Y1,CVB5_5Y

hsa_circ_0004581	-	ENSG00000114316;	CVB5_5Y1,CVB5_5Y
hsa_circ_0007868	+	ENSG00000031698;	CVB5_5Y1,CVB5_5Y
hsa_circ_0006760	+	ENSG00000143363;	CVB5_5Y1,CVB5_5Y
hsa_circ_0083756	-	ENSG00000104228;	CVB5_5Y1,CVB5_5Y
novel_circ_0001576	+	ENSG00000110075;	CVB5_5Y1,CVB5_5Y
novel_circ_0000961	-	ENSG00000077147;	CVB5_5Y1,CVB5_5Y
novel_circ_0009846	-	ENSG00000100353;	CVB5_5Y1,CVB5_5Y
novel_circ_0012004	-	ENSG00000114686;	CVB5_5Y1,CVB5_5Y
novel_circ_0013026	+	ENSG00000134077;	CVB5_5Y1,CVB5_5Y
novel_circ_0010097	+	ENSG00000114999;	CVB5_5Y1,CVB5_5Y
hsa_circ_0001932	-	ENSG00000085224;	CVB5_5Y1,CVB5_5Y
hsa_circ_0007044	+	ENSG00000198431;	CVB5_5Y1,CVB5_5Y
hsa_circ_0002783	-	ENSG00000196498;	CVB5_5Y1,CVB5_5Y
hsa_circ_0002357	+	ENSG00000110841;	CVB5_5Y1,CVB5_5Y
hsa_circ_0024946	-	ENSG00000073614;	CVB5_5Y1,CVB5_5Y
hsa_circ_0021843	-	ENSG00000110497;	CVB5_5Y1,CVB5_5Y
hsa_circ_0008251	-	ENSG00000165322;	CVB5_5Y1,CVB5_5Y
hsa_circ_0003605	+	ENSG00000108175;	CVB5_5Y1,CVB5_5Y
hsa_circ_0003258	-	ENSG00000198740;	CVB5_5Y1,CVB5_5Y
hsa_circ_0046419	+	ENSG00000141562;	CVB5_5Y1,CVB5_5Y
hsa_circ_0038132	+	ENSG00000103222;	CVB5_5Y1,CVB5_5Y
hsa_circ_0005818	+	ENSG00000020426;	CVB5_5Y1,CVB5_5Y
hsa_circ_0050247	-	ENSG00000105717;	CVB5_5Y1,CVB5_5Y
hsa_circ_0000832	+	ENSG00000101639;	CVB5_5Y1,CVB5_5Y
hsa_circ_0062948	+	ENSG00000100150;ENSG0	CVB5_5Y1,CVB5_5Y
hsa_circ_0001172	-	ENSG00000124224;	CVB5_5Y1,CVB5_5Y
hsa_circ_0005515	+	ENSG00000135164;	CVB5_5Y1,CVB5_5Y
hsa_circ_0005451	+	ENSG00000170734;	CVB5_5Y1,CVB5_5Y
hsa_circ_0070590	-	ENSG00000145348;	CVB5_5Y1,CVB5_5Y
hsa_circ_0005994	-	ENSG00000135749;	CVB5_5Y1,CVB5_5Y
hsa_circ_0088494	+	ENSG00000119408;	CVB5_5Y1,CVB5_5Y
novel_circ_0002116	+	ENSG00000184445;	CVB5_5Y1,CVB5_5Y
novel_circ_0000513	-	ENSG00000170759;	CVB5_5Y1,CVB5_5Y
novel_circ_0000850	-	ENSG00000062650;	CVB5_5Y1,CVB5_5Y
novel_circ_0006738	+	ENSG00000167460;	CVB5_5Y1,CVB5_5Y
novel_circ_0011779	-	ENSG00000066422;	CVB5_5Y1,CVB5_5Y
novel_circ_0012490	+	ENSG00000122068;	CVB5_5Y1,CVB5_5Y
novel_circ_0007766	+	ENSG00000116747;	CVB5_5Y1,CVB5_5Y
novel_circ_0017470	-	ENSG00000198363;	CVB5_5Y1,CVB5_5Y
hsa_circ_0006628	-	ENSG00000107745;	CVB5_5Y1,CVB5_5Y
hsa_circ_0043244	-	ENSG00000278540;	CVB5_5Y1,CVB5_5Y
hsa_circ_0006886	-	ENSG00000172775;	CVB5_5Y1,CVB5_5Y
hsa_circ_0000532	+	ENSG00000185246;	CVB5_5Y1,CVB5_5Y
hsa_circ_0001236	+	ENSG00000100403;	CVB5_5Y1,CVB5_5Y
hsa_circ_0067230	-	ENSG00000163902;	CVB5_5Y1,CVB5_5Y
hsa_circ_0007344	-	ENSG00000115761;	CVB5_5Y1,CVB5_5Y
hsa_circ_0013644	-	ENSG00000009307;	CVB5_5Y1,CVB5_5Y
hsa_circ_0012964	+	ENSG00000117054;	CVB5_5Y1,CVB5_5Y
novel_circ_0002865	-	ENSG00000121390;	CVB5_5Y1,CVB5_5Y
novel_circ_0002703	-	ENSG00000177425;	CVB5_5Y1,CVB5_5Y
novel_circ_0002737	-	ENSG00000049130;	CVB5_5Y1,CVB5_5Y
novel_circ_0007170	-	ENSG00000171466;	CVB5_5Y1,CVB5_5Y
novel_circ_0009909	+	ENSG00000100393;	CVB5_5Y1,CVB5_5Y
novel_circ_0015896	-	ENSG00000091127;	CVB5_5Y1,CVB5_5Y
novel_circ_0016793	-	ENSG00000106244;	CVB5_5Y1,CVB5_5Y
novel_circ_0014422	+	ENSG00000165671;	CVB5_5Y1,CVB5_5Y
hsa_circ_0004278	+	ENSG00000090889;	CVB5_5Y1,CVB5_5Y
hsa_circ_0003133	-	ENSG00000084112;	CVB5_5Y1,CVB5_5Y

hsa_circ_0041871	+	ENSG00000181222;	CVB5_5Y1,CVB5_5Y
hsa_circ_0035875	-	ENSG00000090487;	CVB5_5Y1,CVB5_5Y
hsa_circ_0004578	+	ENSG00000101752;	CVB5_5Y1,CVB5_5Y
hsa_circ_0003032	+	ENSG00000151422;	CVB5_5Y1,CVB5_5Y
hsa_circ_0057703	+	ENSG00000196141;	CVB5_5Y1,CVB5_5Y
hsa_circ_0004916	+	ENSG00000285542;ENSG0	CVB5_5Y1,CVB5_5Y
hsa_circ_0002321	+	ENSG00000066027;	CVB5_5Y1,CVB5_5Y
novel_circ_0002476	+	ENSG00000161813;	CVB5_5Y1,CVB5_5Y
novel_circ_0001767	+	ENSG00000284057;ENSG0	CVB5_5Y1,CVB5_5Y
novel_circ_0005208	-	ENSG00000167522;	CVB5_5Y1,CVB5_5Y
novel_circ_0004221	-	ENSG00000140299;	CVB5_5Y1,CVB5_5Y
novel_circ_0015624	+	ENSG00000118482;	CVB5_5Y1,CVB5_5Y
novel_circ_0014278	-	ENSG00000186314;	CVB5_5Y1,CVB5_5Y
novel_circ_0014603	+	ENSG00000039123;	CVB5_5Y1,CVB5_5Y
novel_circ_0013076	-	ENSG00000138814;	CVB5_5Y1,CVB5_5Y
novel_circ_0018183	+	ENSG00000099810;	CVB5_5Y1,CVB5_5Y
hsa_circ_0029974	+	ENSG00000172915;	CVB5_5Y2
hsa_circ_0030507	-	ENSG00000005810;	CVB5_5Y2
hsa_circ_0006871	-	ENSG00000125257;	CVB5_5Y2
hsa_circ_0008092	-	ENSG00000110880;	CVB5_5Y2
hsa_circ_0007830	-	ENSG00000110880;	CVB5_5Y2
hsa_circ_0028282	-	ENSG00000089234;	CVB5_5Y2
hsa_circ_0005005	-	ENSG00000089154;	CVB5_5Y2
hsa_circ_0029192	-	ENSG00000051825;	CVB5_5Y2
hsa_circ_0025042	-	ENSG00000111206;	CVB5_5Y2
hsa_circ_0026302	-	ENSG00000110925;	CVB5_5Y2
hsa_circ_0020749	+	ENSG00000183020;	CVB5_5Y2
hsa_circ_0024234	+	ENSG00000149311;	CVB5_5Y2
hsa_circ_0020764	-	ENSG00000078902;	CVB5_5Y2
hsa_circ_0024876	+	ENSG00000149418;	CVB5_5Y2
hsa_circ_0024920	-	ENSG00000151503;	CVB5_5Y2
hsa_circ_0021569	+	ENSG00000060749;	CVB5_5Y2
hsa_circ_0000299	+	ENSG00000134574;	CVB5_5Y2
hsa_circ_0022007	-	ENSG00000149187;	CVB5_5Y2
hsa_circ_0023321	+	ENSG00000131626;	CVB5_5Y2
hsa_circ_0023628	+	ENSG00000149257;	CVB5_5Y2
hsa_circ_0003661	+	ENSG00000137494;	CVB5_5Y2
hsa_circ_0004117	-	ENSG00000120029;	CVB5_5Y2
hsa_circ_0003779	+	ENSG00000107882;	CVB5_5Y2
hsa_circ_0019773	+	ENSG00000270316;ENSG0	CVB5_5Y2
hsa_circ_0020024	+	ENSG00000150593;	CVB5_5Y2
hsa_circ_0000261	+	ENSG00000108061;	CVB5_5Y2
hsa_circ_0005312	+	ENSG00000107651;	CVB5_5Y2
hsa_circ_0020381	+	ENSG00000150760;	CVB5_5Y2
hsa_circ_0006750	+	ENSG00000108239;	CVB5_5Y2
hsa_circ_0002723	-	ENSG00000108953;	CVB5_5Y2
hsa_circ_0042250	-	ENSG00000141030;	CVB5_5Y2
hsa_circ_0008652	-	ENSG00000177302;	CVB5_5Y2
hsa_circ_0042364	+	ENSG00000141127;	CVB5_5Y2
hsa_circ_0042733	-	ENSG00000109118;	CVB5_5Y2
hsa_circ_0004800	+	ENSG00000160551;	CVB5_5Y2
hsa_circ_0043750	-	ENSG00000168259;	CVB5_5Y2
hsa_circ_0043893	-	ENSG00000108799;	CVB5_5Y2
hsa_circ_0008586	+	ENSG00000108424;	CVB5_5Y2
hsa_circ_0009137	+	ENSG00000029725;	CVB5_5Y2
hsa_circ_0045862	+	ENSG00000078687;	CVB5_5Y2
hsa_circ_0045904	+	ENSG00000087157;	CVB5_5Y2
hsa_circ_0046128	-	ENSG00000157637;	CVB5_5Y2

hsa_circ_0003430	-	ENSG00000176155;	CVB5_5Y2
hsa_circ_0038085	+	ENSG00000103222;	CVB5_5Y2
hsa_circ_0038244	-	ENSG00000157106;	CVB5_5Y2
hsa_circ_0038942	+	ENSG00000013364;	CVB5_5Y2
hsa_circ_0039100	+	ENSG00000282034;ENSG0	CVB5_5Y2
hsa_circ_0037737	+	ENSG00000102858;	CVB5_5Y2
hsa_circ_0009167	-	ENSG00000183723;	CVB5_5Y2
hsa_circ_0003220	+	ENSG00000103047;	CVB5_5Y2
hsa_circ_0002521	-	ENSG00000214331;	CVB5_5Y2
hsa_circ_0040496	-	ENSG00000168411;	CVB5_5Y2
hsa_circ_0037078	+	ENSG00000154237;	CVB5_5Y2
hsa_circ_0035861	+	ENSG00000166839;	CVB5_5Y2
hsa_circ_0036356	-	ENSG00000169375;	CVB5_5Y2
hsa_circ_0033191	-	ENSG00000140105;	CVB5_5Y2
hsa_circ_0033424	+	ENSG00000131323;	CVB5_5Y2
hsa_circ_0033431	+	ENSG00000131323;	CVB5_5Y2
hsa_circ_0009051	-	ENSG00000198554;	CVB5_5Y2
hsa_circ_0000546	-	ENSG00000072121;	CVB5_5Y2
hsa_circ_0032464	+	ENSG00000197555;	CVB5_5Y2
hsa_circ_0032651	-	ENSG00000119638;	CVB5_5Y2
hsa_circ_0006149	-	ENSG00000079999;	CVB5_5Y2
hsa_circ_0049330	+	ENSG00000079805;	CVB5_5Y2
hsa_circ_0049792	-	ENSG00000127507;	CVB5_5Y2
hsa_circ_0049998	+	ENSG00000130313;	CVB5_5Y2
hsa_circ_0000925	-	ENSG00000121289;	CVB5_5Y2
hsa_circ_0051081	-	ENSG00000105221;	CVB5_5Y2
hsa_circ_0004952	-	ENSG00000160392;	CVB5_5Y2
hsa_circ_0003061	-	ENSG00000090013;	CVB5_5Y2
hsa_circ_0051494	-	ENSG00000012061;	CVB5_5Y2
hsa_circ_0048687	+	ENSG00000127663;	CVB5_5Y2
hsa_circ_0002941	+	ENSG00000125733;	CVB5_5Y2
hsa_circ_0047125	+	ENSG00000101752;	CVB5_5Y2
hsa_circ_0047285	+	ENSG00000168234;	CVB5_5Y2
hsa_circ_0047473	+	ENSG00000150477;	CVB5_5Y2
hsa_circ_0047478	+	ENSG00000150477;	CVB5_5Y2
hsa_circ_0047594	-	ENSG00000078043;	CVB5_5Y2
hsa_circ_0047676	-	ENSG00000167306;	CVB5_5Y2
hsa_circ_0046861	+	ENSG00000017797;	CVB5_5Y2
hsa_circ_0062563	+	ENSG00000099956;	CVB5_5Y2
hsa_circ_0062610	+	ENSG00000100014;ENSG0	CVB5_5Y2
hsa_circ_0006612	+	ENSG00000101310;	CVB5_5Y2
hsa_circ_0005759	+	ENSG00000118705;	CVB5_5Y2
hsa_circ_0001163	-	ENSG00000101040;	CVB5_5Y2
hsa_circ_0060874	-	ENSG00000054793;	CVB5_5Y2
hsa_circ_0001186	+	ENSG00000243927;	CVB5_5Y2
hsa_circ_0062003	+	ENSG00000197381;	CVB5_5Y2
hsa_circ_0006046	+	ENSG00000160299;	CVB5_5Y2
hsa_circ_0005705	-	ENSG00000091127;	CVB5_5Y2
hsa_circ_0007102	+	ENSG00000136261;	CVB5_5Y2
hsa_circ_0079201	+	ENSG00000106012;	CVB5_5Y2
hsa_circ_0006073	+	ENSG00000106052;	CVB5_5Y2
hsa_circ_0079818	+	ENSG00000122507;	CVB5_5Y2
hsa_circ_0079940	+	ENSG00000065883;	CVB5_5Y2
hsa_circ_0081309	+	ENSG00000196367;	CVB5_5Y2
hsa_circ_0075658	-	ENSG00000010017;	CVB5_5Y2
hsa_circ_0078217	-	ENSG00000186625;	CVB5_5Y2
hsa_circ_0006793	+	ENSG00000120254;	CVB5_5Y2
hsa_circ_0076157	-	ENSG00000096060;	CVB5_5Y2

hsa_circ_0076710	-	ENSG00000244694;	CVB5_5Y2
hsa_circ_0008924	-	ENSG00000151914;	CVB5_5Y2
hsa_circ_0008325	-	ENSG00000112210;	CVB5_5Y2
hsa_circ_0075561	+	ENSG00000124782;	CVB5_5Y2
hsa_circ_0077422	-	ENSG00000132424;	CVB5_5Y2
hsa_circ_0073772	+	ENSG00000064651;	CVB5_5Y2
hsa_circ_0003180	+	ENSG00000113615;	CVB5_5Y2
hsa_circ_0073993	+	ENSG00000113615;	CVB5_5Y2
hsa_circ_0073994	+	ENSG00000113615;	CVB5_5Y2
hsa_circ_0074069	-	ENSG00000031003;	CVB5_5Y2
hsa_circ_0006995	-	ENSG00000113013;	CVB5_5Y2
hsa_circ_0006674	+	ENSG00000038382;	CVB5_5Y2
hsa_circ_0074391	+	ENSG00000156463;	CVB5_5Y2
hsa_circ_0074854	+	ENSG00000038274;	CVB5_5Y2
hsa_circ_0008047	+	ENSG00000157107;	CVB5_5Y2
hsa_circ_0073132	-	ENSG00000152413;	CVB5_5Y2
hsa_circ_0004700	+	ENSG00000198856;	CVB5_5Y2
hsa_circ_0071291	+	ENSG00000109654;	CVB5_5Y2
hsa_circ_0006225	+	ENSG00000121211;	CVB5_5Y2
hsa_circ_0006366	-	ENSG00000154447;	CVB5_5Y2
hsa_circ_0069837	+	ENSG00000174780;	CVB5_5Y2
hsa_circ_0070097	+	ENSG00000138759;	CVB5_5Y2
hsa_circ_0066846	-	ENSG00000181722;	CVB5_5Y2
hsa_circ_0067594	-	ENSG00000114126;	CVB5_5Y2
hsa_circ_0007306	+	ENSG00000175166;	CVB5_5Y2
hsa_circ_0001377	-	ENSG00000061938;	CVB5_5Y2
hsa_circ_0065259	-	ENSG00000173473;	CVB5_5Y2
hsa_circ_0007994	-	ENSG00000173473;	CVB5_5Y2
hsa_circ_0065347	-	ENSG00000047849;	CVB5_5Y2
hsa_circ_0007196	+	ENSG00000003756;	CVB5_5Y2
hsa_circ_0006226	+	ENSG00000144724;	CVB5_5Y2
hsa_circ_0007250	-	ENSG00000143870;	CVB5_5Y2
hsa_circ_0007186	+	ENSG00000144118;	CVB5_5Y2
hsa_circ_0005533	-	ENSG00000151779;	CVB5_5Y2
hsa_circ_0057168	-	ENSG00000116044;	CVB5_5Y2
hsa_circ_0002087	-	ENSG00000187231;	CVB5_5Y2
hsa_circ_0058565	-	ENSG00000153827;	CVB5_5Y2
hsa_circ_0006391	-	ENSG00000115687;	CVB5_5Y2
hsa_circ_0053035	-	ENSG00000138101;	CVB5_5Y2
hsa_circ_0053343	+	ENSG00000163811;	CVB5_5Y2
hsa_circ_0053394	-	ENSG00000162961;ENSG0	CVB5_5Y2
hsa_circ_0000993	-	ENSG00000119787;	CVB5_5Y2
hsa_circ_0054706	+	ENSG00000162929;	CVB5_5Y2
hsa_circ_0001021	+	ENSG00000119844;	CVB5_5Y2
hsa_circ_0006251	+	ENSG00000187605;	CVB5_5Y2
hsa_circ_0055426	-	ENSG00000115486;	CVB5_5Y2
hsa_circ_0009682	+	ENSG00000162444;	CVB5_5Y2
hsa_circ_0009732	+	ENSG00000142657;	CVB5_5Y2
hsa_circ_0010024	-	ENSG00000162496;	CVB5_5Y2
hsa_circ_0006851	+	ENSG00000143379;	CVB5_5Y2
hsa_circ_0014558	-	ENSG00000116539;	CVB5_5Y2
hsa_circ_0000140	-	ENSG00000132680;	CVB5_5Y2
hsa_circ_0010048	+	ENSG00000197312;	CVB5_5Y2
hsa_circ_0014824	-	ENSG00000132694;	CVB5_5Y2
hsa_circ_0015211	+	ENSG00000117523;	CVB5_5Y2
hsa_circ_0015211	+	ENSG00000117523;	CVB5_5Y2
hsa_circ_0007538	+	ENSG00000157181;	CVB5_5Y2
hsa_circ_0010536	-	ENSG00000090686;	CVB5_5Y2

hsa_circ_0002720	+	ENSG00000133216;	CVB5_5Y2
hsa_circ_0004802	+	ENSG00000117602;	CVB5_5Y2
hsa_circ_0011946	-	ENSG00000010803;	CVB5_5Y2
hsa_circ_0012363	-	ENSG00000123473;	CVB5_5Y2
hsa_circ_0012460	+	ENSG00000117859;	CVB5_5Y2
hsa_circ_0006676	-	ENSG00000058804;	CVB5_5Y2
hsa_circ_0012732	-	ENSG00000162601;	CVB5_5Y2
hsa_circ_0009527	-	ENSG00000162408;	CVB5_5Y2
hsa_circ_0089078	+	ENSG00000148358;	CVB5_5Y2
hsa_circ_0089131	+	ENSG00000097007;	CVB5_5Y2
hsa_circ_0002407	+	ENSG00000197724;	CVB5_5Y2
hsa_circ_0002796	+	ENSG00000197816;ENSG0	CVB5_5Y2
hsa_circ_0002125	+	ENSG00000131773;	CVB5_5Y2
hsa_circ_0083270	+	ENSG00000104728;ENSG0	CVB5_5Y2
hsa_circ_0002131	+	ENSG00000104765;	CVB5_5Y2
hsa_circ_0008397	+	ENSG00000168522;	CVB5_5Y2
hsa_circ_0084281	-	ENSG00000253729;	CVB5_5Y2
hsa_circ_0084336	-	ENSG00000253729;	CVB5_5Y2
hsa_circ_0002576	-	ENSG00000251136;	CVB5_5Y2
hsa_circ_0008608	-	ENSG00000175305;	CVB5_5Y2
novel_circ_0018769	-	ENSG00000180815;	CVB5_5Y2
novel_circ_0018906	-	ENSG00000085224;	CVB5_5Y2
novel_circ_0018908	-	ENSG00000085224;	CVB5_5Y2
novel_circ_0018912	+	ENSG00000165240;	CVB5_5Y2
novel_circ_0018926	+	ENSG00000101849;	CVB5_5Y2
novel_circ_0002832	+	ENSG00000139842;	CVB5_5Y2
novel_circ_0002897	-	ENSG00000132953;	CVB5_5Y2
novel_circ_0003196	-	ENSG00000139746;	CVB5_5Y2
novel_circ_0003237	-	ENSG00000102595;	CVB5_5Y2
novel_circ_0001833	-	ENSG00000075188;	CVB5_5Y2
novel_circ_0001850	-	ENSG00000120837;	CVB5_5Y2
novel_circ_0001861	+	ENSG00000198431;	CVB5_5Y2
novel_circ_0001979	-	ENSG00000111300;	CVB5_5Y2
novel_circ_0002008	-	ENSG00000111344;	CVB5_5Y2
novel_circ_0002085	-	ENSG00000212694;ENSG0	CVB5_5Y2
novel_circ_0002087	+	ENSG00000110801;	CVB5_5Y2
novel_circ_0002089	+	ENSG00000110987;	CVB5_5Y2
novel_circ_0002099	-	ENSG00000130779;	CVB5_5Y2
novel_circ_0002145	-	ENSG00000139697;	CVB5_5Y2
novel_circ_0002289	-	ENSG00000064115;	CVB5_5Y2
novel_circ_0002455	+	ENSG00000123352;	CVB5_5Y2
novel_circ_0002486	+	ENSG00000066084;	CVB5_5Y2
novel_circ_0002565	+	ENSG00000061987;	CVB5_5Y2
novel_circ_0002573	+	ENSG00000196935;	CVB5_5Y2
novel_circ_0002754	-	ENSG00000102189;	CVB5_5Y2
novel_circ_0001008	-	ENSG00000137692;	CVB5_5Y2
novel_circ_0001029	+	ENSG00000177112;	CVB5_5Y2
novel_circ_0001052	+	ENSG00000285696;	CVB5_5Y2
novel_circ_0001114	-	ENSG00000137656;	CVB5_5Y2
novel_circ_0001527	-	ENSG00000168056;	CVB5_5Y2
novel_circ_0001547	-	ENSG00000177030;	CVB5_5Y2
novel_circ_0001580	+	ENSG00000110075;	CVB5_5Y2
novel_circ_0001743	+	ENSG00000254860;	CVB5_5Y2
novel_circ_0001760	+	ENSG00000166004;	CVB5_5Y2
novel_circ_0000036	-	ENSG00000198408;	CVB5_5Y2
novel_circ_0000066	+	ENSG00000107862;	CVB5_5Y2
novel_circ_0000135	+	n/a	CVB5_5Y2
novel_circ_0000139	+	ENSG00000148737;	CVB5_5Y2

novel_circ_0000176	-	ENSG00000107560;	CVB5_5Y2
novel_circ_0000177	+	ENSG00000148426;	CVB5_5Y2
novel_circ_0000179	-	ENSG00000151893;	CVB5_5Y2
novel_circ_0000204	-	ENSG00000151923;	CVB5_5Y2
novel_circ_0000360	+	ENSG00000065328;	CVB5_5Y2
novel_circ_0000378	-	ENSG00000152464;ENSG0	CVB5_5Y2
novel_circ_0000441	-	ENSG00000136754;	CVB5_5Y2
novel_circ_0000558	-	ENSG00000151240;	CVB5_5Y2
novel_circ_0000571	-	ENSG00000172671;	CVB5_5Y2
novel_circ_0000573	-	ENSG00000172671;	CVB5_5Y2
novel_circ_0000705	-	ENSG00000138346;	CVB5_5Y2
novel_circ_0000907	-	ENSG00000119912;	CVB5_5Y2
novel_circ_0000921	-	ENSG00000138119;	CVB5_5Y2
novel_circ_0000934	-	ENSG00000173145;	CVB5_5Y2
novel_circ_0000936	+	ENSG00000108239;	CVB5_5Y2
novel_circ_0000955	+	ENSG00000177853;	CVB5_5Y2
novel_circ_0005332	+	ENSG00000141127;	CVB5_5Y2
novel_circ_0005395	+	ENSG00000109111;	CVB5_5Y2
novel_circ_0005415	+	ENSG00000126653;	CVB5_5Y2
novel_circ_0005549	+	ENSG00000108344;	CVB5_5Y2
novel_circ_0005659	-	ENSG00000141456;	CVB5_5Y2
novel_circ_0005809	-	ENSG00000108395;	CVB5_5Y2
novel_circ_0005970	-	ENSG00000198920;	CVB5_5Y2
novel_circ_0005997	+	ENSG00000171634;	CVB5_5Y2
novel_circ_0006074	-	ENSG00000070495;	CVB5_5Y2
novel_circ_0006135	-	ENSG00000182446;	CVB5_5Y2
novel_circ_0004648	+	ENSG00000072864;	CVB5_5Y2
novel_circ_0004735	+	ENSG00000103319;	CVB5_5Y2
novel_circ_0004834	+	ENSG00000213918;	CVB5_5Y2
novel_circ_0004859	-	ENSG00000171241;	CVB5_5Y2
novel_circ_0004905	+	ENSG00000121274;	CVB5_5Y2
novel_circ_0004937	+	ENSG00000102900;	CVB5_5Y2
novel_circ_0005048	+	ENSG00000189091;	CVB5_5Y2
novel_circ_0005049	+	ENSG00000189091;	CVB5_5Y2
novel_circ_0005051	+	ENSG00000189091;	CVB5_5Y2
novel_circ_0003961	-	ENSG00000185418;	CVB5_5Y2
novel_circ_0004061	-	ENSG00000128908;	CVB5_5Y2
novel_circ_0004195	+	ENSG00000069943;	CVB5_5Y2
novel_circ_0004463	+	ENSG00000140403;	CVB5_5Y2
novel_circ_0004486	-	ENSG00000117899;	CVB5_5Y2
novel_circ_0004542	+	ENSG00000140575;	CVB5_5Y2
novel_circ_0004547	-	ENSG00000284946;ENSG0	CVB5_5Y2
novel_circ_0003276	+	ENSG00000176473;	CVB5_5Y2
novel_circ_0003368	+	ENSG00000066735;	CVB5_5Y2
novel_circ_0003626	-	ENSG00000198554;	CVB5_5Y2
novel_circ_0003629	-	ENSG00000198554;	CVB5_5Y2
novel_circ_0003656	-	ENSG00000100612;	CVB5_5Y2
novel_circ_0003922	+	ENSG00000100749;	CVB5_5Y2
novel_circ_0006762	+	ENSG00000130479;	CVB5_5Y2
novel_circ_0006845	-	ENSG00000105186;	CVB5_5Y2
novel_circ_0007041	+	ENSG00000126464;	CVB5_5Y2
novel_circ_0007044	+	ENSG00000104973;	CVB5_5Y2
novel_circ_0007088	-	ENSG00000167608;	CVB5_5Y2
novel_circ_0007107	+	ENSG00000160633;	CVB5_5Y2
novel_circ_0006383	+	ENSG00000101596;	CVB5_5Y2
novel_circ_0006416	+	ENSG00000075643;	CVB5_5Y2
novel_circ_0009824	+	ENSG00000242082;	CVB5_5Y2
novel_circ_0009072	-	ENSG00000089048;	CVB5_5Y2

novel_circ_0009276	+	n/a	CVB5_5Y2
novel_circ_0009325	-	ENSG00000198026;	CVB5_5Y2
novel_circ_0009358	+	ENSG00000124207;	CVB5_5Y2
novel_circ_0009360	+	ENSG00000124207;	CVB5_5Y2
novel_circ_0009379	-	ENSG00000124201;	CVB5_5Y2
novel_circ_0009464	-	ENSG00000101191;	CVB5_5Y2
novel_circ_0009499	+	ENSG00000155313;	CVB5_5Y2
novel_circ_0009577	+	ENSG00000159256;	CVB5_5Y2
novel_circ_0009665	-	ENSG00000186866;	CVB5_5Y2
novel_circ_0009666	+	ENSG00000223768;	CVB5_5Y2
novel_circ_0015857	+	ENSG00000105819;ENSG0	CVB5_5Y2
novel_circ_0016026	+	ENSG00000131558;	CVB5_5Y2
novel_circ_0016042	-	ENSG00000122778;	CVB5_5Y2
novel_circ_0016060	-	ENSG00000064393;	CVB5_5Y2
novel_circ_0016082	-	ENSG00000146904;	CVB5_5Y2
novel_circ_0016309	-	ENSG00000070882;	CVB5_5Y2
novel_circ_0016328	+	ENSG00000106052;	CVB5_5Y2
novel_circ_0016338	-	ENSG00000136193;	CVB5_5Y2
novel_circ_0016526	-	ENSG00000169919;	CVB5_5Y2
novel_circ_0016721	+	ENSG00000127914;	CVB5_5Y2
novel_circ_0015167	-	ENSG00000146414;	CVB5_5Y2
novel_circ_0015215	+	ENSG00000131016;	CVB5_5Y2
novel_circ_0015436	-	ENSG00000204463;	CVB5_5Y2
novel_circ_0015533	+	ENSG00000137221;	CVB5_5Y2
novel_circ_0015699	-	ENSG00000005700;	CVB5_5Y2
novel_circ_0014106	-	ENSG00000273217;ENSG0	CVB5_5Y2
novel_circ_0014276	-	ENSG00000186314;	CVB5_5Y2
novel_circ_0014292	+	ENSG00000091009;	CVB5_5Y2
novel_circ_0014315	+	ENSG00000145907;	CVB5_5Y2
novel_circ_0014518	-	ENSG00000197603;	CVB5_5Y2
novel_circ_0014520	-	ENSG00000113569;	CVB5_5Y2
novel_circ_0014539	+	ENSG00000082068;	CVB5_5Y2
novel_circ_0014778	-	ENSG00000132842;	CVB5_5Y2
novel_circ_0014787	+	ENSG00000085365;	CVB5_5Y2
novel_circ_0014809	-	ENSG00000164300;	CVB5_5Y2
novel_circ_0013099	-	ENSG00000138777;	CVB5_5Y2
novel_circ_0013104	-	ENSG00000236699;ENSG0	CVB5_5Y2
novel_circ_0013312	+	ENSG00000071205;	CVB5_5Y2
novel_circ_0013384	-	ENSG00000171497;	CVB5_5Y2
novel_circ_0013440	-	ENSG00000109576;	CVB5_5Y2
novel_circ_0013457	-	ENSG00000178177;	CVB5_5Y2
novel_circ_0013541	-	ENSG00000281501;ENSG0	CVB5_5Y2
novel_circ_0013642	+	ENSG00000078177;	CVB5_5Y2
novel_circ_0013660	-	ENSG00000163697;	CVB5_5Y2
novel_circ_0013672	-	ENSG00000145220;	CVB5_5Y2
novel_circ_0013740	+	ENSG00000145216;	CVB5_5Y2
novel_circ_0013766	+	ENSG00000013288;	CVB5_5Y2
novel_circ_0013768	-	ENSG00000033178;	CVB5_5Y2
novel_circ_0011781	-	ENSG00000066422;	CVB5_5Y2
novel_circ_0011861	-	n/a	CVB5_5Y2
novel_circ_0011910	+	ENSG00000132170;	CVB5_5Y2
novel_circ_0011945	-	ENSG00000114520;	CVB5_5Y2
novel_circ_0011958	+	ENSG00000073111;	CVB5_5Y2
novel_circ_0012415	+	ENSG00000188001;	CVB5_5Y2
novel_circ_0012488	-	ENSG00000075711;	CVB5_5Y2
novel_circ_0012491	+	ENSG00000122068;	CVB5_5Y2
novel_circ_0012493	+	ENSG00000122068;	CVB5_5Y2
novel_circ_0012595	+	ENSG00000182973;	CVB5_5Y2

novel_circ_0012733	-	ENSG00000181555;	CVB5_5Y2
novel_circ_0012780	-	ENSG00000173473;	CVB5_5Y2
novel_circ_0012838	+	ENSG00000003756;	CVB5_5Y2
novel_circ_0012907	-	ENSG00000163947;	CVB5_5Y2
novel_circ_0010063	+	ENSG00000163006;	CVB5_5Y2
novel_circ_0010078	-	n/a	CVB5_5Y2
novel_circ_0010140	+	ENSG00000144118;	CVB5_5Y2
novel_circ_0010237	-	ENSG00000076003;	CVB5_5Y2
novel_circ_0010264	+	ENSG00000080345;	CVB5_5Y2
novel_circ_0010351	+	ENSG00000136536;	CVB5_5Y2
novel_circ_0010413	-	ENSG00000198586;	CVB5_5Y2
novel_circ_0010527	-	n/a	CVB5_5Y2
novel_circ_0010816	+	ENSG00000123983;	CVB5_5Y2
novel_circ_0010967	-	ENSG00000122085;	CVB5_5Y2
novel_circ_0011037	-	ENSG00000138002;	CVB5_5Y2
novel_circ_0011154	+	ENSG00000049323;	CVB5_5Y2
novel_circ_0011225	-	ENSG00000205111;	CVB5_5Y2
novel_circ_0011235	-	ENSG00000138050;	CVB5_5Y2
novel_circ_0011415	-	ENSG00000115464;	CVB5_5Y2
novel_circ_0011436	-	ENSG00000115464;	CVB5_5Y2
novel_circ_0011649	+	ENSG00000168883;	CVB5_5Y2
novel_circ_0011686	+	ENSG00000119203;	CVB5_5Y2
novel_circ_0011748	+	ENSG00000158417;	CVB5_5Y2
novel_circ_0007261	+	ENSG00000118655;	CVB5_5Y2
novel_circ_0007263	-	ENSG00000197323;	CVB5_5Y2
novel_circ_0007375	+	ENSG00000143458;	CVB5_5Y2
novel_circ_0007502	+	ENSG00000065526;	CVB5_5Y2
novel_circ_0007691	+	ENSG00000135837;	CVB5_5Y2
novel_circ_0007728	-	ENSG00000116406;	CVB5_5Y2
novel_circ_0007767	-	ENSG00000023572;	CVB5_5Y2
novel_circ_0007940	+	ENSG00000117724;	CVB5_5Y2
novel_circ_0007971	-	ENSG00000136628;	CVB5_5Y2
novel_circ_0007975	-	ENSG00000136628;	CVB5_5Y2
novel_circ_0008108	+	ENSG00000004487;ENSG0	CVB5_5Y2
novel_circ_0008167	-	ENSG00000125944;	CVB5_5Y2
novel_circ_0008277	-	ENSG00000162851;	CVB5_5Y2
novel_circ_0008352	+	ENSG00000060656;	CVB5_5Y2
novel_circ_0008353	+	ENSG00000060656;	CVB5_5Y2
novel_circ_0008355	-	ENSG00000134644;	CVB5_5Y2
novel_circ_0008392	+	ENSG00000025800;	CVB5_5Y2
novel_circ_0008402	-	ENSG00000176261;	CVB5_5Y2
novel_circ_0008416	-	ENSG00000163867;ENSG0	CVB5_5Y2
novel_circ_0008436	-	ENSG00000142687;	CVB5_5Y2
novel_circ_0008496	+	ENSG00000117000;	CVB5_5Y2
novel_circ_0017821	-	ENSG00000136813;	CVB5_5Y2
novel_circ_0017866	-	ENSG00000095397;	CVB5_5Y2
novel_circ_0017875	-	ENSG00000136861;	CVB5_5Y2
novel_circ_0018182	+	ENSG00000099810;	CVB5_5Y2
novel_circ_0018325	-	ENSG00000107249;	CVB5_5Y2
novel_circ_0018379	+	ENSG00000119125;	CVB5_5Y2
novel_circ_0018409	-	n/a	CVB5_5Y2
novel_circ_0018455	-	ENSG00000135052;	CVB5_5Y2
novel_circ_0016833	-	ENSG00000104517;	CVB5_5Y2
novel_circ_0016856	-	ENSG00000247081;	CVB5_5Y2
novel_circ_0016981	+	ENSG00000156831;	CVB5_5Y2
novel_circ_0017236	+	ENSG00000147459;	CVB5_5Y2
novel_circ_0017463	+	ENSG00000171316;	CVB5_5Y2
novel_circ_0017614	+	ENSG00000104325;	CVB5_5Y2

novel_circ_0017643	+	ENSG00000164953;	CVB5_5Y2
hsa_circ_0006430	-	ENSG00000110713;	CVB5_5Y2,Con_5Y1
hsa_circ_0005861	-	ENSG00000133316;	CVB5_5Y2,Con_5Y1
hsa_circ_0019606	-	ENSG00000198408;	CVB5_5Y2,Con_5Y1
hsa_circ_0006610	+	ENSG00000151553;	CVB5_5Y2,Con_5Y1
hsa_circ_0005767	-	ENSG00000148484;	CVB5_5Y2,Con_5Y1
hsa_circ_0002478	-	ENSG00000057608;	CVB5_5Y2,Con_5Y1
hsa_circ_0000240	+	ENSG00000060339;	CVB5_5Y2,Con_5Y1
hsa_circ_0042986	+	ENSG00000178691;	CVB5_5Y2,Con_5Y1
hsa_circ_0044647	-	ENSG00000008294;	CVB5_5Y2,Con_5Y1
hsa_circ_0045219	-	ENSG00000108588;	CVB5_5Y2,Con_5Y1
hsa_circ_0042009	+	ENSG00000178921;	CVB5_5Y2,Con_5Y1
hsa_circ_0006836	+	ENSG00000038532;	CVB5_5Y2,Con_5Y1
hsa_circ_0038054	+	ENSG00000072864;	CVB5_5Y2,Con_5Y1
hsa_circ_0036586	-	ENSG00000064726;	CVB5_5Y2,Con_5Y1
hsa_circ_0048466	+	ENSG00000141905;	CVB5_5Y2,Con_5Y1
hsa_circ_0002558	+	ENSG00000267216;ENSG0	CVB5_5Y2,Con_5Y1
hsa_circ_0008190	+	ENSG00000101752;	CVB5_5Y2,Con_5Y1
hsa_circ_0062714	-	ENSG00000183765;	CVB5_5Y2,Con_5Y1
hsa_circ_0061031	+	ENSG00000149657;	CVB5_5Y2,Con_5Y1
hsa_circ_0061291	+	ENSG00000155313;	CVB5_5Y2,Con_5Y1
hsa_circ_0081797	-	ENSG00000164815;	CVB5_5Y2,Con_5Y1
hsa_circ_0078762	+	ENSG00000130023;	CVB5_5Y2,Con_5Y1
hsa_circ_0073992	+	ENSG00000113615;	CVB5_5Y2,Con_5Y1
hsa_circ_0071384	+	ENSG00000109466;	CVB5_5Y2,Con_5Y1
hsa_circ_0004358	-	ENSG00000132466;	CVB5_5Y2,Con_5Y1
hsa_circ_0070133	+	ENSG00000138759;	CVB5_5Y2,Con_5Y1
hsa_circ_0007975	+	ENSG00000197548;	CVB5_5Y2,Con_5Y1
hsa_circ_0003533	-	ENSG00000160746;	CVB5_5Y2,Con_5Y1
hsa_circ_0003091	-	ENSG00000114268;	CVB5_5Y2,Con_5Y1
hsa_circ_0003060	-	ENSG00000172340;	CVB5_5Y2,Con_5Y1
hsa_circ_0004276	-	ENSG00000172340;	CVB5_5Y2,Con_5Y1
hsa_circ_0002652	-	ENSG00000115524;	CVB5_5Y2,Con_5Y1
hsa_circ_0003081	+	ENSG00000168958;	CVB5_5Y2,Con_5Y1
hsa_circ_0054282	+	ENSG00000057935;	CVB5_5Y2,Con_5Y1
hsa_circ_0005951	+	ENSG00000162869;	CVB5_5Y2,Con_5Y1
hsa_circ_0015077	-	ENSG00000143183;	CVB5_5Y2,Con_5Y1
hsa_circ_0006869	-	ENSG00000078369;	CVB5_5Y2,Con_5Y1
hsa_circ_0007234	+	ENSG00000082512;	CVB5_5Y2,Con_5Y1
hsa_circ_0010835	+	ENSG00000004487;	CVB5_5Y2,Con_5Y1
hsa_circ_0004839	-	ENSG00000116209;	CVB5_5Y2,Con_5Y1
hsa_circ_0088744	-	ENSG00000148337;	CVB5_5Y2,Con_5Y1
hsa_circ_0086422	+	ENSG00000044459;	CVB5_5Y2,Con_5Y1
hsa_circ_0087247	+	ENSG00000197969;	CVB5_5Y2,Con_5Y1
novel_circ_0018676	-	ENSG00000147255;	CVB5_5Y2,Con_5Y1
novel_circ_0002858	+	n/a	CVB5_5Y2,Con_5Y1
novel_circ_0001807	+	ENSG00000075089;	CVB5_5Y2,Con_5Y1
novel_circ_0001851	-	ENSG00000120837;	CVB5_5Y2,Con_5Y1
novel_circ_0002268	+	ENSG00000111700;ENSG0	CVB5_5Y2,Con_5Y1
novel_circ_0002307	+	ENSG00000004478;	CVB5_5Y2,Con_5Y1
novel_circ_0001310	-	ENSG00000170959;	CVB5_5Y2,Con_5Y1
novel_circ_0001579	+	ENSG00000110075;	CVB5_5Y2,Con_5Y1
novel_circ_0005486	-	n/a	CVB5_5Y2,Con_5Y1
novel_circ_0005213	-	ENSG00000167522;	CVB5_5Y2,Con_5Y1
novel_circ_0004078	+	ENSG00000174197;	CVB5_5Y2,Con_5Y1
novel_circ_0004560	+	ENSG00000173575;	CVB5_5Y2,Con_5Y1
novel_circ_0003383	-	ENSG00000129566;	CVB5_5Y2,Con_5Y1
novel_circ_0006740	-	ENSG00000071564;	CVB5_5Y2,Con_5Y1

novel_circ_0006384	+	ENSG00000101596;	CVB5_5Y2,Con_5Y1
novel_circ_0009431	+	ENSG00000286235;ENSG0	CVB5_5Y2,Con_5Y1
novel_circ_0009543	+	ENSG00000166979;	CVB5_5Y2,Con_5Y1
novel_circ_0015183	+	ENSG00000055211;	CVB5_5Y2,Con_5Y1
novel_circ_0015764	-	ENSG00000112159;	CVB5_5Y2,Con_5Y1
novel_circ_0013299	+	ENSG00000071205;	CVB5_5Y2,Con_5Y1
novel_circ_0013815	+	ENSG00000138768;	CVB5_5Y2,Con_5Y1
novel_circ_0010909	+	ENSG00000077044;	CVB5_5Y2,Con_5Y1
novel_circ_0007377	-	ENSG00000163159;	CVB5_5Y2,Con_5Y1
novel_circ_0007536	+	ENSG00000143179;	CVB5_5Y2,Con_5Y1
novel_circ_0017944	-	ENSG00000136935;	CVB5_5Y2,Con_5Y1
novel_circ_0018471	+	ENSG00000123975;	CVB5_5Y2,Con_5Y1
novel_circ_0017030	-	ENSG00000129295;	CVB5_5Y2,Con_5Y1
novel_circ_0017253	-	ENSG00000147419;	CVB5_5Y2,Con_5Y1
hsa_circ_0030347	-	ENSG00000136100;	CVB5_5Y2,Con_5Y1,
hsa_circ_0002513	-	ENSG00000073921;	CVB5_5Y2,Con_5Y1,
hsa_circ_0008393	-	ENSG00000107554;	CVB5_5Y2,Con_5Y1,
hsa_circ_0007882	+	ENSG00000160551;	CVB5_5Y2,Con_5Y1,
hsa_circ_0042952	-	ENSG00000108651;	CVB5_5Y2,Con_5Y1,
hsa_circ_0007248	-	ENSG00000141698;	CVB5_5Y2,Con_5Y1,
hsa_circ_0044016	-	ENSG00000108840;	CVB5_5Y2,Con_5Y1,
hsa_circ_0044646	-	ENSG00000008294;	CVB5_5Y2,Con_5Y1,
hsa_circ_0005652	+	ENSG00000141198;	CVB5_5Y2,Con_5Y1,
hsa_circ_0005582	-	ENSG00000083093;	CVB5_5Y2,Con_5Y1,
hsa_circ_0035296	+	ENSG00000138594;	CVB5_5Y2,Con_5Y1,
hsa_circ_0008683	+	ENSG00000197102;	CVB5_5Y2,Con_5Y1,
hsa_circ_0002369	-	ENSG00000159082;	CVB5_5Y2,Con_5Y1,
hsa_circ_0001736	+	ENSG00000005483;	CVB5_5Y2,Con_5Y1,
hsa_circ_0083142	+	ENSG00000009335;	CVB5_5Y2,Con_5Y1,
hsa_circ_0007172	-	ENSG00000196821;	CVB5_5Y2,Con_5Y1,
hsa_circ_0076078	+	ENSG00000064999;	CVB5_5Y2,Con_5Y1,
hsa_circ_0004990	-	ENSG00000135317;ENSG0	CVB5_5Y2,Con_5Y1,
hsa_circ_0001534	-	ENSG00000031003;	CVB5_5Y2,Con_5Y1,
hsa_circ_0003057	-	ENSG00000154122;	CVB5_5Y2,Con_5Y1,
hsa_circ_0070911	+	ENSG00000164070;	CVB5_5Y2,Con_5Y1,
hsa_circ_0071123	+	ENSG00000071205;	CVB5_5Y2,Con_5Y1,
hsa_circ_0006673	+	ENSG00000154743;	CVB5_5Y2,Con_5Y1,
hsa_circ_0067531	-	ENSG00000051382;	CVB5_5Y2,Con_5Y1,
hsa_circ_0067997	+	ENSG00000075420;	CVB5_5Y2,Con_5Y1,
hsa_circ_0008550	-	ENSG00000078070;	CVB5_5Y2,Con_5Y1,
hsa_circ_0068629	-	ENSG00000072274;	CVB5_5Y2,Con_5Y1,
hsa_circ_0005873	+	ENSG00000186001;	CVB5_5Y2,Con_5Y1,
hsa_circ_0001988	+	ENSG00000163635;ENSG0	CVB5_5Y2,Con_5Y1,
hsa_circ_0008465	+	ENSG00000168137;	CVB5_5Y2,Con_5Y1,
hsa_circ_0002795	+	ENSG00000021574;	CVB5_5Y2,Con_5Y1,
hsa_circ_0001011	+	ENSG00000162924;	CVB5_5Y2,Con_5Y1,
hsa_circ_0010064	+	ENSG00000116786;	CVB5_5Y2,Con_5Y1,
hsa_circ_0008857	+	ENSG00000118217;	CVB5_5Y2,Con_5Y1,
hsa_circ_0011159	-	ENSG00000120656;	CVB5_5Y2,Con_5Y1,
hsa_circ_0011555	+	ENSG00000146463;	CVB5_5Y2,Con_5Y1,
hsa_circ_0088511	-	ENSG00000148200;	CVB5_5Y2,Con_5Y1,
hsa_circ_0002976	-	ENSG00000137073;	CVB5_5Y2,Con_5Y1,
hsa_circ_0086729	-	ENSG00000137073;	CVB5_5Y2,Con_5Y1,
hsa_circ_0008613	+	ENSG00000147854;	CVB5_5Y2,Con_5Y1,
hsa_circ_0008075	+	ENSG00000197969;	CVB5_5Y2,Con_5Y1,
hsa_circ_0007800	-	ENSG00000120875;	CVB5_5Y2,Con_5Y1,
novel_circ_0018659	-	ENSG00000125676;	CVB5_5Y2,Con_5Y1,
novel_circ_0001509	-	ENSG00000133315;	CVB5_5Y2,Con_5Y1,

novel_circ_0000129	+	n/a	CVB5_5Y2,Con_5Y1,
novel_circ_0005998	+	ENSG00000171634;	CVB5_5Y2,Con_5Y1,
novel_circ_0004489	+	ENSG00000259429;	CVB5_5Y2,Con_5Y1,
novel_circ_0003701	+	ENSG00000033170;	CVB5_5Y2,Con_5Y1,
novel_circ_0006705	-	ENSG00000105612;	CVB5_5Y2,Con_5Y1,
novel_circ_0009793	+	ENSG00000159873;	CVB5_5Y2,Con_5Y1,
novel_circ_0016138	+	ENSG00000184863;	CVB5_5Y2,Con_5Y1,
novel_circ_0015047	+	ENSG00000146373;	CVB5_5Y2,Con_5Y1,
novel_circ_0014446	+	ENSG00000127022;	CVB5_5Y2,Con_5Y1,
novel_circ_0014661	+	ENSG00000153015;	CVB5_5Y2,Con_5Y1,
novel_circ_0013760	+	ENSG00000174780;	CVB5_5Y2,Con_5Y1,
novel_circ_0012755	-	ENSG00000173473;	CVB5_5Y2,Con_5Y1,
novel_circ_0011320	+	ENSG00000171132;	CVB5_5Y2,Con_5Y1,
novel_circ_0011654	-	ENSG00000068654;	CVB5_5Y2,Con_5Y1,
novel_circ_0007737	+	ENSG00000157181;	CVB5_5Y2,Con_5Y1,
novel_circ_0008066	-	ENSG00000143776;	CVB5_5Y2,Con_5Y1,
novel_circ_0018196	-	ENSG00000120159;	CVB5_5Y2,Con_5Y1,
hsa_circ_0091103	-	ENSG00000085224;	CVB5_5Y2,Con_5Y1,
hsa_circ_0031007	-	ENSG00000139835;	CVB5_5Y2,Con_5Y1,
hsa_circ_0002473	+	ENSG00000102580;	CVB5_5Y2,Con_5Y1,
hsa_circ_0005424	+	ENSG00000198431;	CVB5_5Y2,Con_5Y1,
hsa_circ_0000438	-	ENSG00000204842;	CVB5_5Y2,Con_5Y1,
hsa_circ_0018046	-	ENSG00000169126;	CVB5_5Y2,Con_5Y1,
hsa_circ_0043297	-	ENSG00000275066;	CVB5_5Y2,Con_5Y1,
hsa_circ_0005877	-	ENSG00000131747;	CVB5_5Y2,Con_5Y1,
hsa_circ_0038074	+	ENSG00000103222;	CVB5_5Y2,Con_5Y1,
hsa_circ_0040458	-	ENSG00000090863;	CVB5_5Y2,Con_5Y1,
hsa_circ_0005220	+	ENSG00000172530;	CVB5_5Y2,Con_5Y1,
hsa_circ_0003973	+	ENSG00000166949;	CVB5_5Y2,Con_5Y1,
hsa_circ_0000542	+	ENSG00000032219;	CVB5_5Y2,Con_5Y1,
hsa_circ_0032821	-	ENSG00000100629;	CVB5_5Y2,Con_5Y1,
hsa_circ_0004786	+	ENSG00000125753;	CVB5_5Y2,Con_5Y1,
hsa_circ_0006332	+	ENSG00000101057;	CVB5_5Y2,Con_5Y1,
hsa_circ_0081822	+	ENSG00000005483;	CVB5_5Y2,Con_5Y1,
hsa_circ_0002494	-	ENSG00000157800;	CVB5_5Y2,Con_5Y1,
hsa_circ_0083226	+	ENSG00000126870;	CVB5_5Y2,Con_5Y1,
hsa_circ_0076560	-	ENSG00000124571;	CVB5_5Y2,Con_5Y1,
hsa_circ_0006499	-	ENSG00000145723;	CVB5_5Y2,Con_5Y1,
hsa_circ_0007773	-	ENSG00000055147;	CVB5_5Y2,Con_5Y1,
hsa_circ_0006894	-	ENSG00000120137;	CVB5_5Y2,Con_5Y1,
hsa_circ_0008164	-	ENSG00000132842;	CVB5_5Y2,Con_5Y1,
hsa_circ_0068956	-	ENSG00000087269;	CVB5_5Y2,Con_5Y1,
hsa_circ_0007820	+	ENSG00000065882;	CVB5_5Y2,Con_5Y1,
hsa_circ_0007143	+	ENSG00000144824;	CVB5_5Y2,Con_5Y1,
hsa_circ_0053378	+	ENSG00000172954;	CVB5_5Y2,Con_5Y1,
hsa_circ_0014088	+	ENSG00000143363;	CVB5_5Y2,Con_5Y1,
hsa_circ_0000018	+	ENSG00000116138;	CVB5_5Y2,Con_5Y1,
hsa_circ_0004777	+	ENSG00000143842;	CVB5_5Y2,Con_5Y1,
hsa_circ_0001958	-	ENSG00000135749;	CVB5_5Y2,Con_5Y1,
hsa_circ_0004109	-	ENSG00000106771;	CVB5_5Y2,Con_5Y1,
hsa_circ_0088072	-	ENSG00000119314;	CVB5_5Y2,Con_5Y1,
novel_circ_0001025	+	ENSG00000133805;	CVB5_5Y2,Con_5Y1,
novel_circ_0000408	-	ENSG00000078114;	CVB5_5Y2,Con_5Y1,
novel_circ_0004472	+	ENSG00000041357;	CVB5_5Y2,Con_5Y1,
novel_circ_0015867	-	ENSG00000164815;	CVB5_5Y2,Con_5Y1,
novel_circ_0015924	+	ENSG00000106443;	CVB5_5Y2,Con_5Y1,
novel_circ_0014126	+	ENSG00000170606;	CVB5_5Y2,Con_5Y1,
novel_circ_0014497	-	ENSG00000056097;	CVB5_5Y2,Con_5Y1,

novel_circ_0014751	-	ENSG00000164347;	CVB5_5Y2,Con_5Y1,
novel_circ_0010265	+	ENSG00000080345;	CVB5_5Y2,Con_5Y1,
novel_circ_0010368	-	ENSG00000198648;	CVB5_5Y2,Con_5Y1,
novel_circ_0007806	-	ENSG00000118193;	CVB5_5Y2,Con_5Y1,
novel_circ_0008088	+	ENSG00000168118;	CVB5_5Y2,Con_5Y1,
novel_circ_0016866	-	ENSG00000253649;	CVB5_5Y2,Con_5Y1,
novel_circ_0017485	+	ENSG00000147316;	CVB5_5Y2,Con_5Y1,
hsa_circ_0000469	+	ENSG00000152520;	CVB5_5Y2,Con_5Y1,
hsa_circ_0029311	-	ENSG00000196498;	CVB5_5Y2,Con_5Y1,
hsa_circ_0000409	-	ENSG00000198056;	CVB5_5Y2,Con_5Y1,
hsa_circ_0002539	-	ENSG00000179912;ENSG0	CVB5_5Y2,Con_5Y1,
hsa_circ_0009168	-	ENSG00000108588;	CVB5_5Y2,Con_5Y1,
hsa_circ_0000721	+	ENSG00000197943;	CVB5_5Y2,Con_5Y1,
hsa_circ_0035289	+	ENSG00000138594;	CVB5_5Y2,Con_5Y1,
hsa_circ_0031417	+	ENSG00000092108;	CVB5_5Y2,Con_5Y1,
hsa_circ_0032511	+	ENSG00000100767;	CVB5_5Y2,Con_5Y1,
hsa_circ_0005030	-	ENSG00000119682;	CVB5_5Y2,Con_5Y1,
hsa_circ_0049072	+	ENSG00000099783;	CVB5_5Y2,Con_5Y1,
hsa_circ_0060219	-	ENSG00000149639;	CVB5_5Y2,Con_5Y1,
hsa_circ_0061726	-	ENSG00000185658;	CVB5_5Y2,Con_5Y1,
hsa_circ_0004381	+	ENSG00000049618;	CVB5_5Y2,Con_5Y1,
hsa_circ_0005608	+	ENSG00000043143;	CVB5_5Y2,Con_5Y1,
hsa_circ_0074158	+	ENSG00000044115;	CVB5_5Y2,Con_5Y1,
hsa_circ_0073434	+	ENSG00000164292;	CVB5_5Y2,Con_5Y1,
hsa_circ_0008757	+	ENSG00000138768;	CVB5_5Y2,Con_5Y1,
hsa_circ_0006866	-	ENSG00000145332;	CVB5_5Y2,Con_5Y1,
hsa_circ_0008649	-	ENSG00000167103;	CVB5_5Y2,Con_5Y1,
hsa_circ_0085278	-	ENSG00000155096;	CVB5_5Y2,Con_5Y1,
novel_circ_0002656	-	ENSG00000133858;	CVB5_5Y2,Con_5Y1,
novel_circ_0001215	+	ENSG00000084234;	CVB5_5Y2,Con_5Y1,
novel_circ_0000041	-	ENSG00000198408;	CVB5_5Y2,Con_5Y1,
novel_circ_0005112	-	ENSG00000153774;	CVB5_5Y2,Con_5Y1,
novel_circ_0014005	-	ENSG00000129595;	CVB5_5Y2,Con_5Y1,
novel_circ_0014364	+	ENSG00000113327;	CVB5_5Y2,Con_5Y1,
novel_circ_0012569	-	ENSG00000113851;	CVB5_5Y2,Con_5Y1,
novel_circ_0012716	-	ENSG00000163814;	CVB5_5Y2,Con_5Y1,
novel_circ_0016980	+	ENSG00000156831;	CVB5_5Y2,Con_5Y1,
hsa_circ_0005816	+	ENSG00000239407;	CVB5_5Y2,Con_5Y2
hsa_circ_0008775	+	ENSG00000028203;	CVB5_5Y2,Con_5Y2
hsa_circ_0003712	-	ENSG00000148950;	CVB5_5Y2,Con_5Y2
hsa_circ_0005793	+	ENSG00000173715;	CVB5_5Y2,Con_5Y2
hsa_circ_0024032	-	ENSG00000020922;	CVB5_5Y2,Con_5Y2
hsa_circ_0003499	+	ENSG00000150760;	CVB5_5Y2,Con_5Y2
hsa_circ_0002118	+	ENSG00000108021;	CVB5_5Y2,Con_5Y2
hsa_circ_0043157	-	ENSG00000278259;	CVB5_5Y2,Con_5Y2
hsa_circ_0006692	-	n/a	CVB5_5Y2,Con_5Y2
hsa_circ_0008805	+	ENSG00000275832;	CVB5_5Y2,Con_5Y2
hsa_circ_0043528	+	ENSG00000131759;	CVB5_5Y2,Con_5Y2
hsa_circ_0004090	-	ENSG00000108395;	CVB5_5Y2,Con_5Y2
hsa_circ_0008480	+	ENSG00000138834;	CVB5_5Y2,Con_5Y2
hsa_circ_0038376	+	ENSG00000174628;	CVB5_5Y2,Con_5Y2
hsa_circ_0040908	+	ENSG00000176715;	CVB5_5Y2,Con_5Y2
hsa_circ_0035828	-	ENSG00000259316;ENSG0	CVB5_5Y2,Con_5Y2
hsa_circ_0037010	+	ENSG00000140563;	CVB5_5Y2,Con_5Y2
hsa_circ_0049207	-	ENSG00000130816;	CVB5_5Y2,Con_5Y2
hsa_circ_0046968	+	ENSG00000141391;	CVB5_5Y2,Con_5Y2
hsa_circ_0047678	-	ENSG00000167306;	CVB5_5Y2,Con_5Y2
hsa_circ_0004128	-	ENSG00000183765;	CVB5_5Y2,Con_5Y2

hsa_circ_0062713	-	ENSG00000183765;	CVB5_5Y2,Con_5Y2
hsa_circ_0003723	-	ENSG00000100401;	CVB5_5Y2,Con_5Y2
hsa_circ_0004359	-	ENSG00000124177;	CVB5_5Y2,Con_5Y2
hsa_circ_0008452	-	ENSG00000185658;	CVB5_5Y2,Con_5Y2
hsa_circ_0001197	-	ENSG00000160193;	CVB5_5Y2,Con_5Y2
hsa_circ_0006887	-	ENSG00000146376;	CVB5_5Y2,Con_5Y2
hsa_circ_0006305	+	ENSG00000049618;	CVB5_5Y2,Con_5Y2
hsa_circ_0003042	+	ENSG00000137275;	CVB5_5Y2,Con_5Y2
hsa_circ_0008801	+	ENSG00000137161;	CVB5_5Y2,Con_5Y2
hsa_circ_0004894	-	ENSG00000031003;	CVB5_5Y2,Con_5Y2
hsa_circ_0074082	-	ENSG00000031003;	CVB5_5Y2,Con_5Y2
hsa_circ_0073052	+	ENSG00000122008;	CVB5_5Y2,Con_5Y2
hsa_circ_0070939	+	ENSG00000138709;	CVB5_5Y2,Con_5Y2
hsa_circ_0006213	+	ENSG00000004534;	CVB5_5Y2,Con_5Y2
hsa_circ_0009020	-	ENSG00000186522;	CVB5_5Y2,Con_5Y2
hsa_circ_0056262	+	ENSG00000088179;	CVB5_5Y2,Con_5Y2
hsa_circ_0004442	-	ENSG00000198586;	CVB5_5Y2,Con_5Y2
hsa_circ_0007529	+	ENSG00000115827;	CVB5_5Y2,Con_5Y2
hsa_circ_0058710	+	ENSG00000135930;	CVB5_5Y2,Con_5Y2
hsa_circ_0053063	-	ENSG00000084754;	CVB5_5Y2,Con_5Y2
hsa_circ_0054474	+	ENSG00000116062;	CVB5_5Y2,Con_5Y2
hsa_circ_0054802	-	ENSG00000115464;	CVB5_5Y2,Con_5Y2
hsa_circ_0055161	-	ENSG00000003137;	CVB5_5Y2,Con_5Y2
hsa_circ_0009248	-	ENSG00000160087;	CVB5_5Y2,Con_5Y2
hsa_circ_0004861	+	ENSG00000116731;	CVB5_5Y2,Con_5Y2
hsa_circ_0008653	+	ENSG00000116199;	CVB5_5Y2,Con_5Y2
hsa_circ_0000190	+	ENSG00000143771;	CVB5_5Y2,Con_5Y2
hsa_circ_0000192	+	ENSG00000143641;	CVB5_5Y2,Con_5Y2
hsa_circ_0000053	-	ENSG00000196182;	CVB5_5Y2,Con_5Y2
hsa_circ_0089720	+	ENSG00000181090;	CVB5_5Y2,Con_5Y2
hsa_circ_0008367	-	ENSG00000196305;	CVB5_5Y2,Con_5Y2
hsa_circ_0085714	-	ENSG00000123908;	CVB5_5Y2,Con_5Y2
hsa_circ_0084119	+	ENSG00000078668;	CVB5_5Y2,Con_5Y2
novel_circ_0018696	-	ENSG00000213468;	CVB5_5Y2,Con_5Y2
novel_circ_0001846	-	ENSG00000111696;	CVB5_5Y2,Con_5Y2
novel_circ_0002179	-	ENSG00000196498;	CVB5_5Y2,Con_5Y2
novel_circ_0002186	-	ENSG00000196498;	CVB5_5Y2,Con_5Y2
novel_circ_0002426	-	ENSG00000111371;	CVB5_5Y2,Con_5Y2
novel_circ_0000020	+	ENSG00000107815;	CVB5_5Y2,Con_5Y2
novel_circ_0000251	-	ENSG00000107669;	CVB5_5Y2,Con_5Y2
novel_circ_0000673	+	ENSG00000165476;	CVB5_5Y2,Con_5Y2
novel_circ_0000774	-	ENSG00000122884;	CVB5_5Y2,Con_5Y2
novel_circ_0000945	+	ENSG00000119969;	CVB5_5Y2,Con_5Y2
novel_circ_0000989	-	ENSG00000107554;	CVB5_5Y2,Con_5Y2
novel_circ_0005550	-	ENSG00000008838;	CVB5_5Y2,Con_5Y2
novel_circ_0005698	+	ENSG00000198933;	CVB5_5Y2,Con_5Y2
novel_circ_0006098	-	ENSG00000055483;	CVB5_5Y2,Con_5Y2
novel_circ_0006169	-	ENSG00000141551;	CVB5_5Y2,Con_5Y2
novel_circ_0004652	-	ENSG00000133393;	CVB5_5Y2,Con_5Y2
novel_circ_0005047	+	ENSG00000189091;	CVB5_5Y2,Con_5Y2
novel_circ_0005109	+	ENSG00000186187;	CVB5_5Y2,Con_5Y2
novel_circ_0004083	+	ENSG00000159433;	CVB5_5Y2,Con_5Y2
novel_circ_0004351	+	ENSG00000033800;	CVB5_5Y2,Con_5Y2
novel_circ_0003773	+	ENSG00000100767;	CVB5_5Y2,Con_5Y2
novel_circ_0007111	+	ENSG00000160633;	CVB5_5Y2,Con_5Y2
novel_circ_0006411	+	ENSG00000134759;	CVB5_5Y2,Con_5Y2
novel_circ_0009737	+	ENSG00000099991;	CVB5_5Y2,Con_5Y2
novel_circ_0009284	+	ENSG00000125779;	CVB5_5Y2,Con_5Y2

novel_circ_0015907	-	ENSG00000146776;	CVB5_5Y2,Con_5Y2
novel_circ_0015961	-	ENSG00000048405;	CVB5_5Y2,Con_5Y2
novel_circ_0016481	+	ENSG00000106305;	CVB5_5Y2,Con_5Y2
novel_circ_0015009	-	ENSG00000153975;	CVB5_5Y2,Con_5Y2
novel_circ_0015389	-	ENSG00000172197;	CVB5_5Y2,Con_5Y2
novel_circ_0014151	+	ENSG00000145833;	CVB5_5Y2,Con_5Y2
novel_circ_0013417	-	ENSG00000137601;	CVB5_5Y2,Con_5Y2
novel_circ_0013517	+	ENSG00000248515;	CVB5_5Y2,Con_5Y2
novel_circ_0013697	-	ENSG00000170448;	CVB5_5Y2,Con_5Y2
novel_circ_0012810	+	ENSG00000177479;	CVB5_5Y2,Con_5Y2
novel_circ_0012910	+	n/a	CVB5_5Y2,Con_5Y2
novel_circ_0012998	-	n/a	CVB5_5Y2,Con_5Y2
novel_circ_0010582	-	ENSG00000197121;	CVB5_5Y2,Con_5Y2
novel_circ_0011058	+	ENSG00000163811;	CVB5_5Y2,Con_5Y2
novel_circ_0007229	+	n/a	CVB5_5Y2,Con_5Y2
novel_circ_0007800	+	ENSG00000169914;	CVB5_5Y2,Con_5Y2
novel_circ_0008250	-	ENSG00000153187;	CVB5_5Y2,Con_5Y2
novel_circ_0008347	-	ENSG00000116350;	CVB5_5Y2,Con_5Y2
novel_circ_0008535	+	ENSG00000066135;ENSG0	CVB5_5Y2,Con_5Y2
novel_circ_0017992	+	ENSG00000167123;	CVB5_5Y2,Con_5Y2
novel_circ_0017251	+	ENSG00000120899;	CVB5_5Y2,Con_5Y2
hsa_circ_0004408	+	ENSG00000185896;	CVB5_5Y2,Con_5Y2,
hsa_circ_0030707	-	ENSG00000102572;	CVB5_5Y2,Con_5Y2,
hsa_circ_0034657	-	ENSG00000128908;	CVB5_5Y2,Con_5Y2,
hsa_circ_0034989	-	ENSG00000067369;	CVB5_5Y2,Con_5Y2,
hsa_circ_0000645	-	ENSG00000117899;	CVB5_5Y2,Con_5Y2,
hsa_circ_0000659	+	ENSG00000140563;	CVB5_5Y2,Con_5Y2,
hsa_circ_0006831	-	ENSG00000198554;	CVB5_5Y2,Con_5Y2,
hsa_circ_0007163	+	ENSG00000033170;	CVB5_5Y2,Con_5Y2,
hsa_circ_0032526	+	ENSG00000258653;ENSG0	CVB5_5Y2,Con_5Y2,
hsa_circ_0005973	+	ENSG00000127616;	CVB5_5Y2,Con_5Y2,
hsa_circ_0062545	-	ENSG00000228315;	CVB5_5Y2,Con_5Y2,
hsa_circ_0061938	+	ENSG00000160218;	CVB5_5Y2,Con_5Y2,
hsa_circ_0007017	-	ENSG00000055609;	CVB5_5Y2,Con_5Y2,
hsa_circ_0001714	-	ENSG00000009954;	CVB5_5Y2,Con_5Y2,
hsa_circ_0007842	-	ENSG00000049541;	CVB5_5Y2,Con_5Y2,
hsa_circ_0077496	-	ENSG00000112249;	CVB5_5Y2,Con_5Y2,
hsa_circ_0001542	+	ENSG00000131503;ENSG0	CVB5_5Y2,Con_5Y2,
hsa_circ_0073486	-	ENSG00000153922;	CVB5_5Y2,Con_5Y2,
hsa_circ_0006977	-	ENSG00000138757;	CVB5_5Y2,Con_5Y2,
hsa_circ_0068129	+	ENSG00000114416;	CVB5_5Y2,Con_5Y2,
hsa_circ_0003927	-	ENSG00000047849;	CVB5_5Y2,Con_5Y2,
hsa_circ_0066147	-	ENSG00000163935;	CVB5_5Y2,Con_5Y2,
hsa_circ_0058809	+	ENSG00000157985;	CVB5_5Y2,Con_5Y2,
hsa_circ_0008161	-	ENSG00000116539;	CVB5_5Y2,Con_5Y2,
hsa_circ_0005814	+	ENSG00000074964;	CVB5_5Y2,Con_5Y2,
hsa_circ_0002008	+	ENSG00000065243;	CVB5_5Y2,Con_5Y2,
hsa_circ_0007592	+	ENSG00000120158;	CVB5_5Y2,Con_5Y2,
hsa_circ_0006928	-	ENSG00000165138;	CVB5_5Y2,Con_5Y2,
novel_circ_0001856	-	ENSG00000255641;	CVB5_5Y2,Con_5Y2,
novel_circ_0002628	+	ENSG00000135679;	CVB5_5Y2,Con_5Y2,
novel_circ_0006519	-	ENSG00000134440;	CVB5_5Y2,Con_5Y2,
novel_circ_0009944	+	ENSG00000241484;ENSG0	CVB5_5Y2,Con_5Y2,
novel_circ_0015107	-	ENSG00000118514;	CVB5_5Y2,Con_5Y2,
novel_circ_0015384	-	ENSG00000124795;	CVB5_5Y2,Con_5Y2,
novel_circ_0011369	-	ENSG00000068878;	CVB5_5Y2,Con_5Y2,
novel_circ_0011372	+	ENSG00000214595;	CVB5_5Y2,Con_5Y2,
novel_circ_0008182	+	ENSG00000284770;ENSG0	CVB5_5Y2,Con_5Y2,

novel_circ_0018586	+	ENSG00000136937;	CVB5_5Y2,Con_5Y2,
hsa_circ_0029762	-	ENSG00000102699;	CVB5_5Y2,Con_5Y3
hsa_circ_0029441	+	ENSG00000061936;	CVB5_5Y2,Con_5Y3
hsa_circ_0025128	-	ENSG00000067182;	CVB5_5Y2,Con_5Y3
hsa_circ_0000360	-	ENSG00000110367;	CVB5_5Y2,Con_5Y3
hsa_circ_0002955	+	ENSG00000196914;	CVB5_5Y2,Con_5Y3
hsa_circ_0022631	+	ENSG00000168439;	CVB5_5Y2,Con_5Y3
hsa_circ_0002417	+	ENSG00000110075;	CVB5_5Y2,Con_5Y3
hsa_circ_0023558	-	ENSG00000168014;	CVB5_5Y2,Con_5Y3
hsa_circ_0020251	-	ENSG00000107669;	CVB5_5Y2,Con_5Y3
hsa_circ_0044158	-	ENSG00000161714;	CVB5_5Y2,Con_5Y3
hsa_circ_0002809	-	ENSG00000108395;	CVB5_5Y2,Con_5Y3
hsa_circ_0003864	+	ENSG00000013364;	CVB5_5Y2,Con_5Y3
hsa_circ_0040738	+	ENSG00000131149;	CVB5_5Y2,Con_5Y3
hsa_circ_0032190	+	ENSG00000100714;	CVB5_5Y2,Con_5Y3
hsa_circ_0050338	+	ENSG00000105176;	CVB5_5Y2,Con_5Y3
hsa_circ_0063452	-	ENSG00000196588;	CVB5_5Y2,Con_5Y3
hsa_circ_0061208	+	ENSG00000101161;	CVB5_5Y2,Con_5Y3
hsa_circ_0006087	+	ENSG00000091009;ENSG0	CVB5_5Y2,Con_5Y3
hsa_circ_0073102	-	ENSG00000132842;	CVB5_5Y2,Con_5Y3
hsa_circ_0068960	-	ENSG00000087269;	CVB5_5Y2,Con_5Y3
hsa_circ_0069740	+	ENSG00000145216;	CVB5_5Y2,Con_5Y3
hsa_circ_0069086	+	ENSG00000013288;	CVB5_5Y2,Con_5Y3
hsa_circ_0005830	-	ENSG00000178950;	CVB5_5Y2,Con_5Y3
hsa_circ_0067222	-	ENSG00000163902;	CVB5_5Y2,Con_5Y3
hsa_circ_0058779	-	ENSG00000085982;	CVB5_5Y2,Con_5Y3
hsa_circ_0055190	-	ENSG00000144036;	CVB5_5Y2,Con_5Y3
hsa_circ_0004095	+	ENSG00000197312;	CVB5_5Y2,Con_5Y3
hsa_circ_0006578	-	ENSG00000196182;	CVB5_5Y2,Con_5Y3
hsa_circ_0088680	-	ENSG00000136830;	CVB5_5Y2,Con_5Y3
hsa_circ_0006088	+	ENSG00000197694;	CVB5_5Y2,Con_5Y3
hsa_circ_0084929	+	ENSG00000104413;	CVB5_5Y2,Con_5Y3
novel_circ_0018747	+	ENSG00000071859;	CVB5_5Y2,Con_5Y3
novel_circ_0000178	-	ENSG00000151893;	CVB5_5Y2,Con_5Y3
novel_circ_0000465	-	ENSG00000169126;	CVB5_5Y2,Con_5Y3
novel_circ_0005606	-	ENSG00000132388;	CVB5_5Y2,Con_5Y3
novel_circ_0005770	-	ENSG00000005100;	CVB5_5Y2,Con_5Y3
novel_circ_0005115	-	ENSG00000153774;	CVB5_5Y2,Con_5Y3
novel_circ_0005144	-	ENSG00000153786;	CVB5_5Y2,Con_5Y3
novel_circ_0004006	-	ENSG00000166912;	CVB5_5Y2,Con_5Y3
novel_circ_0006942	+	ENSG00000077312;	CVB5_5Y2,Con_5Y3
novel_circ_0007133	-	ENSG00000070423;	CVB5_5Y2,Con_5Y3
novel_circ_0006377	+	ENSG00000101596;	CVB5_5Y2,Con_5Y3
novel_circ_0014935	-	ENSG00000112249;	CVB5_5Y2,Con_5Y3
novel_circ_0015777	+	ENSG00000118412;	CVB5_5Y2,Con_5Y3
novel_circ_0014862	-	ENSG00000164180;	CVB5_5Y2,Con_5Y3
novel_circ_0013257	-	ENSG00000145391;	CVB5_5Y2,Con_5Y3
novel_circ_0013704	+	ENSG00000109171;	CVB5_5Y2,Con_5Y3
novel_circ_0012803	-	ENSG00000114268;	CVB5_5Y2,Con_5Y3
novel_circ_0010389	-	ENSG00000152253;	CVB5_5Y2,Con_5Y3
novel_circ_0010590	+	ENSG00000270757;ENSG0	CVB5_5Y2,Con_5Y3
novel_circ_0010772	+	n/a	CVB5_5Y2,Con_5Y3
novel_circ_0007823	+	ENSG00000198700;	CVB5_5Y2,Con_5Y3
novel_circ_0008379	-	ENSG00000184007;	CVB5_5Y2,Con_5Y3
novel_circ_0008831	+	n/a	CVB5_5Y2,Con_5Y3
novel_circ_0016898	-	ENSG00000147677;	CVB5_5Y2,Con_5Y3
novel_circ_0016927	-	ENSG00000136986;	CVB5_5Y2,Con_5Y3
hsa_circ_0029934	-	ENSG00000244754;	CVB5_5Y2,CVB5_5Y

hsa_circ_0003170	-	ENSG00000102572;	CVB5_5Y2,CVB5_5Y
hsa_circ_0028339	-	ENSG00000111300;	CVB5_5Y2,CVB5_5Y
hsa_circ_0005186	+	ENSG00000061936;	CVB5_5Y2,CVB5_5Y
hsa_circ_0005450	+	ENSG00000067798;	CVB5_5Y2,CVB5_5Y
hsa_circ_0024230	+	ENSG00000149311;	CVB5_5Y2,CVB5_5Y
hsa_circ_0020340	+	ENSG00000019995;	CVB5_5Y2,CVB5_5Y
hsa_circ_0018937	-	ENSG00000151208;	CVB5_5Y2,CVB5_5Y
hsa_circ_0042799	-	ENSG00000141298;	CVB5_5Y2,CVB5_5Y
hsa_circ_0006860	-	ENSG00000278259;	CVB5_5Y2,CVB5_5Y
hsa_circ_0045881	+	ENSG00000183077;	CVB5_5Y2,CVB5_5Y
hsa_circ_0005429	+	ENSG00000072415;	CVB5_5Y2,CVB5_5Y
hsa_circ_0032377	-	ENSG00000006432;	CVB5_5Y2,CVB5_5Y
hsa_circ_0032379	-	ENSG00000006432;	CVB5_5Y2,CVB5_5Y
hsa_circ_0005007	+	ENSG00000090060;	CVB5_5Y2,CVB5_5Y
hsa_circ_0005679	-	ENSG00000242247;	CVB5_5Y2,CVB5_5Y
hsa_circ_0060236	-	ENSG00000080839;	CVB5_5Y2,CVB5_5Y
hsa_circ_0061746	-	ENSG00000185658;	CVB5_5Y2,CVB5_5Y
hsa_circ_0081849	-	ENSG00000091127;	CVB5_5Y2,CVB5_5Y
hsa_circ_0008612	+	ENSG00000106144;	CVB5_5Y2,CVB5_5Y
hsa_circ_0003249	-	ENSG00000120256;ENSG0	CVB5_5Y2,CVB5_5Y
hsa_circ_0002165	-	ENSG00000096063;	CVB5_5Y2,CVB5_5Y
hsa_circ_0073527	+	ENSG00000151422;	CVB5_5Y2,CVB5_5Y
hsa_circ_0006472	-	ENSG00000164099;	CVB5_5Y2,CVB5_5Y
hsa_circ_0008910	-	ENSG00000129187;	CVB5_5Y2,CVB5_5Y
hsa_circ_0008394	+	ENSG00000113845;	CVB5_5Y2,CVB5_5Y
hsa_circ_0001361	+	ENSG00000075420;	CVB5_5Y2,CVB5_5Y
hsa_circ_0065269	-	ENSG00000173473;	CVB5_5Y2,CVB5_5Y
hsa_circ_0056473	+	ENSG00000136002;	CVB5_5Y2,CVB5_5Y
hsa_circ_0004642	-	ENSG00000055917;	CVB5_5Y2,CVB5_5Y
hsa_circ_0004554	-	ENSG00000153827;	CVB5_5Y2,CVB5_5Y
hsa_circ_0000996	+	ENSG00000143919;	CVB5_5Y2,CVB5_5Y
hsa_circ_0003435	+	ENSG00000126070;	CVB5_5Y2,CVB5_5Y
hsa_circ_0005157	+	ENSG00000171793;	CVB5_5Y2,CVB5_5Y
hsa_circ_0088281	-	ENSG00000136861;	CVB5_5Y2,CVB5_5Y
hsa_circ_0088389	-	ENSG00000056586;	CVB5_5Y2,CVB5_5Y
novel_circ_0002133	-	ENSG00000280138;	CVB5_5Y2,CVB5_5Y
novel_circ_0001692	+	ENSG00000165490;	CVB5_5Y2,CVB5_5Y
novel_circ_0000255	+	ENSG00000151465;	CVB5_5Y2,CVB5_5Y
novel_circ_0000492	-	ENSG00000107951;	CVB5_5Y2,CVB5_5Y
novel_circ_0005564	-	ENSG00000131746;	CVB5_5Y2,CVB5_5Y
novel_circ_0005686	+	ENSG00000141279;	CVB5_5Y2,CVB5_5Y
novel_circ_0005886	-	ENSG00000136492;	CVB5_5Y2,CVB5_5Y
novel_circ_0004789	-	ENSG00000169180;	CVB5_5Y2,CVB5_5Y
novel_circ_0005185	+	ENSG00000158545;	CVB5_5Y2,CVB5_5Y
novel_circ_0006288	+	ENSG00000101654;	CVB5_5Y2,CVB5_5Y
novel_circ_0006301	-	ENSG00000141446;	CVB5_5Y2,CVB5_5Y
novel_circ_0015105	+	ENSG00000028839;	CVB5_5Y2,CVB5_5Y
novel_circ_0014179	-	ENSG00000031003;	CVB5_5Y2,CVB5_5Y
novel_circ_0012681	-	ENSG00000168038;	CVB5_5Y2,CVB5_5Y
novel_circ_0010400	+	ENSG00000138398;	CVB5_5Y2,CVB5_5Y
novel_circ_0010799	+	ENSG00000236451;	CVB5_5Y2,CVB5_5Y
novel_circ_0007929	+	ENSG00000136643;	CVB5_5Y2,CVB5_5Y
novel_circ_0016970	+	ENSG00000180938;	CVB5_5Y2,CVB5_5Y
novel_circ_0017188	+	ENSG00000078674;	CVB5_5Y2,CVB5_5Y
novel_circ_0017231	+	ENSG00000246582;	CVB5_5Y2,CVB5_5Y
novel_circ_0017462	+	ENSG00000171316;	CVB5_5Y2,CVB5_5Y
hsa_circ_0005574	-	ENSG00000147010;	CVB5_5Y2,CVB5_5Y
hsa_circ_0025909	+	ENSG00000134283;	CVB5_5Y2,CVB5_5Y

hsa_circ_0019658	-	ENSG00000120029;	CVB5_5Y2,CVB5_5Y
hsa_circ_0006408	-	ENSG00000170759;	CVB5_5Y2,CVB5_5Y
hsa_circ_0003020	-	ENSG00000265354;	CVB5_5Y2,CVB5_5Y
hsa_circ_0007743	+	ENSG00000006695;	CVB5_5Y2,CVB5_5Y
hsa_circ_0008603	+	ENSG00000072210;	CVB5_5Y2,CVB5_5Y
hsa_circ_0003796	+	ENSG00000171634;	CVB5_5Y2,CVB5_5Y
hsa_circ_0040609	+	ENSG00000197943;	CVB5_5Y2,CVB5_5Y
hsa_circ_0032305	+	ENSG00000182185;	CVB5_5Y2,CVB5_5Y
hsa_circ_0049192	-	ENSG00000130816;	CVB5_5Y2,CVB5_5Y
hsa_circ_0061178	-	ENSG00000130584;	CVB5_5Y2,CVB5_5Y
hsa_circ_0082171	-	ENSG00000106344;	CVB5_5Y2,CVB5_5Y
hsa_circ_0075675	+	ENSG00000008083;	CVB5_5Y2,CVB5_5Y
hsa_circ_0004271	+	ENSG00000181789;	CVB5_5Y2,CVB5_5Y
hsa_circ_0068472	-	ENSG00000163918;	CVB5_5Y2,CVB5_5Y
hsa_circ_0064679	+	ENSG00000153551;	CVB5_5Y2,CVB5_5Y
hsa_circ_0064889	+	ENSG00000157036;	CVB5_5Y2,CVB5_5Y
hsa_circ_0008639	-	ENSG00000173473;	CVB5_5Y2,CVB5_5Y
hsa_circ_0006838	+	ENSG00000177479;	CVB5_5Y2,CVB5_5Y
hsa_circ_0004355	+	ENSG00000241553;ENSG0	CVB5_5Y2,CVB5_5Y
hsa_circ_0004919	+	ENSG00000138380;	CVB5_5Y2,CVB5_5Y
hsa_circ_0008339	-	ENSG00000132680;	CVB5_5Y2,CVB5_5Y
hsa_circ_0015136	-	ENSG00000143147;	CVB5_5Y2,CVB5_5Y
hsa_circ_0002924	-	ENSG00000118873;	CVB5_5Y2,CVB5_5Y
hsa_circ_0089728	+	ENSG00000181090;	CVB5_5Y2,CVB5_5Y
novel_circ_0005264	-	n/a	CVB5_5Y2,CVB5_5Y
novel_circ_0005717	+	ENSG00000108848;	CVB5_5Y2,CVB5_5Y
novel_circ_0005013	-	ENSG00000132604;	CVB5_5Y2,CVB5_5Y
novel_circ_0006753	+	ENSG00000099331;	CVB5_5Y2,CVB5_5Y
novel_circ_0006605	+	ENSG00000060069;	CVB5_5Y2,CVB5_5Y
novel_circ_0013189	-	ENSG00000159692;	CVB5_5Y2,CVB5_5Y
novel_circ_0012416	+	ENSG00000188001;	CVB5_5Y2,CVB5_5Y
novel_circ_0013033	+	ENSG00000169379;	CVB5_5Y2,CVB5_5Y
novel_circ_0010031	-	ENSG00000135966;	CVB5_5Y2,CVB5_5Y
hsa_circ_0027775	+	ENSG00000111142;	CVB5_5Y2,CVB5_5Y
hsa_circ_0008451	-	ENSG00000110713;	CVB5_5Y2,CVB5_5Y
hsa_circ_0007866	-	ENSG00000234857;ENSG0	CVB5_5Y2,CVB5_5Y
hsa_circ_0018281	-	ENSG00000172671;	CVB5_5Y2,CVB5_5Y
hsa_circ_0006243	-	ENSG00000225830;	CVB5_5Y2,CVB5_5Y
hsa_circ_0008874	+	ENSG00000108239;	CVB5_5Y2,CVB5_5Y
hsa_circ_0003638	+	ENSG00000087095;	CVB5_5Y2,CVB5_5Y
hsa_circ_0043280	+	ENSG00000276234;	CVB5_5Y2,CVB5_5Y
hsa_circ_0006631	-	ENSG00000074755;	CVB5_5Y2,CVB5_5Y
hsa_circ_0006255	-	ENSG00000108840;	CVB5_5Y2,CVB5_5Y
hsa_circ_0045931	-	ENSG00000055483;	CVB5_5Y2,CVB5_5Y
hsa_circ_0004519	-	ENSG00000168411;	CVB5_5Y2,CVB5_5Y
hsa_circ_0004033	-	ENSG00000159322;	CVB5_5Y2,CVB5_5Y
hsa_circ_0031615	-	ENSG00000198604;	CVB5_5Y2,CVB5_5Y
hsa_circ_0051399	+	ENSG00000142273;	CVB5_5Y2,CVB5_5Y
hsa_circ_0060745	+	ENSG00000124207;	CVB5_5Y2,CVB5_5Y
hsa_circ_0004343	-	ENSG00000002822;	CVB5_5Y2,CVB5_5Y
hsa_circ_0007823	+	ENSG00000106346;	CVB5_5Y2,CVB5_5Y
hsa_circ_0005629	-	ENSG00000135074;	CVB5_5Y2,CVB5_5Y
hsa_circ_0075143	+	ENSG00000113761;	CVB5_5Y2,CVB5_5Y
hsa_circ_0071189	-	ENSG00000198589;	CVB5_5Y2,CVB5_5Y
hsa_circ_0006498	+	ENSG00000153767;	CVB5_5Y2,CVB5_5Y
hsa_circ_0067013	+	ENSG00000065485;	CVB5_5Y2,CVB5_5Y
hsa_circ_0057041	-	ENSG00000123600;	CVB5_5Y2,CVB5_5Y
hsa_circ_0008979	-	ENSG00000180398;	CVB5_5Y2,CVB5_5Y

hsa_circ_0000015	+	ENSG00000251503;	CVB5_5Y2,CVB5_5Y
hsa_circ_0015837	+	ENSG00000118200;	CVB5_5Y2,CVB5_5Y
hsa_circ_0004493	-	ENSG00000127483;	CVB5_5Y2,CVB5_5Y
hsa_circ_0006556	+	ENSG00000137145;	CVB5_5Y2,CVB5_5Y
hsa_circ_0084663	+	ENSG00000104218;	CVB5_5Y2,CVB5_5Y
novel_circ_0003076	-	ENSG00000123200;	CVB5_5Y2,CVB5_5Y
novel_circ_0002050	+	ENSG00000082805;	CVB5_5Y2,CVB5_5Y
novel_circ_0005737	-	n/a	CVB5_5Y2,CVB5_5Y
novel_circ_0006129	-	ENSG00000157637;	CVB5_5Y2,CVB5_5Y
novel_circ_0009591	+	ENSG00000182670;	CVB5_5Y2,CVB5_5Y
novel_circ_0015933	+	ENSG00000106443;	CVB5_5Y2,CVB5_5Y
novel_circ_0016332	+	ENSG00000106052;	CVB5_5Y2,CVB5_5Y
novel_circ_0012547	-	ENSG00000235493;	CVB5_5Y2,CVB5_5Y
novel_circ_0011668	+	ENSG00000153561;	CVB5_5Y2,CVB5_5Y
novel_circ_0007770	+	ENSG00000134371;	CVB5_5Y2,CVB5_5Y
novel_circ_0008577	-	ENSG00000123473;	CVB5_5Y2,CVB5_5Y
novel_circ_0018377	+	ENSG00000119125;	CVB5_5Y2,CVB5_5Y
novel_circ_0016873	-	ENSG00000254093;	CVB5_5Y2,CVB5_5Y
hsa_circ_0030117	-	ENSG00000102763;	CVB5_5Y2,CVB5_5Y
hsa_circ_0002154	+	ENSG00000111785;	CVB5_5Y2,CVB5_5Y
hsa_circ_0007468	-	ENSG00000110851;	CVB5_5Y2,CVB5_5Y
hsa_circ_0007776	-	ENSG00000089234;	CVB5_5Y2,CVB5_5Y
hsa_circ_0005310	-	ENSG00000139697;	CVB5_5Y2,CVB5_5Y
hsa_circ_0002826	+	ENSG00000112787;	CVB5_5Y2,CVB5_5Y
hsa_circ_0027618	-	ENSG00000091039;	CVB5_5Y2,CVB5_5Y
hsa_circ_0024295	+	ENSG00000150768;	CVB5_5Y2,CVB5_5Y
hsa_circ_0020670	+	ENSG00000070047;	CVB5_5Y2,CVB5_5Y
hsa_circ_0022308	-	ENSG00000167986;	CVB5_5Y2,CVB5_5Y
hsa_circ_0019610	-	ENSG00000198408;	CVB5_5Y2,CVB5_5Y
hsa_circ_0000235	+	ENSG00000108100;	CVB5_5Y2,CVB5_5Y
hsa_circ_0000239	-	ENSG00000204130;	CVB5_5Y2,CVB5_5Y
hsa_circ_0008102	+	ENSG00000196233;	CVB5_5Y2,CVB5_5Y
hsa_circ_0041152	-	ENSG00000181031;	CVB5_5Y2,CVB5_5Y
hsa_circ_0005320	+	ENSG00000184640;	CVB5_5Y2,CVB5_5Y
hsa_circ_0005606	+	ENSG00000007545;	CVB5_5Y2,CVB5_5Y
hsa_circ_0006795	-	ENSG00000103550;	CVB5_5Y2,CVB5_5Y
hsa_circ_0004603	+	ENSG00000162063;	CVB5_5Y2,CVB5_5Y
hsa_circ_0002439	-	ENSG00000090447;	CVB5_5Y2,CVB5_5Y
hsa_circ_0008168	+	ENSG00000174442;	CVB5_5Y2,CVB5_5Y
hsa_circ_0031446	-	ENSG00000196792;	CVB5_5Y2,CVB5_5Y
hsa_circ_0008554	-	ENSG00000197256;	CVB5_5Y2,CVB5_5Y
hsa_circ_0006941	-	ENSG00000205517;	CVB5_5Y2,CVB5_5Y
hsa_circ_0007180	+	ENSG00000101751;	CVB5_5Y2,CVB5_5Y
hsa_circ_0001202	+	ENSG00000237438;	CVB5_5Y2,CVB5_5Y
hsa_circ_0062577	+	ENSG00000099991;	CVB5_5Y2,CVB5_5Y
hsa_circ_0004868	-	ENSG00000158470;	CVB5_5Y2,CVB5_5Y
hsa_circ_0004770	-	ENSG00000054793;	CVB5_5Y2,CVB5_5Y
hsa_circ_0001730	-	ENSG00000196411;	CVB5_5Y2,CVB5_5Y
hsa_circ_0079422	-	ENSG00000003147;	CVB5_5Y2,CVB5_5Y
hsa_circ_0006715	+	ENSG00000120254;	CVB5_5Y2,CVB5_5Y
hsa_circ_0078539	-	ENSG00000092820;	CVB5_5Y2,CVB5_5Y
hsa_circ_0075917	-	ENSG00000204623;	CVB5_5Y2,CVB5_5Y
hsa_circ_0001571	+	ENSG00000137275;	CVB5_5Y2,CVB5_5Y
hsa_circ_0001603	+	ENSG00000024048;	CVB5_5Y2,CVB5_5Y
hsa_circ_0073748	+	ENSG00000113368;	CVB5_5Y2,CVB5_5Y
hsa_circ_0008595	+	ENSG00000281398;ENSG0	CVB5_5Y2,CVB5_5Y
hsa_circ_0002785	-	ENSG00000152990;	CVB5_5Y2,CVB5_5Y
hsa_circ_0003985	+	ENSG00000174799;	CVB5_5Y2,CVB5_5Y

hsa_circ_0002004	+	ENSG00000114416;	CVB5_5Y2,CVB5_5Y
hsa_circ_0002266	+	ENSG00000186001;	CVB5_5Y2,CVB5_5Y
hsa_circ_0001289	-	ENSG00000181555;	CVB5_5Y2,CVB5_5Y
hsa_circ_0065243	-	ENSG00000173473;	CVB5_5Y2,CVB5_5Y
hsa_circ_0065871	+	ENSG00000114738;	CVB5_5Y2,CVB5_5Y
hsa_circ_0004862	+	ENSG00000144535;	CVB5_5Y2,CVB5_5Y
hsa_circ_0001018	-	ENSG00000115484;	CVB5_5Y2,CVB5_5Y
hsa_circ_0013743	+	ENSG00000198162;	CVB5_5Y2,CVB5_5Y
hsa_circ_0003942	+	ENSG00000135837;	CVB5_5Y2,CVB5_5Y
hsa_circ_0000166	+	ENSG00000157181;	CVB5_5Y2,CVB5_5Y
hsa_circ_0017241	+	ENSG00000054282;	CVB5_5Y2,CVB5_5Y
hsa_circ_0005174	-	ENSG00000197989;	CVB5_5Y2,CVB5_5Y
hsa_circ_0008389	-	ENSG00000007923;	CVB5_5Y2,CVB5_5Y
hsa_circ_0001905	+	ENSG00000181090;	CVB5_5Y2,CVB5_5Y
hsa_circ_0007367	-	ENSG00000137073;	CVB5_5Y2,CVB5_5Y
hsa_circ_0003066	-	ENSG00000169398;	CVB5_5Y2,CVB5_5Y
hsa_circ_0083465	+	ENSG00000078674;	CVB5_5Y2,CVB5_5Y
hsa_circ_0002961	+	ENSG00000147649;	CVB5_5Y2,CVB5_5Y
novel_circ_0003070	-	ENSG00000123200;	CVB5_5Y2,CVB5_5Y
novel_circ_0001028	-	ENSG00000110315;	CVB5_5Y2,CVB5_5Y
novel_circ_0001525	+	ENSG00000251562;	CVB5_5Y2,CVB5_5Y
novel_circ_0001550	-	ENSG00000110066;	CVB5_5Y2,CVB5_5Y
novel_circ_0001562	+	ENSG00000110075;	CVB5_5Y2,CVB5_5Y
novel_circ_0005791	-	ENSG00000266086;ENSG0	CVB5_5Y2,CVB5_5Y
novel_circ_0005184	+	ENSG00000158545;	CVB5_5Y2,CVB5_5Y
novel_circ_0003942	-	ENSG00000140470;	CVB5_5Y2,CVB5_5Y
novel_circ_0004529	+	ENSG00000140525;	CVB5_5Y2,CVB5_5Y
novel_circ_0003684	+	ENSG00000054654;	CVB5_5Y2,CVB5_5Y
novel_circ_0006699	+	ENSG00000099622;	CVB5_5Y2,CVB5_5Y
novel_circ_0016348	-	ENSG00000196295;	CVB5_5Y2,CVB5_5Y
novel_circ_0012458	-	ENSG00000163961;	CVB5_5Y2,CVB5_5Y
novel_circ_0012782	-	ENSG00000173473;	CVB5_5Y2,CVB5_5Y
novel_circ_0011626	+	n/a	CVB5_5Y2,CVB5_5Y
novel_circ_0017032	-	ENSG00000129295;	CVB5_5Y2,CVB5_5Y
novel_circ_0017572	-	ENSG00000076554;ENSG0	CVB5_5Y2,CVB5_5Y
hsa_circ_0009152	+	ENSG00000172766;	CVB5_5Y2,CVB5_5Y
hsa_circ_0006104	-	ENSG00000175203;	CVB5_5Y2,CVB5_5Y
hsa_circ_0009130	-	ENSG00000064932;	CVB5_5Y2,CVB5_5Y
hsa_circ_0005837	+	ENSG00000276043;	CVB5_5Y2,CVB5_5Y
hsa_circ_0006476	-	ENSG00000197961;	CVB5_5Y2,CVB5_5Y
hsa_circ_0005133	+	ENSG00000204580;	CVB5_5Y2,CVB5_5Y
hsa_circ_0005372	+	ENSG00000155903;	CVB5_5Y2,CVB5_5Y
hsa_circ_0064789	-	ENSG00000163539;	CVB5_5Y2,CVB5_5Y
hsa_circ_0004340	-	ENSG00000144736;	CVB5_5Y2,CVB5_5Y
hsa_circ_0004883	-	ENSG00000136861;	CVB5_5Y2,CVB5_5Y
novel_circ_0004345	+	ENSG00000188779;	CVB5_5Y2,CVB5_5Y
novel_circ_0013744	+	ENSG00000134851;	CVB5_5Y2,CVB5_5Y
novel_circ_0012325	+	ENSG00000131374;	CVB5_5Y2,CVB5_5Y
novel_circ_0008195	+	ENSG00000077585;	CVB5_5Y2,CVB5_5Y
novel_circ_0018100	-	ENSG00000165724;	CVB5_5Y2,CVB5_5Y
hsa_circ_0091175	-	ENSG00000165288;	CVB5_5Y2,CVB5_5Y
hsa_circ_0022591	+	ENSG00000168003;	CVB5_5Y2,CVB5_5Y
hsa_circ_0002042	-	ENSG00000151240;	CVB5_5Y2,CVB5_5Y
hsa_circ_0040786	-	ENSG00000104731;	CVB5_5Y2,CVB5_5Y
hsa_circ_0031357	-	ENSG00000260669;ENSG0	CVB5_5Y2,CVB5_5Y
hsa_circ_0031359	-	ENSG00000254692;ENSG0	CVB5_5Y2,CVB5_5Y
hsa_circ_0002324	-	ENSG00000160392;	CVB5_5Y2,CVB5_5Y
hsa_circ_0008809	+	ENSG00000106263;	CVB5_5Y2,CVB5_5Y

hsa_circ_0067163	-	ENSG00000163870;	CVB5_5Y2,CVB5_5Y
hsa_circ_0006840	-	ENSG00000136527;	CVB5_5Y2,CVB5_5Y
hsa_circ_0068462	+	ENSG00000156976;	CVB5_5Y2,CVB5_5Y
hsa_circ_0002229	+	ENSG00000134324;	CVB5_5Y2,CVB5_5Y
hsa_circ_0057725	-	ENSG00000013441;	CVB5_5Y2,CVB5_5Y
hsa_circ_0009910	+	ENSG00000116688;	CVB5_5Y2,CVB5_5Y
hsa_circ_0007110	+	ENSG00000137145;	CVB5_5Y2,CVB5_5Y
hsa_circ_0006915	-	ENSG00000040341;	CVB5_5Y2,CVB5_5Y
novel_circ_0000460	+	ENSG00000285737;	CVB5_5Y2,CVB5_5Y
novel_circ_0000821	-	ENSG00000148606;	CVB5_5Y2,CVB5_5Y
novel_circ_0005371	-	ENSG00000070444;	CVB5_5Y2,CVB5_5Y
novel_circ_0005792	+	ENSG00000264364;	CVB5_5Y2,CVB5_5Y
novel_circ_0003487	-	ENSG00000198604;	CVB5_5Y2,CVB5_5Y
novel_circ_0003759	+	ENSG00000119707;	CVB5_5Y2,CVB5_5Y
novel_circ_0006849	-	ENSG00000121289;	CVB5_5Y2,CVB5_5Y
novel_circ_0015108	-	ENSG00000112339;	CVB5_5Y2,CVB5_5Y
novel_circ_0012809	+	ENSG00000177479;	CVB5_5Y2,CVB5_5Y
novel_circ_0008291	-	ENSG00000162714;	CVB5_5Y2,CVB5_5Y
novel_circ_0008741	+	ENSG00000132849;	CVB5_5Y2,CVB5_5Y
novel_circ_0018209	-	ENSG00000080608;	CVB5_5Y2,CVB5_5Y
hsa_circ_0091768	-	ENSG00000185825;	CVB5_5Y2,CVB5_5Y
hsa_circ_0089974	+	ENSG00000188158;	CVB5_5Y2,CVB5_5Y
hsa_circ_0027774	+	ENSG00000111142;	CVB5_5Y2,CVB5_5Y
hsa_circ_0005285	-	ENSG00000107554;	CVB5_5Y2,CVB5_5Y
hsa_circ_0004678	+	ENSG00000159202;	CVB5_5Y2,CVB5_5Y
hsa_circ_0005330	+	ENSG00000189091;	CVB5_5Y2,CVB5_5Y
hsa_circ_0004866	+	ENSG00000140455;	CVB5_5Y2,CVB5_5Y
hsa_circ_0004386	+	ENSG00000189042;	CVB5_5Y2,CVB5_5Y
hsa_circ_0005317	+	ENSG00000130529;	CVB5_5Y2,CVB5_5Y
hsa_circ_0063604	+	ENSG00000100147;	CVB5_5Y2,CVB5_5Y
hsa_circ_0081746	+	ENSG00000105819;	CVB5_5Y2,CVB5_5Y
hsa_circ_0079929	+	ENSG00000065883;	CVB5_5Y2,CVB5_5Y
hsa_circ_0078241	-	ENSG00000120253;	CVB5_5Y2,CVB5_5Y
hsa_circ_0075872	+	ENSG00000079691;	CVB5_5Y2,CVB5_5Y
hsa_circ_0005153	+	ENSG00000153113;	CVB5_5Y2,CVB5_5Y
hsa_circ_0003461	+	ENSG00000151718;	CVB5_5Y2,CVB5_5Y
hsa_circ_0002554	-	ENSG00000113812;	CVB5_5Y2,CVB5_5Y
hsa_circ_0066290	+	ENSG00000136068;	CVB5_5Y2,CVB5_5Y
hsa_circ_0009028	+	ENSG00000198162;	CVB5_5Y2,CVB5_5Y
hsa_circ_0010146	-	ENSG00000037637;	CVB5_5Y2,CVB5_5Y
hsa_circ_0010458	-	ENSG00000075151;	CVB5_5Y2,CVB5_5Y
hsa_circ_0011115	-	ENSG00000158161;	CVB5_5Y2,CVB5_5Y
novel_circ_0001598	+	ENSG00000131626;	CVB5_5Y2,CVB5_5Y
novel_circ_0004104	-	ENSG00000140259;	CVB5_5Y2,CVB5_5Y
novel_circ_0007139	-	ENSG00000125731;	CVB5_5Y2,CVB5_5Y
novel_circ_0016274	+	ENSG00000169193;	CVB5_5Y2,CVB5_5Y
novel_circ_0016650	+	ENSG00000187257;	CVB5_5Y2,CVB5_5Y
novel_circ_0015807	-	ENSG00000123552;	CVB5_5Y2,CVB5_5Y
novel_circ_0014516	+	ENSG00000164190;	CVB5_5Y2,CVB5_5Y
novel_circ_0013034	+	ENSG00000168137;	CVB5_5Y2,CVB5_5Y
novel_circ_0007952	-	ENSG00000090686;	CVB5_5Y2,CVB5_5Y
novel_circ_0008184	+	ENSG00000285053;ENSG0	CVB5_5Y2,CVB5_5Y
hsa_circ_0008902	+	ENSG00000152520;	CVB5_5Y2,CVB5_5Y
hsa_circ_0004766	-	ENSG00000100814;	CVB5_5Y2,CVB5_5Y
hsa_circ_0049282	-	ENSG00000129354;	CVB5_5Y2,CVB5_5Y
hsa_circ_0002462	+	ENSG00000172795;	CVB5_5Y2,CVB5_5Y
hsa_circ_0072271	-	ENSG00000113569;	CVB5_5Y2,CVB5_5Y
hsa_circ_0069249	-	ENSG00000151552;	CVB5_5Y2,CVB5_5Y

hsa_circ_0008062	+	ENSG00000172939;	CVB5_5Y2,CVB5_5Y
hsa_circ_0065258	-	ENSG00000173473;	CVB5_5Y2,CVB5_5Y
hsa_circ_0016733	-	ENSG00000143776;	CVB5_5Y2,CVB5_5Y
hsa_circ_0004834	+	ENSG00000270106;ENSG0	CVB5_5Y2,CVB5_5Y
hsa_circ_0007617	+	ENSG00000121766;	CVB5_5Y2,CVB5_5Y
hsa_circ_0001880	+	ENSG00000119318;	CVB5_5Y2,CVB5_5Y
novel_circ_0018731	-	ENSG00000181544;	CVB5_5Y2,CVB5_5Y
novel_circ_0014596	+	ENSG00000164258;	CVB5_5Y2,CVB5_5Y
novel_circ_0010613	-	ENSG00000115942;	CVB5_5Y2,CVB5_5Y
novel_circ_0009026	+	ENSG00000117528;	CVB5_5Y2,CVB5_5Y
hsa_circ_0006598	-	ENSG00000185291;	CVB5_5Y3
hsa_circ_0005518	-	ENSG00000086758;	CVB5_5Y3
hsa_circ_0091038	+	ENSG00000147162;	CVB5_5Y3
hsa_circ_0029772	-	ENSG00000102699;	CVB5_5Y3
hsa_circ_0030207	+	ENSG00000136141;	CVB5_5Y3
hsa_circ_0030676	+	ENSG00000065150;	CVB5_5Y3
hsa_circ_0027867	+	ENSG00000136021;	CVB5_5Y3
hsa_circ_0029348	-	ENSG00000073060;	CVB5_5Y3
hsa_circ_0025851	+	ENSG00000087470;	CVB5_5Y3
hsa_circ_0026240	+	ENSG00000161813;	CVB5_5Y3
hsa_circ_0004538	-	ENSG00000110925;	CVB5_5Y3
hsa_circ_0026626	+	ENSG00000185591;	CVB5_5Y3
hsa_circ_0027380	+	ENSG00000196935;	CVB5_5Y3
hsa_circ_0027459	-	ENSG00000111554;	CVB5_5Y3
hsa_circ_0027502	+	ENSG00000111605;	CVB5_5Y3
hsa_circ_0005612	+	ENSG00000149311;	CVB5_5Y3
hsa_circ_0024296	+	ENSG00000150768;	CVB5_5Y3
hsa_circ_0003509	-	ENSG00000149187;	CVB5_5Y3
hsa_circ_0022723	-	ENSG00000110047;	CVB5_5Y3
hsa_circ_0023885	-	ENSG00000073921;	CVB5_5Y3
hsa_circ_0023925	-	ENSG00000073921;	CVB5_5Y3
hsa_circ_0006555	-	ENSG00000166169;	CVB5_5Y3
hsa_circ_0002676	-	ENSG00000065154;	CVB5_5Y3
hsa_circ_0008123	+	ENSG00000107938;	CVB5_5Y3
hsa_circ_0000225	+	ENSG00000120539;	CVB5_5Y3
hsa_circ_0018074	-	ENSG00000107951;	CVB5_5Y3
hsa_circ_0018196	+	ENSG00000095794;	CVB5_5Y3
hsa_circ_0018882	+	ENSG00000035403;	CVB5_5Y3
hsa_circ_0018889	+	ENSG00000035403;	CVB5_5Y3
hsa_circ_0019240	-	ENSG00000173145;	CVB5_5Y3
hsa_circ_0019488	-	ENSG00000107554;	CVB5_5Y3
hsa_circ_0041290	-	ENSG00000174231;	CVB5_5Y3
hsa_circ_0042341	-	ENSG00000176974;	CVB5_5Y3
hsa_circ_0041383	-	ENSG00000070366;	CVB5_5Y3
hsa_circ_0005336	+	ENSG00000109046;	CVB5_5Y3
hsa_circ_0043695	-	ENSG00000141698;	CVB5_5Y3
hsa_circ_0007272	-	ENSG00000170832;	CVB5_5Y3
hsa_circ_0045041	-	ENSG00000108506;	CVB5_5Y3
hsa_circ_0045698	+	ENSG00000132470;	CVB5_5Y3
hsa_circ_0046264	-	ENSG00000185624;	CVB5_5Y3
hsa_circ_0046430	+	ENSG00000141568;	CVB5_5Y3
hsa_circ_0038073	+	ENSG00000103222;	CVB5_5Y3
hsa_circ_0006663	-	ENSG00000007392;	CVB5_5Y3
hsa_circ_0039202	-	ENSG00000069329;	CVB5_5Y3
hsa_circ_0040559	-	ENSG00000065427;	CVB5_5Y3
hsa_circ_0034762	+	ENSG00000137802;	CVB5_5Y3
hsa_circ_0003829	+	ENSG00000166734;	CVB5_5Y3
hsa_circ_0035818	+	ENSG00000028528;	CVB5_5Y3

hsa_circ_0004506	-	ENSG00000090487;	CVB5_5Y3
hsa_circ_0004393	-	ENSG00000174485;	CVB5_5Y3
hsa_circ_0003166	+	ENSG00000137807;	CVB5_5Y3
hsa_circ_0007103	-	ENSG00000169375;	CVB5_5Y3
hsa_circ_0036768	+	ENSG00000185033;	CVB5_5Y3
hsa_circ_0033598	+	ENSG00000179364;	CVB5_5Y3
hsa_circ_0031114	-	ENSG00000129566;	CVB5_5Y3
hsa_circ_0031725	-	ENSG00000100934;	CVB5_5Y3
hsa_circ_0031845	-	ENSG00000087299;	CVB5_5Y3
hsa_circ_0032496	+	ENSG00000119707;	CVB5_5Y3
hsa_circ_0032704	+	ENSG00000119685;	CVB5_5Y3
hsa_circ_0032740	+	ENSG00000089916;	CVB5_5Y3
hsa_circ_0049383	+	ENSG00000130164;	CVB5_5Y3
hsa_circ_0049434	-	ENSG00000130158;	CVB5_5Y3
hsa_circ_0048454	+	ENSG00000125912;	CVB5_5Y3
hsa_circ_0000941	+	ENSG00000007047;	CVB5_5Y3
hsa_circ_0046995	+	ENSG00000101639;	CVB5_5Y3
hsa_circ_0047749	+	ENSG00000091157;	CVB5_5Y3
hsa_circ_0062239	-	ENSG00000242259;ENSG0	CVB5_5Y3
hsa_circ_0063327	-	ENSG00000100201;	CVB5_5Y3
hsa_circ_0063707	+	ENSG00000188677;	CVB5_5Y3
hsa_circ_0059187	-	ENSG00000256566;ENSG0	CVB5_5Y3
hsa_circ_0059858	+	ENSG00000078699;	CVB5_5Y3
hsa_circ_0001139	+	ENSG00000125970;	CVB5_5Y3
hsa_circ_0060043	-	ENSG00000088298;	CVB5_5Y3
hsa_circ_0006158	+	ENSG00000156273;	CVB5_5Y3
hsa_circ_0006075	+	ENSG00000182670;	CVB5_5Y3
hsa_circ_0081739	-	ENSG00000161048;	CVB5_5Y3
hsa_circ_0081863	+	ENSG00000135249;	CVB5_5Y3
hsa_circ_0082401	+	ENSG00000128585;	CVB5_5Y3
hsa_circ_0082854	-	ENSG00000197362;	CVB5_5Y3
hsa_circ_0001770	-	ENSG00000055609;	CVB5_5Y3
hsa_circ_0079519	-	ENSG00000071189;	CVB5_5Y3
hsa_circ_0079766	+	ENSG00000105778;	CVB5_5Y3
hsa_circ_0080059	+	ENSG00000105953;	CVB5_5Y3
hsa_circ_0079327	-	ENSG00000086232;	CVB5_5Y3
hsa_circ_0079377	+	ENSG00000136247;	CVB5_5Y3
hsa_circ_0079390	+	ENSG00000146576;	CVB5_5Y3
hsa_circ_0080881	+	ENSG00000127955;	CVB5_5Y3
hsa_circ_0079085	+	ENSG00000239857;ENSG0	CVB5_5Y3
hsa_circ_0081026	-	ENSG00000127980;	CVB5_5Y3
hsa_circ_0081301	+	ENSG00000196367;	CVB5_5Y3
hsa_circ_0077648	-	ENSG00000009413;	CVB5_5Y3
hsa_circ_0077726	-	ENSG00000047932;	CVB5_5Y3
hsa_circ_0077913	-	ENSG00000135541;	CVB5_5Y3
hsa_circ_0001984	+	ENSG00000120265;	CVB5_5Y3
hsa_circ_0078674	+	ENSG00000130396;	CVB5_5Y3
hsa_circ_0078737	-	ENSG00000184465;	CVB5_5Y3
hsa_circ_0078773	+	ENSG00000112584;	CVB5_5Y3
hsa_circ_0076155	-	ENSG00000096060;	CVB5_5Y3
hsa_circ_0076747	-	ENSG00000112118;	CVB5_5Y3
hsa_circ_0001612	+	ENSG00000112701;	CVB5_5Y3
hsa_circ_0001622	+	ENSG00000118412;	CVB5_5Y3
hsa_circ_0077408	-	ENSG00000146263;	CVB5_5Y3
hsa_circ_0073691	+	ENSG00000205302;	CVB5_5Y3
hsa_circ_0073747	+	ENSG00000113368;	CVB5_5Y3
hsa_circ_0074206	+	ENSG00000015479;ENSG0	CVB5_5Y3
hsa_circ_0008731	-	ENSG00000177058;	CVB5_5Y3

hsa_circ_0072540	+	ENSG00000062194;	CVB5_5Y3
hsa_circ_0072548	+	ENSG00000062194;	CVB5_5Y3
hsa_circ_0072732	+	ENSG00000112851;	CVB5_5Y3
hsa_circ_0071758	-	ENSG00000037474;	CVB5_5Y3
hsa_circ_0006859	-	ENSG00000171617;	CVB5_5Y3
hsa_circ_0008408	+	ENSG00000109685;	CVB5_5Y3
hsa_circ_0069776	+	ENSG00000134851;	CVB5_5Y3
hsa_circ_0069819	+	ENSG00000174799;	CVB5_5Y3
hsa_circ_0070000	-	ENSG00000138757;	CVB5_5Y3
hsa_circ_0004334	-	ENSG00000138674;	CVB5_5Y3
hsa_circ_0067322	-	ENSG00000172765;	CVB5_5Y3
hsa_circ_0067619	-	ENSG00000114127;	CVB5_5Y3
hsa_circ_0067627	-	ENSG00000175054;	CVB5_5Y3
hsa_circ_0067680	+	ENSG00000181744;	CVB5_5Y3
hsa_circ_0067880	+	ENSG00000008952;	CVB5_5Y3
hsa_circ_0006893	-	ENSG00000173889;	CVB5_5Y3
hsa_circ_0065649	-	ENSG00000067560;	CVB5_5Y3
hsa_circ_0008107	-	ENSG00000168374;	CVB5_5Y3
hsa_circ_0066273	+	ENSG00000163681;	CVB5_5Y3
hsa_circ_0052602	-	ENSG00000115758;	CVB5_5Y3
hsa_circ_0056285	+	ENSG00000144118;	CVB5_5Y3
hsa_circ_0005892	-	ENSG00000144233;	CVB5_5Y3
hsa_circ_0006445	+	ENSG00000115183;	CVB5_5Y3
hsa_circ_0008427	+	ENSG00000144426;	CVB5_5Y3
hsa_circ_0004778	-	ENSG00000055917;	CVB5_5Y3
hsa_circ_0008091	-	ENSG00000023228;	CVB5_5Y3
hsa_circ_0058905	-	ENSG00000068024;	CVB5_5Y3
hsa_circ_0054609	-	ENSG00000085760;	CVB5_5Y3
hsa_circ_0008361	+	ENSG00000162929;	CVB5_5Y3
hsa_circ_0054961	+	ENSG00000119844;	CVB5_5Y3
hsa_circ_0002234	-	ENSG00000134222;	CVB5_5Y3
hsa_circ_0009964	+	ENSG00000048707;	CVB5_5Y3
hsa_circ_0005948	+	ENSG00000143624;	CVB5_5Y3
hsa_circ_0010112	+	ENSG00000065526;	CVB5_5Y3
hsa_circ_0015257	+	ENSG00000010165;	CVB5_5Y3
hsa_circ_0015489	+	ENSG00000135837;	CVB5_5Y3
hsa_circ_0000007	-	ENSG00000078369;	CVB5_5Y3
hsa_circ_0000027	-	ENSG00000117298;	CVB5_5Y3
hsa_circ_0016485	-	ENSG00000136628;	CVB5_5Y3
hsa_circ_0007543	+	ENSG00000054282;	CVB5_5Y3
hsa_circ_0017348	-	ENSG00000196418;	CVB5_5Y3
hsa_circ_0011422	+	ENSG00000254553;ENSG0	CVB5_5Y3
hsa_circ_0011543	+	ENSG00000146463;	CVB5_5Y3
hsa_circ_0011625	+	ENSG00000092847;	CVB5_5Y3
hsa_circ_0012476	-	ENSG00000078618;	CVB5_5Y3
hsa_circ_0009472	-	ENSG00000131697;	CVB5_5Y3
hsa_circ_0012930	+	ENSG00000116754;	CVB5_5Y3
hsa_circ_0013218	-	ENSG00000067334;	CVB5_5Y3
hsa_circ_0088025	-	ENSG00000136813;	CVB5_5Y3
hsa_circ_0088563	+	ENSG00000136933;	CVB5_5Y3
hsa_circ_0088581	+	ENSG00000165219;	CVB5_5Y3
hsa_circ_0089277	-	ENSG00000107290;	CVB5_5Y3
hsa_circ_0005118	+	ENSG00000240498;	CVB5_5Y3
hsa_circ_0086600	-	ENSG00000137055;	CVB5_5Y3
hsa_circ_0001837	+	ENSG00000120158;	CVB5_5Y3
hsa_circ_0087162	-	ENSG00000188647;	CVB5_5Y3
hsa_circ_0085567	-	ENSG00000153310;	CVB5_5Y3
hsa_circ_0083807	+	ENSG00000012232;	CVB5_5Y3

hsa_circ_0084318	-	ENSG00000253729;	CVB5_5Y3
hsa_circ_0002562	+	ENSG00000285791;	CVB5_5Y3
novel_circ_0018745	-	ENSG00000198910;	CVB5_5Y3
novel_circ_0018871	-	ENSG00000131089;	CVB5_5Y3
novel_circ_0003065	-	ENSG00000123200;	CVB5_5Y3
novel_circ_0003174	-	ENSG00000136111;	CVB5_5Y3
novel_circ_0001990	-	ENSG00000173064;	CVB5_5Y3
novel_circ_0002007	+	ENSG00000111331;	CVB5_5Y3
novel_circ_0002200	+	ENSG00000082805;	CVB5_5Y3
novel_circ_0002548	-	ENSG00000166881;	CVB5_5Y3
novel_circ_0002575	+	ENSG00000196935;	CVB5_5Y3
novel_circ_0002614	+	ENSG00000111581;	CVB5_5Y3
novel_circ_0002629	+	ENSG00000135679;	CVB5_5Y3
novel_circ_0002651	+	ENSG00000258168;	CVB5_5Y3
novel_circ_0002666	-	ENSG00000180881;	CVB5_5Y3
novel_circ_0001049	+	ENSG00000166266;	CVB5_5Y3
novel_circ_0001360	+	ENSG00000026508;	CVB5_5Y3
novel_circ_0001393	-	ENSG00000110713;	CVB5_5Y3
novel_circ_0001532	+	ENSG00000174516;	CVB5_5Y3
novel_circ_0001565	+	ENSG00000110075;	CVB5_5Y3
novel_circ_0001573	+	ENSG00000110075;	CVB5_5Y3
novel_circ_0000005	+	ENSG00000166135;	CVB5_5Y3
novel_circ_0000021	-	ENSG00000186862;	CVB5_5Y3
novel_circ_0000032	-	ENSG00000198408;	CVB5_5Y3
novel_circ_0000187	-	ENSG00000107581;	CVB5_5Y3
novel_circ_0000341	-	ENSG00000148773;	CVB5_5Y3
novel_circ_0000382	-	ENSG00000152465;	CVB5_5Y3
novel_circ_0000516	-	ENSG00000170759;	CVB5_5Y3
novel_circ_0000608	-	ENSG00000227345;	CVB5_5Y3
novel_circ_0000632	+	ENSG00000108021;	CVB5_5Y3
novel_circ_0000688	-	ENSG00000108187;	CVB5_5Y3
novel_circ_0000700	-	ENSG00000138346;	CVB5_5Y3
novel_circ_0000752	+	ENSG00000099284;	CVB5_5Y3
novel_circ_0000911	+	ENSG00000138160;	CVB5_5Y3
novel_circ_0000912	+	ENSG00000138160;	CVB5_5Y3
novel_circ_0000984	+	ENSG00000023839;	CVB5_5Y3
novel_circ_0005278	-	ENSG00000175106;	CVB5_5Y3
novel_circ_0005307	-	ENSG00000186532;	CVB5_5Y3
novel_circ_0005335	+	ENSG00000072134;	CVB5_5Y3
novel_circ_0005452	+	ENSG00000196712;	CVB5_5Y3
novel_circ_0005572	+	ENSG00000141756;	CVB5_5Y3
novel_circ_0005712	+	ENSG00000108846;	CVB5_5Y3
novel_circ_0005767	-	ENSG00000005100;	CVB5_5Y3
novel_circ_0005897	-	ENSG00000108510;	CVB5_5Y3
novel_circ_0005921	-	ENSG00000008283;	CVB5_5Y3
novel_circ_0005937	-	ENSG00000256525;	CVB5_5Y3
novel_circ_0006055	-	ENSG00000108469;	CVB5_5Y3
novel_circ_0006160	-	ENSG00000169710;	CVB5_5Y3
novel_circ_0004602	-	ENSG00000188897;	CVB5_5Y3
novel_circ_0004647	+	ENSG00000072864;	CVB5_5Y3
novel_circ_0004685	-	ENSG00000260342;ENSG0	CVB5_5Y3
novel_circ_0004817	+	ENSG00000080603;ENSG0	CVB5_5Y3
novel_circ_0004845	-	ENSG00000005339;	CVB5_5Y3
novel_circ_0004902	-	ENSG00000118898;	CVB5_5Y3
novel_circ_0004904	+	ENSG00000155393;	CVB5_5Y3
novel_circ_0004103	-	ENSG00000140259;	CVB5_5Y3
novel_circ_0004133	+	ENSG00000185880;	CVB5_5Y3
novel_circ_0004212	-	ENSG00000137776;	CVB5_5Y3

novel_circ_0004264	-	ENSG00000259316;ENSG0	CVB5_5Y3
novel_circ_0004423	-	ENSG00000169371;	CVB5_5Y3
novel_circ_0004491	+	ENSG00000259429;	CVB5_5Y3
novel_circ_0003362	-	ENSG00000126215;	CVB5_5Y3
novel_circ_0003402	-	ENSG00000092036;ENSG0	CVB5_5Y3
novel_circ_0003553	-	ENSG00000165525;	CVB5_5Y3
novel_circ_0003586	-	ENSG00000151748;	CVB5_5Y3
novel_circ_0003720	-	ENSG00000139990;	CVB5_5Y3
novel_circ_0003721	-	ENSG00000139990;	CVB5_5Y3
novel_circ_0003783	+	ENSG00000119725;ENSG0	CVB5_5Y3
novel_circ_0003876	-	ENSG00000015133;	CVB5_5Y3
novel_circ_0003892	-	ENSG00000011114;	CVB5_5Y3
novel_circ_0006744	-	ENSG00000127526;	CVB5_5Y3
novel_circ_0006827	+	n/a	CVB5_5Y3
novel_circ_0006840	+	ENSG00000168813;	CVB5_5Y3
novel_circ_0006928	-	ENSG00000275395;	CVB5_5Y3
novel_circ_0006968	+	ENSG00000167674;	CVB5_5Y3
novel_circ_0006991	-	ENSG00000125755;	CVB5_5Y3
novel_circ_0006997	-	ENSG00000130749;	CVB5_5Y3
novel_circ_0007036	-	ENSG00000197813;	CVB5_5Y3
novel_circ_0007150	+	ENSG00000099783;	CVB5_5Y3
novel_circ_0006386	+	ENSG00000101596;	CVB5_5Y3
novel_circ_0006436	-	ENSG00000078043;	CVB5_5Y3
novel_circ_0009895	+	ENSG00000196236;	CVB5_5Y3
novel_circ_0009898	+	ENSG00000100393;	CVB5_5Y3
novel_circ_0009089	+	ENSG00000132664;	CVB5_5Y3
novel_circ_0009281	+	ENSG00000125779;	CVB5_5Y3
novel_circ_0009289	+	ENSG00000198900;	CVB5_5Y3
novel_circ_0009354	+	ENSG00000124207;	CVB5_5Y3
novel_circ_0009392	-	ENSG00000000419;	CVB5_5Y3
novel_circ_0009481	+	ENSG00000182621;	CVB5_5Y3
novel_circ_0009521	-	ENSG00000156299;	CVB5_5Y3
novel_circ_0009537	-	ENSG00000142207;	CVB5_5Y3
novel_circ_0009576	+	ENSG00000159256;	CVB5_5Y3
novel_circ_0009633	+	ENSG00000285815;ENSG0	CVB5_5Y3
novel_circ_0009650	+	ENSG00000160218;	CVB5_5Y3
novel_circ_0009658	+	ENSG00000197381;	CVB5_5Y3
novel_circ_0015973	-	ENSG00000106344;	CVB5_5Y3
novel_circ_0015989	+	ENSG00000158467;	CVB5_5Y3
novel_circ_0016049	+	ENSG00000157741;	CVB5_5Y3
novel_circ_0016072	-	ENSG00000157764;	CVB5_5Y3
novel_circ_0016291	+	ENSG00000105926;	CVB5_5Y3
novel_circ_0016460	+	ENSG00000132434;	CVB5_5Y3
novel_circ_0016594	-	ENSG00000009954;	CVB5_5Y3
novel_circ_0016604	+	ENSG00000164818;	CVB5_5Y3
novel_circ_0014924	-	n/a	CVB5_5Y3
novel_circ_0014956	+	ENSG00000111845;	CVB5_5Y3
novel_circ_0014975	+	ENSG00000272162;ENSG0	CVB5_5Y3
novel_circ_0015025	-	ENSG00000111885;	CVB5_5Y3
novel_circ_0015296	-	ENSG00000130363;	CVB5_5Y3
novel_circ_0015454	+	ENSG00000096433;	CVB5_5Y3
novel_circ_0015564	-	ENSG00000112118;	CVB5_5Y3
novel_circ_0015703	-	ENSG00000005700;	CVB5_5Y3
novel_circ_0015710	-	ENSG00000118420;	CVB5_5Y3
novel_circ_0015715	+	ENSG00000083097;	CVB5_5Y3
novel_circ_0014093	+	ENSG00000064651;	CVB5_5Y3
novel_circ_0014118	-	ENSG00000131437;	CVB5_5Y3
novel_circ_0014157	+	ENSG00000145833;	CVB5_5Y3

novel_circ_0014198	-	ENSG00000113013;	CVB5_5Y3
novel_circ_0014378	+	ENSG00000113643;	CVB5_5Y3
novel_circ_0014388	+	ENSG00000204764;	CVB5_5Y3
novel_circ_0014464	-	ENSG00000131459;	CVB5_5Y3
novel_circ_0014499	+	ENSG00000113407;	CVB5_5Y3
novel_circ_0014767	+	ENSG00000164252;ENSG0	CVB5_5Y3
novel_circ_0013253	+	ENSG00000164134;	CVB5_5Y3
novel_circ_0013292	+	ENSG00000071205;	CVB5_5Y3
novel_circ_0013341	-	ENSG00000198589;	CVB5_5Y3
novel_circ_0013439	-	ENSG00000163950;	CVB5_5Y3
novel_circ_0013486	+	ENSG00000205129;	CVB5_5Y3
novel_circ_0013568	+	ENSG00000125386;	CVB5_5Y3
novel_circ_0013753	+	ENSG00000090989;	CVB5_5Y3
novel_circ_0013837	-	ENSG00000118816;	CVB5_5Y3
novel_circ_0013919	-	ENSG00000138640;	CVB5_5Y3
novel_circ_0011879	+	ENSG00000153767;	CVB5_5Y3
novel_circ_0011934	-	ENSG00000163848;	CVB5_5Y3
novel_circ_0011979	-	ENSG00000114656;	CVB5_5Y3
novel_circ_0012031	+	ENSG00000114054;	CVB5_5Y3
novel_circ_0012090	+	ENSG00000069849;	CVB5_5Y3
novel_circ_0012118	+	ENSG00000131379;	CVB5_5Y3
novel_circ_0012161	+	ENSG00000131375;	CVB5_5Y3
novel_circ_0012209	-	ENSG00000068885;ENSG0	CVB5_5Y3
novel_circ_0012333	-	ENSG00000043093;	CVB5_5Y3
novel_circ_0012427	-	ENSG00000114331;	CVB5_5Y3
novel_circ_0012439	-	ENSG00000286168;	CVB5_5Y3
novel_circ_0012586	+	ENSG00000153551;	CVB5_5Y3
novel_circ_0012991	-	ENSG00000172340;	CVB5_5Y3
novel_circ_0010029	-	ENSG00000115758;	CVB5_5Y3
novel_circ_0010095	+	ENSG00000188177;	CVB5_5Y3
novel_circ_0010263	+	ENSG00000080345;	CVB5_5Y3
novel_circ_0010272	-	ENSG00000151779;	CVB5_5Y3
novel_circ_0010284	-	ENSG00000196504;	CVB5_5Y3
novel_circ_0010568	+	ENSG00000196950;	CVB5_5Y3
novel_circ_0010612	-	ENSG00000115942;	CVB5_5Y3
novel_circ_0010664	-	ENSG00000055917;	CVB5_5Y3
novel_circ_0010672	+	ENSG00000138443;	CVB5_5Y3
novel_circ_0010801	+	n/a	CVB5_5Y3
novel_circ_0010844	+	ENSG00000168958;	CVB5_5Y3
novel_circ_0010859	-	ENSG00000035115;	CVB5_5Y3
novel_circ_0010860	-	ENSG00000163053;	CVB5_5Y3
novel_circ_0010870	+	ENSG00000135932;	CVB5_5Y3
novel_circ_0010957	-	ENSG00000130414;	CVB5_5Y3
novel_circ_0010994	-	ENSG00000198399;	CVB5_5Y3
novel_circ_0011036	-	ENSG00000138002;	CVB5_5Y3
novel_circ_0011614	+	ENSG00000124356;	CVB5_5Y3
novel_circ_0011623	-	ENSG00000114993;	CVB5_5Y3
novel_circ_0011670	-	ENSG00000172086;	CVB5_5Y3
novel_circ_0011671	-	ENSG00000134313;	CVB5_5Y3
novel_circ_0007209	+	ENSG00000116266;	CVB5_5Y3
novel_circ_0007221	-	ENSG00000134222;	CVB5_5Y3
novel_circ_0007226	+	n/a	CVB5_5Y3
novel_circ_0007488	-	ENSG00000160075;	CVB5_5Y3
novel_circ_0007540	+	ENSG00000143179;	CVB5_5Y3
novel_circ_0007636	+	ENSG00000152061;	CVB5_5Y3
novel_circ_0007662	-	ENSG00000008130;	CVB5_5Y3
novel_circ_0007801	-	ENSG00000118193;	CVB5_5Y3
novel_circ_0007843	+	ENSG00000198625;	CVB5_5Y3

novel_circ_0007845	-	ENSG00000133059;	CVB5_5Y3
novel_circ_0007882	-	ENSG00000196878;	CVB5_5Y3
novel_circ_0007912	+	ENSG00000162772;	CVB5_5Y3
novel_circ_0007938	+	ENSG00000067606;	CVB5_5Y3
novel_circ_0007969	-	ENSG00000196660;	CVB5_5Y3
novel_circ_0008126	+	ENSG00000270106;ENSG0	CVB5_5Y3
novel_circ_0008174	-	ENSG00000054267;	CVB5_5Y3
novel_circ_0008281	+	ENSG00000162852;	CVB5_5Y3
novel_circ_0008292	-	ENSG00000020633;	CVB5_5Y3
novel_circ_0008346	-	ENSG00000116350;	CVB5_5Y3
novel_circ_0008412	-	ENSG00000116525;	CVB5_5Y3
novel_circ_0008420	+	ENSG00000197056;	CVB5_5Y3
novel_circ_0008996	+	ENSG00000122483;	CVB5_5Y3
novel_circ_0017773	+	ENSG00000106701;	CVB5_5Y3
novel_circ_0017879	-	ENSG00000119402;	CVB5_5Y3
novel_circ_0017885	+	ENSG00000119397;	CVB5_5Y3
novel_circ_0017916	+	ENSG00000011454;	CVB5_5Y3
novel_circ_0018027	+	ENSG00000130723;	CVB5_5Y3
novel_circ_0018047	-	ENSG00000125485;	CVB5_5Y3
novel_circ_0018060	+	ENSG00000196363;	CVB5_5Y3
novel_circ_0018077	-	ENSG00000130560;	CVB5_5Y3
novel_circ_0018156	+	ENSG00000188352;	CVB5_5Y3
novel_circ_0018194	-	ENSG00000120159;	CVB5_5Y3
novel_circ_0016913	-	ENSG00000064313;	CVB5_5Y3
novel_circ_0016996	+	ENSG00000249859;	CVB5_5Y3
novel_circ_0017127	+	ENSG00000105339;	CVB5_5Y3
novel_circ_0017376	+	ENSG00000165102;	CVB5_5Y3
novel_circ_0017531	-	ENSG00000066777;	CVB5_5Y3
novel_circ_0017536	+	ENSG00000155189;	CVB5_5Y3
hsa_circ_0001931	-	ENSG00000085224;	CVB5_5Y3,Con_5Y1
hsa_circ_0028090	-	ENSG00000084112;	CVB5_5Y3,Con_5Y1
hsa_circ_0029531	-	ENSG00000177084;	CVB5_5Y3,Con_5Y1
hsa_circ_0025006	+	ENSG00000006831;	CVB5_5Y3,Con_5Y1
hsa_circ_0001964	+	ENSG00000029153;	CVB5_5Y3,Con_5Y1
hsa_circ_0020671	+	ENSG00000070047;	CVB5_5Y3,Con_5Y1
hsa_circ_0003417	+	ENSG00000141279;	CVB5_5Y3,Con_5Y1
hsa_circ_0004510	-	ENSG00000188603;ENSG0	CVB5_5Y3,Con_5Y1
hsa_circ_0006601	-	ENSG00000179151;	CVB5_5Y3,Con_5Y1
hsa_circ_0003333	+	ENSG00000140563;	CVB5_5Y3,Con_5Y1
hsa_circ_0005412	+	ENSG00000127616;	CVB5_5Y3,Con_5Y1
hsa_circ_0049375	+	ENSG00000127616;	CVB5_5Y3,Con_5Y1
hsa_circ_0004163	+	ENSG00000176890;	CVB5_5Y3,Con_5Y1
hsa_circ_0002799	+	ENSG00000130856;	CVB5_5Y3,Con_5Y1
hsa_circ_0006002	-	ENSG00000122490;	CVB5_5Y3,Con_5Y1
hsa_circ_0063623	+	ENSG00000198911;	CVB5_5Y3,Con_5Y1
hsa_circ_0063816	-	ENSG00000075275;	CVB5_5Y3,Con_5Y1
hsa_circ_0060522	+	ENSG00000101109;	CVB5_5Y3,Con_5Y1
hsa_circ_0061491	-	ENSG00000159086;	CVB5_5Y3,Con_5Y1
hsa_circ_0005592	+	ENSG00000284461;ENSG0	CVB5_5Y3,Con_5Y1
hsa_circ_0001726	-	ENSG00000198556;ENSG0	CVB5_5Y3,Con_5Y1
hsa_circ_0070236	-	ENSG00000138674;	CVB5_5Y3,Con_5Y1
hsa_circ_0064222	+	ENSG00000144554;	CVB5_5Y3,Con_5Y1
hsa_circ_0002580	+	ENSG00000174738;	CVB5_5Y3,Con_5Y1
hsa_circ_0064696	+	ENSG00000182973;	CVB5_5Y3,Con_5Y1
hsa_circ_0065394	-	ENSG00000114268;	CVB5_5Y3,Con_5Y1
hsa_circ_0004955	-	ENSG00000115170;	CVB5_5Y3,Con_5Y1
hsa_circ_0004575	+	ENSG00000116117;	CVB5_5Y3,Con_5Y1
hsa_circ_0003478	+	ENSG00000068724;	CVB5_5Y3,Con_5Y1

hsa_circ_0054861	-	ENSG00000082898;	CVB5_5Y3,Con_5Y1
hsa_circ_0016969	-	ENSG00000135749;	CVB5_5Y3,Con_5Y1
hsa_circ_0006164	-	ENSG00000043514;	CVB5_5Y3,Con_5Y1
hsa_circ_0089417	+	ENSG00000186350;	CVB5_5Y3,Con_5Y1
hsa_circ_0007816	+	ENSG00000147854;	CVB5_5Y3,Con_5Y1
hsa_circ_0004605	-	ENSG00000130958;ENSG0	CVB5_5Y3,Con_5Y1
novel_circ_0002034	+	ENSG00000174989;	CVB5_5Y3,Con_5Y1
novel_circ_0002317	+	ENSG00000111203;	CVB5_5Y3,Con_5Y1
novel_circ_0002659	-	ENSG00000133858;	CVB5_5Y3,Con_5Y1
novel_circ_0001122	+	ENSG00000167257;	CVB5_5Y3,Con_5Y1
novel_circ_0001705	-	ENSG00000130413;	CVB5_5Y3,Con_5Y1
novel_circ_0000269	-	ENSG00000182022;	CVB5_5Y3,Con_5Y1
novel_circ_0004935	-	ENSG00000159461;	CVB5_5Y3,Con_5Y1
novel_circ_0004965	+	ENSG00000067955;	CVB5_5Y3,Con_5Y1
novel_circ_0005187	+	ENSG00000158545;	CVB5_5Y3,Con_5Y1
novel_circ_0003992	-	ENSG00000128731;	CVB5_5Y3,Con_5Y1
novel_circ_0003461	-	ENSG00000092148;	CVB5_5Y3,Con_5Y1
novel_circ_0003547	-	ENSG00000100479;	CVB5_5Y3,Con_5Y1
novel_circ_0003791	+	ENSG00000119596;	CVB5_5Y3,Con_5Y1
novel_circ_0009768	-	ENSG00000180957;	CVB5_5Y3,Con_5Y1
novel_circ_0009933	-	ENSG00000100304;	CVB5_5Y3,Con_5Y1
novel_circ_0009948	+	ENSG00000100376;	CVB5_5Y3,Con_5Y1
novel_circ_0009268	+	ENSG00000088888;	CVB5_5Y3,Con_5Y1
novel_circ_0016046	-	ENSG00000146858;	CVB5_5Y3,Con_5Y1
novel_circ_0015121	+	ENSG00000124523;	CVB5_5Y3,Con_5Y1
novel_circ_0015446	-	ENSG00000213676;	CVB5_5Y3,Con_5Y1
novel_circ_0014067	+	ENSG00000151292;	CVB5_5Y3,Con_5Y1
novel_circ_0014183	-	ENSG00000031003;	CVB5_5Y3,Con_5Y1
novel_circ_0014677	+	ENSG00000123213;	CVB5_5Y3,Con_5Y1
novel_circ_0014899	+	ENSG00000164292;	CVB5_5Y3,Con_5Y1
novel_circ_0012127	-	ENSG00000018408;	CVB5_5Y3,Con_5Y1
novel_circ_0012492	+	ENSG00000122068;	CVB5_5Y3,Con_5Y1
novel_circ_0011071	+	ENSG00000163811;	CVB5_5Y3,Con_5Y1
novel_circ_0011117	+	ENSG00000115760;	CVB5_5Y3,Con_5Y1
novel_circ_0007427	-	ENSG00000116539;	CVB5_5Y3,Con_5Y1
novel_circ_0007799	+	ENSG00000169914;	CVB5_5Y3,Con_5Y1
novel_circ_0007864	-	ENSG00000127483;	CVB5_5Y3,Con_5Y1
novel_circ_0018340	+	ENSG00000120158;	CVB5_5Y3,Con_5Y1
novel_circ_0017629	+	ENSG00000147606;	CVB5_5Y3,Con_5Y1
novel_circ_0017710	+	ENSG00000104356;	CVB5_5Y3,Con_5Y1
hsa_circ_0004851	+	ENSG00000135387;	CVB5_5Y3,Con_5Y1,
hsa_circ_0000349	+	ENSG00000042429;ENSG0	CVB5_5Y3,Con_5Y1,
hsa_circ_0008456	-	ENSG00000189319;ENSG0	CVB5_5Y3,Con_5Y1,
hsa_circ_0043184	+	ENSG00000278311;	CVB5_5Y3,Con_5Y1,
hsa_circ_0005287	+	ENSG00000189149;	CVB5_5Y3,Con_5Y1,
hsa_circ_0039783	+	ENSG00000067955;	CVB5_5Y3,Con_5Y1,
hsa_circ_0040264	+	ENSG00000189091;	CVB5_5Y3,Con_5Y1,
hsa_circ_0004172	-	ENSG00000168411;	CVB5_5Y3,Con_5Y1,
hsa_circ_0006945	+	ENSG00000158805;	CVB5_5Y3,Con_5Y1,
hsa_circ_0004137	-	ENSG00000100461;	CVB5_5Y3,Con_5Y1,
hsa_circ_0031431	+	ENSG00000100473;	CVB5_5Y3,Con_5Y1,
hsa_circ_0003305	+	ENSG00000100731;	CVB5_5Y3,Con_5Y1,
hsa_circ_0002143	-	ENSG00000100596;	CVB5_5Y3,Con_5Y1,
hsa_circ_0059481	-	ENSG00000089177;	CVB5_5Y3,Con_5Y1,
hsa_circ_0079294	-	ENSG00000011275;	CVB5_5Y3,Con_5Y1,
hsa_circ_0007281	-	ENSG00000136240;	CVB5_5Y3,Con_5Y1,
hsa_circ_0076254	-	ENSG00000183826;	CVB5_5Y3,Con_5Y1,
hsa_circ_0005949	-	ENSG00000168916;	CVB5_5Y3,Con_5Y1,

hsa_circ_0008089	-	ENSG00000164253;	CVB5_5Y3,Con_5Y1,
hsa_circ_0071197	-	ENSG00000198589;	CVB5_5Y3,Con_5Y1,
hsa_circ_0068958	-	ENSG00000087269;	CVB5_5Y3,Con_5Y1,
hsa_circ_0002962	-	ENSG00000163812;	CVB5_5Y3,Con_5Y1,
hsa_circ_0055514	-	ENSG00000132305;	CVB5_5Y3,Con_5Y1,
hsa_circ_0008936	-	ENSG00000160049;	CVB5_5Y3,Con_5Y1,
hsa_circ_0004717	+	ENSG00000143569;	CVB5_5Y3,Con_5Y1,
hsa_circ_0002898	+	ENSG00000116138;	CVB5_5Y3,Con_5Y1,
hsa_circ_0000137	-	ENSG00000116539;	CVB5_5Y3,Con_5Y1,
hsa_circ_0011588	-	ENSG00000092853;	CVB5_5Y3,Con_5Y1,
hsa_circ_0012545	-	ENSG00000134744;	CVB5_5Y3,Con_5Y1,
hsa_circ_0087564	+	ENSG00000175787;	CVB5_5Y3,Con_5Y1,
novel_circ_0002760	-	ENSG00000173588;	CVB5_5Y3,Con_5Y1,
novel_circ_0000098	-	ENSG00000119953;	CVB5_5Y3,Con_5Y1,
novel_circ_0005019	-	ENSG00000102908;	CVB5_5Y3,Con_5Y1,
novel_circ_0005235	+	ENSG00000141002;	CVB5_5Y3,Con_5Y1,
novel_circ_0003994	-	ENSG00000104067;	CVB5_5Y3,Con_5Y1,
novel_circ_0004466	+	ENSG00000140403;	CVB5_5Y3,Con_5Y1,
novel_circ_0003751	+	ENSG00000197555;	CVB5_5Y3,Con_5Y1,
novel_circ_0007064	-	ENSG00000161551;	CVB5_5Y3,Con_5Y1,
novel_circ_0011993	-	ENSG00000172765;	CVB5_5Y3,Con_5Y1,
novel_circ_0012053	-	ENSG00000118007;	CVB5_5Y3,Con_5Y1,
novel_circ_0011666	-	ENSG00000115561;	CVB5_5Y3,Con_5Y1,
novel_circ_0007406	-	ENSG00000143614;	CVB5_5Y3,Con_5Y1,
novel_circ_0008932	+	ENSG00000097033;	CVB5_5Y3,Con_5Y1,
novel_circ_0018215	+	ENSG00000086061;	CVB5_5Y3,Con_5Y1,
hsa_circ_0008399	+	ENSG00000102317;	CVB5_5Y3,Con_5Y1,
hsa_circ_0003040	+	ENSG00000182957;	CVB5_5Y3,Con_5Y1,
hsa_circ_0030704	-	ENSG00000102572;	CVB5_5Y3,Con_5Y1,
hsa_circ_0002881	-	ENSG00000073614;	CVB5_5Y3,Con_5Y1,
hsa_circ_0024402	+	ENSG00000167257;	CVB5_5Y3,Con_5Y1,
hsa_circ_0000339	-	ENSG00000175582;	CVB5_5Y3,Con_5Y1,
hsa_circ_0007242	+	ENSG00000123240;	CVB5_5Y3,Con_5Y1,
hsa_circ_0003508	-	ENSG00000186566;	CVB5_5Y3,Con_5Y1,
hsa_circ_0008284	+	ENSG00000103319;	CVB5_5Y3,Con_5Y1,
hsa_circ_0005217	+	ENSG00000197912;	CVB5_5Y3,Con_5Y1,
hsa_circ_0000657	+	ENSG00000140563;	CVB5_5Y3,Con_5Y1,
hsa_circ_0049398	+	ENSG00000130164;	CVB5_5Y3,Con_5Y1,
hsa_circ_0083012	-	ENSG00000055609;	CVB5_5Y3,Con_5Y1,
hsa_circ_0079492	+	ENSG00000136261;	CVB5_5Y3,Con_5Y1,
hsa_circ_0077717	+	ENSG00000164465;	CVB5_5Y3,Con_5Y1,
hsa_circ_0005820	-	ENSG00000135316;	CVB5_5Y3,Con_5Y1,
hsa_circ_0002449	+	ENSG00000120306;	CVB5_5Y3,Con_5Y1,
hsa_circ_0008839	+	ENSG00000114354;	CVB5_5Y3,Con_5Y1,
hsa_circ_0007348	+	ENSG00000114098;	CVB5_5Y3,Con_5Y1,
hsa_circ_0064555	-	ENSG00000182568;	CVB5_5Y3,Con_5Y1,
hsa_circ_0002912	+	ENSG00000163808;	CVB5_5Y3,Con_5Y1,
hsa_circ_0057173	+	ENSG00000018510;	CVB5_5Y3,Con_5Y1,
hsa_circ_0004164	-	ENSG00000176946;	CVB5_5Y3,Con_5Y1,
hsa_circ_0007608	-	ENSG00000116539;	CVB5_5Y3,Con_5Y1,
hsa_circ_0016115	+	ENSG00000058673;ENSG0	CVB5_5Y3,Con_5Y1,
hsa_circ_0011572	-	ENSG00000142687;	CVB5_5Y3,Con_5Y1,
hsa_circ_0005246	-	ENSG00000117419;	CVB5_5Y3,Con_5Y1,
hsa_circ_0089392	-	ENSG00000160293;	CVB5_5Y3,Con_5Y1,
hsa_circ_0008678	+	ENSG00000188352;	CVB5_5Y3,Con_5Y1,
hsa_circ_0086242	-	ENSG00000080298;	CVB5_5Y3,Con_5Y1,
hsa_circ_0006728	+	ENSG00000086102;	CVB5_5Y3,Con_5Y1,
hsa_circ_0087255	+	ENSG00000197969;	CVB5_5Y3,Con_5Y1,

hsa_circ_0006586	+	ENSG00000168522;ENSG0	CVB5_5Y3,Con_5Y1,
novel_circ_0002936	-	ENSG00000151849;	CVB5_5Y3,Con_5Y1,
novel_circ_0006041	+	ENSG00000181222;	CVB5_5Y3,Con_5Y1,
novel_circ_0006615	+	ENSG00000168502;	CVB5_5Y3,Con_5Y1,
novel_circ_0009247	-	ENSG00000080839;	CVB5_5Y3,Con_5Y1,
novel_circ_0015597	+	n/a	CVB5_5Y3,Con_5Y1,
novel_circ_0012045	-	ENSG00000118007;	CVB5_5Y3,Con_5Y1,
novel_circ_0012581	+	ENSG00000153551;	CVB5_5Y3,Con_5Y1,
novel_circ_0008423	-	ENSG00000116560;	CVB5_5Y3,Con_5Y1,
hsa_circ_0029693	-	ENSG00000150457;	CVB5_5Y3,Con_5Y1,
hsa_circ_0051450	+	ENSG00000007047;	CVB5_5Y3,Con_5Y1,
hsa_circ_0069320	-	ENSG00000152990;	CVB5_5Y3,Con_5Y1,
hsa_circ_0069795	-	ENSG00000134852;	CVB5_5Y3,Con_5Y1,
hsa_circ_0070113	+	ENSG00000138759;	CVB5_5Y3,Con_5Y1,
hsa_circ_0004086	-	ENSG00000173905;	CVB5_5Y3,Con_5Y1,
hsa_circ_0065635	-	ENSG00000114316;	CVB5_5Y3,Con_5Y1,
hsa_circ_0057657	+	ENSG00000115520;	CVB5_5Y3,Con_5Y1,
hsa_circ_0005853	-	ENSG00000286239;ENSG0	CVB5_5Y3,Con_5Y1,
hsa_circ_0007439	+	ENSG00000213639;ENSG0	CVB5_5Y3,Con_5Y1,
hsa_circ_0015207	+	ENSG00000117523;	CVB5_5Y3,Con_5Y1,
hsa_circ_0013084	+	ENSG00000097033;	CVB5_5Y3,Con_5Y1,
hsa_circ_0089197	+	ENSG00000130723;	CVB5_5Y3,Con_5Y1,
novel_circ_0002068	+	ENSG00000022840;	CVB5_5Y3,Con_5Y1,
novel_circ_0001428	-	ENSG00000175216;	CVB5_5Y3,Con_5Y1,
novel_circ_0005703	+	ENSG00000159202;	CVB5_5Y3,Con_5Y1,
novel_circ_0009118	+	ENSG00000101003;	CVB5_5Y3,Con_5Y1,
novel_circ_0014717	+	ENSG00000145734;	CVB5_5Y3,Con_5Y1,
novel_circ_0013166	-	ENSG00000159692;	CVB5_5Y3,Con_5Y1,
novel_circ_0013363	-	ENSG00000109670;	CVB5_5Y3,Con_5Y1,
novel_circ_0010673	+	ENSG00000138443;	CVB5_5Y3,Con_5Y1,
novel_circ_0010771	+	n/a	CVB5_5Y3,Con_5Y1,
novel_circ_0011539	-	ENSG00000273398;ENSG0	CVB5_5Y3,Con_5Y1,
novel_circ_0008962	+	ENSG00000189195;	CVB5_5Y3,Con_5Y1,
hsa_circ_0091459	+	ENSG00000101972;	CVB5_5Y3,Con_5Y2
hsa_circ_0003587	-	ENSG00000102781;	CVB5_5Y3,Con_5Y2
hsa_circ_0007007	-	ENSG00000110880;	CVB5_5Y3,Con_5Y2
hsa_circ_0028088	-	ENSG00000084112;	CVB5_5Y3,Con_5Y2
hsa_circ_0005916	+	ENSG00000174989;	CVB5_5Y3,Con_5Y2
hsa_circ_0025836	+	ENSG00000174718;	CVB5_5Y3,Con_5Y2
hsa_circ_0024271	-	ENSG00000137713;	CVB5_5Y3,Con_5Y2
hsa_circ_0003307	+	ENSG00000177156;	CVB5_5Y3,Con_5Y2
hsa_circ_0019664	-	ENSG00000198728;	CVB5_5Y3,Con_5Y2
hsa_circ_0000268	+	ENSG00000019995;	CVB5_5Y3,Con_5Y2
hsa_circ_0017856	-	ENSG00000148484;	CVB5_5Y3,Con_5Y2
hsa_circ_0017510	-	ENSG00000107959;	CVB5_5Y3,Con_5Y2
hsa_circ_0002758	+	ENSG00000099282;	CVB5_5Y3,Con_5Y2
hsa_circ_0005565	+	ENSG00000010244;	CVB5_5Y3,Con_5Y2
hsa_circ_0043278	+	ENSG00000276234;	CVB5_5Y3,Con_5Y2
hsa_circ_0043954	-	ENSG00000012048;	CVB5_5Y3,Con_5Y2
hsa_circ_0004796	+	ENSG00000108424;	CVB5_5Y3,Con_5Y2
hsa_circ_0045310	+	ENSG00000258890;	CVB5_5Y3,Con_5Y2
hsa_circ_0045714	+	ENSG00000132478;	CVB5_5Y3,Con_5Y2
hsa_circ_0000709	+	ENSG00000102974;	CVB5_5Y3,Con_5Y2
hsa_circ_0000714	-	ENSG00000090861;	CVB5_5Y3,Con_5Y2
hsa_circ_0005417	-	ENSG00000128881;	CVB5_5Y3,Con_5Y2
hsa_circ_0035873	-	ENSG00000090487;	CVB5_5Y3,Con_5Y2
hsa_circ_0033614	+	ENSG00000182979;	CVB5_5Y3,Con_5Y2
hsa_circ_0002012	+	ENSG00000020577;	CVB5_5Y3,Con_5Y2

hsa_circ_0049329	+	ENSG00000079805;	CVB5_5Y3,Con_5Y2
hsa_circ_0005660	+	ENSG00000008441;	CVB5_5Y3,Con_5Y2
hsa_circ_0008299	-	ENSG00000186111;	CVB5_5Y3,Con_5Y2
hsa_circ_0002403	+	ENSG00000168234;	CVB5_5Y3,Con_5Y2
hsa_circ_0063300	-	ENSG00000283900;ENSG0	CVB5_5Y3,Con_5Y2
hsa_circ_0063408	+	ENSG00000100354;	CVB5_5Y3,Con_5Y2
hsa_circ_0081839	-	ENSG00000091127;	CVB5_5Y3,Con_5Y2
hsa_circ_0081872	-	ENSG00000008282;	CVB5_5Y3,Con_5Y2
hsa_circ_0082333	+	ENSG00000128607;	CVB5_5Y3,Con_5Y2
hsa_circ_0080849	+	ENSG00000127947;	CVB5_5Y3,Con_5Y2
hsa_circ_0006554	-	ENSG00000197442;	CVB5_5Y3,Con_5Y2
hsa_circ_0077276	-	ENSG00000146282;	CVB5_5Y3,Con_5Y2
hsa_circ_0073761	+	ENSG00000064651;	CVB5_5Y3,Con_5Y2
hsa_circ_0003989	+	ENSG00000038382;	CVB5_5Y3,Con_5Y2
hsa_circ_0003227	-	ENSG00000113569;	CVB5_5Y3,Con_5Y2
hsa_circ_0008243	+	ENSG00000071205;	CVB5_5Y3,Con_5Y2
hsa_circ_0058763	+	ENSG00000077044;	CVB5_5Y3,Con_5Y2
hsa_circ_0000115	-	ENSG00000009307;	CVB5_5Y3,Con_5Y2
hsa_circ_0006575	-	ENSG00000143622;	CVB5_5Y3,Con_5Y2
hsa_circ_0005357	-	ENSG00000143207;	CVB5_5Y3,Con_5Y2
hsa_circ_0005356	+	ENSG00000176393;	CVB5_5Y3,Con_5Y2
hsa_circ_0016519	+	ENSG00000116141;	CVB5_5Y3,Con_5Y2
hsa_circ_0011174	+	ENSG00000159023;	CVB5_5Y3,Con_5Y2
hsa_circ_0088153	+	ENSG00000138835;	CVB5_5Y3,Con_5Y2
hsa_circ_0003782	+	ENSG00000188352;	CVB5_5Y3,Con_5Y2
hsa_circ_0004982	-	ENSG00000155096;	CVB5_5Y3,Con_5Y2
hsa_circ_0004759	-	ENSG00000147687;	CVB5_5Y3,Con_5Y2
hsa_circ_0083905	-	ENSG00000172728;	CVB5_5Y3,Con_5Y2
hsa_circ_0083964	+	ENSG00000129691;	CVB5_5Y3,Con_5Y2
hsa_circ_0001802	-	ENSG00000168300;	CVB5_5Y3,Con_5Y2
hsa_circ_0008736	-	ENSG00000104714;	CVB5_5Y3,Con_5Y2
hsa_circ_0084974	+	ENSG00000156471;	CVB5_5Y3,Con_5Y2
novel_circ_0002856	+	ENSG00000130177;	CVB5_5Y3,Con_5Y2
novel_circ_0001887	+	ENSG00000082805;	CVB5_5Y3,Con_5Y2
novel_circ_0002634	+	n/a	CVB5_5Y3,Con_5Y2
novel_circ_0001322	+	ENSG00000060749;	CVB5_5Y3,Con_5Y2
novel_circ_0001469	+	ENSG00000070047;	CVB5_5Y3,Con_5Y2
novel_circ_0000265	+	ENSG00000151465;	CVB5_5Y3,Con_5Y2
novel_circ_0005838	+	ENSG00000062716;	CVB5_5Y3,Con_5Y2
novel_circ_0006052	+	ENSG00000073350;	CVB5_5Y3,Con_5Y2
novel_circ_0005090	-	ENSG00000090863;	CVB5_5Y3,Con_5Y2
novel_circ_0005118	-	ENSG00000065457;	CVB5_5Y3,Con_5Y2
novel_circ_0003768	+	ENSG00000080815;	CVB5_5Y3,Con_5Y2
novel_circ_0006909	+	ENSG00000105738;	CVB5_5Y3,Con_5Y2
novel_circ_0007047	+	ENSG00000104960;	CVB5_5Y3,Con_5Y2
novel_circ_0006385	+	ENSG00000101596;	CVB5_5Y3,Con_5Y2
novel_circ_0009981	+	ENSG00000054611;	CVB5_5Y3,Con_5Y2
novel_circ_0009409	-	ENSG00000019186;	CVB5_5Y3,Con_5Y2
novel_circ_0009637	+	ENSG00000182240;	CVB5_5Y3,Con_5Y2
novel_circ_0015956	-	ENSG00000081803;	CVB5_5Y3,Con_5Y2
novel_circ_0015190	-	ENSG00000131023;	CVB5_5Y3,Con_5Y2
novel_circ_0015490	-	ENSG00000112079;	CVB5_5Y3,Con_5Y2
novel_circ_0015712	-	ENSG00000118420;	CVB5_5Y3,Con_5Y2
novel_circ_0014057	+	ENSG00000151304;	CVB5_5Y3,Con_5Y2
novel_circ_0014241	+	ENSG00000131507;	CVB5_5Y3,Con_5Y2
novel_circ_0014358	+	ENSG00000113312;	CVB5_5Y3,Con_5Y2
novel_circ_0014450	+	ENSG00000161021;	CVB5_5Y3,Con_5Y2
novel_circ_0014473	+	n/a	CVB5_5Y3,Con_5Y2

novel_circ_0014505	+	ENSG00000113460;	CVB5_5Y3,Con_5Y2
novel_circ_0012176	-	ENSG00000169359;	CVB5_5Y3,Con_5Y2
novel_circ_0012382	+	ENSG00000073803;	CVB5_5Y3,Con_5Y2
novel_circ_0012575	+	ENSG00000152642;	CVB5_5Y3,Con_5Y2
novel_circ_0012579	+	ENSG00000170293;	CVB5_5Y3,Con_5Y2
novel_circ_0012707	+	ENSG00000163808;	CVB5_5Y3,Con_5Y2
novel_circ_0010241	-	ENSG00000121964;	CVB5_5Y3,Con_5Y2
novel_circ_0010267	+	ENSG00000080345;	CVB5_5Y3,Con_5Y2
novel_circ_0010452	+	ENSG00000144354;	CVB5_5Y3,Con_5Y2
novel_circ_0007324	+	ENSG00000116731;	CVB5_5Y3,Con_5Y2
novel_circ_0007521	+	ENSG00000117143;	CVB5_5Y3,Con_5Y2
novel_circ_0008287	-	ENSG00000196418;	CVB5_5Y3,Con_5Y2
novel_circ_0008819	-	ENSG00000118454;	CVB5_5Y3,Con_5Y2
novel_circ_0017815	+	ENSG00000188959;	CVB5_5Y3,Con_5Y2
novel_circ_0018076	-	ENSG00000130559;	CVB5_5Y3,Con_5Y2
novel_circ_0017147	-	ENSG00000261236;	CVB5_5Y3,Con_5Y2
novel_circ_0017415	-	ENSG00000253729;	CVB5_5Y3,Con_5Y2
novel_circ_0017446	+	ENSG00000137574;	CVB5_5Y3,Con_5Y2
novel_circ_0017523	+	ENSG00000104218;	CVB5_5Y3,Con_5Y2
hsa_circ_0008930	-	ENSG00000147099;ENSG0	CVB5_5Y3,Con_5Y2,
hsa_circ_0030771	+	ENSG00000175198;	CVB5_5Y3,Con_5Y2,
hsa_circ_0002009	+	ENSG00000111203;	CVB5_5Y3,Con_5Y2,
hsa_circ_0027731	-	ENSG00000057704;	CVB5_5Y3,Con_5Y2,
hsa_circ_0037527	+	ENSG00000162063;	CVB5_5Y3,Con_5Y2,
hsa_circ_0040487	-	ENSG00000168411;	CVB5_5Y3,Con_5Y2,
hsa_circ_0040816	+	ENSG00000172530;	CVB5_5Y3,Con_5Y2,
hsa_circ_0000617	-	ENSG00000074603;	CVB5_5Y3,Con_5Y2,
hsa_circ_0005261	-	ENSG00000105486;	CVB5_5Y3,Con_5Y2,
hsa_circ_0082689	-	ENSG00000059378;	CVB5_5Y3,Con_5Y2,
hsa_circ_0006010	-	ENSG00000229358;	CVB5_5Y3,Con_5Y2,
hsa_circ_0079363	-	ENSG00000164535;	CVB5_5Y3,Con_5Y2,
hsa_circ_0004074	+	n/a	CVB5_5Y3,Con_5Y2,
hsa_circ_0008975	+	ENSG00000071205;	CVB5_5Y3,Con_5Y2,
hsa_circ_0056840	+	ENSG00000136536;	CVB5_5Y3,Con_5Y2,
hsa_circ_0005899	-	ENSG00000143669;	CVB5_5Y3,Con_5Y2,
hsa_circ_0011571	-	ENSG00000142687;	CVB5_5Y3,Con_5Y2,
hsa_circ_0005684	+	ENSG00000137145;	CVB5_5Y3,Con_5Y2,
hsa_circ_0085189	-	ENSG00000104517;	CVB5_5Y3,Con_5Y2,
hsa_circ_0085760	-	ENSG00000169398;	CVB5_5Y3,Con_5Y2,
hsa_circ_0002946	+	ENSG00000147649;	CVB5_5Y3,Con_5Y2,
novel_circ_0000447	-	ENSG00000107890;	CVB5_5Y3,Con_5Y2,
novel_circ_0000788	-	ENSG00000166348;	CVB5_5Y3,Con_5Y2,
novel_circ_0004799	-	n/a	CVB5_5Y3,Con_5Y2,
novel_circ_0004464	+	ENSG00000140403;	CVB5_5Y3,Con_5Y2,
novel_circ_0003870	-	ENSG00000100784;	CVB5_5Y3,Con_5Y2,
novel_circ_0003883	-	ENSG00000100815;	CVB5_5Y3,Con_5Y2,
novel_circ_0006714	+	ENSG00000132003;	CVB5_5Y3,Con_5Y2,
novel_circ_0006330	+	ENSG00000101773;	CVB5_5Y3,Con_5Y2,
novel_circ_0009834	-	ENSG00000233080;	CVB5_5Y3,Con_5Y2,
novel_circ_0009373	-	ENSG00000124214;	CVB5_5Y3,Con_5Y2,
novel_circ_0015603	+	ENSG00000112200;	CVB5_5Y3,Con_5Y2,
novel_circ_0015623	+	ENSG00000118482;	CVB5_5Y3,Con_5Y2,
hsa_circ_0004438	-	ENSG00000150990;	CVB5_5Y3,Con_5Y3
hsa_circ_0025039	-	ENSG00000111206;	CVB5_5Y3,Con_5Y3
hsa_circ_0002886	+	ENSG00000179104;	CVB5_5Y3,Con_5Y3
hsa_circ_0020919	-	ENSG00000110713;	CVB5_5Y3,Con_5Y3
hsa_circ_0022512	-	ENSG00000234857;ENSG0	CVB5_5Y3,Con_5Y3
hsa_circ_0007583	+	ENSG00000158636;	CVB5_5Y3,Con_5Y3

hsa_circ_0004792	-	ENSG00000107581;	CVB5_5Y3,Con_5Y3
hsa_circ_0008851	+	ENSG00000095794;	CVB5_5Y3,Con_5Y3
hsa_circ_0000787	-	ENSG00000121060;	CVB5_5Y3,Con_5Y3
hsa_circ_0008052	+	ENSG00000047578;	CVB5_5Y3,Con_5Y3
hsa_circ_0037710	+	ENSG00000103423;	CVB5_5Y3,Con_5Y3
hsa_circ_0008471	+	ENSG00000156970;	CVB5_5Y3,Con_5Y3
hsa_circ_0051106	+	ENSG00000160410;	CVB5_5Y3,Con_5Y3
hsa_circ_0048782	-	ENSG00000196365;	CVB5_5Y3,Con_5Y3
hsa_circ_0047596	-	ENSG00000078043;	CVB5_5Y3,Con_5Y3
hsa_circ_0006917	-	ENSG00000101391;	CVB5_5Y3,Con_5Y3
hsa_circ_0060193	+	ENSG00000101084;ENSG0	CVB5_5Y3,Con_5Y3
hsa_circ_0062090	+	ENSG00000160299;	CVB5_5Y3,Con_5Y3
hsa_circ_0079039	-	ENSG00000188191;	CVB5_5Y3,Con_5Y3
hsa_circ_0005145	+	ENSG00000154124;	CVB5_5Y3,Con_5Y3
hsa_circ_0074719	+	ENSG00000155508;	CVB5_5Y3,Con_5Y3
hsa_circ_0070573	-	ENSG00000138785;	CVB5_5Y3,Con_5Y3
hsa_circ_0005298	+	ENSG00000281028;ENSG0	CVB5_5Y3,Con_5Y3
hsa_circ_0006908	+	ENSG00000115295;	CVB5_5Y3,Con_5Y3
hsa_circ_0006583	+	ENSG00000081026;	CVB5_5Y3,Con_5Y3
hsa_circ_0008815	-	ENSG00000134744;	CVB5_5Y3,Con_5Y3
hsa_circ_0006519	-	ENSG00000198363;	CVB5_5Y3,Con_5Y3
novel_circ_0018714	-	ENSG00000129680;	CVB5_5Y3,Con_5Y3
novel_circ_0001919	-	ENSG00000231887;ENSG0	CVB5_5Y3,Con_5Y3
novel_circ_0002488	+	ENSG00000123268;	CVB5_5Y3,Con_5Y3
novel_circ_0001147	+	ENSG00000110395;	CVB5_5Y3,Con_5Y3
novel_circ_0000193	-	ENSG00000107581;	CVB5_5Y3,Con_5Y3
novel_circ_0000440	-	ENSG00000136754;	CVB5_5Y3,Con_5Y3
novel_circ_0000726	+	ENSG00000060339;	CVB5_5Y3,Con_5Y3
novel_circ_0005295	-	ENSG00000141027;	CVB5_5Y3,Con_5Y3
novel_circ_0005746	-	ENSG00000011258;	CVB5_5Y3,Con_5Y3
novel_circ_0003938	-	ENSG00000140470;	CVB5_5Y3,Con_5Y3
novel_circ_0004022	-	ENSG00000021776;	CVB5_5Y3,Con_5Y3
novel_circ_0003543	-	ENSG00000100479;	CVB5_5Y3,Con_5Y3
novel_circ_0003925	-	ENSG00000127152;	CVB5_5Y3,Con_5Y3
novel_circ_0007033	+	ENSG00000177380;	CVB5_5Y3,Con_5Y3
novel_circ_0015416	-	ENSG00000021355;	CVB5_5Y3,Con_5Y3
novel_circ_0015481	-	ENSG00000096063;	CVB5_5Y3,Con_5Y3
novel_circ_0015535	-	ENSG00000124571;	CVB5_5Y3,Con_5Y3
novel_circ_0012568	+	n/a	CVB5_5Y3,Con_5Y3
novel_circ_0012617	-	ENSG00000153560;	CVB5_5Y3,Con_5Y3
novel_circ_0012686	+	ENSG00000114812;	CVB5_5Y3,Con_5Y3
novel_circ_0010117	+	ENSG00000125629;	CVB5_5Y3,Con_5Y3
novel_circ_0011636	+	ENSG00000176407;	CVB5_5Y3,Con_5Y3
novel_circ_0008936	+	ENSG00000153936;ENSG0	CVB5_5Y3,Con_5Y3
novel_circ_0018332	+	ENSG00000120158;	CVB5_5Y3,Con_5Y3
novel_circ_0018454	+	ENSG00000135040;	CVB5_5Y3,Con_5Y3
novel_circ_0016991	-	ENSG00000254166;	CVB5_5Y3,Con_5Y3

on_5Y3
on_5Y3
on_5Y3
on_5Y3
on_5Y3
on_5Y3
on_5Y3
on_5Y3
on_5Y3
on_5Y3
on_5Y3
on_5Y3
on_5Y3
on_5Y3
on_5Y3
on_5Y3
on_5Y3
on_5Y3
on_5Y3
on_5Y3
on_5Y3
on_5Y3
on_5Y3
on_5Y3
on_5Y3
on_5Y3
on_5Y3
on_5Y3
on_5Y3
on_5Y3
on_5Y3
on_5Y3
on_5Y3
on_5Y3
on_5Y3
on_5Y3
on_5Y3
on_5Y3
on_5Y3
on_5Y3
on_5Y3
on_5Y3
on_5Y3
on_5Y3
on_5Y3
on_5Y3
on_5Y3
on_5Y3
on_5Y3
on_5Y3
on_5Y3
on_5Y3
on_5Y3
on_5Y3
on_5Y3

,Con_5Y2
,Con_5Y2
,Con_5Y2
,Con_5Y2
,Con_5Y2
,Con_5Y2
,Con_5Y2
,Con_5Y2
,Con_5Y2
,Con_5Y2
,Con_5Y2
,Con_5Y2
,Con_5Y2
,Con_5Y2
,Con_5Y2
,Con_5Y2
,Con_5Y2
,Con_5Y2
,Con_5Y2
,Con_5Y2
,Con_5Y2
,Con_5Y2
,Con_5Y2
,Con_5Y2
,Con_5Y2
,Con_5Y2
,Con_5Y2
,Con_5Y2
,Con_5Y2

,Con_5Y2
,Con_5Y2
,Con_5Y2,Con_5Y3
,Con_5Y2,Con_5Y3
,Con_5Y2,Con_5Y3
,Con_5Y2,Con_5Y3
,Con_5Y2,Con_5Y3
,Con_5Y2,Con_5Y3
,Con_5Y2,Con_5Y3
,Con_5Y2,Con_5Y3
,Con_5Y2,Con_5Y3
,Con_5Y2,Con_5Y3
,Con_5Y2,Con_5Y3
,Con_5Y2,Con_5Y3
,Con_5Y2,Con_5Y3
,Con_5Y2,Con_5Y3
,Con_5Y2,Con_5Y3
,Con_5Y2,Con_5Y3
,Con_5Y2,Con_5Y3
,Con_5Y2,Con_5Y3
,Con_5Y2,Con_5Y3
,Con_5Y2,Con_5Y3
,Con_5Y2,Con_5Y3
,Con_5Y2,Con_5Y3
,Con_5Y2,Con_5Y3
,Con_5Y2,Con_5Y3
,Con_5Y2,Con_5Y3
,Con_5Y2,Con_5Y3
,Con_5Y2,Con_5Y3
,Con_5Y2,Con_5Y3
,Con_5Y2,Con_5Y3
,Con_5Y2,Con_5Y3
,Con_5Y2,Con_5Y3
,Con_5Y2,Con_5Y3
,Con_5Y2,Con_5Y3
,Con_5Y2,Con_5Y3
,Con_5Y2,Con_5Y3
,Con_5Y2,Con_5Y3
,Con_5Y2,Con_5Y3
,Con_5Y2,Con_5Y3
,Con_5Y2,Con_5Y3
,Con_5Y2,Con_5Y3
,Con_5Y2,Con_5Y3
,Con_5Y2,Con_5Y3
,Con_5Y2,Con_5Y3
,Con_5Y3
,Con_5Y3
,Con_5Y3
,Con_5Y3
,Con_5Y3
,Con_5Y3
,Con_5Y3
,Con_5Y3
,Con_5Y3
,Con_5Y3
,Con_5Y3
,Con_5Y3
,Con_5Y3

,Con_5Y3
,Con_5Y3
,Con_5Y3
,Con_5Y3
,Con_5Y3
,Con_5Y3
,Con_5Y3
,Con_5Y3

,Con_5Y3
,Con_5Y3
,Con_5Y3
,Con_5Y3
,Con_5Y3
,Con_5Y3
,Con_5Y3
,Con_5Y3
,Con_5Y3
,Con_5Y3
,Con_5Y3
,Con_5Y3
,Con_5Y3
,Con_5Y3
,Con_5Y3
,Con_5Y3
,Con_5Y3

,Con_5Y3
,Con_5Y3
,Con_5Y3
,Con_5Y3
,Con_5Y3
,Con_5Y3
,Con_5Y3
,Con_5Y3
,Con_5Y3
,Con_5Y3
,Con_5Y3
,Con_5Y3
,Con_5Y3
,Con_5Y3
,Con_5Y3
,Con_5Y3
,Con_5Y3

/2
/2
/2
/2

Y2
Y2
Y2
Y2
Y2
Y2
Y2
Y2
Y2
Y2
Y2
Y2
Y2
Y2
Y2
Y2
Y2
Y2
Y2
Y2
Y2
Y2
Y2
Y2
Y2
Y2
Y2
Y2
Y2,Con_5Y1
Y2,Con_5Y1
Y2,Con_5Y1
Y2,Con_5Y1
Y2,Con_5Y1
Y2,Con_5Y1
Y2,Con_5Y1
Y2,Con_5Y1
Y2,Con_5Y1
Y2,Con_5Y1
Y2,Con_5Y1
Y2,Con_5Y1
Y2,Con_5Y1
Y2,Con_5Y1
Y2,Con_5Y1
Y2,Con_5Y1
Y2,Con_5Y1
Y2,Con_5Y1
Y2,Con_5Y1
Y2,Con_5Y1,Con_5Y2
Y2,Con_5Y1,Con_5Y2
Y2,Con_5Y1,Con_5Y2
Y2,Con_5Y1,Con_5Y2
Y2,Con_5Y1,Con_5Y2
Y2,Con_5Y1,Con_5Y2
Y2,Con_5Y1,Con_5Y2
Y2,Con_5Y1,Con_5Y2
Y2,Con_5Y1,Con_5Y2
Y2,Con_5Y1,Con_5Y2
Y2,Con_5Y1,Con_5Y2

2,Con_5Y1,Con_5Y2
2,Con_5Y1,Con_5Y2
2,Con_5Y1,Con_5Y2
2,Con_5Y1,Con_5Y2
2,Con_5Y1,Con_5Y2
2,Con_5Y1,Con_5Y2
2,Con_5Y1,Con_5Y2
2,Con_5Y1,Con_5Y2
2,Con_5Y1,Con_5Y2
2,Con_5Y1,Con_5Y2
2,Con_5Y1,Con_5Y2
2,Con_5Y1,Con_5Y2
2,Con_5Y1,Con_5Y2
2,Con_5Y1,Con_5Y2
2,Con_5Y1,Con_5Y2
2,Con_5Y1,Con_5Y2
2,Con_5Y1,Con_5Y2
2,Con_5Y1,Con_5Y2
2,Con_5Y1,Con_5Y2
2,Con_5Y1,Con_5Y2
2,Con_5Y1,Con_5Y2
2,Con_5Y1,Con_5Y2
2,Con_5Y1,Con_5Y2
2,Con_5Y1,Con_5Y2
2,Con_5Y1,Con_5Y2
2,Con_5Y1,Con_5Y2
2,Con_5Y1,Con_5Y2
2,Con_5Y1,Con_5Y2
2,Con_5Y1,Con_5Y2
2,Con_5Y1,Con_5Y2,Con_5Y3
2,Con_5Y1,Con_5Y2,Con_5Y3
2,Con_5Y1,Con_5Y2,Con_5Y3
2,Con_5Y1,Con_5Y2,Con_5Y3
2,Con_5Y1,Con_5Y2,Con_5Y3
2,Con_5Y1,Con_5Y2,Con_5Y3
2,Con_5Y1,Con_5Y2,Con_5Y3
2,Con_5Y1,Con_5Y2,Con_5Y3
2,Con_5Y1,Con_5Y2,Con_5Y3
2,Con_5Y1,Con_5Y2,Con_5Y3
2,Con_5Y1,Con_5Y2,Con_5Y3
2,Con_5Y1,Con_5Y2,Con_5Y3
2,Con_5Y1,Con_5Y2,Con_5Y3
2,Con_5Y1,Con_5Y2,Con_5Y3
2,Con_5Y1,Con_5Y2,Con_5Y3
2,Con_5Y1,Con_5Y2,Con_5Y3
2,Con_5Y1,Con_5Y2,Con_5Y3
2,Con_5Y1,Con_5Y2,Con_5Y3
2,Con_5Y1,Con_5Y2,Con_5Y3
2,Con_5Y1,Con_5Y2,Con_5Y3
2,Con_5Y1,Con_5Y2,Con_5Y3
2,Con_5Y1,Con_5Y2,Con_5Y3
2,Con_5Y1,Con_5Y2,Con_5Y3
2,Con_5Y1,Con_5Y2,Con_5Y3
2,Con_5Y1,Con_5Y2,Con_5Y3
2,Con_5Y1,Con_5Y2,Con_5Y3
2,Con_5Y1,Con_5Y2,Con_5Y3
2,Con_5Y1,Con_5Y2,Con_5Y3
2,Con_5Y1,Con_5Y2,Con_5Y3

Y2,Con_5Y1,Con_5Y2,Con_5Y3
Y2,Con_5Y1,Con_5Y2,Con_5Y3
Y2,Con_5Y1,Con_5Y2,Con_5Y3
Y2,Con_5Y1,Con_5Y2,Con_5Y3
Y2,Con_5Y1,Con_5Y2,Con_5Y3
Y2,Con_5Y1,Con_5Y2,Con_5Y3
Y2,Con_5Y1,Con_5Y2,Con_5Y3
Y2,Con_5Y1,Con_5Y2,Con_5Y3
Y2,Con_5Y1,Con_5Y2,Con_5Y3
Y2,Con_5Y1,Con_5Y2,Con_5Y3
Y2,Con_5Y1,Con_5Y2,Con_5Y3
Y2,Con_5Y1,Con_5Y2,Con_5Y3
Y2,Con_5Y1,Con_5Y2,Con_5Y3
Y2,Con_5Y1,Con_5Y2,Con_5Y3
Y2,Con_5Y1,Con_5Y2,Con_5Y3
Y2,Con_5Y1,Con_5Y2,Con_5Y3
Y2,Con_5Y1,Con_5Y2,Con_5Y3
Y2,Con_5Y1,Con_5Y2,Con_5Y3
Y2,Con_5Y1,Con_5Y2,Con_5Y3
Y2,Con_5Y1,Con_5Y2,Con_5Y3
Y2,Con_5Y1,Con_5Y2,Con_5Y3
Y2,Con_5Y1,Con_5Y2,Con_5Y3
Y2,Con_5Y1,Con_5Y2,Con_5Y3
Y2,Con_5Y1,Con_5Y2,Con_5Y3
Y2,Con_5Y1,Con_5Y2,Con_5Y3
Y2,Con_5Y1,Con_5Y2,Con_5Y3
Y2,Con_5Y1,Con_5Y2,Con_5Y3
Y2,Con_5Y1,Con_5Y2,Con_5Y3
Y2,Con_5Y1,Con_5Y2,Con_5Y3
Y2,Con_5Y1,Con_5Y2,Con_5Y3
Y2,Con_5Y1,Con_5Y2,Con_5Y3
Y2,Con_5Y1,Con_5Y2,Con_5Y3
Y2,Con_5Y1,Con_5Y2,Con_5Y3
Y2,Con_5Y1,Con_5Y2,Con_5Y3
Y2,Con_5Y1,Con_5Y2,Con_5Y3
Y2,Con_5Y1,Con_5Y2,Con_5Y3
Y2,Con_5Y1,Con_5Y2,Con_5Y3
Y2,Con_5Y1,Con_5Y2,Con_5Y3
Y2,Con_5Y1,Con_5Y2,Con_5Y3
Y2,Con_5Y1,Con_5Y2,Con_5Y3
Y2,Con_5Y1,Con_5Y3
Y2,Con_5Y1,Con_5Y3
Y2,Con_5Y1,Con_5Y3
Y2,Con_5Y1,Con_5Y3
Y2,Con_5Y1,Con_5Y3
Y2,Con_5Y1,Con_5Y3
Y2,Con_5Y1,Con_5Y3
Y2,Con_5Y1,Con_5Y3
Y2,Con_5Y1,Con_5Y3
Y2,Con_5Y1,Con_5Y3
Y2,Con_5Y1,Con_5Y3
Y2,Con_5Y1,Con_5Y3
Y2,Con_5Y2
Y2,Con_5Y2
Y2,Con_5Y2
Y2,Con_5Y2
Y2,Con_5Y2
Y2,Con_5Y2

Y2,Con_5Y2
Y2,Con_5Y2
Y2,Con_5Y2
Y2,Con_5Y2
Y2,Con_5Y2
Y2,Con_5Y2
Y2,Con_5Y2
Y2,Con_5Y2
Y2,Con_5Y2
Y2,Con_5Y2
Y2,Con_5Y2
Y2,Con_5Y2
Y2,Con_5Y2
Y2,Con_5Y2
Y2,Con_5Y2
Y2,Con_5Y2
Y2,Con_5Y2
Y2,Con_5Y2
Y2,Con_5Y2
Y2,Con_5Y2
Y2,Con_5Y2
Y2,Con_5Y2
Y2,Con_5Y2,Con_5Y3
Y2,Con_5Y2,Con_5Y3
Y2,Con_5Y2,Con_5Y3
Y2,Con_5Y2,Con_5Y3
Y2,Con_5Y2,Con_5Y3
Y2,Con_5Y2,Con_5Y3
Y2,Con_5Y2,Con_5Y3
Y2,Con_5Y2,Con_5Y3
Y2,Con_5Y2,Con_5Y3
Y2,Con_5Y2,Con_5Y3
Y2,Con_5Y2,Con_5Y3
Y2,Con_5Y2,Con_5Y3
Y2,Con_5Y2,Con_5Y3
Y2,Con_5Y2,Con_5Y3
Y2,Con_5Y2,Con_5Y3
Y2,Con_5Y2,Con_5Y3
Y2,Con_5Y2,Con_5Y3
Y2,Con_5Y2,Con_5Y3
Y2,Con_5Y2,Con_5Y3
Y2,Con_5Y2,Con_5Y3
Y2,Con_5Y2,Con_5Y3
Y2,Con_5Y2,Con_5Y3
Y2,Con_5Y2,Con_5Y3
Y2,Con_5Y2,Con_5Y3
Y2,Con_5Y2,Con_5Y3
Y2,Con_5Y2,Con_5Y3
Y2,Con_5Y2,Con_5Y3
Y2,Con_5Y2,Con_5Y3
Y2,Con_5Y3
Y2,Con_5Y3
Y2,Con_5Y3
Y2,Con_5Y3
Y2,Con_5Y3
Y2,Con_5Y3
Y2,Con_5Y3
Y2,Con_5Y3

Y2,Con_5Y3
Y2,Con_5Y3
Y2,Con_5Y3
Y2,Con_5Y3
Y2,Con_5Y3
Y2,Con_5Y3
Y2,Con_5Y3
Y2,Con_5Y3
Y2,Con_5Y3
Y2,Con_5Y3
Y2,Con_5Y3
Y2,Con_5Y3
Y2,CVB5_5Y3
Y2,CVB5_5Y3
Y2,CVB5_5Y3
Y2,CVB5_5Y3
Y2,CVB5_5Y3
Y2,CVB5_5Y3
Y2,CVB5_5Y3
Y2,CVB5_5Y3
Y2,CVB5_5Y3
Y2,CVB5_5Y3
Y2,CVB5_5Y3
Y2,CVB5_5Y3
Y2,CVB5_5Y3
Y2,CVB5_5Y3
Y2,CVB5_5Y3
Y2,CVB5_5Y3
Y2,CVB5_5Y3
Y2,CVB5_5Y3
Y2,CVB5_5Y3,Con_5Y1
Y2,CVB5_5Y3,Con_5Y1
Y2,CVB5_5Y3,Con_5Y1
Y2,CVB5_5Y3,Con_5Y1
Y2,CVB5_5Y3,Con_5Y1
Y2,CVB5_5Y3,Con_5Y1
Y2,CVB5_5Y3,Con_5Y1
Y2,CVB5_5Y3,Con_5Y1
Y2,CVB5_5Y3,Con_5Y1
Y2,CVB5_5Y3,Con_5Y1
Y2,CVB5_5Y3,Con_5Y1
Y2,CVB5_5Y3,Con_5Y1
Y2,CVB5_5Y3,Con_5Y1
Y2,CVB5_5Y3,Con_5Y1
Y2,CVB5_5Y3,Con_5Y1
Y2,CVB5_5Y3,Con_5Y1
Y2,CVB5_5Y3,Con_5Y1,Con_5Y2
Y2,CVB5_5Y3,Con_5Y1,Con_5Y2
Y2,CVB5_5Y3,Con_5Y1,Con_5Y2
Y2,CVB5_5Y3,Con_5Y1,Con_5Y2
Y2,CVB5_5Y3,Con_5Y1,Con_5Y2
Y2,CVB5_5Y3,Con_5Y1,Con_5Y2
Y2,CVB5_5Y3,Con_5Y1,Con_5Y2
Y2,CVB5_5Y3,Con_5Y1,Con_5Y2
Y2,CVB5_5Y3,Con_5Y1,Con_5Y2
Y2,CVB5_5Y3,Con_5Y1,Con_5Y2
Y2,CVB5_5Y3,Con_5Y1,Con_5Y2
Y2,CVB5_5Y3,Con_5Y1,Con_5Y2

Y2,CVB5_5Y3,Con_5Y1,Con_5Y2
Y2,CVB5_5Y3,Con_5Y1,Con_5Y2
Y2,CVB5_5Y3,Con_5Y1,Con_5Y2
Y2,CVB5_5Y3,Con_5Y1,Con_5Y2
Y2,CVB5_5Y3,Con_5Y1,Con_5Y2
Y2,CVB5_5Y3,Con_5Y1,Con_5Y2
Y2,CVB5_5Y3,Con_5Y1,Con_5Y2
Y2,CVB5_5Y3,Con_5Y1,Con_5Y2
Y2,CVB5_5Y3,Con_5Y1,Con_5Y2
Y2,CVB5_5Y3,Con_5Y1,Con_5Y2
Y2,CVB5_5Y3,Con_5Y1,Con_5Y2
Y2,CVB5_5Y3,Con_5Y1,Con_5Y2
Y2,CVB5_5Y3,Con_5Y1,Con_5Y2
Y2,CVB5_5Y3,Con_5Y1,Con_5Y2
Y2,CVB5_5Y3,Con_5Y1,Con_5Y2
Y2,CVB5_5Y3,Con_5Y1,Con_5Y2
Y2,CVB5_5Y3,Con_5Y1,Con_5Y2
Y2,CVB5_5Y3,Con_5Y1,Con_5Y2
Y2,CVB5_5Y3,Con_5Y1,Con_5Y2
Y2,CVB5_5Y3,Con_5Y1,Con_5Y2
Y2,CVB5_5Y3,Con_5Y1,Con_5Y2
Y2,CVB5_5Y3,Con_5Y1,Con_5Y2
Y2,CVB5_5Y3,Con_5Y1,Con_5Y2
Y2,CVB5_5Y3,Con_5Y1,Con_5Y2
Y2,CVB5_5Y3,Con_5Y1,Con_5Y2
Y2,CVB5_5Y3,Con_5Y1,Con_5Y2
Y2,CVB5_5Y3,Con_5Y1,Con_5Y2
Y2,CVB5_5Y3,Con_5Y1,Con_5Y2
Y2,CVB5_5Y3,Con_5Y1,Con_5Y2
Y2,CVB5_5Y3,Con_5Y1,Con_5Y2
Y2,CVB5_5Y3,Con_5Y1,Con_5Y2
Y2,CVB5_5Y3,Con_5Y1,Con_5Y2
Y2,CVB5_5Y3,Con_5Y1,Con_5Y2
Y2,CVB5_5Y3,Con_5Y1,Con_5Y2
Y2,CVB5_5Y3,Con_5Y1,Con_5Y2
Y2,CVB5_5Y3,Con_5Y1,Con_5Y2
Y2,CVB5_5Y3,Con_5Y1,Con_5Y2
Y2,CVB5_5Y3,Con_5Y1,Con_5Y2
Y2,CVB5_5Y3,Con_5Y1,Con_5Y2
Y2,CVB5_5Y3,Con_5Y1,Con_5Y2
Y2,CVB5_5Y3,Con_5Y1,Con_5Y2
Y2,CVB5_5Y3,Con_5Y1,Con_5Y2
Y2,CVB5_5Y3,Con_5Y1,Con_5Y2
Y2,CVB5_5Y3,Con_5Y1,Con_5Y2
Y2,CVB5_5Y3,Con_5Y1,Con_5Y2
Y2,CVB5_5Y3,Con_5Y1,Con_5Y2
Y2,CVB5_5Y3,Con_5Y1,Con_5Y2
Y2,CVB5_5Y3,Con_5Y1,Con_5Y2
Y2,CVB5_5Y3,Con_5Y1,Con_5Y2
Y2,CVB5_5Y3,Con_5Y1,Con_5Y2
Y2,CVB5_5Y3,Con_5Y1,Con_5Y2
Y2,CVB5_5Y3,Con_5Y1,Con_5Y2
Y2,CVB5_5Y3,Con_5Y1,Con_5Y2
Y2,CVB5_5Y3,Con_5Y1,Con_5Y2
Y2,CVB5_5Y3,Con_5Y1,Con_5Y2
Y2,CVB5_5Y3,Con_5Y1,Con_5Y2
Y2,CVB5_5Y3,Con_5Y1,Con_5Y2
Y2,CVB5_5Y3,Con_5Y1,Con_5Y2

Y2,CVB5_5Y3,Con_5Y1,Con_5Y2
Y2,CVB5_5Y3,Con_5Y1,Con_5Y2
Y2,CVB5_5Y3,Con_5Y1,Con_5Y2
Y2,CVB5_5Y3,Con_5Y1,Con_5Y2
Y2,CVB5_5Y3,Con_5Y1,Con_5Y2
Y2,CVB5_5Y3,Con_5Y1,Con_5Y2
Y2,CVB5_5Y3,Con_5Y1,Con_5Y2
Y2,CVB5_5Y3,Con_5Y1,Con_5Y2
Y2,CVB5_5Y3,Con_5Y1,Con_5Y2
Y2,CVB5_5Y3,Con_5Y1,Con_5Y2
Y2,CVB5_5Y3,Con_5Y1,Con_5Y2
Y2,CVB5_5Y3,Con_5Y1,Con_5Y2,Con_5Y3
Y2,CVB5_5Y3,Con_5Y1,Con_5Y2,Con_5Y3
Y2,CVB5_5Y3,Con_5Y1,Con_5Y2,Con_5Y3
Y2,CVB5_5Y3,Con_5Y1,Con_5Y2,Con_5Y3
Y2,CVB5_5Y3,Con_5Y1,Con_5Y2,Con_5Y3
Y2,CVB5_5Y3,Con_5Y1,Con_5Y2,Con_5Y3
Y2,CVB5_5Y3,Con_5Y1,Con_5Y2,Con_5Y3
Y2,CVB5_5Y3,Con_5Y1,Con_5Y2,Con_5Y3
Y2,CVB5_5Y3,Con_5Y1,Con_5Y2,Con_5Y3
Y2,CVB5_5Y3,Con_5Y1,Con_5Y2,Con_5Y3
Y2,CVB5_5Y3,Con_5Y1,Con_5Y2,Con_5Y3
Y2,CVB5_5Y3,Con_5Y1,Con_5Y2,Con_5Y3
Y2,CVB5_5Y3,Con_5Y1,Con_5Y2,Con_5Y3
Y2,CVB5_5Y3,Con_5Y1,Con_5Y2,Con_5Y3
Y2,CVB5_5Y3,Con_5Y1,Con_5Y2,Con_5Y3
Y2,CVB5_5Y3,Con_5Y1,Con_5Y2,Con_5Y3
Y2,CVB5_5Y3,Con_5Y1,Con_5Y2,Con_5Y3
Y2,CVB5_5Y3,Con_5Y1,Con_5Y2,Con_5Y3
Y2,CVB5_5Y3,Con_5Y1,Con_5Y2,Con_5Y3
Y2,CVB5_5Y3,Con_5Y1,Con_5Y2,Con_5Y3
Y2,CVB5_5Y3,Con_5Y1,Con_5Y2,Con_5Y3
Y2,CVB5_5Y3,Con_5Y1,Con_5Y2,Con_5Y3
Y2,CVB5_5Y3,Con_5Y1,Con_5Y2,Con_5Y3
Y2,CVB5_5Y3,Con_5Y1,Con_5Y2,Con_5Y3
Y2,CVB5_5Y3,Con_5Y1,Con_5Y2,Con_5Y3
Y2,CVB5_5Y3,Con_5Y1,Con_5Y2,Con_5Y3
Y2,CVB5_5Y3,Con_5Y1,Con_5Y2,Con_5Y3
Y2,CVB5_5Y3,Con_5Y1,Con_5Y2,Con_5Y3
Y2,CVB5_5Y3,Con_5Y1,Con_5Y2,Con_5Y3
Y2,CVB5_5Y3,Con_5Y1,Con_5Y2,Con_5Y3
Y2,CVB5_5Y3,Con_5Y1,Con_5Y2,Con_5Y3
Y2,CVB5_5Y3,Con_5Y1,Con_5Y2,Con_5Y3
Y2,CVB5_5Y3,Con_5Y1,Con_5Y2,Con_5Y3
Y2,CVB5_5Y3,Con_5Y1,Con_5Y2,Con_5Y3
Y2,CVB5_5Y3,Con_5Y1,Con_5Y2,Con_5Y3
Y2,CVB5_5Y3,Con_5Y1,Con_5Y2,Con_5Y3
Y2,CVB5_5Y3,Con_5Y1,Con_5Y2,Con_5Y3
Y2,CVB5_5Y3,Con_5Y1,Con_5Y2,Con_5Y3
Y2,CVB5_5Y3,Con_5Y1,Con_5Y2,Con_5Y3
Y2,CVB5_5Y3,Con_5Y1,Con_5Y2,Con_5Y3
Y2,CVB5_5Y3,Con_5Y1,Con_5Y2,Con_5Y3
Y2,CVB5_5Y3,Con_5Y1,Con_5Y2,Con_5Y3
Y2,CVB5_5Y3,Con_5Y1,Con_5Y2,Con_5Y3
Y2,CVB5_5Y3,Con_5Y1,Con_5Y2,Con_5Y3
Y2,CVB5_5Y3,Con_5Y1,Con_5Y2,Con_5Y3
Y2,CVB5_5Y3,Con_5Y1,Con_5Y2,Con_5Y3
Y2,CVB5_5Y3,Con_5Y1,Con_5Y2,Con_5Y3

Y2,CVB5_5Y3,Con_5Y1,Con_5Y2,Con_5Y3
Y2,CVB5_5Y3,Con_5Y1,Con_5Y2,Con_5Y3
Y2,CVB5_5Y3,Con_5Y1,Con_5Y2,Con_5Y3
Y2,CVB5_5Y3,Con_5Y1,Con_5Y2,Con_5Y3
Y2,CVB5_5Y3,Con_5Y1,Con_5Y2,Con_5Y3
Y2,CVB5_5Y3,Con_5Y1,Con_5Y2,Con_5Y3
Y2,CVB5_5Y3,Con_5Y1,Con_5Y2,Con_5Y3
Y2,CVB5_5Y3,Con_5Y1,Con_5Y2,Con_5Y3
Y2,CVB5_5Y3,Con_5Y1,Con_5Y2,Con_5Y3
Y2,CVB5_5Y3,Con_5Y1,Con_5Y2,Con_5Y3
Y2,CVB5_5Y3,Con_5Y1,Con_5Y2,Con_5Y3
Y2,CVB5_5Y3,Con_5Y1,Con_5Y2,Con_5Y3
Y2,CVB5_5Y3,Con_5Y1,Con_5Y2,Con_5Y3
Y2,CVB5_5Y3,Con_5Y1,Con_5Y2,Con_5Y3
Y2,CVB5_5Y3,Con_5Y1,Con_5Y2,Con_5Y3
Y2,CVB5_5Y3,Con_5Y1,Con_5Y2,Con_5Y3
Y2,CVB5_5Y3,Con_5Y1,Con_5Y2,Con_5Y3
Y2,CVB5_5Y3,Con_5Y1,Con_5Y2,Con_5Y3
Y2,CVB5_5Y3,Con_5Y1,Con_5Y2,Con_5Y3
Y2,CVB5_5Y3,Con_5Y1,Con_5Y2,Con_5Y3
Y2,CVB5_5Y3,Con_5Y1,Con_5Y2,Con_5Y3
Y2,CVB5_5Y3,Con_5Y1,Con_5Y2,Con_5Y3
Y2,CVB5_5Y3,Con_5Y1,Con_5Y2,Con_5Y3
Y2,CVB5_5Y3,Con_5Y1,Con_5Y2,Con_5Y3
Y2,CVB5_5Y3,Con_5Y1,Con_5Y2,Con_5Y3
Y2,CVB5_5Y3,Con_5Y1,Con_5Y2,Con_5Y3
Y2,CVB5_5Y3,Con_5Y1,Con_5Y2,Con_5Y3
Y2,CVB5_5Y3,Con_5Y1,Con_5Y2,Con_5Y3
Y2,CVB5_5Y3,Con_5Y1,Con_5Y2,Con_5Y3
Y2,CVB5_5Y3,Con_5Y1,Con_5Y2,Con_5Y3
Y2,CVB5_5Y3,Con_5Y1,Con_5Y2,Con_5Y3
Y2,CVB5_5Y3,Con_5Y1,Con_5Y2,Con_5Y3
Y2,CVB5_5Y3,Con_5Y1,Con_5Y2,Con_5Y3
Y2,CVB5_5Y3,Con_5Y1,Con_5Y2,Con_5Y3
Y2,CVB5_5Y3,Con_5Y1,Con_5Y2,Con_5Y3
Y2,CVB5_5Y3,Con_5Y1,Con_5Y2,Con_5Y3
Y2,CVB5_5Y3,Con_5Y1,Con_5Y2,Con_5Y3
Y2,CVB5_5Y3,Con_5Y1,Con_5Y2,Con_5Y3
Y2,CVB5_5Y3,Con_5Y1,Con_5Y2,Con_5Y3
Y2,CVB5_5Y3,Con_5Y1,Con_5Y2,Con_5Y3
Y2,CVB5_5Y3,Con_5Y1,Con_5Y2,Con_5Y3
Y2,CVB5_5Y3,Con_5Y1,Con_5Y2,Con_5Y3
Y2,CVB5_5Y3,Con_5Y1,Con_5Y2,Con_5Y3
Y2,CVB5_5Y3,Con_5Y1,Con_5Y2,Con_5Y3
Y2,CVB5_5Y3,Con_5Y1,Con_5Y2,Con_5Y3
Y2,CVB5_5Y3,Con_5Y1,Con_5Y2,Con_5Y3
Y2,CVB5_5Y3,Con_5Y1,Con_5Y2,Con_5Y3
Y2,CVB5_5Y3,Con_5Y1,Con_5Y2,Con_5Y3
Y2,CVB5_5Y3,Con_5Y1,Con_5Y2,Con_5Y3
Y2,CVB5_5Y3,Con_5Y1,Con_5Y2,Con_5Y3
Y2,CVB5_5Y3,Con_5Y1,Con_5Y2,Con_5Y3
Y2,CVB5_5Y3,Con_5Y1,Con_5Y2,Con_5Y3
Y2,CVB5_5Y3,Con_5Y1,Con_5Y2,Con_5Y3
Y2,CVB5_5Y3,Con_5Y1,Con_5Y2,Con_5Y3
Y2,CVB5_5Y3,Con_5Y1,Con_5Y2,Con_5Y3
Y2,CVB5_5Y3,Con_5Y1,Con_5Y2,Con_5Y3
Y2,CVB5_5Y3,Con_5Y1,Con_5Y2,Con_5Y3
Y2,CVB5_5Y3,Con_5Y1,Con_5Y2,Con_5Y3

Y2,CVB5_5Y3,Con_5Y1,Con_5Y2,Con_5Y3
Y2,CVB5_5Y3,Con_5Y1,Con_5Y2,Con_5Y3
Y2,CVB5_5Y3,Con_5Y1,Con_5Y2,Con_5Y3
Y2,CVB5_5Y3,Con_5Y1,Con_5Y2,Con_5Y3
Y2,CVB5_5Y3,Con_5Y1,Con_5Y2,Con_5Y3
Y2,CVB5_5Y3,Con_5Y1,Con_5Y2,Con_5Y3
Y2,CVB5_5Y3,Con_5Y1,Con_5Y2,Con_5Y3
Y2,CVB5_5Y3,Con_5Y1,Con_5Y2,Con_5Y3
Y2,CVB5_5Y3,Con_5Y1,Con_5Y2,Con_5Y3
Y2,CVB5_5Y3,Con_5Y1,Con_5Y2,Con_5Y3
Y2,CVB5_5Y3,Con_5Y1,Con_5Y2,Con_5Y3
Y2,CVB5_5Y3,Con_5Y1,Con_5Y2,Con_5Y3
Y2,CVB5_5Y3,Con_5Y1,Con_5Y2,Con_5Y3
Y2,CVB5_5Y3,Con_5Y1,Con_5Y2,Con_5Y3
Y2,CVB5_5Y3,Con_5Y1,Con_5Y2,Con_5Y3
Y2,CVB5_5Y3,Con_5Y1,Con_5Y2,Con_5Y3
Y2,CVB5_5Y3,Con_5Y1,Con_5Y2,Con_5Y3
Y2,CVB5_5Y3,Con_5Y1,Con_5Y2,Con_5Y3
Y2,CVB5_5Y3,Con_5Y1,Con_5Y2,Con_5Y3
Y2,CVB5_5Y3,Con_5Y1,Con_5Y2,Con_5Y3
Y2,CVB5_5Y3,Con_5Y1,Con_5Y2,Con_5Y3
Y2,CVB5_5Y3,Con_5Y1,Con_5Y2,Con_5Y3
Y2,CVB5_5Y3,Con_5Y1,Con_5Y2,Con_5Y3
Y2,CVB5_5Y3,Con_5Y1,Con_5Y2,Con_5Y3
Y2,CVB5_5Y3,Con_5Y1,Con_5Y2,Con_5Y3
Y2,CVB5_5Y3,Con_5Y1,Con_5Y2,Con_5Y3
Y2,CVB5_5Y3,Con_5Y1,Con_5Y2,Con_5Y3
Y2,CVB5_5Y3,Con_5Y1,Con_5Y2,Con_5Y3
Y2,CVB5_5Y3,Con_5Y1,Con_5Y2,Con_5Y3
Y2,CVB5_5Y3,Con_5Y1,Con_5Y2,Con_5Y3
Y2,CVB5_5Y3,Con_5Y1,Con_5Y2,Con_5Y3
Y2,CVB5_5Y3,Con_5Y1,Con_5Y2,Con_5Y3
Y2,CVB5_5Y3,Con_5Y1,Con_5Y2,Con_5Y3
Y2,CVB5_5Y3,Con_5Y1,Con_5Y2,Con_5Y3
Y2,CVB5_5Y3,Con_5Y1,Con_5Y2,Con_5Y3
Y2,CVB5_5Y3,Con_5Y1,Con_5Y2,Con_5Y3
Y2,CVB5_5Y3,Con_5Y1,Con_5Y2,Con_5Y3
Y2,CVB5_5Y3,Con_5Y1,Con_5Y2,Con_5Y3
Y2,CVB5_5Y3,Con_5Y1,Con_5Y2,Con_5Y3
Y2,CVB5_5Y3,Con_5Y1,Con_5Y2,Con_5Y3
Y2,CVB5_5Y3,Con_5Y1,Con_5Y2,Con_5Y3
Y2,CVB5_5Y3,Con_5Y1,Con_5Y2,Con_5Y3
Y2,CVB5_5Y3,Con_5Y1,Con_5Y2,Con_5Y3
Y2,CVB5_5Y3,Con_5Y1,Con_5Y2,Con_5Y3
Y2,CVB5_5Y3,Con_5Y1,Con_5Y2,Con_5Y3
Y2,CVB5_5Y3,Con_5Y1,Con_5Y2,Con_5Y3
Y2,CVB5_5Y3,Con_5Y1,Con_5Y2,Con_5Y3
Y2,CVB5_5Y3,Con_5Y1,Con_5Y2,Con_5Y3
Y2,CVB5_5Y3,Con_5Y1,Con_5Y2,Con_5Y3
Y2,CVB5_5Y3,Con_5Y1,Con_5Y2,Con_5Y3
Y2,CVB5_5Y3,Con_5Y1,Con_5Y2,Con_5Y3
Y2,CVB5_5Y3,Con_5Y1,Con_5Y2,Con_5Y3
Y2,CVB5_5Y3,Con_5Y1,Con_5Y2,Con_5Y3
Y2,CVB5_5Y3,Con_5Y1,Con_5Y2,Con_5Y3
Y2,CVB5_5Y3,Con_5Y1,Con_5Y2,Con_5Y3
Y2,CVB5_5Y3,Con_5Y1,Con_5Y2,Con_5Y3
Y2,CVB5_5Y3,Con_5Y1,Con_5Y2,Con_5Y3
Y2,CVB5_5Y3,Con_5Y1,Con_5Y2,Con_5Y3

Y2,CVB5_5Y3,Con_5Y1,Con_5Y2,Con_5Y3
Y2,CVB5_5Y3,Con_5Y1,Con_5Y2,Con_5Y3
Y2,CVB5_5Y3,Con_5Y1,Con_5Y2,Con_5Y3
Y2,CVB5_5Y3,Con_5Y1,Con_5Y2,Con_5Y3
Y2,CVB5_5Y3,Con_5Y1,Con_5Y2,Con_5Y3
Y2,CVB5_5Y3,Con_5Y1,Con_5Y2,Con_5Y3
Y2,CVB5_5Y3,Con_5Y1,Con_5Y2,Con_5Y3
Y2,CVB5_5Y3,Con_5Y1,Con_5Y2,Con_5Y3
Y2,CVB5_5Y3,Con_5Y1,Con_5Y2,Con_5Y3
Y2,CVB5_5Y3,Con_5Y1,Con_5Y2,Con_5Y3
Y2,CVB5_5Y3,Con_5Y1,Con_5Y2,Con_5Y3
Y2,CVB5_5Y3,Con_5Y1,Con_5Y2,Con_5Y3
Y2,CVB5_5Y3,Con_5Y1,Con_5Y2,Con_5Y3
Y2,CVB5_5Y3,Con_5Y1,Con_5Y2,Con_5Y3
Y2,CVB5_5Y3,Con_5Y1,Con_5Y2,Con_5Y3
Y2,CVB5_5Y3,Con_5Y1,Con_5Y2,Con_5Y3
Y2,CVB5_5Y3,Con_5Y1,Con_5Y2,Con_5Y3
Y2,CVB5_5Y3,Con_5Y1,Con_5Y2,Con_5Y3
Y2,CVB5_5Y3,Con_5Y1,Con_5Y2,Con_5Y3
Y2,CVB5_5Y3,Con_5Y1,Con_5Y2,Con_5Y3
Y2,CVB5_5Y3,Con_5Y1,Con_5Y2,Con_5Y3
Y2,CVB5_5Y3,Con_5Y1,Con_5Y2,Con_5Y3
Y2,CVB5_5Y3,Con_5Y1,Con_5Y2,Con_5Y3
Y2,CVB5_5Y3,Con_5Y1,Con_5Y2,Con_5Y3
Y2,CVB5_5Y3,Con_5Y1,Con_5Y2,Con_5Y3
Y2,CVB5_5Y3,Con_5Y1,Con_5Y2,Con_5Y3
Y2,CVB5_5Y3,Con_5Y1,Con_5Y2,Con_5Y3
Y2,CVB5_5Y3,Con_5Y1,Con_5Y2,Con_5Y3
Y2,CVB5_5Y3,Con_5Y1,Con_5Y2,Con_5Y3
Y2,CVB5_5Y3,Con_5Y1,Con_5Y2,Con_5Y3
Y2,CVB5_5Y3,Con_5Y1,Con_5Y2,Con_5Y3
Y2,CVB5_5Y3,Con_5Y1,Con_5Y2,Con_5Y3
Y2,CVB5_5Y3,Con_5Y1,Con_5Y2,Con_5Y3
Y2,CVB5_5Y3,Con_5Y1,Con_5Y2,Con_5Y3
Y2,CVB5_5Y3,Con_5Y1,Con_5Y2,Con_5Y3
Y2,CVB5_5Y3,Con_5Y1,Con_5Y2,Con_5Y3
Y2,CVB5_5Y3,Con_5Y1,Con_5Y2,Con_5Y3
Y2,CVB5_5Y3,Con_5Y1,Con_5Y2,Con_5Y3
Y2,CVB5_5Y3,Con_5Y1,Con_5Y2,Con_5Y3
Y2,CVB5_5Y3,Con_5Y1,Con_5Y2,Con_5Y3
Y2,CVB5_5Y3,Con_5Y1,Con_5Y2,Con_5Y3
Y2,CVB5_5Y3,Con_5Y1,Con_5Y2,Con_5Y3
Y2,CVB5_5Y3,Con_5Y1,Con_5Y2,Con_5Y3
Y2,CVB5_5Y3,Con_5Y1,Con_5Y2,Con_5Y3
Y2,CVB5_5Y3,Con_5Y1,Con_5Y2,Con_5Y3
Y2,CVB5_5Y3,Con_5Y1,Con_5Y2,Con_5Y3
Y2,CVB5_5Y3,Con_5Y1,Con_5Y2,Con_5Y3
Y2,CVB5_5Y3,Con_5Y1,Con_5Y2,Con_5Y3
Y2,CVB5_5Y3,Con_5Y1,Con_5Y2,Con_5Y3
Y2,CVB5_5Y3,Con_5Y1,Con_5Y2,Con_5Y3
Y2,CVB5_5Y3,Con_5Y1,Con_5Y2,Con_5Y3
Y2,CVB5_5Y3,Con_5Y1,Con_5Y2,Con_5Y3
Y2,CVB5_5Y3,Con_5Y1,Con_5Y2,Con_5Y3
Y2,CVB5_5Y3,Con_5Y1,Con_5Y2,Con_5Y3
Y2,CVB5_5Y3,Con_5Y1,Con_5Y2,Con_5Y3
Y2,CVB5_5Y3,Con_5Y1,Con_5Y2,Con_5Y3
Y2,CVB5_5Y3,Con_5Y1,Con_5Y2,Con_5Y3
Y2,CVB5_5Y3,Con_5Y1,Con_5Y2,Con_5Y3

Y2,CVB5_5Y3,Con_5Y1,Con_5Y2,Con_5Y3
Y2,CVB5_5Y3,Con_5Y1,Con_5Y2,Con_5Y3
Y2,CVB5_5Y3,Con_5Y1,Con_5Y2,Con_5Y3
Y2,CVB5_5Y3,Con_5Y1,Con_5Y2,Con_5Y3
Y2,CVB5_5Y3,Con_5Y1,Con_5Y2,Con_5Y3
Y2,CVB5_5Y3,Con_5Y1,Con_5Y2,Con_5Y3
Y2,CVB5_5Y3,Con_5Y1,Con_5Y2,Con_5Y3
Y2,CVB5_5Y3,Con_5Y1,Con_5Y2,Con_5Y3
Y2,CVB5_5Y3,Con_5Y1,Con_5Y2,Con_5Y3
Y2,CVB5_5Y3,Con_5Y1,Con_5Y2,Con_5Y3
Y2,CVB5_5Y3,Con_5Y1,Con_5Y2,Con_5Y3
Y2,CVB5_5Y3,Con_5Y1,Con_5Y2,Con_5Y3
Y2,CVB5_5Y3,Con_5Y1,Con_5Y2,Con_5Y3
Y2,CVB5_5Y3,Con_5Y1,Con_5Y2,Con_5Y3
Y2,CVB5_5Y3,Con_5Y1,Con_5Y2,Con_5Y3
Y2,CVB5_5Y3,Con_5Y1,Con_5Y2,Con_5Y3
Y2,CVB5_5Y3,Con_5Y1,Con_5Y2,Con_5Y3
Y2,CVB5_5Y3,Con_5Y1,Con_5Y2,Con_5Y3
Y2,CVB5_5Y3,Con_5Y1,Con_5Y2,Con_5Y3
Y2,CVB5_5Y3,Con_5Y1,Con_5Y2,Con_5Y3
Y2,CVB5_5Y3,Con_5Y1,Con_5Y2,Con_5Y3
Y2,CVB5_5Y3,Con_5Y1,Con_5Y2,Con_5Y3
Y2,CVB5_5Y3,Con_5Y1,Con_5Y2,Con_5Y3
Y2,CVB5_5Y3,Con_5Y1,Con_5Y2,Con_5Y3
Y2,CVB5_5Y3,Con_5Y1,Con_5Y2,Con_5Y3
Y2,CVB5_5Y3,Con_5Y1,Con_5Y2,Con_5Y3
Y2,CVB5_5Y3,Con_5Y1,Con_5Y2,Con_5Y3
Y2,CVB5_5Y3,Con_5Y1,Con_5Y2,Con_5Y3
Y2,CVB5_5Y3,Con_5Y1,Con_5Y2,Con_5Y3
Y2,CVB5_5Y3,Con_5Y1,Con_5Y2,Con_5Y3
Y2,CVB5_5Y3,Con_5Y1,Con_5Y2,Con_5Y3
Y2,CVB5_5Y3,Con_5Y1,Con_5Y2,Con_5Y3
Y2,CVB5_5Y3,Con_5Y1,Con_5Y2,Con_5Y3
Y2,CVB5_5Y3,Con_5Y1,Con_5Y2,Con_5Y3
Y2,CVB5_5Y3,Con_5Y1,Con_5Y2,Con_5Y3
Y2,CVB5_5Y3,Con_5Y1,Con_5Y2,Con_5Y3
Y2,CVB5_5Y3,Con_5Y1,Con_5Y2,Con_5Y3
Y2,CVB5_5Y3,Con_5Y1,Con_5Y2,Con_5Y3
Y2,CVB5_5Y3,Con_5Y1,Con_5Y2,Con_5Y3
Y2,CVB5_5Y3,Con_5Y1,Con_5Y2,Con_5Y3
Y2,CVB5_5Y3,Con_5Y1,Con_5Y2,Con_5Y3
Y2,CVB5_5Y3,Con_5Y1,Con_5Y2,Con_5Y3
Y2,CVB5_5Y3,Con_5Y1,Con_5Y2,Con_5Y3
Y2,CVB5_5Y3,Con_5Y1,Con_5Y2,Con_5Y3
Y2,CVB5_5Y3,Con_5Y1,Con_5Y2,Con_5Y3
Y2,CVB5_5Y3,Con_5Y1,Con_5Y2,Con_5Y3
Y2,CVB5_5Y3,Con_5Y1,Con_5Y2,Con_5Y3
Y2,CVB5_5Y3,Con_5Y1,Con_5Y2,Con_5Y3
Y2,CVB5_5Y3,Con_5Y1,Con_5Y2,Con_5Y3
Y2,CVB5_5Y3,Con_5Y1,Con_5Y2,Con_5Y3
Y2,CVB5_5Y3,Con_5Y1,Con_5Y2,Con_5Y3
Y2,CVB5_5Y3,Con_5Y1,Con_5Y2,Con_5Y3
Y2,CVB5_5Y3,Con_5Y1,Con_5Y2,Con_5Y3
Y2,CVB5_5Y3,Con_5Y1,Con_5Y2,Con_5Y3
Y2,CVB5_5Y3,Con_5Y1,Con_5Y2,Con_5Y3
Y2,CVB5_5Y3,Con_5Y1,Con_5Y2,Con_5Y3
Y2,CVB5_5Y3,Con_5Y1,Con_5Y2,Con_5Y3
Y2,CVB5_5Y3,Con_5Y1,Con_5Y2,Con_5Y3

Y2,CVB5_5Y3,Con_5Y1,Con_5Y2,Con_5Y3
Y2,CVB5_5Y3,Con_5Y1,Con_5Y2,Con_5Y3
Y2,CVB5_5Y3,Con_5Y1,Con_5Y2,Con_5Y3
Y2,CVB5_5Y3,Con_5Y1,Con_5Y2,Con_5Y3
Y2,CVB5_5Y3,Con_5Y1,Con_5Y2,Con_5Y3
Y2,CVB5_5Y3,Con_5Y1,Con_5Y2,Con_5Y3
Y2,CVB5_5Y3,Con_5Y1,Con_5Y2,Con_5Y3
Y2,CVB5_5Y3,Con_5Y1,Con_5Y2,Con_5Y3
Y2,CVB5_5Y3,Con_5Y1,Con_5Y2,Con_5Y3
Y2,CVB5_5Y3,Con_5Y1,Con_5Y2,Con_5Y3
Y2,CVB5_5Y3,Con_5Y1,Con_5Y2,Con_5Y3
Y2,CVB5_5Y3,Con_5Y1,Con_5Y2,Con_5Y3
Y2,CVB5_5Y3,Con_5Y1,Con_5Y2,Con_5Y3
Y2,CVB5_5Y3,Con_5Y1,Con_5Y2,Con_5Y3
Y2,CVB5_5Y3,Con_5Y1,Con_5Y2,Con_5Y3
Y2,CVB5_5Y3,Con_5Y1,Con_5Y2,Con_5Y3
Y2,CVB5_5Y3,Con_5Y1,Con_5Y2,Con_5Y3
Y2,CVB5_5Y3,Con_5Y1,Con_5Y2,Con_5Y3
Y2,CVB5_5Y3,Con_5Y1,Con_5Y2,Con_5Y3
Y2,CVB5_5Y3,Con_5Y1,Con_5Y2,Con_5Y3
Y2,CVB5_5Y3,Con_5Y1,Con_5Y2,Con_5Y3
Y2,CVB5_5Y3,Con_5Y1,Con_5Y2,Con_5Y3
Y2,CVB5_5Y3,Con_5Y1,Con_5Y2,Con_5Y3
Y2,CVB5_5Y3,Con_5Y1,Con_5Y2,Con_5Y3
Y2,CVB5_5Y3,Con_5Y1,Con_5Y2,Con_5Y3
Y2,CVB5_5Y3,Con_5Y1,Con_5Y2,Con_5Y3
Y2,CVB5_5Y3,Con_5Y1,Con_5Y2,Con_5Y3
Y2,CVB5_5Y3,Con_5Y1,Con_5Y2,Con_5Y3
Y2,CVB5_5Y3,Con_5Y1,Con_5Y2,Con_5Y3
Y2,CVB5_5Y3,Con_5Y1,Con_5Y2,Con_5Y3
Y2,CVB5_5Y3,Con_5Y1,Con_5Y2,Con_5Y3
Y2,CVB5_5Y3,Con_5Y1,Con_5Y2,Con_5Y3
Y2,CVB5_5Y3,Con_5Y1,Con_5Y2,Con_5Y3
Y2,CVB5_5Y3,Con_5Y1,Con_5Y2,Con_5Y3
Y2,CVB5_5Y3,Con_5Y1,Con_5Y2,Con_5Y3
Y2,CVB5_5Y3,Con_5Y1,Con_5Y2,Con_5Y3
Y2,CVB5_5Y3,Con_5Y1,Con_5Y2,Con_5Y3
Y2,CVB5_5Y3,Con_5Y1,Con_5Y2,Con_5Y3
Y2,CVB5_5Y3,Con_5Y1,Con_5Y2,Con_5Y3
Y2,CVB5_5Y3,Con_5Y1,Con_5Y2,Con_5Y3
Y2,CVB5_5Y3,Con_5Y1,Con_5Y2,Con_5Y3
Y2,CVB5_5Y3,Con_5Y1,Con_5Y2,Con_5Y3
Y2,CVB5_5Y3,Con_5Y1,Con_5Y2,Con_5Y3
Y2,CVB5_5Y3,Con_5Y1,Con_5Y2,Con_5Y3
Y2,CVB5_5Y3,Con_5Y1,Con_5Y2,Con_5Y3
Y2,CVB5_5Y3,Con_5Y1,Con_5Y2,Con_5Y3
Y2,CVB5_5Y3,Con_5Y1,Con_5Y2,Con_5Y3
Y2,CVB5_5Y3,Con_5Y1,Con_5Y2,Con_5Y3
Y2,CVB5_5Y3,Con_5Y1,Con_5Y2,Con_5Y3
Y2,CVB5_5Y3,Con_5Y1,Con_5Y2,Con_5Y3
Y2,CVB5_5Y3,Con_5Y1,Con_5Y2,Con_5Y3
Y2,CVB5_5Y3,Con_5Y1,Con_5Y2,Con_5Y3
Y2,CVB5_5Y3,Con_5Y1,Con_5Y2,Con_5Y3
Y2,CVB5_5Y3,Con_5Y1,Con_5Y2,Con_5Y3
Y2,CVB5_5Y3,Con_5Y1,Con_5Y2,Con_5Y3
Y2,CVB5_5Y3,Con_5Y1,Con_5Y2,Con_5Y3
Y2,CVB5_5Y3,Con_5Y1,Con_5Y2,Con_5Y3
Y2,CVB5_5Y3,Con_5Y1,Con_5Y2,Con_5Y3

Y2,CVB5_5Y3,Con_5Y1,Con_5Y2,Con_5Y3
Y2,CVB5_5Y3,Con_5Y1,Con_5Y2,Con_5Y3
Y2,CVB5_5Y3,Con_5Y1,Con_5Y2,Con_5Y3
Y2,CVB5_5Y3,Con_5Y1,Con_5Y2,Con_5Y3
Y2,CVB5_5Y3,Con_5Y1,Con_5Y2,Con_5Y3
Y2,CVB5_5Y3,Con_5Y1,Con_5Y2,Con_5Y3
Y2,CVB5_5Y3,Con_5Y1,Con_5Y2,Con_5Y3
Y2,CVB5_5Y3,Con_5Y1,Con_5Y2,Con_5Y3
Y2,CVB5_5Y3,Con_5Y1,Con_5Y2,Con_5Y3
Y2,CVB5_5Y3,Con_5Y1,Con_5Y2,Con_5Y3
Y2,CVB5_5Y3,Con_5Y1,Con_5Y2,Con_5Y3
Y2,CVB5_5Y3,Con_5Y1,Con_5Y2,Con_5Y3
Y2,CVB5_5Y3,Con_5Y1,Con_5Y2,Con_5Y3
Y2,CVB5_5Y3,Con_5Y1,Con_5Y2,Con_5Y3
Y2,CVB5_5Y3,Con_5Y1,Con_5Y2,Con_5Y3
Y2,CVB5_5Y3,Con_5Y1,Con_5Y2,Con_5Y3
Y2,CVB5_5Y3,Con_5Y1,Con_5Y2,Con_5Y3
Y2,CVB5_5Y3,Con_5Y1,Con_5Y2,Con_5Y3
Y2,CVB5_5Y3,Con_5Y1,Con_5Y2,Con_5Y3
Y2,CVB5_5Y3,Con_5Y1,Con_5Y2,Con_5Y3
Y2,CVB5_5Y3,Con_5Y1,Con_5Y2,Con_5Y3
Y2,CVB5_5Y3,Con_5Y1,Con_5Y2,Con_5Y3
Y2,CVB5_5Y3,Con_5Y1,Con_5Y2,Con_5Y3
Y2,CVB5_5Y3,Con_5Y1,Con_5Y2,Con_5Y3
Y2,CVB5_5Y3,Con_5Y1,Con_5Y2,Con_5Y3
Y2,CVB5_5Y3,Con_5Y1,Con_5Y2,Con_5Y3
Y2,CVB5_5Y3,Con_5Y1,Con_5Y2,Con_5Y3
Y2,CVB5_5Y3,Con_5Y1,Con_5Y2,Con_5Y3
Y2,CVB5_5Y3,Con_5Y1,Con_5Y2,Con_5Y3
Y2,CVB5_5Y3,Con_5Y1,Con_5Y2,Con_5Y3
Y2,CVB5_5Y3,Con_5Y1,Con_5Y2,Con_5Y3
Y2,CVB5_5Y3,Con_5Y1,Con_5Y2,Con_5Y3
Y2,CVB5_5Y3,Con_5Y1,Con_5Y2,Con_5Y3
Y2,CVB5_5Y3,Con_5Y1,Con_5Y2,Con_5Y3
Y2,CVB5_5Y3,Con_5Y1,Con_5Y2,Con_5Y3
Y2,CVB5_5Y3,Con_5Y1,Con_5Y2,Con_5Y3
Y2,CVB5_5Y3,Con_5Y1,Con_5Y2,Con_5Y3
Y2,CVB5_5Y3,Con_5Y1,Con_5Y2,Con_5Y3
Y2,CVB5_5Y3,Con_5Y1,Con_5Y2,Con_5Y3
Y2,CVB5_5Y3,Con_5Y1,Con_5Y2,Con_5Y3
Y2,CVB5_5Y3,Con_5Y1,Con_5Y2,Con_5Y3
Y2,CVB5_5Y3,Con_5Y1,Con_5Y2,Con_5Y3
Y2,CVB5_5Y3,Con_5Y1,Con_5Y2,Con_5Y3
Y2,CVB5_5Y3,Con_5Y1,Con_5Y2,Con_5Y3
Y2,CVB5_5Y3,Con_5Y1,Con_5Y2,Con_5Y3
Y2,CVB5_5Y3,Con_5Y1,Con_5Y2,Con_5Y3
Y2,CVB5_5Y3,Con_5Y1,Con_5Y2,Con_5Y3
Y2,CVB5_5Y3,Con_5Y1,Con_5Y2,Con_5Y3
Y2,CVB5_5Y3,Con_5Y1,Con_5Y2,Con_5Y3
Y2,CVB5_5Y3,Con_5Y1,Con_5Y2,Con_5Y3
Y2,CVB5_5Y3,Con_5Y1,Con_5Y2,Con_5Y3
Y2,CVB5_5Y3,Con_5Y1,Con_5Y2,Con_5Y3
Y2,CVB5_5Y3,Con_5Y1,Con_5Y2,Con_5Y3
Y2,CVB5_5Y3,Con_5Y1,Con_5Y2,Con_5Y3
Y2,CVB5_5Y3,Con_5Y1,Con_5Y2,Con_5Y3
Y2,CVB5_5Y3,Con_5Y1,Con_5Y2,Con_5Y3
Y2,CVB5_5Y3,Con_5Y1,Con_5Y2,Con_5Y3
Y2,CVB5_5Y3,Con_5Y1,Con_5Y2,Con_5Y3

Y2,CVB5_5Y3,Con_5Y1,Con_5Y2,Con_5Y3
Y2,CVB5_5Y3,Con_5Y1,Con_5Y2,Con_5Y3
Y2,CVB5_5Y3,Con_5Y1,Con_5Y2,Con_5Y3
Y2,CVB5_5Y3,Con_5Y1,Con_5Y2,Con_5Y3
Y2,CVB5_5Y3,Con_5Y1,Con_5Y2,Con_5Y3
Y2,CVB5_5Y3,Con_5Y1,Con_5Y2,Con_5Y3
Y2,CVB5_5Y3,Con_5Y1,Con_5Y2,Con_5Y3
Y2,CVB5_5Y3,Con_5Y1,Con_5Y2,Con_5Y3
Y2,CVB5_5Y3,Con_5Y1,Con_5Y2,Con_5Y3
Y2,CVB5_5Y3,Con_5Y1,Con_5Y2,Con_5Y3
Y2,CVB5_5Y3,Con_5Y1,Con_5Y2,Con_5Y3
Y2,CVB5_5Y3,Con_5Y1,Con_5Y2,Con_5Y3
Y2,CVB5_5Y3,Con_5Y1,Con_5Y2,Con_5Y3
Y2,CVB5_5Y3,Con_5Y1,Con_5Y2,Con_5Y3
Y2,CVB5_5Y3,Con_5Y1,Con_5Y2,Con_5Y3
Y2,CVB5_5Y3,Con_5Y1,Con_5Y2,Con_5Y3
Y2,CVB5_5Y3,Con_5Y1,Con_5Y2,Con_5Y3
Y2,CVB5_5Y3,Con_5Y1,Con_5Y2,Con_5Y3
Y2,CVB5_5Y3,Con_5Y1,Con_5Y2,Con_5Y3
Y2,CVB5_5Y3,Con_5Y1,Con_5Y2,Con_5Y3
Y2,CVB5_5Y3,Con_5Y1,Con_5Y2,Con_5Y3
Y2,CVB5_5Y3,Con_5Y1,Con_5Y2,Con_5Y3
Y2,CVB5_5Y3,Con_5Y1,Con_5Y2,Con_5Y3
Y2,CVB5_5Y3,Con_5Y1,Con_5Y2,Con_5Y3
Y2,CVB5_5Y3,Con_5Y1,Con_5Y2,Con_5Y3
Y2,CVB5_5Y3,Con_5Y1,Con_5Y2,Con_5Y3
Y2,CVB5_5Y3,Con_5Y1,Con_5Y2,Con_5Y3
Y2,CVB5_5Y3,Con_5Y1,Con_5Y2,Con_5Y3
Y2,CVB5_5Y3,Con_5Y1,Con_5Y2,Con_5Y3
Y2,CVB5_5Y3,Con_5Y1,Con_5Y2,Con_5Y3
Y2,CVB5_5Y3,Con_5Y1,Con_5Y2,Con_5Y3
Y2,CVB5_5Y3,Con_5Y1,Con_5Y2,Con_5Y3
Y2,CVB5_5Y3,Con_5Y1,Con_5Y2,Con_5Y3
Y2,CVB5_5Y3,Con_5Y1,Con_5Y2,Con_5Y3
Y2,CVB5_5Y3,Con_5Y1,Con_5Y3
Y2,CVB5_5Y3,Con_5Y1,Con_5Y3
Y2,CVB5_5Y3,Con_5Y1,Con_5Y3
Y2,CVB5_5Y3,Con_5Y1,Con_5Y3
Y2,CVB5_5Y3,Con_5Y1,Con_5Y3
Y2,CVB5_5Y3,Con_5Y1,Con_5Y3
Y2,CVB5_5Y3,Con_5Y1,Con_5Y3
Y2,CVB5_5Y3,Con_5Y1,Con_5Y3
Y2,CVB5_5Y3,Con_5Y1,Con_5Y3
Y2,CVB5_5Y3,Con_5Y1,Con_5Y3
Y2,CVB5_5Y3,Con_5Y1,Con_5Y3
Y2,CVB5_5Y3,Con_5Y1,Con_5Y3
Y2,CVB5_5Y3,Con_5Y1,Con_5Y3
Y2,CVB5_5Y3,Con_5Y1,Con_5Y3
Y2,CVB5_5Y3,Con_5Y1,Con_5Y3
Y2,CVB5_5Y3,Con_5Y1,Con_5Y3
Y2,CVB5_5Y3,Con_5Y1,Con_5Y3
Y2,CVB5_5Y3,Con_5Y1,Con_5Y3
Y2,CVB5_5Y3,Con_5Y1,Con_5Y3
Y2,CVB5_5Y3,Con_5Y1,Con_5Y3
Y2,CVB5_5Y3,Con_5Y1,Con_5Y3
Y2,CVB5_5Y3,Con_5Y1,Con_5Y3
Y2,CVB5_5Y3,Con_5Y1,Con_5Y3
Y2,CVB5_5Y3,Con_5Y1,Con_5Y3

Y2,CVB5_5Y3,Con_5Y1,Con_5Y3
Y2,CVB5_5Y3,Con_5Y1,Con_5Y3
Y2,CVB5_5Y3,Con_5Y1,Con_5Y3
Y2,CVB5_5Y3,Con_5Y1,Con_5Y3
Y2,CVB5_5Y3,Con_5Y1,Con_5Y3
Y2,CVB5_5Y3,Con_5Y1,Con_5Y3
Y2,CVB5_5Y3,Con_5Y2
Y2,CVB5_5Y3,Con_5Y2
Y2,CVB5_5Y3,Con_5Y2
Y2,CVB5_5Y3,Con_5Y2
Y2,CVB5_5Y3,Con_5Y2
Y2,CVB5_5Y3,Con_5Y2
Y2,CVB5_5Y3,Con_5Y2
Y2,CVB5_5Y3,Con_5Y2
Y2,CVB5_5Y3,Con_5Y2
Y2,CVB5_5Y3,Con_5Y2
Y2,CVB5_5Y3,Con_5Y2
Y2,CVB5_5Y3,Con_5Y2
Y2,CVB5_5Y3,Con_5Y2
Y2,CVB5_5Y3,Con_5Y2
Y2,CVB5_5Y3,Con_5Y2
Y2,CVB5_5Y3,Con_5Y2
Y2,CVB5_5Y3,Con_5Y2
Y2,CVB5_5Y3,Con_5Y2
Y2,CVB5_5Y3,Con_5Y2
Y2,CVB5_5Y3,Con_5Y2
Y2,CVB5_5Y3,Con_5Y2
Y2,CVB5_5Y3,Con_5Y2
Y2,CVB5_5Y3,Con_5Y2
Y2,CVB5_5Y3,Con_5Y2
Y2,CVB5_5Y3,Con_5Y2
Y2,CVB5_5Y3,Con_5Y2
Y2,CVB5_5Y3,Con_5Y2
Y2,CVB5_5Y3,Con_5Y2
Y2,CVB5_5Y3,Con_5Y2
Y2,CVB5_5Y3,Con_5Y2
Y2,CVB5_5Y3,Con_5Y2
Y2,CVB5_5Y3,Con_5Y2
Y2,CVB5_5Y3,Con_5Y2
Y2,CVB5_5Y3,Con_5Y2
Y2,CVB5_5Y3,Con_5Y2,Con_5Y3
Y2,CVB5_5Y3,Con_5Y2,Con_5Y3
Y2,CVB5_5Y3,Con_5Y2,Con_5Y3
Y2,CVB5_5Y3,Con_5Y2,Con_5Y3
Y2,CVB5_5Y3,Con_5Y2,Con_5Y3
Y2,CVB5_5Y3,Con_5Y2,Con_5Y3
Y2,CVB5_5Y3,Con_5Y2,Con_5Y3
Y2,CVB5_5Y3,Con_5Y2,Con_5Y3
Y2,CVB5_5Y3,Con_5Y2,Con_5Y3
Y2,CVB5_5Y3,Con_5Y2,Con_5Y3
Y2,CVB5_5Y3,Con_5Y2,Con_5Y3
Y2,CVB5_5Y3,Con_5Y2,Con_5Y3
Y2,CVB5_5Y3,Con_5Y2,Con_5Y3
Y2,CVB5_5Y3,Con_5Y2,Con_5Y3
Y2,CVB5_5Y3,Con_5Y2,Con_5Y3
Y2,CVB5_5Y3,Con_5Y2,Con_5Y3
Y2,CVB5_5Y3,Con_5Y2,Con_5Y3
Y2,CVB5_5Y3,Con_5Y2,Con_5Y3

Y2,CVB5_5Y3,Con_5Y2,Con_5Y3
Y2,CVB5_5Y3,Con_5Y2,Con_5Y3
Y2,CVB5_5Y3,Con_5Y2,Con_5Y3
Y2,CVB5_5Y3,Con_5Y2,Con_5Y3
Y2,CVB5_5Y3,Con_5Y2,Con_5Y3
Y2,CVB5_5Y3,Con_5Y2,Con_5Y3
Y2,CVB5_5Y3,Con_5Y2,Con_5Y3
Y2,CVB5_5Y3,Con_5Y2,Con_5Y3
Y2,CVB5_5Y3,Con_5Y2,Con_5Y3
Y2,CVB5_5Y3,Con_5Y2,Con_5Y3
Y2,CVB5_5Y3,Con_5Y2,Con_5Y3
Y2,CVB5_5Y3,Con_5Y2,Con_5Y3
Y2,CVB5_5Y3,Con_5Y2,Con_5Y3
Y2,CVB5_5Y3,Con_5Y2,Con_5Y3
Y2,CVB5_5Y3,Con_5Y2,Con_5Y3
Y2,CVB5_5Y3,Con_5Y2,Con_5Y3
Y2,CVB5_5Y3,Con_5Y3
Y2,CVB5_5Y3,Con_5Y3
Y2,CVB5_5Y3,Con_5Y3
Y2,CVB5_5Y3,Con_5Y3
Y2,CVB5_5Y3,Con_5Y3
Y2,CVB5_5Y3,Con_5Y3
Y2,CVB5_5Y3,Con_5Y3
Y2,CVB5_5Y3,Con_5Y3
Y2,CVB5_5Y3,Con_5Y3
Y2,CVB5_5Y3,Con_5Y3
Y3
Y3
Y3
Y3
Y3
Y3
Y3
Y3
Y3
Y3
Y3
Y3
Y3
Y3
Y3
Y3
Y3
Y3
Y3
Y3
Y3
Y3
Y3
Y3
Y3
Y3
Y3
Y3
Y3
Y3
Y3
Y3

Y3
Y3
Y3,Con_5Y1
Y3,Con_5Y1
Y3,Con_5Y1
Y3,Con_5Y1
Y3,Con_5Y1
Y3,Con_5Y1
Y3,Con_5Y1
Y3,Con_5Y1
Y3,Con_5Y1
Y3,Con_5Y1
Y3,Con_5Y1
Y3,Con_5Y1
Y3,Con_5Y1
Y3,Con_5Y1
Y3,Con_5Y1
Y3,Con_5Y1
Y3,Con_5Y1
Y3,Con_5Y1
Y3,Con_5Y1,Con_5Y2
Y3,Con_5Y1,Con_5Y2
Y3,Con_5Y1,Con_5Y2
Y3,Con_5Y1,Con_5Y2
Y3,Con_5Y1,Con_5Y2
Y3,Con_5Y1,Con_5Y2
Y3,Con_5Y1,Con_5Y2
Y3,Con_5Y1,Con_5Y2
Y3,Con_5Y1,Con_5Y2
Y3,Con_5Y1,Con_5Y2
Y3,Con_5Y1,Con_5Y2
Y3,Con_5Y1,Con_5Y2
Y3,Con_5Y1,Con_5Y2
Y3,Con_5Y1,Con_5Y2
Y3,Con_5Y1,Con_5Y2
Y3,Con_5Y1,Con_5Y2
Y3,Con_5Y1,Con_5Y2
Y3,Con_5Y1,Con_5Y2
Y3,Con_5Y1,Con_5Y2
Y3,Con_5Y1,Con_5Y2
Y3,Con_5Y1,Con_5Y2
Y3,Con_5Y1,Con_5Y2
Y3,Con_5Y1,Con_5Y2
Y3,Con_5Y1,Con_5Y2
Y3,Con_5Y1,Con_5Y2
Y3,Con_5Y1,Con_5Y2
Y3,Con_5Y1,Con_5Y2
Y3,Con_5Y1,Con_5Y2
Y3,Con_5Y1,Con_5Y2
Y3,Con_5Y1,Con_5Y2
Y3,Con_5Y1,Con_5Y2
Y3,Con_5Y1,Con_5Y2
Y3,Con_5Y1,Con_5Y2
Y3,Con_5Y1,Con_5Y2,Con_5Y3
Y3,Con_5Y1,Con_5Y2,Con_5Y3
Y3,Con_5Y1,Con_5Y2,Con_5Y3

Y3,Con_5Y1,Con_5Y2,Con_5Y3
Y3,Con_5Y1,Con_5Y2,Con_5Y3
Y3,Con_5Y1,Con_5Y2,Con_5Y3
Y3,Con_5Y1,Con_5Y2,Con_5Y3
Y3,Con_5Y1,Con_5Y2,Con_5Y3
Y3,Con_5Y1,Con_5Y2,Con_5Y3
Y3,Con_5Y1,Con_5Y2,Con_5Y3
Y3,Con_5Y1,Con_5Y2,Con_5Y3
Y3,Con_5Y1,Con_5Y2,Con_5Y3
Y3,Con_5Y1,Con_5Y2,Con_5Y3
Y3,Con_5Y1,Con_5Y2,Con_5Y3
Y3,Con_5Y1,Con_5Y2,Con_5Y3
Y3,Con_5Y1,Con_5Y2,Con_5Y3
Y3,Con_5Y1,Con_5Y2,Con_5Y3
Y3,Con_5Y1,Con_5Y2,Con_5Y3
Y3,Con_5Y1,Con_5Y2,Con_5Y3
Y3,Con_5Y1,Con_5Y2,Con_5Y3
Y3,Con_5Y1,Con_5Y2,Con_5Y3
Y3,Con_5Y1,Con_5Y2,Con_5Y3
Y3,Con_5Y1,Con_5Y2,Con_5Y3
Y3,Con_5Y1,Con_5Y2,Con_5Y3
Y3,Con_5Y1,Con_5Y2,Con_5Y3
Y3,Con_5Y1,Con_5Y2,Con_5Y3
Y3,Con_5Y1,Con_5Y2,Con_5Y3
Y3,Con_5Y1,Con_5Y2,Con_5Y3
Y3,Con_5Y1,Con_5Y2,Con_5Y3
Y3,Con_5Y1,Con_5Y2,Con_5Y3
Y3,Con_5Y1,Con_5Y2,Con_5Y3
Y3,Con_5Y1,Con_5Y2,Con_5Y3
Y3,Con_5Y1,Con_5Y2,Con_5Y3
Y3,Con_5Y1,Con_5Y2,Con_5Y3
Y3,Con_5Y1,Con_5Y2,Con_5Y3
Y3,Con_5Y1,Con_5Y2,Con_5Y3
Y3,Con_5Y1,Con_5Y2,Con_5Y3
Y3,Con_5Y1,Con_5Y2,Con_5Y3
Y3,Con_5Y1,Con_5Y2,Con_5Y3
Y3,Con_5Y1,Con_5Y2,Con_5Y3
Y3,Con_5Y1,Con_5Y2,Con_5Y3
Y3,Con_5Y1,Con_5Y2,Con_5Y3
Y3,Con_5Y1,Con_5Y2,Con_5Y3
Y3,Con_5Y1,Con_5Y2,Con_5Y3
Y3,Con_5Y1,Con_5Y2,Con_5Y3
Y3,Con_5Y1,Con_5Y2,Con_5Y3
Y3,Con_5Y1,Con_5Y2,Con_5Y3
Y3,Con_5Y1,Con_5Y2,Con_5Y3
Y3,Con_5Y1,Con_5Y2,Con_5Y3
Y3,Con_5Y1,Con_5Y2,Con_5Y3
Y3,Con_5Y1,Con_5Y2,Con_5Y3
Y3,Con_5Y1,Con_5Y2,Con_5Y3
Y3,Con_5Y1,Con_5Y2,Con_5Y3
Y3,Con_5Y1,Con_5Y2,Con_5Y3
Y3,Con_5Y1,Con_5Y2,Con_5Y3
Y3,Con_5Y1,Con_5Y2,Con_5Y3
Y3,Con_5Y1,Con_5Y3
Y3,Con_5Y1,Con_5Y3
Y3,Con_5Y1,Con_5Y3
Y3,Con_5Y1,Con_5Y3
Y3,Con_5Y1,Con_5Y3

Y3,Con_5Y1,Con_5Y3
Y3,Con_5Y1,Con_5Y3
Y3,Con_5Y1,Con_5Y3
Y3,Con_5Y1,Con_5Y3
Y3,Con_5Y1,Con_5Y3
Y3,Con_5Y1,Con_5Y3
Y3,Con_5Y1,Con_5Y3
Y3,Con_5Y1,Con_5Y3
Y3,Con_5Y1,Con_5Y3
Y3,Con_5Y1,Con_5Y3
Y3,Con_5Y2
Y3,Con_5Y2
Y3,Con_5Y2
Y3,Con_5Y2
Y3,Con_5Y2
Y3,Con_5Y2
Y3,Con_5Y2
Y3,Con_5Y2
Y3,Con_5Y2
Y3,Con_5Y2
Y3,Con_5Y2
Y3,Con_5Y2
Y3,Con_5Y2
Y3,Con_5Y2
Y3,Con_5Y2
Y3,Con_5Y2
Y3,Con_5Y2
Y3,Con_5Y2
Y3,Con_5Y2
Y3,Con_5Y2
Y3,Con_5Y2
Y3,Con_5Y2
Y3,Con_5Y2
Y3,Con_5Y2
Y3,Con_5Y2
Y3,Con_5Y2
Y3,Con_5Y2
Y3,Con_5Y2
Y3,Con_5Y2
Y3,Con_5Y2,Con_5Y3
Y3,Con_5Y2,Con_5Y3
Y3,Con_5Y2,Con_5Y3
Y3,Con_5Y2,Con_5Y3
Y3,Con_5Y2,Con_5Y3
Y3,Con_5Y2,Con_5Y3
Y3,Con_5Y2,Con_5Y3
Y3,Con_5Y2,Con_5Y3
Y3,Con_5Y2,Con_5Y3
Y3,Con_5Y2,Con_5Y3
Y3,Con_5Y2,Con_5Y3
Y3,Con_5Y2,Con_5Y3
Y3,Con_5Y2,Con_5Y3
Y3,Con_5Y2,Con_5Y3
Y3,Con_5Y2,Con_5Y3
Y3,Con_5Y2,Con_5Y3
Y3,Con_5Y2,Con_5Y3
Y3,Con_5Y3
Y3,Con_5Y3

'3,Con_5Y3
'3,Con_5Y3
'3,Con_5Y3
'3,Con_5Y3
'3,Con_5Y3
'3,Con_5Y3
'3,Con_5Y3
'3,Con_5Y3
'3,Con_5Y3
'3,Con_5Y3
'3,Con_5Y3
'3,Con_5Y3
'3,Con_5Y3
'3,Con_5Y3
'3,Con_5Y3
'3,Con_5Y3

,Con_5Y2
,Con_5Y2
,Con_5Y2
,Con_5Y2
,Con_5Y2
,Con_5Y2
,Con_5Y2
,Con_5Y2
,Con_5Y2
,Con_5Y2
,Con_5Y2
,Con_5Y2
,Con_5Y2
,Con_5Y2
,Con_5Y2
,Con_5Y2
,Con_5Y2
,Con_5Y2
,Con_5Y2
,Con_5Y2
,Con_5Y2
,Con_5Y2
,Con_5Y2
,Con_5Y2
,Con_5Y2
,Con_5Y2
,Con_5Y2
,Con_5Y2
,Con_5Y2
,Con_5Y2
,Con_5Y2
,Con_5Y2
,Con_5Y2
,Con_5Y2
,Con_5Y2
,Con_5Y2
,Con_5Y2
,Con_5Y2
,Con_5Y2
,Con_5Y2
,Con_5Y2
,Con_5Y2
,Con_5Y2
,Con_5Y2

,Con_5Y2
,Con_5Y2
,Con_5Y2
,Con_5Y2
,Con_5Y2
,Con_5Y2
,Con_5Y2
,Con_5Y2
,Con_5Y2
,Con_5Y2
,Con_5Y2
,Con_5Y2
,Con_5Y2
,Con_5Y2
,Con_5Y2
,Con_5Y2
,Con_5Y2
,Con_5Y2,Con_5Y3
,Con_5Y2,Con_5Y3
,Con_5Y2,Con_5Y3
,Con_5Y2,Con_5Y3
,Con_5Y2,Con_5Y3
,Con_5Y2,Con_5Y3
,Con_5Y2,Con_5Y3
,Con_5Y2,Con_5Y3
,Con_5Y2,Con_5Y3
,Con_5Y2,Con_5Y3
,Con_5Y2,Con_5Y3
,Con_5Y2,Con_5Y3
,Con_5Y2,Con_5Y3
,Con_5Y2,Con_5Y3
,Con_5Y2,Con_5Y3
,Con_5Y2,Con_5Y3
,Con_5Y2,Con_5Y3
,Con_5Y2,Con_5Y3
,Con_5Y2,Con_5Y3
,Con_5Y2,Con_5Y3
,Con_5Y2,Con_5Y3
,Con_5Y2,Con_5Y3
,Con_5Y2,Con_5Y3
,Con_5Y2,Con_5Y3
,Con_5Y2,Con_5Y3
,Con_5Y2,Con_5Y3
,Con_5Y2,Con_5Y3
,Con_5Y2,Con_5Y3
,Con_5Y2,Con_5Y3
,Con_5Y2,Con_5Y3
,Con_5Y2,Con_5Y3
,Con_5Y2,Con_5Y3
,Con_5Y2,Con_5Y3
,Con_5Y2,Con_5Y3
,Con_5Y2,Con_5Y3
,Con_5Y2,Con_5Y3
,Con_5Y2,Con_5Y3
,Con_5Y2,Con_5Y3
,Con_5Y2,Con_5Y3
,Con_5Y2,Con_5Y3

,Con_5Y2,Con_5Y3
,Con_5Y2,Con_5Y3
,Con_5Y2,Con_5Y3
,Con_5Y2,Con_5Y3
,Con_5Y2,Con_5Y3
,Con_5Y2,Con_5Y3
,Con_5Y2,Con_5Y3
,Con_5Y3
,Con_5Y3
,Con_5Y3
,Con_5Y3
,Con_5Y3
,Con_5Y3
,Con_5Y3
,Con_5Y3
,Con_5Y3
,Con_5Y3
,Con_5Y3
,Con_5Y3
,Con_5Y3
,Con_5Y3
,Con_5Y3
,Con_5Y3
,Con_5Y3
,Con_5Y3
,Con_5Y3
,Con_5Y3
,Con_5Y3
,Con_5Y3
,Con_5Y3
,Con_5Y3
,Con_5Y3
,Con_5Y3
,Con_5Y3
,Con_5Y3
,Con_5Y3
,Con_5Y3

,Con_5Y3
,Con_5Y3
,Con_5Y3
,Con_5Y3
,Con_5Y3
,Con_5Y3
,Con_5Y3
,Con_5Y3
,Con_5Y3
,Con_5Y3
,Con_5Y3
,Con_5Y3
,Con_5Y3
,Con_5Y3
,Con_5Y3
,Con_5Y3
,Con_5Y3
,Con_5Y3
,Con_5Y3
,Con_5Y3
,Con_5Y3
,Con_5Y3
,Con_5Y3
,Con_5Y3
,Con_5Y3
,Con_5Y3
,Con_5Y3
,Con_5Y3
,Con_5Y3
,Con_5Y3
,Con_5Y3
,Con_5Y3
,Con_5Y3
,Con_5Y3
,Con_5Y3
,Con_5Y3
,Con_5Y3

,Con_5Y3

'Y3

Y3
Y3
Y3
Y3
Y3
Y3
Y3
Y3
Y3
Y3
Y3
Y3
Y3
Y3
Y3
Y3
Y3
Y3
Y3
Y3
Y3
Y3
Y3
Y3
Y3
Y3
Y3
Y3
Y3
Y3
Y3
Y3
Y3
Y3
Y3
Y3
Y3
Y3
Y3
Y3
Y3
Y3
Y3
Y3
Y3
Y3
Y3
Y3
Y3
Y3
Y3
Y3
Y3
Y3
Y3
Y3
Y3,Con_5Y1
Y3,Con_5Y1

Y3,Con_5Y1
Y3,Con_5Y1
Y3,Con_5Y1
Y3,Con_5Y1
Y3,Con_5Y1
Y3,Con_5Y1
Y3,Con_5Y1
Y3,Con_5Y1
Y3,Con_5Y1
Y3,Con_5Y1
Y3,Con_5Y1
Y3,Con_5Y1
Y3,Con_5Y1
Y3,Con_5Y1
Y3,Con_5Y1
Y3,Con_5Y1
Y3,Con_5Y1
Y3,Con_5Y1
Y3,Con_5Y1
Y3,Con_5Y1
Y3,Con_5Y1
Y3,Con_5Y1
Y3,Con_5Y1
Y3,Con_5Y1
Y3,Con_5Y1
Y3,Con_5Y1
Y3,Con_5Y1
Y3,Con_5Y1
Y3,Con_5Y1
Y3,Con_5Y1
Y3,Con_5Y1
Y3,Con_5Y1
Y3,Con_5Y1
Y3,Con_5Y1,Con_5Y2
Y3,Con_5Y1,Con_5Y2
Y3,Con_5Y1,Con_5Y2
Y3,Con_5Y1,Con_5Y2
Y3,Con_5Y1,Con_5Y2
Y3,Con_5Y1,Con_5Y2
Y3,Con_5Y1,Con_5Y2
Y3,Con_5Y1,Con_5Y2
Y3,Con_5Y1,Con_5Y2
Y3,Con_5Y1,Con_5Y2
Y3,Con_5Y1,Con_5Y2
Y3,Con_5Y1,Con_5Y2
Y3,Con_5Y1,Con_5Y2
Y3,Con_5Y1,Con_5Y2
Y3,Con_5Y1,Con_5Y2
Y3,Con_5Y1,Con_5Y2
Y3,Con_5Y1,Con_5Y2
Y3,Con_5Y1,Con_5Y2
Y3,Con_5Y1,Con_5Y2
Y3,Con_5Y1,Con_5Y2
Y3,Con_5Y1,Con_5Y2
Y3,Con_5Y1,Con_5Y2
Y3,Con_5Y1,Con_5Y2
Y3,Con_5Y1,Con_5Y2
Y3,Con_5Y1,Con_5Y2

Y3,Con_5Y1,Con_5Y2
Y3,Con_5Y1,Con_5Y2
Y3,Con_5Y1,Con_5Y2
Y3,Con_5Y1,Con_5Y2
Y3,Con_5Y1,Con_5Y2
Y3,Con_5Y1,Con_5Y2
Y3,Con_5Y1,Con_5Y2
Y3,Con_5Y1,Con_5Y2
Y3,Con_5Y1,Con_5Y2
Y3,Con_5Y1,Con_5Y2
Y3,Con_5Y1,Con_5Y2
Y3,Con_5Y1,Con_5Y2
Y3,Con_5Y1,Con_5Y2
Y3,Con_5Y1,Con_5Y2
Y3,Con_5Y1,Con_5Y2
Y3,Con_5Y1,Con_5Y2
Y3,Con_5Y1,Con_5Y2
Y3,Con_5Y1,Con_5Y2
Y3,Con_5Y1,Con_5Y2,Con_5Y3
Y3,Con_5Y1,Con_5Y2,Con_5Y3
Y3,Con_5Y1,Con_5Y2,Con_5Y3
Y3,Con_5Y1,Con_5Y2,Con_5Y3
Y3,Con_5Y1,Con_5Y2,Con_5Y3
Y3,Con_5Y1,Con_5Y2,Con_5Y3
Y3,Con_5Y1,Con_5Y2,Con_5Y3
Y3,Con_5Y1,Con_5Y2,Con_5Y3
Y3,Con_5Y1,Con_5Y2,Con_5Y3
Y3,Con_5Y1,Con_5Y2,Con_5Y3
Y3,Con_5Y1,Con_5Y2,Con_5Y3
Y3,Con_5Y1,Con_5Y2,Con_5Y3
Y3,Con_5Y1,Con_5Y2,Con_5Y3
Y3,Con_5Y1,Con_5Y2,Con_5Y3
Y3,Con_5Y1,Con_5Y2,Con_5Y3
Y3,Con_5Y1,Con_5Y2,Con_5Y3
Y3,Con_5Y1,Con_5Y2,Con_5Y3
Y3,Con_5Y1,Con_5Y2,Con_5Y3
Y3,Con_5Y1,Con_5Y2,Con_5Y3
Y3,Con_5Y1,Con_5Y2,Con_5Y3
Y3,Con_5Y1,Con_5Y2,Con_5Y3
Y3,Con_5Y1,Con_5Y2,Con_5Y3
Y3,Con_5Y1,Con_5Y2,Con_5Y3
Y3,Con_5Y1,Con_5Y2,Con_5Y3
Y3,Con_5Y1,Con_5Y2,Con_5Y3
Y3,Con_5Y1,Con_5Y2,Con_5Y3
Y3,Con_5Y1,Con_5Y2,Con_5Y3
Y3,Con_5Y1,Con_5Y2,Con_5Y3
Y3,Con_5Y1,Con_5Y2,Con_5Y3
Y3,Con_5Y1,Con_5Y2,Con_5Y3
Y3,Con_5Y1,Con_5Y2,Con_5Y3
Y3,Con_5Y1,Con_5Y2,Con_5Y3
Y3,Con_5Y1,Con_5Y2,Con_5Y3
Y3,Con_5Y1,Con_5Y2,Con_5Y3
Y3,Con_5Y1,Con_5Y2,Con_5Y3
Y3,Con_5Y1,Con_5Y2,Con_5Y3
Y3,Con_5Y1,Con_5Y2,Con_5Y3
Y3,Con_5Y1,Con_5Y2,Con_5Y3
Y3,Con_5Y1,Con_5Y2,Con_5Y3
Y3,Con_5Y1,Con_5Y2,Con_5Y3

Y3,Con_5Y1,Con_5Y2,Con_5Y3
Y3,Con_5Y1,Con_5Y2,Con_5Y3
Y3,Con_5Y1,Con_5Y2,Con_5Y3
Y3,Con_5Y1,Con_5Y2,Con_5Y3
Y3,Con_5Y1,Con_5Y2,Con_5Y3
Y3,Con_5Y1,Con_5Y2,Con_5Y3
Y3,Con_5Y1,Con_5Y2,Con_5Y3
Y3,Con_5Y1,Con_5Y2,Con_5Y3
Y3,Con_5Y1,Con_5Y2,Con_5Y3
Y3,Con_5Y1,Con_5Y2,Con_5Y3
Y3,Con_5Y1,Con_5Y2,Con_5Y3
Y3,Con_5Y1,Con_5Y2,Con_5Y3
Y3,Con_5Y1,Con_5Y2,Con_5Y3
Y3,Con_5Y1,Con_5Y2,Con_5Y3
Y3,Con_5Y1,Con_5Y2,Con_5Y3
Y3,Con_5Y1,Con_5Y2,Con_5Y3
Y3,Con_5Y1,Con_5Y2,Con_5Y3
Y3,Con_5Y1,Con_5Y2,Con_5Y3
Y3,Con_5Y1,Con_5Y2,Con_5Y3
Y3,Con_5Y1,Con_5Y2,Con_5Y3
Y3,Con_5Y1,Con_5Y2,Con_5Y3
Y3,Con_5Y1,Con_5Y2,Con_5Y3
Y3,Con_5Y1,Con_5Y2,Con_5Y3
Y3,Con_5Y1,Con_5Y2,Con_5Y3
Y3,Con_5Y1,Con_5Y2,Con_5Y3
Y3,Con_5Y1,Con_5Y2,Con_5Y3
Y3,Con_5Y1,Con_5Y2,Con_5Y3
Y3,Con_5Y1,Con_5Y2,Con_5Y3
Y3,Con_5Y1,Con_5Y2,Con_5Y3
Y3,Con_5Y1,Con_5Y2,Con_5Y3
Y3,Con_5Y1,Con_5Y2,Con_5Y3
Y3,Con_5Y1,Con_5Y2,Con_5Y3
Y3,Con_5Y1,Con_5Y2,Con_5Y3
Y3,Con_5Y1,Con_5Y2,Con_5Y3
Y3,Con_5Y1,Con_5Y2,Con_5Y3
Y3,Con_5Y1,Con_5Y3
Y3,Con_5Y1,Con_5Y3
Y3,Con_5Y1,Con_5Y3
Y3,Con_5Y1,Con_5Y3
Y3,Con_5Y1,Con_5Y3
Y3,Con_5Y1,Con_5Y3
Y3,Con_5Y1,Con_5Y3
Y3,Con_5Y1,Con_5Y3
Y3,Con_5Y1,Con_5Y3
Y3,Con_5Y1,Con_5Y3
Y3,Con_5Y1,Con_5Y3
Y3,Con_5Y1,Con_5Y3
Y3,Con_5Y1,Con_5Y3
Y3,Con_5Y1,Con_5Y3
Y3,Con_5Y1,Con_5Y3
Y3,Con_5Y2
Y3,Con_5Y2
Y3,Con_5Y2
Y3,Con_5Y2
Y3,Con_5Y2
Y3,Con_5Y2
Y3,Con_5Y2
Y3,Con_5Y2

Y3,Con_5Y2
Y3,Con_5Y2
Y3,Con_5Y2
Y3,Con_5Y2
Y3,Con_5Y2
Y3,Con_5Y2
Y3,Con_5Y2
Y3,Con_5Y2
Y3,Con_5Y2
Y3,Con_5Y2
Y3,Con_5Y2
Y3,Con_5Y2
Y3,Con_5Y2
Y3,Con_5Y2
Y3,Con_5Y2
Y3,Con_5Y2
Y3,Con_5Y2
Y3,Con_5Y2
Y3,Con_5Y2
Y3,Con_5Y2
Y3,Con_5Y2,Con_5Y3
Y3,Con_5Y2,Con_5Y3
Y3,Con_5Y2,Con_5Y3
Y3,Con_5Y2,Con_5Y3
Y3,Con_5Y2,Con_5Y3
Y3,Con_5Y2,Con_5Y3
Y3,Con_5Y2,Con_5Y3
Y3,Con_5Y2,Con_5Y3
Y3,Con_5Y2,Con_5Y3
Y3,Con_5Y2,Con_5Y3
Y3,Con_5Y2,Con_5Y3
Y3,Con_5Y2,Con_5Y3
Y3,Con_5Y2,Con_5Y3
Y3,Con_5Y2,Con_5Y3
Y3,Con_5Y2,Con_5Y3
Y3,Con_5Y2,Con_5Y3
Y3,Con_5Y2,Con_5Y3
Y3,Con_5Y2,Con_5Y3
Y3,Con_5Y2,Con_5Y3
Y3,Con_5Y2,Con_5Y3
Y3,Con_5Y2,Con_5Y3
Y3,Con_5Y2,Con_5Y3
Y3,Con_5Y2,Con_5Y3
Y3,Con_5Y2,Con_5Y3
Y3,Con_5Y2,Con_5Y3
Y3,Con_5Y2,Con_5Y3
Y3,Con_5Y2,Con_5Y3
Y3,Con_5Y2,Con_5Y3
Y3,Con_5Y2,Con_5Y3
Y3,Con_5Y2,Con_5Y3
Y3,Con_5Y2,Con_5Y3
Y3,Con_5Y2,Con_5Y3
Y3,Con_5Y3
Y3,Con_5Y3
Y3,Con_5Y3
Y3,Con_5Y3
Y3,Con_5Y3
Y3,Con_5Y3

Y3,Con_5Y3
Y3,Con_5Y3
Y3,Con_5Y3
Y3,Con_5Y3
Y3,Con_5Y3
Y3,Con_5Y3
Y3,Con_5Y3
Y3,Con_5Y3
Y3,Con_5Y3
Y3,Con_5Y3

,Con_5Y2
,Con_5Y2
,Con_5Y2
,Con_5Y2
,Con_5Y2
,Con_5Y2
,Con_5Y2
,Con_5Y2
,Con_5Y2
,Con_5Y2
,Con_5Y2
,Con_5Y2
,Con_5Y2
,Con_5Y2
,Con_5Y2
,Con_5Y2
,Con_5Y2
,Con_5Y2

,Con_5Y2
,Con_5Y2
,Con_5Y2
,Con_5Y2
,Con_5Y2
,Con_5Y2
,Con_5Y2
,Con_5Y2
,Con_5Y2
,Con_5Y2
,Con_5Y2
,Con_5Y2
,Con_5Y2
,Con_5Y2
,Con_5Y2
,Con_5Y2
,Con_5Y2
,Con_5Y2
,Con_5Y2
,Con_5Y2
,Con_5Y2
,Con_5Y2
,Con_5Y2
,Con_5Y2
,Con_5Y2
,Con_5Y2
,Con_5Y2,Con_5Y3
,Con_5Y2,Con_5Y3
,Con_5Y2,Con_5Y3
,Con_5Y2,Con_5Y3
,Con_5Y2,Con_5Y3
,Con_5Y2,Con_5Y3
,Con_5Y2,Con_5Y3
,Con_5Y2,Con_5Y3
,Con_5Y2,Con_5Y3
,Con_5Y2,Con_5Y3
,Con_5Y2,Con_5Y3
,Con_5Y2,Con_5Y3
,Con_5Y2,Con_5Y3
,Con_5Y2,Con_5Y3
,Con_5Y2,Con_5Y3
,Con_5Y2,Con_5Y3
,Con_5Y2,Con_5Y3
,Con_5Y2,Con_5Y3
,Con_5Y2,Con_5Y3
,Con_5Y2,Con_5Y3
,Con_5Y2,Con_5Y3
,Con_5Y2,Con_5Y3
,Con_5Y2,Con_5Y3
,Con_5Y2,Con_5Y3
,Con_5Y2,Con_5Y3
,Con_5Y2,Con_5Y3
,Con_5Y2,Con_5Y3
,Con_5Y2,Con_5Y3
,Con_5Y2,Con_5Y3
,Con_5Y2,Con_5Y3
,Con_5Y2,Con_5Y3
,Con_5Y2,Con_5Y3

,Con_5Y2,Con_5Y3
,Con_5Y2,Con_5Y3
,Con_5Y2,Con_5Y3
,Con_5Y2,Con_5Y3
,Con_5Y2,Con_5Y3
,Con_5Y2,Con_5Y3
,Con_5Y2,Con_5Y3
,Con_5Y2,Con_5Y3
,Con_5Y2,Con_5Y3
,Con_5Y3
,Con_5Y3
,Con_5Y3
,Con_5Y3
,Con_5Y3
,Con_5Y3
,Con_5Y3
,Con_5Y3
,Con_5Y3
,Con_5Y3
,Con_5Y3
,Con_5Y3
,Con_5Y3
,Con_5Y3
,Con_5Y3
,Con_5Y3
,Con_5Y3
,Con_5Y3
,Con_5Y3
,Con_5Y3
,Con_5Y3
,Con_5Y3
,Con_5Y3
,Con_5Y3

,Con_5Y3
,Con_5Y3
,Con_5Y3
,Con_5Y3
,Con_5Y3
,Con_5Y3
,Con_5Y3
,Con_5Y3
,Con_5Y3
,Con_5Y3
,Con_5Y3
,Con_5Y3
,Con_5Y3
,Con_5Y3
,Con_5Y3
,Con_5Y3
,Con_5Y3
,Con_5Y3
,Con_5Y3
,Con_5Y3
,Con_5Y3
,Con_5Y3
,Con_5Y3
,Con_5Y3
,Con_5Y3
,Con_5Y3
,Con_5Y3
,Con_5Y3
,Con_5Y3
,Con_5Y3
,Con_5Y3
,Con_5Y3
,Con_5Y3
