# Supplementary material for: Expression Profiles of Differentially Expressed Circular RNAs and circRNA–miRNA–mRNA Regulatory Networks in SH-SY5Y Cells Infected with Coxsackievirus B5
Source: Int J Genomics. 2022 Oct 10;2022:9298149. doi: 10.1155/2022/9298149 (PMC9577011; doi:10.1155/2022/9298149)
Supplement: Supplementary 2 — Supplementary Table 2 Feature of the identified circRNA genome. [file 9298149.f2.pdf]

Table S2. Feature of identified circRNA genomic

Feature	Sample	Num
exon	CVB5_5Y1	7274
intron	CVB5_5Y1	187
intergenic	CVB5_5Y1	100
exon	CVB5_5Y2	7936
intron	CVB5_5Y2	234
intergenic	CVB5_5Y2	110
exon	CVB5_5Y3	7993
intron	CVB5_5Y3	201
intergenic	CVB5_5Y3	98
exon	Con_5Y1	9604
intron	Con_5Y1	267
intergenic	Con_5Y1	127
exon	Con_5Y2	10492
intron	Con_5Y2	322
intergenic	Con_5Y2	136
exon	Con_5Y3	8111
intron	Con_5Y3	203
intergenic	Con_5Y3	111
