# Supplementary material for: Expression Profiles of Differentially Expressed Circular RNAs and circRNA–miRNA–mRNA Regulatory Networks in SH-SY5Y Cells Infected with Coxsackievirus B5
Source: Int J Genomics. 2022 Oct 10;2022:9298149. doi: 10.1155/2022/9298149 (PMC9577011; doi:10.1155/2022/9298149)
Supplement: Supplementary 3 — Supplementary Table 3 Summary of sequencing data quality. [file 9298149.f3.pdf]

Table S3. Summary of sequencing data quality

**Sample_name**	**Raw_reads**	**Clean_reads**	**Raw_bases(G)**	**Clean_bases(G)**	**Error rate(%)**	**Q20(%)**	**Q30(%)**	**GC_content(%)**
Con_5Y1	104866878	102846726	15.73	15.43	0.03	97.78	93.73	51.71
Con_5Y2	127653032	125404704	19.15	18.81	0.03	97.81	93.78	51.02
Con_5Y3	83004818	81340414	12.45	12.2	0.03	97.9	93.97	51.04
CVB5_5Y1	89868976	87419108	13.48	13.11	0.03	97.77	93.75	53.48
CVB5_5Y2	100094218	97465572	15.01	14.62	0.03	97.99	94.23	52.96
CVB5_5Y3	99830494	97972780	14.97	14.7	0.03	97.9	94.02	52.25
