# Supplementary material for: Expression Profiles of Differentially Expressed Circular RNAs and circRNA–miRNA–mRNA Regulatory Networks in SH-SY5Y Cells Infected with Coxsackievirus B5
Source: Int J Genomics. 2022 Oct 10;2022:9298149. doi: 10.1155/2022/9298149 (PMC9577011; doi:10.1155/2022/9298149)
Supplement: Supplementary 4 — Supplementary Table 4 Distribution of TPM values of circRNAs. [file 9298149.f4.pdf]

Table S4. The distribution of TPM values of circRNAs

TPM Interval	CVB5_5Y1.tpm	CVB5_5Y2.tpm	CVB5_5Y3.tpm	Con_5Y1.tpm	Con_5Y2.tpm	Con_5Y3.tpm
0-0.1	7697(69.21%)	7352(66.11%)	7401(66.55%)	6586(59.22%)	6102(54.87%)	7303(65.67%)
0.1-0.3	0(0.00%)	0(0.00%)	0(0.00%)	0(0.00%)	0(0.00%)	0(0.00%)
0.3-3.57	0(0.00%)	0(0.00%)	0(0.00%)	0(0.00%)	0(0.00%)	0(0.00%)
3.57-15	0(0.00%)	0(0.00%)	0(0.00%)	0(0.00%)	0(0.00%)	0(0.00%)
15-60	0(0.00%)	0(0.00%)	0(0.00%)	0(0.00%)	1219(10.96%)	0(0.00%)
>60	3424(30.79%)	3769(33.89%)	3720(33.45%)	4535(40.78%)	3800(34.17%)	3818(34.33%)
