# Supplementary material for: Expression Profiles of Differentially Expressed Circular RNAs and circRNA–miRNA–mRNA Regulatory Networks in SH-SY5Y Cells Infected with Coxsackievirus B5
Source: Int J Genomics. 2022 Oct 10;2022:9298149. doi: 10.1155/2022/9298149 (PMC9577011; doi:10.1155/2022/9298149)
Supplement: Supplementary 5 — Supplementary Table 5 miRNA targets of circRNAs. [file 9298149.f5.pdf]

Table S5. miRNA targets of circRNA

**miRNA**	**circRNA**
hsa-miR-124-3p	novel_circ_0014617
hsa-miR-1254	novel_circ_0014617
hsa-miR-1270	novel_circ_0014617
hsa-miR-1285-5p	novel_circ_0014617
hsa-miR-1288-3p	novel_circ_0014617
hsa-miR-1298-5p	novel_circ_0014617
hsa-miR-138-2-3p	novel_circ_0014617
hsa-miR-138-5p	novel_circ_0014617
hsa-miR-2110	novel_circ_0014617
hsa-miR-2115-5p	novel_circ_0014617
hsa-miR-2681-5p	novel_circ_0014617
hsa-miR-29a-5p	novel_circ_0014617
hsa-miR-302b-5p	novel_circ_0014617
hsa-miR-302d-5p	novel_circ_0014617
hsa-miR-3116	novel_circ_0014617
hsa-miR-3150a-3p	novel_circ_0014617
hsa-miR-3169	novel_circ_0014617
hsa-miR-320e	novel_circ_0014617
hsa-miR-3690	novel_circ_0014617
hsa-miR-382-3p	novel_circ_0014617
hsa-miR-4254	novel_circ_0014617
hsa-miR-4299	novel_circ_0014617
hsa-miR-4326	novel_circ_0014617
hsa-miR-4423-5p	novel_circ_0014617
hsa-miR-4434	novel_circ_0014617
hsa-miR-4516	novel_circ_0014617
hsa-miR-4535	novel_circ_0014617
hsa-miR-4639-5p	novel_circ_0014617
hsa-miR-4667-5p	novel_circ_0014617
hsa-miR-4700-5p	novel_circ_0014617
hsa-miR-4720-5p	novel_circ_0014617
hsa-miR-4762-3p	novel_circ_0014617
hsa-miR-4768-3p	novel_circ_0014617
hsa-miR-4799-3p	novel_circ_0014617
hsa-miR-5006-5p	novel_circ_0014617
hsa-miR-5089-5p	novel_circ_0014617
hsa-miR-509-3p	novel_circ_0014617
hsa-miR-5100	novel_circ_0014617
hsa-miR-512-5p	novel_circ_0014617
hsa-miR-518c-5p	novel_circ_0014617
hsa-miR-550b-3p	novel_circ_0014617
hsa-miR-5588-5p	novel_circ_0014617
hsa-miR-5689	novel_circ_0014617
hsa-miR-5703	novel_circ_0014617
hsa-miR-605-3p	novel_circ_0014617
hsa-miR-6079	novel_circ_0014617
hsa-miR-619-5p	novel_circ_0014617
hsa-miR-620	novel_circ_0014617
hsa-miR-629-3p	novel_circ_0014617
hsa-miR-6501-5p	novel_circ_0014617
hsa-miR-6506-5p	novel_circ_0014617
hsa-miR-656-5p	novel_circ_0014617
hsa-miR-664a-3p	novel_circ_0014617
hsa-miR-665	novel_circ_0014617
hsa-miR-670-5p	novel_circ_0014617
hsa-miR-6763-5p	novel_circ_0014617

**miRNA**	**circRNA**
**miRNA**	**circRNA**
hsa-miR-105-5p	hsa_circ_0008378
hsa-miR-1294	hsa_circ_0008378
hsa-miR-133a-3p	hsa_circ_0008378
hsa-miR-133b	hsa_circ_0008378
hsa-miR-17-3p	hsa_circ_0008378
hsa-miR-224-5p	hsa_circ_0008378
hsa-miR-23a-3p	hsa_circ_0008378
hsa-miR-23b-3p	hsa_circ_0008378
hsa-miR-23c	hsa_circ_0008378
hsa-miR-2681-3p	hsa_circ_0008378
hsa-miR-29a-5p	hsa_circ_0008378
hsa-miR-3138	hsa_circ_0008378
hsa-miR-3160-5p	hsa_circ_0008378
hsa-miR-3611	hsa_circ_0008378
hsa-miR-3654	hsa_circ_0008378
hsa-miR-3689a-5p	hsa_circ_0008378
hsa-miR-3689b-5p	hsa_circ_0008378
hsa-miR-3689e	hsa_circ_0008378
hsa-miR-3689f	hsa_circ_0008378
hsa-miR-4315	hsa_circ_0008378
hsa-miR-4325	hsa_circ_0008378
hsa-miR-4505	hsa_circ_0008378
hsa-miR-4520-3p	hsa_circ_0008378
hsa-miR-4535	hsa_circ_0008378
hsa-miR-455-3p	hsa_circ_0008378
hsa-miR-4693-5p	hsa_circ_0008378
hsa-miR-4793-3p	hsa_circ_0008378
hsa-miR-486-5p	hsa_circ_0008378
hsa-miR-492	hsa_circ_0008378
hsa-miR-5002-5p	hsa_circ_0008378
hsa-miR-518a-3p	hsa_circ_0008378
hsa-miR-518b	hsa_circ_0008378
hsa-miR-518c-3p	hsa_circ_0008378
hsa-miR-518d-3p	hsa_circ_0008378
hsa-miR-518e-3p	hsa_circ_0008378
hsa-miR-518f-3p	hsa_circ_0008378
hsa-miR-544a	hsa_circ_0008378
hsa-miR-548aw	hsa_circ_0008378
hsa-miR-5589-5p	hsa_circ_0008378
hsa-miR-5787	hsa_circ_0008378
hsa-miR-606	hsa_circ_0008378
hsa-miR-6500-3p	hsa_circ_0008378
hsa-miR-6825-3p	hsa_circ_0008378
hsa-miR-7853-5p	hsa_circ_0008378
hsa-miR-7854-3p	hsa_circ_0008378
hsa-miR-943	hsa_circ_0008378

hsa-miR-6768-5p	novel_circ_0014617
hsa-miR-6770-5p	novel_circ_0014617
hsa-miR-6780a-3p	novel_circ_0014617
hsa-miR-6787-3p	novel_circ_0014617
hsa-miR-6800-3p	novel_circ_0014617
hsa-miR-6803-3p	novel_circ_0014617
hsa-miR-6821-3p	novel_circ_0014617
hsa-miR-6845-3p	novel_circ_0014617
hsa-miR-6861-3p	novel_circ_0014617
hsa-miR-6867-3p	novel_circ_0014617
hsa-miR-7113-3p	novel_circ_0014617
hsa-miR-7151-5p	novel_circ_0014617
hsa-miR-7843-3p	novel_circ_0014617
hsa-miR-8089	novel_circ_0014617
